# The 15‐Year Survival Advantage: Immune Resilience as a Salutogenic Force in Healthy Aging

**DOI:** 10.1111/acel.70063

**Published:** 2025-04-23

**Authors:** Muthu Saravanan Manoharan, Grace C. Lee, Nathan Harper, Justin A. Meunier, Marcos I. Restrepo, Fabio Jimenez, Sreenath Karekatt, Anne P. Branum, Alvaro A. Gaitan, Kian Andampour, Alisha M. Smith, Michael Mader, Michelle Noronha, Devjit Tripathy, Nu Zhang, Alvaro G. Moreira, Lavanya Pandranki, South Texas Veterans Health Care System (STVHCS) COVID‐19 Clinical team, Mohamed I. Abdalla, Sandra G. Adams, Yemi Adebayo, Joseph Agnew, Saleem Ali, Gregory Anstead, Antonio Anzueto, Marichu Balmes, Jennifer Barker, Raymond Benavides, Velma Bible, Angela Birdwell, Stacy Braddy, Stephen Bradford, Heather Briggs, Jose A. Cadena Zuluaga, Judith Marin‐Corral, Jennifer J. Dacus, Patrick J. Danaher, Scott A. DePaul, Jill Dickerson, Jollynn Doanne, Samantha Elbel, Miguel Escalante, Corina Escamilla, Valerie Escamilla, Robert Farrar, David Feldman, Debra Flores, Julianne Flynn, Delvina Ford, Joanna D. Foy, Megan Freeman, Samantha Galley, Jessica Garcia, Maritza Garza, Sherraine Gilman, Melanie Goel, Jennifer Gomez, Varun K. Goyal, Sally Grassmuck, Susan Grigsby, Joshua Hanson, Brande Harris, Audrey Haywood, Joan M. Hecht, Cecilia Hinojosa, Tony T. Ho, Teri Hopkins, Aneela N. Hussain, Ali Jabur, Pamela Jewell, Thomas B. Johnson, Austin C. Lawler, Monica Lee, Chadwick S. Lester, Stephanie M. Levine, Haidee V. Lewis, Angel Louder, Charmaine Mainor, Rachel Maldonado, Celida Martinez, Yvette Martinez, Chloe Mata, Neil McElligott, Laura Medlin, Myra Mireles, Joanna Moreno, Kathleen Morneau, Julie Muetz, Samuel B. Munro, Charlotte Murray, Anoop Nambiar, Daniel Nassery, Robert Nathanson, Kimberly Oakman, Jane O’Rorke, Cheryl Padgett, Sergi Pascual‐Guardia, Marisa Patterson, Graciela L. Perez, Rogelio Perez, Jay I. Peters, Rogelio Perez, Robert E. Phillips, Patrick B. Polk, Michael A. Pomager, Kristy J. Preston, Kevin C. Proud, Jacqueline A. Pugh, Michelle Rangel, Temple A. Ratcliffe, Renee L. Reichelderfer, Evan M. Renz, Jeanette Ross, Teresa Rudd, Maria E. Sanchez, Tammy Sanders, Kevin C. Schindler, David Schmit, Raj T. Sehgal, Claudio Solorzano, Nilam Soni, Win S. Tam, Edward J. Tovar, Sadie A. Trammell Velasquez, Anna R. Tyler, Anjuli Vasquez, Maria C. Veloso, Steven G. Venticinque, Jorge A. Villalpando, Melissa Villanueva, Lauren Villegas, Megan Walker, Andrew Wallace, Maria Wallace, Emily Wang, Stephanie Wickizer, Andreia Williamson, Andrea Yunes, Katharine H. Zentner, STVHCS COVID‐19 Vaccine team, Azaneth Arellanes, Ashley B. Banfield, Stephanie N. Bolan‐Reding, Roxanne Colazo, Katherine S. DeLeon, Norma G. Diaz, Mario A. Garza, Raed Kadhume, Hue Mang, Erwin Paleracio, Robert F. Quitta, Laura J. Ramirez, Marzieh Salehi, Cynthia J. Varela, STVHCS COVID‐19 Convalescent care team, STVHCS Center for Personalized Medicine, Andrew Carrillo, Chanda Dhami, Gaeun Jo, Krupa Jiva, Ernesto Robinson, Caitlyn A. Winter, Lauryn A. Winter, Erin Stewart, Joseph M. Yabes, Peter Melby, Cody R. Butler, Sandra Sanchez‐Reilly, Hanh D. Trinh, Clea Barnett, Luis Angel, Leopoldo N. Segal, Susannah Nicholson, Robert A. Clark, Weijing He, Jason F. Okulicz, Sunil K. Ahuja

**Affiliations:** ^1^ Veterans Affairs Center for Personalized Medicine South Texas Veterans Health Care System San Antonio Texas USA; ^2^ Department of Medicine University of Texas Health Science Center at San Antonio San Antonio Texas USA; ^3^ Pharmacotherapy Education and Research Center, School of Medicine University of Texas Health Science Center at San Antonio San Antonio Texas USA; ^4^ College of Pharmacy The University of Texas at Austin Austin Texas USA; ^5^ The Foundation for Advancing Veterans' Health Research San Antonio Texas USA; ^6^ Department of Microbiology, Immunology and Molecular Genetics University of Texas Health Science Center at San Antonio San Antonio Texas USA; ^7^ South Texas Veterans Health Care System San Antonio Texas USA; ^8^ Department of Pediatrics University of Texas Health Science Center at San Antonio San Antonio Texas USA; ^9^ Division of Pulmonary and Critical Care Medicine, Department of Medicine New York University Grossman School of Medicine, NYU Langone Health New York New York USA; ^10^ Department of Surgery University of Texas Health Science Center at San Antonio San Antonio Texas USA; ^11^ Gilead Sciences Foster City California USA

**Keywords:** accelerated aging, Alzheimer's disease, cardiac declines with age, inflammation, lifespan, longevity regulation, senescence, T cell

## Abstract

Human aging presents an evolutionary paradox: while aging rates remain constant, healthspan and lifespan vary widely. We address this conundrum via salutogenesis—the active production of health—through immune resilience (IR), the capacity to resist disease despite aging and inflammation. Analyzing ~17,500 individuals across lifespan stages and inflammatory challenges, we identified a core salutogenic mechanism: IR centered on *TCF7*, a conserved transcription factor maintaining T‐cell stemness and regenerative potential. IR integrates innate and adaptive immunity to counter three aging and mortality drivers: chronic inflammation (inflammaging), immune aging, and cellular senescence. By mitigating these aging mechanisms, IR confers survival advantages: At age 40, individuals with poor IR face a 9.7‐fold higher mortality rate—a risk equivalent to that of 55.5‐year‐olds with optimal IR—resulting in a 15.5‐year gap in survival. Optimal IR preserves youthful immune profiles at any age, enhances vaccine responses, and reduces burdens of cardiovascular disease, Alzheimer's, and serious infections. Two key salutogenic evolutionary themes emerge: first, female‐predominant IR, including *TCF7*, likely reflects evolutionary pressures favoring reproductive success and caregiving; second, midlife (40–70 years) is a critical window where optimal IR reduces mortality by 69%. After age 70, mortality rates converge between resilient and non‐resilient groups, reflecting biological limits on longevity extension. TNFα‐blockers restore salutogenesis pathways, indicating IR delays aging‐related processes rather than altering aging rates. By reframing aging as a salutogenic‐pathogenic balance, we establish *TCF7*‐centered IR as central to healthy longevity. Targeted midlife interventions to enhance IR offer actionable strategies to maximize healthspan before biological constraints limit benefits.

## Graphical abstract

Human aging shows puzzling diversity: similar aging rates yet vastly different health outcomes. Our study of ~17,500 people revealed a health‐promoting trait (more common in women) linked to strong immune resilience and high expression of *TCF7*, a key immune gene. This trait enables individuals to fight infections like COVID‐19 more effectively, respond better to vaccines, avoid comorbidities, and live longer. Its decline after age 70 may explain lifespan limits. These findings redefine aging as a tug‐of‐war between health‐promoting (salutogenic) and disease‐driving (pathogenic) processes, opening new treatment paths.

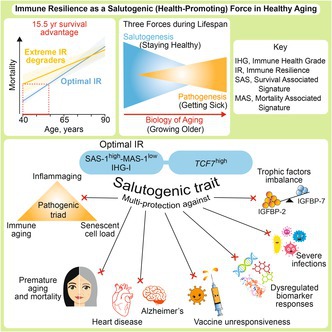

## 1 Introduction

Nothing in Biology Makes Sense Except in the Light of Evolution

Theodosius Dobzhansky, 1973

Environmental factors, particularly infections, have fundamentally shaped human evolution by selecting for protective inflammatory response mechanisms that enhance survival (Finch 2010; Liston et al. 2021; McDade 2023; Medzhitov 2021). This evolutionary pressure has created a core biological paradox: inflammation is indispensable for host defense, yet its dysregulation significantly heightens disease and mortality risk (Furman et al. 2019; Guo et al. 2022; Lopez‐Otin et al. 2023; Medzhitov 2021; Walker et al. 2022). This fundamental tension raises three fundamental questions about human aging and immunity: (1) How have selective pressures driven the evolution of mechanisms to balance inflammation's protective benefits against its harmful consequences? (2) Why does substantial variability in healthspan persist despite historically stable rates of aging? (3) Does evolutionary prioritization of reproductive fitness inherently limit longevity?

To address these questions, we developed an integrated evolutionary framework comprising four interconnected dimensions. At its foundation lies immune robustness—the ability to neutralize pathogenic threats while minimizing collateral tissue damage. This capability represents a critical evolutionary adaptation balancing protection against immediate threats with long‐term tissue integrity. The remaining dimensions include (1) inflammatory stressors (environmental challenges that activate immune responses), (2) salutogenesis (health‐promoting processes derived from Latin roots meaning “the origin of health”—”salus” meaning “health”), (Becker et al. 2010), and (3) immune resilience (the dynamic capacity to respond to and recover from immunological challenges).

When immune robustness fails, it triggers what we term the “pathogenic triad”—three interconnected processes that accelerate biological aging: (1) inflammaging (sterile, chronic low‐grade inflammation), (2) immune senescence (progressive impairment of innate and adaptive immunity), and (2) accumulation of senescent cells through Senescence‐Associated Secretory Phenotype‐driven damage (Guo et al. 2022; Liu et al. 2023; Lopez‐Otin et al. 2023; Walker et al. 2022). Importantly, these processes do not simply correlate with aging—they actively accelerate age‐related morbidity independent of chronological age and mirror the established molecular hallmarks of organismal aging. Environmental triggers can initiate this triad, thereby elevating the risk of infection, multimorbidity, and mortality (Guo et al. 2022; Liu et al. 2023; Lopez‐Otin et al. 2023; Miller et al. 2024; Walker et al. 2022).

**FIGURE 1:**
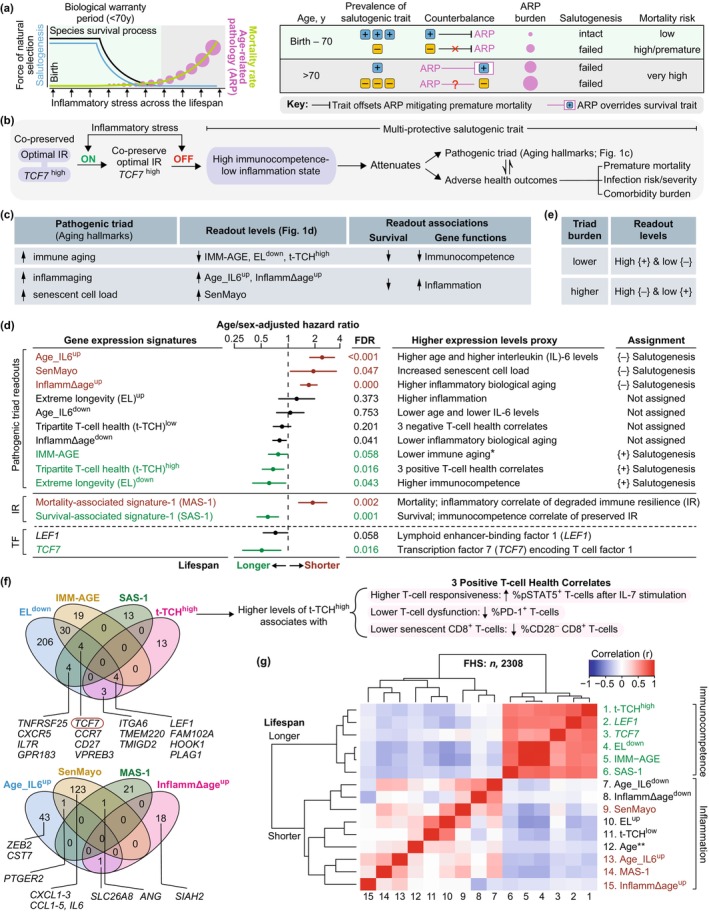
Models and transcriptomic metrics of immune resilience (IR) and the components of the pathogenic triad. (a) Model: Evolution, salutogenesis, salutogenic traits, and aging. + and −, survival‐associated trait (blue) versus mortality‐associated trait (yellow), respectively. (b) Model and hypothesis. Multi‐protective salutogenic trait associated with co‐preservation of optimal IR and higher levels of transcription factor 7 (*TCF7* ^*high*^; expression level > median value) levels during inflammatory stress. (c) Gene expression signatures tracking the pathogenic triad (termed readouts) associated with lifespan in panel d. (d) Association of the readouts, transcriptomic metrics of IR [Survival‐associated signature (SAS)‐1; Mortality‐associated signature (MAS)‐1], and transcription factors (TF) with lifespan in the Framingham Heart Study (FHS). Age‐ and sex‐adjusted hazard ratios of all‐cause mortality by Cox proportional hazards models. False discovery rates (FDR) by Benjamini‐Hochberg method. {+}, less immune aging (positive salutogenesis); {−}, increased inflammaging or senescent load (negative salutogenesis). *, Higher levels of the IMM‐AGE signature were computed to signify an association with fewer senescent T‐cells (less immune aging and lower mortality; a {+}‐salutogenesis readout), as detailed in Section 4.2 of the Supporting Information. (e) Triad burden scoring. (f) Overlaps of genes in (*top*) {+}‐salutogenesis readouts and SAS‐1 and (*bottom*) {−}‐salutogenesis readouts and MAS‐1. *Right*, attributes of the t‐TCH^high^ signature. pSTAT5, phosphorylated STAT5; IL‐7, interleukin 7; PD‐1, programmed cell death protein 1. (g) Correlation and clustering of signatures, TFs, and age in the FHS. Color gradient, Pearson's correlation coefficient (r). The cluster marked as longer lifespan contained gene signatures linked to immunocompetence, and the cluster marked as shorter lifespan contained gene signatures linked to inflammation. Specifically, the green‐colored vs. red‐colored gene signatures associated with longer vs. shorter lifespan respectively, as shown in panel 1d. ** indicates chronological age.

Our framework posits that immune robustness—shaped by evolutionarily optimized strategies—provides the foundation for salutogenesis, supporting systemic resilience by counteracting the aging hallmarks encompassed in the pathogenic triad. These salutogenic mechanisms mitigate age‐related pathologies and extend lifespan through what is conceptualized as a “biological warranty period” encompassing both reproductive and post‐reproductive phases (Carnes et al. 2012; Carnes and Witten 2014; Farrelly 2024) (Figure 1a). This warranty period closely aligns with the 2024 global average life expectancy of 73.4 years (“Life Expectancy by Country 2024,” 2024). We propose that premature mortality (before approximately 70 years) likely reflects a failure to sustain salutogenic adaptations rather than representing inherent biological constraints of aging.

The uniquely human extension of post‐reproductive lifespan enhances offspring survival via intergenerational caregiving mechanisms, often termed the ‘Grandmother hypothesis’—representing a key evolutionary driver of longevity that distinguishes human aging patterns from those of other primates (Cant and Croft 2019; Hawkes and Coxworth 2013). This perspective directly addresses our third question by suggesting that humans evolved specific mechanisms to maintain health beyond reproductive years, providing evolutionary advantages through extended care for descendants.

To empirically investigate these concepts, we conducted longitudinal multi‐omics profiling in approximately 17,500 participants exposed to diverse inflammatory challenges across the lifespan, from birth to over 90 years of age. We specifically mapped immune resilience (IR) trajectories and the emergence of the pathogenic triad across health‐to‐disease transitions. We focused on IR metrics because optimal IR requires two essential, evolutionarily conserved capabilities: (1) maintaining immunocompetence (effective antimicrobial defenses) and (2) controlling inflammation during immunological stress (Ahuja et al. 2023; Lee et al. 2021). These interdependent functions confer distinct survival advantages during inflammatory challenges, directly addressing our first question about evolutionary balancing mechanisms.

Our findings demonstrate that maintaining optimal IR with elevated transcription factor 7 (*TCF7*) levels—which we designate as optimal IR‐*TCF7* ^high^—establishes a clinically actionable salutogenic trait (Figure 1b and Figure S1). This *TCF7*‐associated trait significantly reduces the emergence of the pathogenic triad, thereby mitigating age‐associated immunopathology manifested as increased infection risk and severity, comorbidities such as cardiovascular disease, and mortality. Mechanistically, *TCF7* encodes TCF1, an evolutionarily conserved master regulator of T‐cell immunity and stemness (Sturmlechner et al. 2023; Zhao et al. 2022). Through genome‐wide screening of 1380 transcription factors, we identified a *TCF7*‐centered regulatory network governing IR mechanisms, alongside six co‐regulated factors. This finding helps explain the substantial variability in healthspan despite stable aging rates—our second key question—by identifying specific biological mechanisms that can vary between individuals.

Our findings provide three key translational insights. First, we demonstrate that the pathogenic triad stems from compromised IR integrity during lifelong stress responses—not simply from chronological aging itself. Second, our framework establishes health‐promoting biological processes (salutogenesis) as fundamental to extending disease‐free lifespan and longevity. This is particularly evident during the biological warranty period through the maintenance of dynamic equilibrium between pathology resistance and senescence pathways, a theory first proposed by Hayflick and Vaupel (Hayflick 2010; Vaupel 2010). Third, beyond a high immunocompetence‐low inflammation status, we show that IR‐*TCF7* ^high^ status directly connects with the components of the insulin‐like growth factor (IGF) system—a highly evolutionarily conserved signaling pathway—which has strong ties to aging phenotypes and longevity (Conover and Oxvig 2024; Salminen et al. 2021). Together, we propose a new paradigm in aging research that distinguishes between treating age‐related diseases and modifying the aging process itself through IR‐associated salutogenic mechanisms. This framework identifies targeted strategies to recalibrate immune resilience, paving the way for healthier longevity.

## 2 Results

### 2.1 Study Design, Definitions, and Outcomes

We developed a three‐stage analytical framework to evaluate the relationship between IR status and pathogenic triad, and to assess its clinical relevance. In Stage 1, we quantify IR through two established metrics: first, by measuring the balance between peripheral blood CD8^+^ and CD4^+^ T‐cells, classified into immune health grades (IHGs), and second, through profiling IR‐associated gene expression signatures that independently predict survival outcomes, as validated in prior studies (Ahuja et al. 2023; Lee et al. 2021).

In Stage 2, we assessed triad burden (pathogenic triad) by analyzing peripheral blood gene expression signatures (Figure 1c,d). Each signature had to meet three key requirements (Figure 1c): quantify pathogenic triad components through expression levels, distinguish lifespan groups through expression clustering (longer vs. shorter), and prioritize immunocompetence‐ or inflammation‐related genes. We grouped these signatures as either {+}‐ or {−}‐salutogenesis readouts (Figure 1d). {+}‐salutogenesis readouts met three benchmarks: elevated expression linked to slower immune aging, connection to extended lifespan, and reliance on immunocompetence‐related genes (Figure 1c,d). In contrast, {−}‐salutogenesis readouts showed increased inflammaging/senescent cell burden, ties to shorter lifespan, and contained more inflammation‐related genes (Figure 1c,d).

In Stage 3, we investigated associations between IR metrics and salutogenic indicators, with a focus on their relationships to clinical health outcomes and immune‐inflammatory biomarker profiles (Figure 1b, right) in both COVID‐19 and non‐COVID‐19 cohorts [please see Methods and Supporting Information for cohort details].

### 2.2 Readouts of the Pathogenic Triad: Selection and Characterization

We initially evaluated ten candidate gene expression signatures as potential measures of triad burden, with six meeting predefined salutogenesis readout criteria (Figure 1c–e; derivation details in Figure 1d; Tables S1 and S2 and Supporting Information). Of these, three signatures—Age_IL6^up^, InflammΔage^up^, and SenMayo—emerged as robust {−}‐salutogenesis readouts. Elevated expression of these signatures corresponded to greater inflammaging (Age_IL6^up^, InflammΔage^up^) and senescent cell burden (SenMayo) and demonstrated significant associations with reduced lifespan via higher age‐ and sex‐adjusted hazard ratios (HRs) for 9‐year all‐cause mortality in the Framingham Heart Study (FHS) (Figure 1c,d). Age_IL6^up^ and InflammΔage^up^ were developed through FHS biomarker analyses (Lin et al. 2017; Pilling et al. 2015), while the SenMayo signature quantifies senescent cell load across tissues and species and decreases after senescent cell clearance in mice and humans (Saul et al. 2022). These signatures, which comprise inflammation‐related genes, cluster together (Figure 1f,g; Figure S2 and Table S2).

The IMM‐AGE, EL^down^, and t‐TCH^high^ signatures met the key criteria for {+}‐salutogenesis readouts (Figure 1c,d). Elevated expression levels of these signatures are associated with attenuated immune aging and predict longer lifespan (evidenced by lower age‐ and sex‐adjusted HRs; Figure 1c,d). These immunocompetence‐related signatures formed a tightly correlated cluster (Figure 1f,g; Figure S2; Tables S1 and S2), suggesting coordinated regulation. The IMM‐AGE signature—a validated transcriptomic measure of T‐cell senescence in the FHS (Alpert et al. 2019)—was calculated such that higher levels signify fewer senescent T‐cells, reflecting reduced immune aging and lower mortality risk (Figure 1d). EL^down^ comprises immunocompetence‐related genes exhibiting reduced expression in individuals with extreme longevity (EL) compared to younger controls, as identified through single‐cell RNA‐seq data (Karagiannis et al. 2023). The t‐TCH^high^ (t‐TCH, tripartite T‐cell health) signature (Figure 1f)—developed by our team—integrates genes whose expression profiles correlate with three independent biomarkers of T‐cell health. These include biomarkers associated with STAT5 phosphorylation in T‐cells following IL‐7 stimulation, a vital functional response underpinning immune cell survival and activity (Camargo et al. 2009). While the InflammΔage^down^ signature is also associated with longer lifespan (Figure 1d), it exhibits divergent clustering behavior, grouping with pro‐inflammatory signatures linked to higher mortality (Figure 1g), and thus does not fully satisfy {+}‐salutogenesis readout criteria.

In conclusion, our study demonstrates that higher expression of positive salutogenesis readouts (IMM‐AGE, EL^down^, t‐TCH^high^) alongside lower expression of negative salutogenesis readouts (Age_IL6^up^, InflammΔage^up^, SenMayo) predicts a lower triad burden, whereas the inverse pattern forecasts a higher triad burden (Figure 1c,e). This work establishes a quantitative framework for evaluating the triad burden and its clinical implications in aging and longevity trajectories.

### 2.3 Transcriptomic Metrics of Immune Resilience (IR)

Transcriptomic metrics of IR, specifically Survival‐associated signature‐1 (SAS‐1) and Mortality‐associated signature‐1 (MAS‐1), reveal distinct biological insights [Figure 1c; Tables S1 and S2; (Ahuja et al. 2023; Lee et al. 2021)]. While salutogenesis readouts directly associate with narrowly defined factors like inflammaging/immune aging, SAS‐1 and MAS‐1 emerged from robust concordance with survival and mortality in two independent cohorts: acute COVID‐19 (30‐day, all‐cause mortality) and the FHS (9‐year, all‐cause mortality) (Ahuja et al. 2023; Lee et al. 2021). This broader relevance across disease contexts, compared to salutogenesis readouts, positions these metrics as potential determinants of pathogenic triad severity.

### 2.4 Unique vs. Shared Features of Metrics of IR and the Triad Burden

The analysis of unique and shared features among transcriptomic markers of IR and triad burden uncovered distinct molecular patterns. *TCF7* emerged as a component of SAS‐1 and two {+}‐salutogenesis readouts (EL^down^ and IMM‐AGE; Figure 1f). Its homolog, *LEF1* (Zhao et al. 2022) appears in all three {+}‐salutogenesis readouts but not in SAS‐1 (Figure 1f). Notably, higher *TCF7* expression associates with longer lifespan, while *LEF1* demonstrates a weaker association (Figure 1d). Markers linked to immunocompetence and survival (*TCF7*, *LEF1*, SAS‐1, and {+}‐salutogenesis readouts) converge into a tightly correlated cluster, particularly for IMM‐AGE and EL^down^, which exhibit 38 shared genes (Figure 1g and Figure S2). In contrast, markers linked to inflammation/mortality (MAS‐1, {−}‐salutogenesis readouts) occupy distinct clusters, lack shared transcription factors or substantial gene overlap, and show low inter‐correlations (Figure 1f,g and Figure S2 and Table S2). This divergence suggests distinct regulatory mechanisms underlie immunocompetence/survival and inflammation/mortality pathways, indicating their independent regulation.

The weak age correlation with *TCF7*, SAS‐1, MAS‐1, and salutogenesis readouts (Figure 1g and Figure S2) may partially explain their age‐independent associations with survival/mortality (Figure 1d). These findings imply that lifespan may be influenced by mechanisms distinct from chronological aging, providing new insights into differentiating between age‐dependent and age‐independent drivers of IR and the pathogenic triad.

### 2.5 Expression Patterns of Signatures in Immune Cell Subsets: Single‐Cell Analyses

**FIGURE 2:**
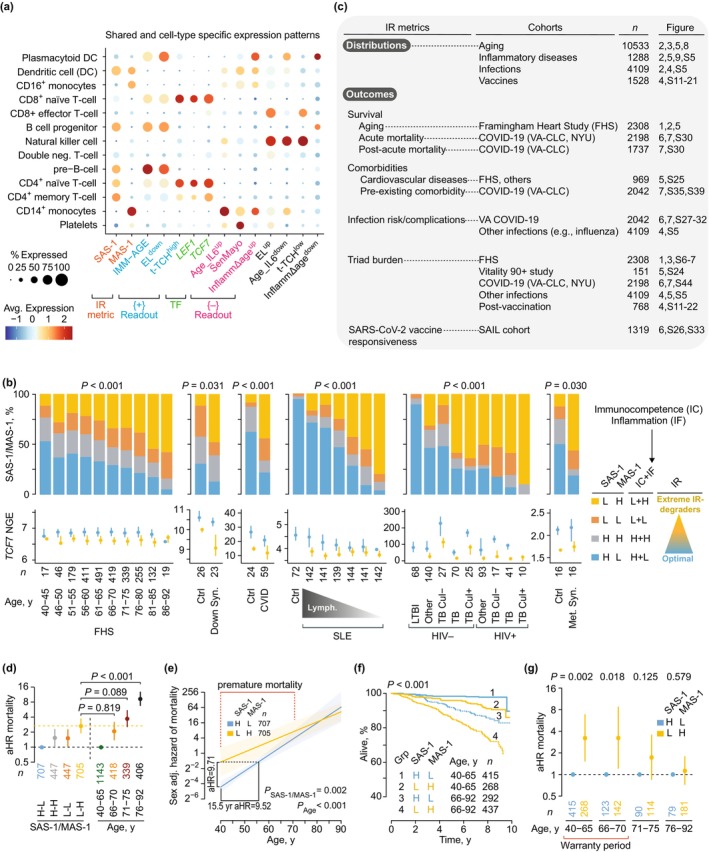
Immune resilience (IR) metrics and associations. (a) Expression of gene signatures and transcription factor genes noted in Figure 1d in single‐cell RNA‐seq‐defined peripheral blood cell populations. MAS‐1, Mortality‐associated signature‐1; SAS‐1, Survival‐associated signature‐1; *TCF7*, transcription factor 7. (b) *Top*, distribution of combined SAS‐1 and MAS‐1 expression (median‐based strata: H, high [expression levels > median value]; L, low [expression levels ≤ median value]) in cohorts. *Bottom*, *TCF7* normalized gene expression [NGE, median (IQR)]. Cohorts (accession numbers): Framingham Heart Study (FHS) (phs000007.v30.p11 and phs000363.v17.p11); Down syndrome (Syn.) (GSE183071); common variable immunodeficiency (CVID) (GSE51405); systemic lupus erythematosus (SLE) (GSE65391) by sextiles of lymphocyte (lymph.) levels; HIV, latent tuberculosis infection (LTBI), TB with positive (+) or negative (−) culture (Cul) status (GSE39941); and metabolic syndrome (Met. Syn.) (GSE145412). *p*, by χ^2^ and Fisher's exact tests. Ctrl, controls. (c) Cohorts: SAIL, San Antonio Immunologic Resilience Longitudinal. NYU, New York University. VA‐CLC, Veterans Affairs COVID‐19 Longitudinal Cohort. (d–g) Cohort: FHS. (d) Sex‐adjusted hazard ratios (aHR) of mortality by SAS‐1/MAS‐1 profiles and age strata. (e) Sex‐adjusted (adj.) mortality hazards. Shaded areas, 95% confidence bands. The dotted lines indicate that the sex‐adjusted mortality hazards of a 40‐year‐old individual with SAS‐1^low^‐MAS‐1^high^ and a 55‐year‐old person with SAS‐1^high^‐MAS‐1^low^ are similar. (f) Time to death in groups defined by SAS‐1/MAS‐1 profiles and age. *p*, by log‐rank test. Grp, group. (g) Sex‐adjusted HRs of mortality with a 95% confidence interval (CI) in groups defined by SAS‐1/MAS‐1 profiles and age. *p* in panels d, e, and g by Cox proportional hazards models.

To elucidate the cellular drivers of IR markers, we profiled their expression across immune cell subsets. By integrating single‐cell RNA sequencing data (Figure S3) with flow‐sorted T‐cell subset analyses (Figure S4), we established cell type‐specific expression patterns for IR‐associated signatures. SAS‐1 co‐expressed with {+}‐salutogenesis pathways and *TCF7* displayed distinct expression patterns with higher levels in dendritic cells and lower expression in naïve CD8^+^ T‐cells (Figure 2a). Conversely, MAS‐1 showed preferential enrichment in CD14^+^ and CD16^+^ monocyte populations (Figure 2a). Notably, NK cell‐enriched signatures (EL^up^, Age_IL6^down^, t‐TCH^low^; Figure 2a) showed no association with lifespan (Figure 1d), suggesting NK cell activity does not primarily determine longevity in this context. These findings reveal that immunocompetence/survival‐linked and inflammation/mortality‐associated gene signatures exhibit fundamental differences in their constituent genes (Figure 1f), correlation networks (Figure 1g and Figure S2), and spatial distribution across cell types (Figure 2a; Figures S3 and S4). Our single‐cell RNA analysis establishes a framework for understanding IR's biological mechanisms.

### 2.6 SAS‐1/MAS‐1 Combinations: Gauges of Optimal IR vs. Extreme IR Degradation

SAS‐1 and MAS‐1 contain genes associated with immunocompetence and inflammation, respectively [Table S2; (Ahuja et al. 2023)]. Optimal IR corresponds to a high immunocompetence–low inflammation state [Figure 2b, far right; (Ahuja et al. 2023; Lee et al. 2021)], prompting us to categorize SAS‐1 and MAS‐1 levels into four distinct IR profiles. Elevated SAS‐1 paired with reduced MAS‐1 (SAS‐1^high^‐MAS‐1^low^) reflects optimal IR status, marked by robust immunocompetence and minimal inflammation. In contrast, other combinations signal suboptimal IR states [Figure 2b, far right; (Ahuja et al. 2023)]. Notably, the SAS‐1^low^‐MAS‐1^high^ profile reflects extreme IR degradation, featuring low immunocompetence and high inflammation (Figure 2b, far right).

The SAS‐1/MAS‐1 profiles demonstrate strong predictive power for longevity. In age‐ and sex‐adjusted analyses of the FHS cohort, our data established a clear lifespan hierarchy: SAS‐1^high^‐MAS‐1^low^ > SAS‐1^high^‐MAS‐1^high^ ≈ SAS‐1^low^‐MAS‐1^low^ > SAS‐1^low^‐MAS‐1^high^ (Ahuja et al. 2023). These findings underscore that preserving robust immunocompetence while minimizing inflammation is critical for maximizing longevity potential. To further validate these patterns, we investigated the distribution patterns and clinical relevance of optimal IR (SAS‐1^high^‐MAS‐1^low^) and extreme IR degradation (SAS‐1^low^‐MAS‐1^high^) across cohorts (Figure 2c), assessing their alignment with our conceptual framework (Figure 1a,b).

### 2.7 Optimal IR Restricts Premature Mortality

The median age of FHS participants was 66 years (IQR: 60–73). Figure 2d (right) demonstrates age's pronounced hierarchical impact on mortality risk: compared to younger individuals (40–65 years), the oldest group (76–92 years) exhibited a nearly ninefold higher sex‐adjusted mortality HR. Strikingly, the mortality risk difference between SAS‐1^low^‐MAS‐1^high^ and SAS‐1^high^‐MAS‐1^low^ was substantial (aHR ~3), paralleling the elevated risk observed when comparing adults aged 66–70 y or 71–75 y to the 40–65 y reference group (Figure 2d). Further analyses revealed two key patterns (Figure 2e–g): (1) immune resilience delays aging‐related mortality effects rather than altering intrinsic aging rates and (2) survival advantages linked to SAS‐1^high^‐MAS‐1^low^ were largely restricted to individuals under 70 years, consistent with the proposed “biological warranty period” (Figure 1a).

First, mortality hazards were persistently higher in participants with SAS‐1^low^‐MAS‐1^high^ versus SAS‐1^high^‐MAS‐1^low^; however, this disparity attenuated among individuals aged over 70 years (Figure 2e). Our modeling demonstrated a striking equivalence: when compared to 40‐year‐olds with SAS‐1^high^‐MAS‐1^low^, 40‐year‐olds with SAS‐1^low^‐MAS‐1^high^ exhibited a significantly higher sex‐adjusted mortality hazard ratio (aHR = 9.71). Moreover, the mortality risk of these 40‐year‐olds with SAS‐1^low^‐MAS‐1^high^ was statistically indistinguishable (*p* = 0.970) from that of individuals aged 55.5 years with SAS‐1^high^‐MAS‐1^low^ (Figure 2e). Thus, our analysis indicates that 40‐year‐olds with extreme IR degradation (SAS‐1^low^‐MAS‐1^high^) versus those with optimal IR (SAS‐1^high^‐MAS‐1^low^) exhibit a 15.5‐year life expectancy disparity.

Second, individuals with the SAS‐1^low^‐MAS‐1^high^, compared to SAS‐1^high^‐MAS‐1^low^ individuals, had a pronounced 3.0‐fold higher aHR for mortality among those aged 40–65 years, a 3.25‐fold elevated risk (*p* = 0.018) in the 66–70 age group, and a 1.74‐fold increase (*p* = 0.125) for those aged 71–75 years (Figure 2f,g, Table S3). Notably, adults aged 40–70 years with SAS‐1^high^‐MAS‐1^low^ exhibited a 69% lower mortality hazard than those with SAS‐1^low^‐MAS‐1^high^ [sex‐adjusted HR = 0.31 (95% CI: 0.15–0.65); *p* = 0.002]. In contrast, mortality risks in the oldest cohort (76–92 years) showed no significant differences across SAS‐1/MAS‐1 profiles (Figure 2g). Thus, the survival benefits of optimal IR status (SAS‐1^high^‐MAS‐1^low^) are primarily observed in mid‐adulthood (40–70 years).

### 2.8 Extreme IR Degradation: A Consequence of Age vs. Inflammatory Stress?

While the prevalence of SAS‐1^low^‐MAS‐1^high^ increases with age (Figure 2b, left panel and Figure S5a), our findings indicate that inflammatory stress—rather than aging itself—primarily drives this phenotypic shift. Three key lines of evidence reinforce this interpretation: First, the overrepresentation of SAS‐1^low^‐MAS‐1^high^ and underrepresentation of SAS‐1^high^‐MAS‐1^low^ in older versus younger individuals persists across all age groups experiencing inflammatory or immunodeficiency conditions (Figure 2b and Figure S5). Notably, this pattern emerges in four distinct contexts: (i) Conditions sharing immunological and phenotypic hallmarks of aging—termed “accelerated aging”—such as Down syndrome, common variable immunodeficiency, systemic lupus erythematosus, and HIV (Chou et al. 2013; Gensous et al. 2020; Kalim et al. 2020; Odnoletkova et al. 2018; Walker et al. 2022) (Figure 2b and Figure S5b); (ii) Infections with pathogens exerting long‐term evolutionary pressure on humans, such as tuberculosis (Figure 2b and Figure S5c), malaria (*Plasmodium falciparum* infection; Figure S5d,e), and dengue (Figure S5f); (iii) Acute inflammatory states such as sepsis and Kawasaki disease (Figure S5g); and (iv) Chronic infections, metabolic syndrome (independent of obesity; Figure 2b and Figure S5h), and immune tolerance pathways (Figure S5h).

Second, the prevalence of SAS‐1^low^‐MAS‐1^high^ correlates directly with inflammatory stress severity, as evidenced through disease progression metrics, infection burden (e.g., malaria parasite density), treatment outcomes (e.g., tuberculosis cure rates), and biomarker profiles (lymphopenia, neutrophilia, inflammatory mediators) (Figure 2b and Figure S5).

Third, resolution of inflammatory stress triggers a phenotypic shift from a SAS‐1^low^‐MAS‐1^high^ to a SAS‐1^high^‐MAS‐1^low^ profile. This transition is observed during tuberculosis treatment (Figure S5c) and dengue recovery (Figure S5f), and in association with acute inflammatory resolution phases (Figure S5g). Together, our findings establish inflammatory stress—rather than chronologic age alone—as the primary driver of the SAS‐1^low^‐MAS‐1^high^ profile.

These observations yield four key implications. First, SAS‐1^low^‐MAS‐1^high^ identifies a reversible, highly immunodeficient‐proinflammatory state across age groups. Second, SAS‐1^low^‐MAS‐1^high^ in older adults reflects a lifetime of cumulative inflammatory stress, mirroring profiles observed in younger individuals with “accelerated aging” features. Third, mortality patterns align with the evolutionary model proposed in Figure 1a: maintaining optimal IR status (SAS‐1^high^‐MAS‐1^low^) during the warranty period represents an evolutionarily conserved salutogenic trait linked to survival, whereas susceptibility to extreme IR‐degrader status (SAS‐1^low^‐MAS‐1^high^) heightens vulnerability to premature mortality (Figure 2e,g). Fourth, during the post‐warranty period, age‐related pathologies overwhelm the survival advantages of SAS‐1^high^‐MAS‐1^low^ (Figure 2g), suggesting a critical threshold beyond which optimal IR's protective capacity wanes. To investigate causal relationships, we examined whether the survival benefits of SAS‐1^high^‐MAS‐1^low^ stem from reducing pathogenic triad burden, independent of age.

### 2.9 Extreme IR Degradation Tracks a Higher Triad Burden Regardless of Age

**FIGURE 3:**
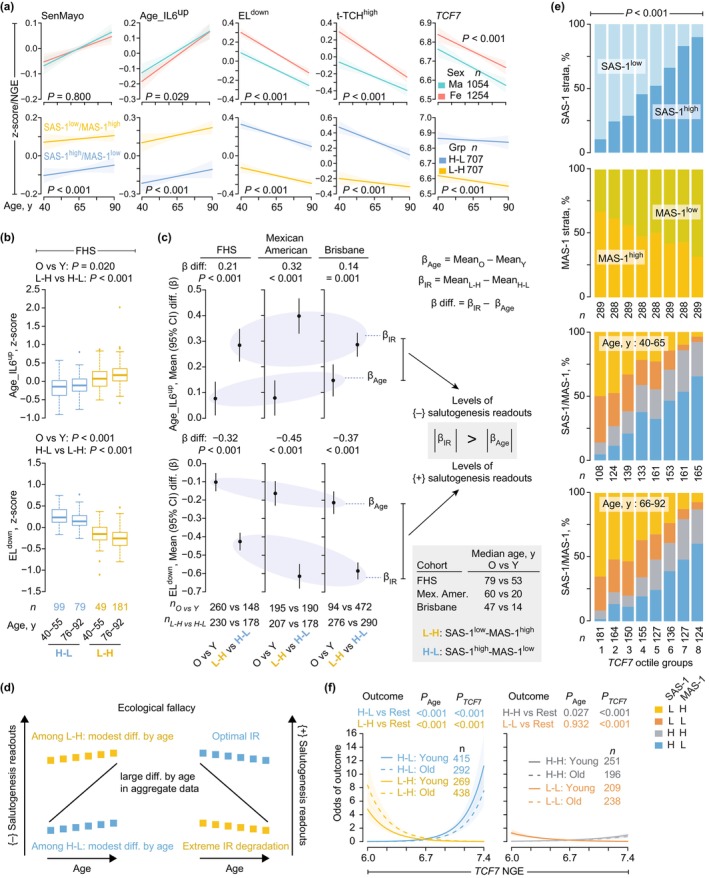
Associations of the transcriptomic metrics of immune resilience (IR), {+} and {−} salutogenesis readouts of the pathogenic triad (readouts), and *TCF7* (transcription factor 7). Panels a, b, e, and f from the Framingham Heart Study (FHS). (a) Linear regression modeling of the levels of the readouts (z‐scores) noted in Figure 1c and *TCF7* [normalized gene expression (NGE)] with 95% confidence bands across age by (*top*) sex and (*bottom*) profiles of the combination of the Survival‐associated signature‐1 (SAS‐1) and Mortality‐associated signature‐1 (MAS‐1) expression levels (median‐based strata: H, high; L, low; H‐L, SAS‐1^high^‐MAS‐1^low^; L‐H, SAS‐1^low^‐MAS‐1^high^). Grp, group. (b) Median (IQR) expression of indicated readouts. *p*, by linear model with likelihood ratio test (LRT). (c) Mean difference (diff., β values) with 95% confidence interval (CI) of readouts by age [older (O) vs. younger (Y)] and IR status (SAS‐1^low^‐MAS‐1^high^ vs. SAS‐1^high^‐MAS‐1^low^ tracking extreme IR degrader vs. optimal IR status, respectively) in the (*left*) FHS, (*middle*) Mexican Americans (E‐TABM‐305; Mex. Amer.), and (*right*) Brisbane (GSE53195) cohorts computed by linear models. *p*, by LRT. Median age is for persons in O vs. Y groups with the following cohort‐wise age cut‐offs: FHS: ≥ 76 versus < 56; Mexican Americans: ≥ 50 versus < 25; Brisbane: ≥ 40 versus < 20. (d) Ecological fallacy schema. (e) Distribution of SAS‐1 and MAS‐1 expression strata and SAS‐1/MAS‐1 profiles by octiles of *TCF7* (NGE) and age groups. *p*, by *χ* ^2^ test. (f) Odds of the SAS‐1/MAS‐1 profiles (H‐L, SAS‐1^high^‐MAS‐1^low^; L‐H, SAS‐1^low^‐MAS‐1^high^; H‐H, SAS‐1^high^‐MAS‐1^high^; L‐L, SAS‐1^low^‐MAS‐1^low^) among younger and older persons by *TCF7* (NGE). Shaded regions, 95% confidence bands. *p*, by logistic regression.

With advancing age, {−}‐salutogenesis readout levels rise as {+}‐salutogenesis readouts decline (Figure 3a, top and Figure S6a). Our analyses revealed greater disparities in triad burden and *TCF7* levels between IR status groups (SAS‐1^low^‐MAS‐1^high^ vs. SAS‐1^high^‐MAS‐1^low^) than age‐related differences (older vs. younger) (Figure 3a–c). Notably, regardless of age, individuals with SAS‐1^high^‐MAS‐1^low^ profiles displayed elevated *TCF7* and {+}‐salutogenesis readouts, whereas those with SAS‐1^low^‐MAS‐1^high^ profiles exhibited higher {−}‐salutogenesis levels [Figures 2b and 3a (bottom row), and Figure S6b]. Triad burden increased only marginally with age within both SAS‐1^high^‐MAS‐1^low^ and SAS‐1^low^‐MAS‐1^high^ groups (Figure 3b and Figure S7). Strikingly, β differences in triad burden were more pronounced between IR status groups than between age groups separated by 2–3 decades (Figure 3c and Figure S7).

These findings indicate that triad burden exemplifies the ecological fallacy (Piantadosi et al. 1988), where population‐level trends fail to capture individual‐level relationships (Figure 3d). We observed sex‐specific differences in gene expression, with females showing higher levels of transcriptomic markers associated with immunocompetence and survival (Figure 3a and Figure S6a). These results—replicated across three independent cohorts and different age ranges—highlight the robustness of our findings and underscore the critical role of individual variations in IR status and sex in shaping immunological health trajectories across the aging process.

### 2.10 *TCF7* Levels Sustain Optimal IR (SAS‐1^high^‐MAS‐1^low^)

What determinants maintain optimal IR (SAS‐1^high^‐MAS‐1^low^)? Since *TCF7* is a component of SAS‐1 (Figure 1f) and transcription factors regulate gene expression (Lambert et al. 2018), we hypothesized that elevated *TCF7* expression sustains SAS‐1^high^‐MAS‐1^low^. Supporting this, higher *TCF7* levels (*TCF7* ^high^) correlated with higher prevalence (Figure 3e) and greater odds (Figure 3f) of SAS‐1^high^‐containing profiles (SAS‐1^high^‐MAS‐1^low^, SAS‐1^high^‐MAS‐1^high^). Conversely, lower *TCF7* levels were associated with higher prevalence and odds of SAS‐1^low^‐containing profiles (Figure 3e–f). Dose–response patterns between *TCF7* expression and SAS‐1^high^‐MAS‐1^low^ prevalence emerged consistently in younger (40–65 years) and older (66–92 years) cohorts (Figure 3e–f). In most datasets, *TCF7* levels remained elevated in SAS‐1^high^‐MAS‐1^low^ individuals versus SAS‐1^low^‐MAS‐1^high^ counterparts, regardless of age or disease status (Figure 2b, bottom row; Figure S5). The co‐occurrence of *TCF7* ^high^ and optimal IR metrics defines a survival‐associated salutogenic trait, independent of age or inflammatory stress. This positions *TCF7* as a mechanistic regulator of optimal IR maintenance.

### 2.11 Multi‐Layered Protection Associated With Optimal IR‐ *TCF7* ^*high*^ : Case Studies

**FIGURE 4:**
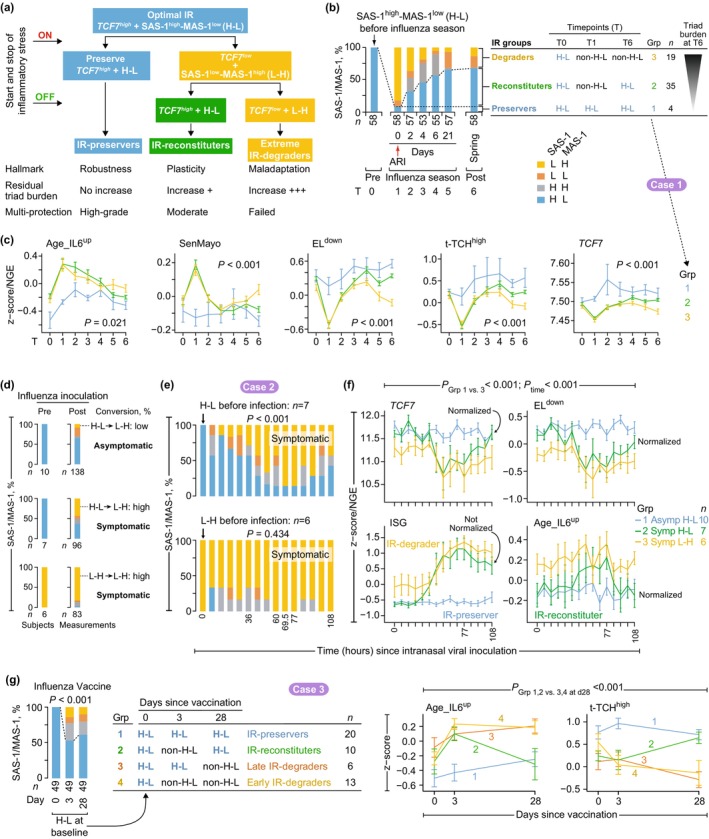
Immune resilience (IR) responses during inflammatory stress. (a) Persons with an optimal IR metric [Survival‐associated signature‐1 (SAS‐1)^high^‐Mortality‐associated signature‐1 (MAS‐1)^low^] and higher levels of transcription factor 7 (*TCF7*) are categorized as IR‐preservers, IR‐reconstituters, and IR‐degraders during inflammatory stress. (b, c) Case study 1: Viral infection during influenza season (GSE68310). (b) *Left*, Distribution of the SAS‐1/MAS‐1 profiles at the timepoints (T) pre‐, during, and post‐influenza season among those with SAS‐1^high^‐MAS‐1^low^ (H‐L) profile pre‐infection (T0). ARI, acute respiratory infection. Profiles by combinations of SAS‐1 and MAS‐1 expression levels [median‐based strata: H, high; L, low]. *Right*, IR groups (Grp) based on SAS‐1/MAS‐1 profiles at T0, T1, and T6. (c) Trajectories (mean ± SE) of the gene signatures tracking the pathogenic triad (readouts in Figure 1c) and normalized gene expression (NGE) of *TCF7* by IR groups in panel b. *p*, by ANOVA at T6. (d–f) Case study 2: Influenza challenge cohort (GSE52428). (d, e) SAS‐1/MAS‐1 profiles (d) pre‐ and post‐virus inoculation and by symptom status [post‐inoculation profiles pooled into a single stacked barplot] and (e) at post‐inoculation timepoints in symptomatic persons with H‐L and L‐H pre‐inoculation. *p*, by Fisher's exact test. H‐L: SAS‐1^high^‐MAS‐1^low^; L‐H: SAS‐1^low^‐MAS‐1^high^. (f) Trajectories (mean ± SE) of *TCF7* NGE and indicated gene signatures. Asymp, asymptomatic; Symp, symptomatic. ISG, interferon‐stimulated genes. *p*, by linear generalized estimating equations with ANOVA. (g) Case study 3: Influenza vaccine cohort (SDY67). *Left*, SAS‐1/MAS‐1 profiles at days 0, 3, and 28 among those with H‐L (SAS‐1^high^‐MAS‐1^low^) pre‐vaccination. *p*, by Fisher's exact test. *Middle*, IR groups (Grp) based on SAS‐1/MAS‐1 profiles at days 0, 3, and 28 since vaccination. *Right*, trajectories (mean ± SE) of the readouts in IR groups. *p*, by Welch's t‐test comparing groups 1 and 2 versus 3 and 4 at day 28.

Our evolutionary framework proposes that immune robustness represents a hallmark of multi‐protective salutogenic traits (Figure 1a). Our comprehensive analyses revealed distinct IR trajectories under diverse inflammatory stressors, delineating three phenotypes: IR‐preservers, IR‐reconstituters, and IR‐degraders (Figure 4a). These patterns represent distinct biological strategies—robustness, plasticity, and maladaptation—corresponding to superior, moderate, and compromised multi‐protection outcomes.

IR‐preservers demonstrate immune robustness through sustained maintenance of optimal IR‐*TCF7* ^high^ status and resistance to pathogenic triad activation during inflammatory stress (Figure 4a, blue). IR‐reconstituters exhibit plasticity, transiently compromising optimal IR‐*TCF7* ^high^ status while retaining the capacity to restore it during convalescence. This trajectory involves transient activation of the pathogenic triad followed by a moderate residual burden (Figure 4a, green). Critically, IR‐preservers and IR‐reconstituters remain indistinguishable during convalescence (constitutive state), as both achieve the optimal IR‐*TCF7* ^high^ status (Figure 4a). IR‐degraders exhibit maladaptation, failing to re‐establish optimal IR‐*TCF7* ^high^ status alongside persistently elevated pathogenic triad burden (Figure 4a, yellow).

**FIGURE 5:**
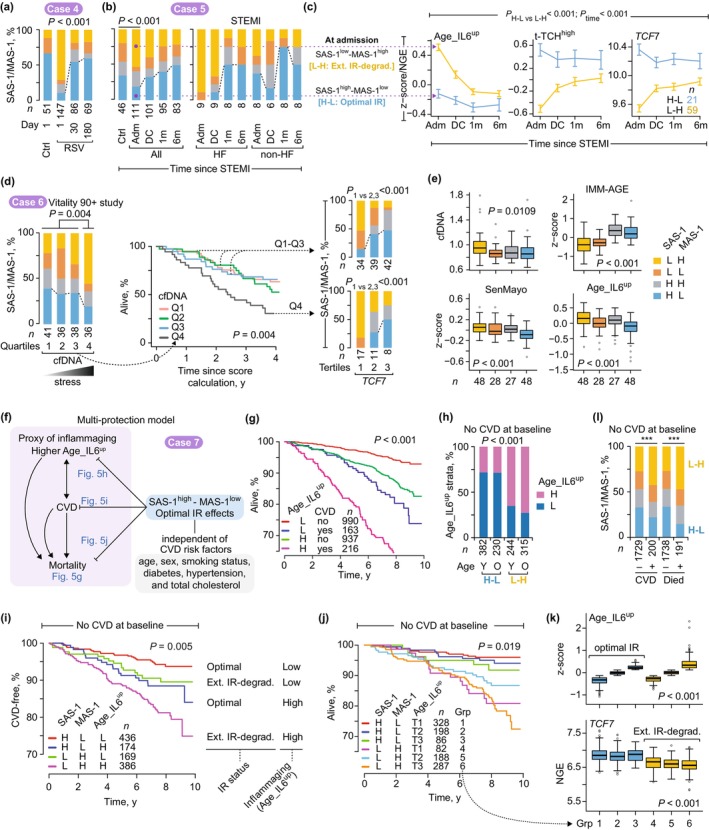
Transcriptomic metrics of immune resilience (IR): Associations in varied inflammatory contexts. (a) Case study 4 (GSE188427): Respiratory syncytial virus (RSV) infection cohort. Survival‐associated signature‐1 (SAS‐1)/Mortality‐associated signature‐1 (MAS‐1) profiles (key next to panel e) in controls (Ctrl) and RSV patients (days 1, 30, and 180). *p*, by *χ* ^2^ test. (b, c) Case study 5 (GSE59867): ST‐elevation myocardial infarction (STEMI) cohort. (b) SAS‐1/MAS‐1 profiles (H‐L: SAS‐1^high^‐MAS‐1^low^; L‐H: SAS‐1^low^‐MAS‐1^high^; median‐based strata: H, high; L, low) in STEMI patients at admission (Adm), discharge (DC), and post‐discharge (*left*) overall and (*right*) by subsequent heart failure (HF) status. *p*, by *χ* ^2^ test. Ext. IR‐degrad., extreme IR‐degraders. (c) Trajectories (mean ± SE) of the readouts (noted in Figure 1c) and normalized gene expression (NGE) of transcription factor 7 (*TCF7*). *p*, by linear generalized estimating equations. (d, e) Case study 6 (GSE65218): Vitality 90+ study. (d) *Left* and *middle*: Distribution of SAS‐1/MAS‐1 profiles and time to death by circulating cell‐free DNA (cfDNA) quartiles (increasing stress). *Right*, distribution of SAS‐1/MAS‐1 profiles by *TCF7* tertiles. *p*, by χ^2^, log‐rank, and Fisher's exact tests. (e) Median (IQR) levels of cfDNA and readouts by SAS‐1/MAS‐1 profiles. *p*, by Kruskal‐Wallis for cfDNA and ANOVA for z‐scores. (f–l) Case 7: Cardiovascular diseases (CVD) and mortality in the Framingham Heart Study. (f) Multi‐protection model. (g) Time to death by CVD and inflammaging proxy (Age_IL6^up^ signature; median‐based strata: H, high; L, low). (h) Age_IL6^up^ strata by age and SAS‐1/MAS‐1 profiles (H‐L: SAS‐1^high^‐MAS‐1^low^; L‐H: SAS‐1^low^‐MAS‐1^high^). *p*, by χ^2^ test. (i) Time to CVD diagnosis and (j) time to death among people without CVD diagnosis at baseline by SAS‐1/MAS‐1 profiles and Age_IL6^up^ strata. Grp, group. *p* in panels g, i and j, by Cox proportional hazards models adjusted for age, sex, smoking status, diabetes, hypertension, and total cholesterol with likelihood ratio test (LRT). (k) Median (IQR) levels of (*top*) Age_IL6^up^ signature (z‐scores) and (*bottom*) *TCF7* (NGE) by groups in panel j. *p*, by ANOVA for z‐score and Kruskal‐Wallis for *TCF7*. (l) Distribution of SAS‐1/MAS‐1 profiles by indicated outcomes (+, present; −, absent). *p*, by *χ* ^2^ test.

**FIGURE 6:**
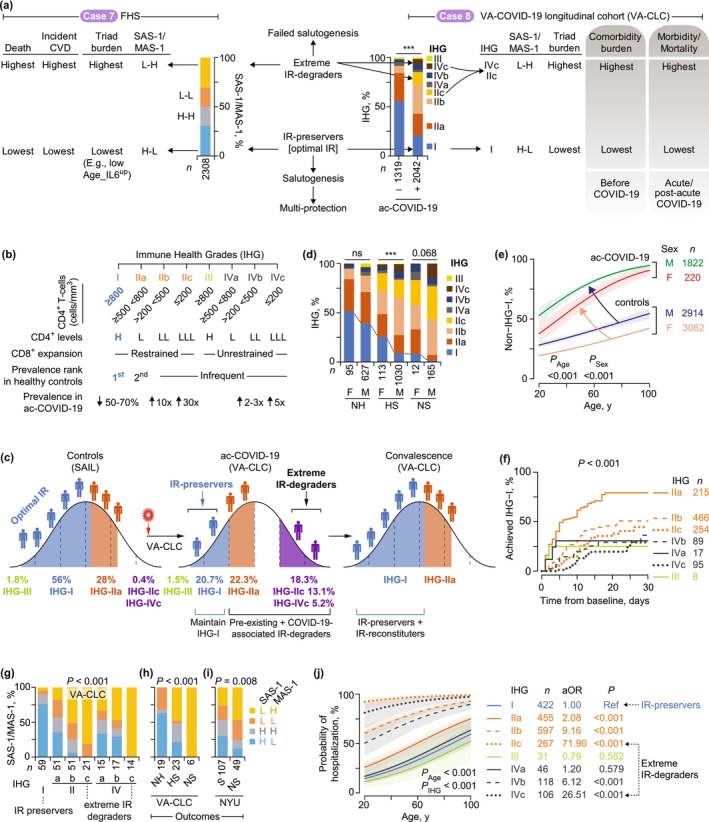
Immune resilience (IR) status and outcomes during acute COVID‐19 (ac‐COVID‐19). Panels b to h and j from the Veterans Affairs COVID‐19 Longitudinal Cohort (VA‐CLC; case study 8). (a) Parallels between associations of IR metrics in the (*left*) Framingham Heart Study (FHS; IR metrics: Survival‐associated signature‐1 (SAS‐1)/Mortality‐associated signature‐1 (MAS‐1) profiles) and (*right*) the VA‐CLC [IR metrics: Immune health grades (IHGs; see panel b) and SAS‐1/MAS‐1 profiles]. ***p < 0.001 (*χ* ^2^ test): IHGs in controls without (−) acute COVID‐19 [San Antonio Immunologic Resilience Longitudinal (SAIL) cohort] vs. with (+) acute COVID‐19 (at baseline). H‐L: SAS‐1^high^‐MAS‐1^low^; L‐H: SAS‐1^low^‐MAS‐1^high^ (median‐based strata: H, high; L, low). (b) Derivation of IHG subgrades based on the balance between levels of CD8^+^ and CD4^+^ T‐cell counts. (H, high; L, low). (c) IHG distributions in study groups. (d) Baseline IHG by outcomes and sex in VA‐CLC. NH, nonhospitalized; HS, hospitalized survivors; NS, nonsurvivors. Dashed line, %IHG‐I. ***p < 0.001 (Fisher's exact test); ns, non‐significant. (e) Percent of non‐IHG‐I grades (with 95% confidence bands) by sex in controls (SAIL, SardiNIA, and UCSD HIV‐seronegative cohorts combined) and acute COVID‐19 at baseline (VA‐CLC) across age. *p*, by logistic regression adjusting for age and sex. (f) Time to achieve IHG‐I within 30 days of presentation by baseline IHG. *p*, by age‐adjusted Cox proportional hazards model. (g–i) SAS‐1/MAS‐1 profiles by (g) baseline IHGs, (h) outcomes, and (i) survivors (S) versus NS [New York University (NYU) cohort]. *p*, by Fisher's exact test. (j) (*Left*) Line plots: Probability (with 95% confidence bands) by logistic regression of being hospitalized across age by baseline IHGs. *p*, by likelihood ratio test. (*Right*) Age‐adjusted odds ratio (aOR) and significance values from the logistic regression model.

**FIGURE 7:**
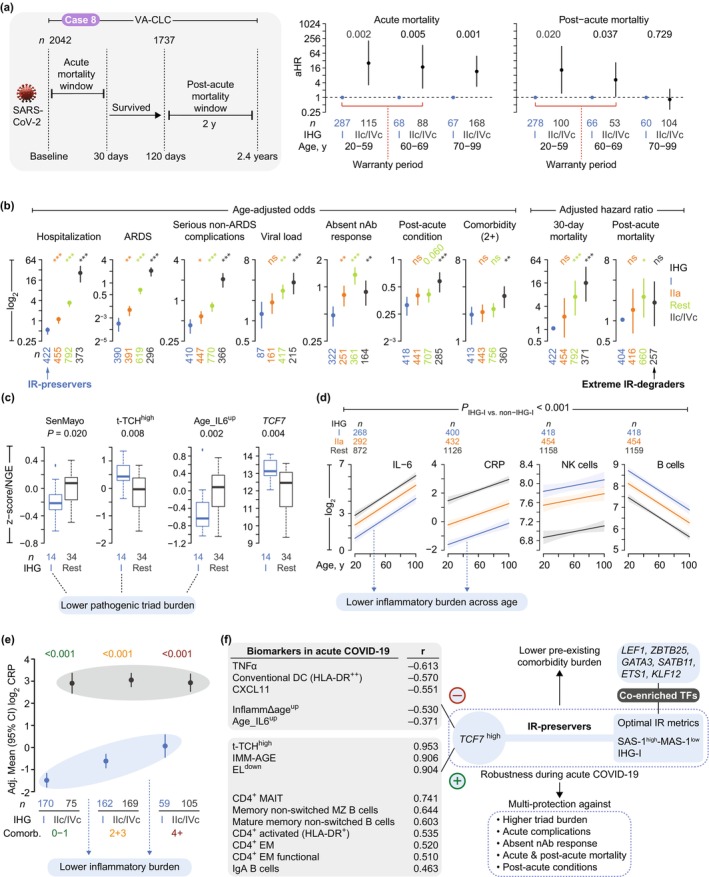
Immune resilience (IR) status and health profiles before, during, and following COVID‐19 in the Veterans Affairs COVID‐19 Longitudinal Cohort (VA‐CLC) (case study 8). (a) *Left*, acute and post‐acute mortality windows. *Right*, sex‐adjusted hazard ratios (aHR) with 95% confidence interval (CI) of acute and post‐acute mortality by baseline immune health grades (IHG) within indicated age strata. *p*, by Cox proportional hazards models. (b) Age‐adjusted odds (with 95% CI) and hazard ratios (with 95% CI) of outcomes by baseline IHG (Rest, grades other than IHGs I, IIa, IIc, and IVc). ARDS, acute respiratory distress syndrome; nAb, neutralizing antibody (absent, < 30% inhibition in surrogate virus neutralization test); 2+, ≥ 2 pre‐existing comorbidities. *p*, by logistic regression and Cox proportional hazards models adjusted for age. (c) Median (IQR) levels of the readouts (*z*‐scores) noted in Figure 1c and normalized gene expression (NGE) of *TCF7* (transcription factor 7). Rest, IHGs other than IHG‐I. *p*, by likelihood ratio test. (d) Linear regression modeling of log_2_ levels (with 95% confidence bands) of biomarkers across age by baseline IHG. IL‐6, interleukin 6; CRP, C‐reactive protein; NK, natural killer. *p*, by likelihood ratio test adjusted for age. (e) Age‐adjusted means (with 95% CI) of baseline log_2_‐transformed CRP levels stratified by preexisting comorbidity (Comorb.) burden and baseline IHG. *p*, by linear model adjusting for age. (f) *Left*, Pearson's correlation coefficient (r) of biomarkers, immune traits, and cytokines with *TCF7* (NGE) expression. *Right*, health profiles of IR‐preservers before, during, and following acute COVID‐19. *Upper right*, transcription factors co‐enriched with optimal IR status (SAS‐1^high^/MAS‐1^low^) in the Framingham Heart Study. cDC, conventional dendritic cells; EM, effector memory; HLA, human leukocyte antigen; IgA, immunoglobulin A; MAS‐1, mortality‐associated signature‐1; MZ, marginal zone; MAIT, mucosal‐associated invariant T‐cells; SAS‐1, survival‐associated signature‐1; TNFα, tumor necrosis factor α; *Higher levels of the IMM‐AGE signature were computed to signify an association with fewer senescent T‐cells (less immune aging and lower mortality; a {+}‐salutogenesis readout), as detailed in Section 4.2 of the Supporting Information. ns, non‐significant; *p < 0.05; **p < 0.01; ***p < 0.001.

**FIGURE 8:**
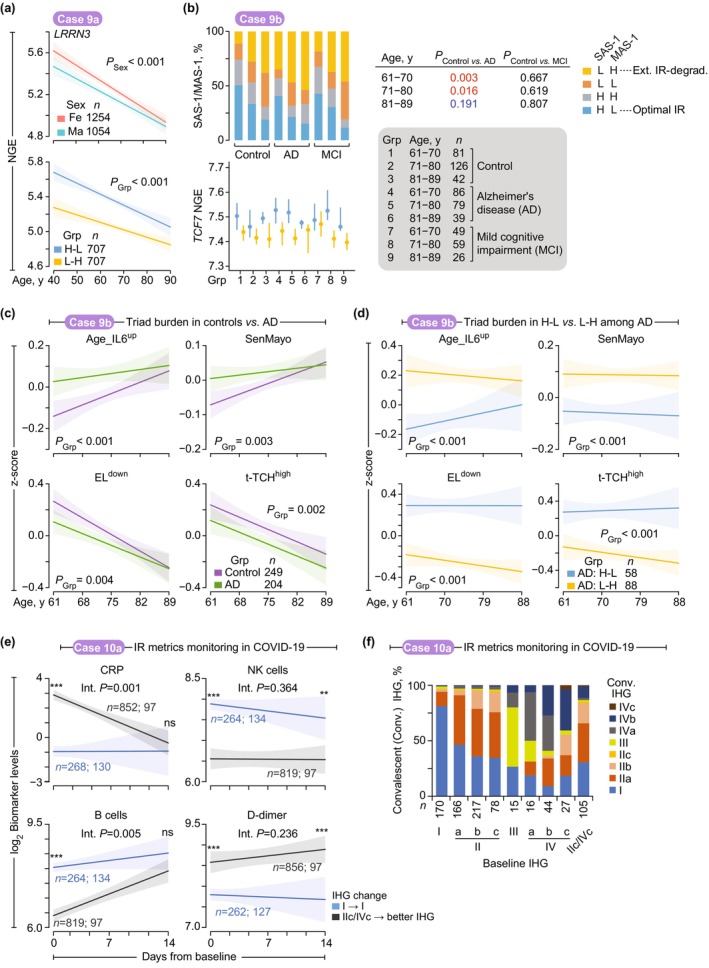
Association of immune resilience (IR) metrics with cortical gray matter thickness (CGMT), Alzheimer's disease (AD), and IR metrics monitoring in COVID‐19. (a) Case study 9a. Cohort: Framingham Heart Study. Linear regression modeling of levels (NGE, normalized gene expression with 95% confidence bands) of a transcriptomic correlate (*LRNN3*) of CGMT stratified by (*top*) sex and (*bottom*) IR status defined by Survival‐associated signature‐1 (SAS‐1) and Mortality‐associated signature‐1 (MAS‐1) profiles. H‐L: SAS‐1^high^‐MAS‐1^low^ (median‐based strata: H, high; L, low); L‐H: SAS‐1^low^‐MAS‐1^high^; Grp, group. (b–d) Case study 9b: AD and controls (GSE140829). (b) SAS‐1/MAS‐1 profiles and *TCF7* (transcription factor 7) normalized gene expression [NGE, median (IQR)] in controls and persons with AD or mild cognitive impairment. Ext. IR‐degrad., extreme IR‐degraders. *p*, by *χ* ^2^ and Fisher's exact test between study groups in age strata. (c‐d) Linear regression modeling of levels (with 95% confidence bands) of readouts (*z*‐scores) noted in Figure 1c in (c) controls and persons with AD, and (d) AD patients with H‐L (SAS‐1^high^‐MAS‐1^low^) and L‐H (SAS‐1^low^‐MAS‐1^high^) profiles. (e, f) Case study 10a: Veterans Affairs COVID‐19 Longitudinal Cohort (VA‐CLC). (e) Biomarker trajectories during 14 days from baseline in patients who remained IR‐preservers (IHG‐I) vs. extreme IR‐degraders (IHG‐IIc/IVc) who improved their IHG status. Two *n*‐values per group presented in panel e correspond to number of biomarker measurements and number of unique persons, respectively. *p*, by linear GEE with interaction term and generalized linear hypothesis tests at Day 0 and Day 14. (f) Distribution of IHG during convalescence (Conv.) by baseline IHG. ns, non‐significant; *, *p* < 0.05; **, *p* < 0.01; ***, *p* < 0.001.

**FIGURE 9:**
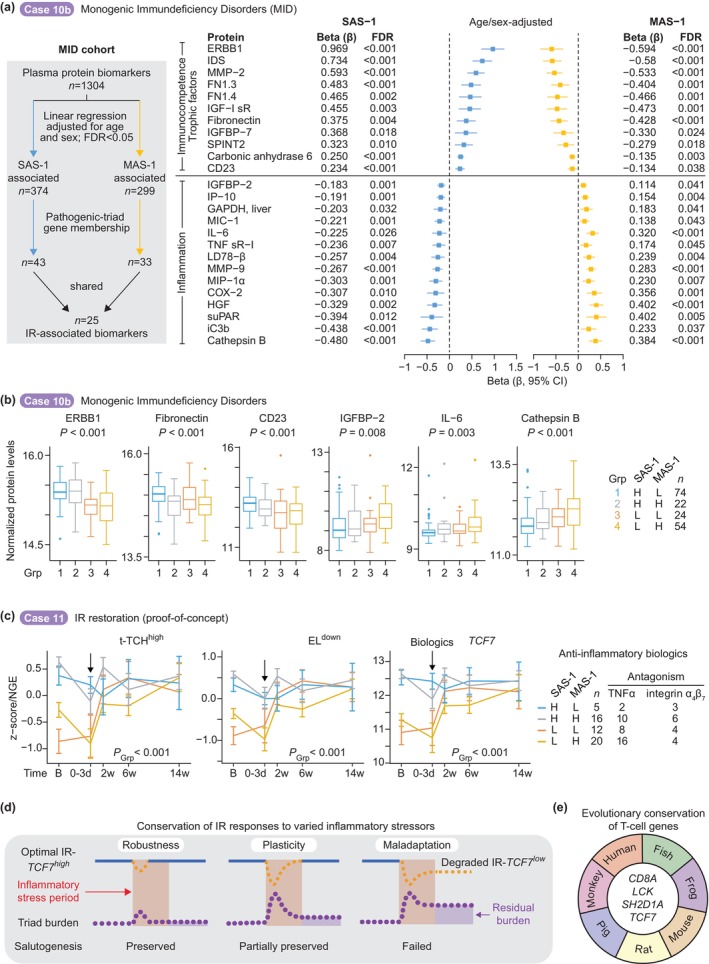
Association of immune resilience (IR) metrics with proteomic biomarkers and biologics. (a, b) Case study 10b: Monogenic immunodeficiencies (MID) multi‐omics cohort (phs002732.v1.p1). (a) *Left*, Summary schema of proteomic biomarkers analysis findings. *Right*, Linear slopes (β) of levels of indicated plasma proteomic biomarkers with survival‐associated signature‐1 (SAS‐1) and mortality‐associated signature‐1 (MAS‐1) gene expression signatures. Analysis performed using linear regression adjusting for age and sex. FDR by Benjamini‐Hochberg. (b) Median (IQR) levels of indicated proteomic biomarkers by SAS‐1/MAS‐1 profiles (median‐based strata: H, high; L, low). *p*, by ANOVA. (c) Case study 11 (GSE191328): Inflammatory bowel disease treated with anti‐inflammatory biologics. Trajectories (mean ± SE) of the levels of readouts (*z*‐scores) noted in Figure 1c and *TCF7* normalized gene expression (NGE) by SAS‐1/MAS‐1 profiles. B, baseline; d, day; w, week. *p*, by linear generalized estimating equations. (d) Model of robustness, plasticity, and maladaptation to inflammatory stress and residual burden of the pathogenic triad. (e) Genes whose expression in T‐cells is conserved across species.

Case studies 1–11 (Figures 4, 5, 6, 7, 8, 9) illustrate these trajectories across both extrinsic inflammatory challenges (e.g., infections) and intrinsic drivers [e.g., aging, cardiovascular disease (CVD)]. These findings confirm the universal nature of IR responses and demonstrate how this framework elucidates immune adaptation to such stimuli.

#### 2.11.1 Case Study 1: IR Responses During Natural Infections

This longitudinal study investigated IR dynamics in adults aged < 50 years before, during, and after symptomatic acute respiratory viral infections (e.g., influenza) (Figure 4b). We focused on 58 subjects with pre‐infection SAS‐1^high^‐MAS‐1^low^ profiles exhibiting optimal IR. IR status at three timepoints—pre‐infection (timepoint 0), symptom onset (timepoint 1), and spring follow‐up (timepoint 6)—defines three distinct IR response trajectories (Figure 4b). Most subjects displayed severe IR degradation at symptom onset, transitioning from SAS‐1^high^‐MAS‐1^low^ to SAS‐1^low^‐MAS‐1^high^. A subgroup (“IR‐preservers”) retained SAS‐1^high^‐MAS‐1^low^ profiles throughout infection and demonstrated exceptional resilience to infection‐induced immune perturbations (Figure 4c). By timepoint 6, two additional groups emerged: “IR‐reconstituters,” who successfully restored SAS‐1^high^‐MAS‐1^low^ status, and “IR‐degraders,” who failed to reconstitute this profile. Analyses at timepoint 6 demonstrated strong correlations between IR status and {+/−}‐salutogenesis readouts that tracked triad burdens (Figure 4b,c and Figure S8). *TCF7* expression—a key IR biomarker—sharply graded across groups: highest in IR‐preservers, intermediate in IR‐reconstituters, and lowest in IR‐degraders (Figure 4c), providing mechanistic insights into differential recovery outcomes.

These patterns generalized to other infections. In both acute and chronic infection cohorts—including tuberculosis—failure to establish SAS‐1^high^‐MAS‐1^low^ immune profiles during recovery or therapy predicted elevated triad burden, reduced *TCF7* expression, and poorer clinical outcomes, including treatment failure or heightened risk of active tuberculosis (Figures S5c and S9). These reproducible findings reveal a conserved IR response mechanism across diverse pathogens.

#### 2.11.2 Case Study 2: IR Responses During Human Infection Challenges

We next examined IR trajectories using human challenge infection studies. Among younger adults (*n* = 41) receiving intranasal inoculation with influenza virus (Woods et al. 2013), 50% remained asymptomatic post‐inoculation. A comparison between participants exhibiting pre‐inoculation SAS‐1^high^‐MAS‐1^low^ versus SAS‐1^low^‐MAS‐1^high^ profiles (Figure 4d–f) revealed two key advantages linked to baseline SAS‐1^high^‐MAS‐1^low^.

First, participants with pre‐inoculation SAS‐1^high^‐MAS‐1^low^ exhibited a lower risk of symptomatic infection, with 59% (10/17) remaining asymptomatic post‐inoculation compared to 25% (2/8) in SAS‐1^low^‐MAS‐1^high^ participants. In the SAS‐1^high^‐MAS‐1^low^ group, symptomatic cases exhibited higher conversion to SAS‐1^low^‐MAS‐1^high^ compared to asymptomatic cases (Figure 4d, middle vs. top). Conversion rates reached peak levels approximately 77 h post‐inoculation (Figure 4e, top). At this peak, symptomatic individuals displayed maximal levels of interferon‐stimulating genes (ISGs) and {−}‐salutogenesis readouts (e.g., Age_IL6^up^), whereas *TCF7* and {+}‐salutogenesis readouts reached minimal levels (Figure 4f and Figure S10a).

Second, pre‐inoculation SAS‐1^high^‐MAS‐1^low^ participants displayed lower triad burdens at study completion (t = 108 h) (Figure 4f, blue/green trajectories). Symptomatic SAS‐1^high^‐MAS‐1^low^ individuals exhibited transient *TCF7* declines and triad burden increases (e.g., elevated Age_IL6^up^ and reduced EL^down^), leading to restoration of baseline *TCF7* levels and triad burden by 108 h despite sustained ISG elevation (Figure 4f, green trajectories and Figure S10a). In contrast, SAS‐1^low^‐MAS‐1^high^ individuals consistently maintained lower *TCF7* levels and higher triad burdens throughout (yellow trajectories; Figure 4f).

A parallel human infection challenge model with *Salmonella typhi* revealed that maintained SAS‐1^high^‐MAS‐1^low^ status was linked to asymptomatic status and notably elevated *TCF7* levels (Figure S10b). Collectively, these findings demonstrate that maintaining a SAS‐1^high^‐MAS‐1^low^ status (optimal IR) exerts protective effects by reducing symptomatic infection risks and keeping triad burdens low.

#### 2.11.3 Case Study 3: IR Responses During Vaccinations

Inflammatory stress induced by influenza (Figures S11–S20) and SARS‐CoV‐2 (Figure S21) vaccinations provided an experimental system to analyze IR degradation patterns. In influenza vaccination cohorts with adequate sample sizes of individuals with optimal IR status (SAS‐1^high^‐MAS‐1^low^) prior to vaccination (Figures S16–S20), we identified three distinct post‐vaccination IR response categories: IR‐preservers, IR‐reconstituters, and IR‐degraders (as illustrated in Figure 4g and Figure S16). In a representative influenza vaccine cohort, 39% of individuals with pre‐vaccination SAS‐1^high^‐MAS‐1^low^ status shifted to IR‐degrader status (non‐SAS‐1^high^‐MAS‐1^low^) post‐vaccination. This shift was associated with increased pathogenic triad burden and reduced *TCF7* levels compared to IR‐preservers (groups 3 and 4 in Figure 4g and Figure S16). IR‐reconstituters exhibited transient increases in pathogenic triad burden following vaccination (group 2 in Figure 4g and Figure S16).

Individuals with SAS‐1^high^‐MAS‐1^low^ status *prior to* influenza or SARS‐CoV‐2 vaccination maintained lower pathogenic triad burdens, higher *TCF7* levels, and elevated expression of gene signature modules linked to lymphocyte subsets (e.g., plasma cells) associated with superior vaccine responsiveness [Figures S11–S21; (Arunachalam et al. 2021; Li et al. 2014)]. These findings indicate that pre‐vaccination IR status and post‐vaccination IR response patterns may serve as critical determinants of immune responses to vaccination.

#### 2.11.4 Case Studies 4–5: Parallels Between Infectious and Noninfectious Inflammatory Stressors

We analyzed SAS‐1/MAS‐1 patterns across two inflammatory models: pediatric acute respiratory syncytial virus (RSV) infection (case study 4; Figure 5a and Figure S22) versus adult ST‐segment elevation myocardial infarction (STEMI) (case study 5; Figure 5b). At baseline, both RSV and STEMI patients demonstrated lower frequencies of the SAS‐1^high^‐MAS‐1^low^ profile and higher frequencies of SAS‐1^low^‐MAS‐1^high^ compared to controls (non‐infected children and those with stable coronary artery disease, respectively). This imbalance was most evident in STEMI patients who subsequently developed heart failure (Figure 5b, right). By 180 days post‐RSV, convalescent children exhibited profile distributions nearly indistinguishable from controls, with 40% displaying non‐SAS‐1^high^‐MAS‐1^low^ profiles (Figure 5a and Figure S22). Post‐STEMI patients demonstrated partial restoration of SAS‐1^high^‐MAS‐1^low^ profile levels, but those with initial SAS‐1^low^‐MAS‐1^high^ profiles maintained elevated triad burden and reduced *TCF7* levels (Figure 5c and Figure S23). These parallel SAS‐1/MAS‐1 trajectory patterns in response to both infectious (RSV) and noninfectious (STEMI) inflammatory stressors indicate a conserved IR response pattern across diverse inflammatory stimuli.

#### 2.11.5 Case Study 6: IR‐Preserver Analogs in Nonagenarians

Elevated circulating cell‐free DNA (cfDNA) levels in older adults are associated with mortality, inflammation biomarkers (including C‐reactive protein [CRP]), cognitive decline, frailty, and CVD (Jylhava et al. 2012; Nidadavolu et al. 2022; Polina et al. 2020). We utilized cfDNA levels as a proxy for intrinsic inflammatory stress to identify phenotypic counterparts of IR‐preservers, reconstituters, and degraders in the Vitality 90+ study (Jylhava et al. 2012). Elevated cfDNA levels (fourth quartile) were associated with a higher prevalence of SAS‐1^low^‐MAS‐1^high^ and a lower prevalence of SAS‐1^high^‐MAS‐1^low^ profiles (Figure 5d, left) and heightened mortality risk (Figure 5d, survival plots). The *TCF7* ^high^ dose–response relationship (increased SAS‐1^high^‐containing profiles), observed in the Framingham Heart Study (Figure 3e,f), extended to nonagenarians (Figure 5d, right stacked barplots). Nonagenarians with SAS‐1^high^‐MAS‐1^low^ status showed three key advantages compared to SAS‐1^low^‐MAS‐1^high^ individuals (Figure 5d,e and Figure S24): higher *TCF7* levels, reduced cfDNA levels, and reduced pathogenic triad burden.

These findings suggest that the IR‐preserver phenotype—characterized by SAS‐1^high^‐MAS‐1^low^ status and elevated *TCF7* levels—correlates with a biomarker (cfDNA) of survival while attenuating pathogenic triad burden even in nonagenarians. The consistency of these patterns across age cohorts—from middle‐aged participants in the Framingham Heart Study to nonagenarians in the Vitality 90+ study—strengthens the biological relevance of the IR‐preserver concept in promoting healthy aging and longevity.

#### 2.11.6 Case Study 7: IR Status Links to Incident CVD in the FHS

Figure 5f (left) illustrates the traditional mortality risk model, where inflammaging predicts CVD, and both predict mortality (Abdellatif et al. 2023; Barcena et al. 2022; Fredman and MacNamara 2021). In the Framingham Heart Study, inflammaging (indexed by elevated Age_IL6^up^) and CVD independently predicted increased mortality hazards (Figure 5g and Table S4a). Higher levels of Age_IL6^up^ also predicted incident CVD (Figure S25 and Table S4b). Among individuals without CVD at baseline, SAS‐1^high^‐MAS‐1^low^ (indicating optimal IR status) showed protective effects controlling for CVD risk factors (Figure 5f), such as reduced inflammaging (Figure 5h), lower incident CVD (Figure 5i), reduced mortality risks (Figure 5j), and elevated *TCF7* expression (Figure 5k)—even with elevated Age_IL6^up^. Notably, results displayed in Figure 5g,i,j are adjusted for age, sex, smoking status, diabetes, hypertension, and total cholesterol.

Two‐thirds of individuals with baseline SAS‐1^high^‐MAS‐1^low^ (analogs of “IR‐preservers”) displayed lower inflammaging (Figure 5h). This IR‐preserver‐like group demonstrated reduced CVD incidence (converting from CVD‐free status to CVD; Figure 5i). In contrast, two‐thirds of those with extreme IR degradation (SAS‐1^low^‐MAS‐1^high^) had higher inflammaging (Figure 5h) and the highest age‐adjusted incident CVD risk (Figure 5i and Table S4b). SAS‐1^high^‐MAS‐1^low^ status associated with lower mortality HRs across all Age_IL6^up^ strata (Figure 5j and Table S4c), including those with elevated Age_IL6^up^ (groups 3 vs. 6 in Figure 5j and Table S4c). *TCF7* levels were higher in optimal versus degraded IR groups regardless of Age_IL6^up^ levels (Figure 5k).

The SAS‐1^low^‐MAS‐1^high^ profile was overrepresented among those who experienced CVD events or mortality, while the SAS‐1^high^‐MAS‐1^low^ profile was underrepresented (Figure 5l). The consistent relationship between optimal IR metrics and favorable cardiovascular outcomes—even in the context of inflammaging—points to a novel protective mechanism that transcends the conventional inflammation‐CVD paradigm (Figure 5f), with implications for cardiovascular aging.

#### 2.11.7 Case Study 8: IR‐Preserver Status and Multi‐Layered Protection During COVID‐19

Figure 6a summarizes the effects of optimal IR‐*TCF7* ^high^ status across two distinct biological contexts: a *non‐infectious* aging framework (Framingham Heart Study; left panel, case 7) and an *infectious disease* scenario (COVID‐19; right panel, case 8). This comparative analysis highlights the versatility of optimal IR‐*TCF7* ^high^ (IR‐preservers) in mitigating diverse health challenges, emphasizing its potential as a universal biomarker for systemic resilience.

##### 2.11.7.1 Case Study 8a: IR Monitoring During COVID‐19

To investigate optimal IR's role in COVID‐19 resistance, we employed two IR‐monitoring metrics: Immune Health Grades (IHGs) for CD8‐CD4 balance assessment (Figure 6b and Note S1) and SAS‐1/MAS‐1 profiles (Ahuja et al. 2023; Lee et al. 2021). We monitored 2042 participants from the Veterans Affairs COVID‐19 Longitudinal Cohort (VA‐CLC), with longitudinal follow‐up spanning up to 2.5 years (baseline characteristics: Table S5 and Figure S26a) (Ahuja et al. 2023; Lee et al. 2021). Initiated in March 2020, the VA‐CLC predominantly consisted of pre‐vaccine‐era veterans and SARS‐CoV‐2‐naive individuals. This design enabled two key objectives through: (i) identifying individuals vulnerable to optimal IR‐*TCF7* ^high^ degradation caused by COVID‐19‐associated inflammatory stress, and (ii) evaluating baseline IR status in relation to health outcomes across three distinct phases—pre‐existing comorbidities, acute COVID‐19, and post‐acute phases (Figure 6a, right).

We defined progressively worsening immune grades by weighting absolute CD8^+^ and CD4^+^ T‐cell counts against IHG‐I as our benchmark for optimal IR (Figure 6b,c [left], and Note S1) (Ahuja et al. 2023). IHG‐I, characterized by high CD4^+^ levels and restrained CD8^+^ expansion (Figure 6b), predominated in four non‐COVID‐19 control cohorts (Figure 6a–c; Figure S26b and Table S6). While IHG‐IIa was the second most prevalent grade, the remaining grades (IHG‐IIb/c, IHG‐III, and IHG‐IV) occurred rarely in controls (Figure 6a [right], Figure 6c [left], and Figure S26b and S27a) (Ahuja et al. 2023; Lee et al. 2021).

##### 2.11.7.2 Case Study 8b: IR‐Preservers, −Reconstituters, and ‐Degraders During Acute COVID‐19

IHG‐I prevalence was significantly higher in control cohorts (55%–70%) compared to the VA‐CLC cohort (20.7%) at baseline (Figure 6c and Figures S26b and S27a). Acute COVID‐19 is marked by reduced IHG‐I prevalence and a 2‐ to 30‐fold increase in non‐IHG‐I grades (e.g., IHG‐IIb/c and IHG‐IVb/c), which are rarely observed in control populations (Figure 6c and Figures S26b and S27a). These non‐IHG‐I grades showed higher prevalence among nonsurvivors (30‐day all‐cause mortality) and hospitalized survivors compared to nonhospitalized patients (Figure 6d), establishing a direct association with disease severity and clinical outcomes.

Acute COVID‐19 accelerates IR degradation rates across all age groups (Figure 6e). Control populations exhibited a near‐linear increase in non‐IHG‐I grades with age. This age‐related pattern became more pronounced during acute infection, resulting in a nonlinear trajectory (Figure 6e and Figure S27a). Males demonstrated higher prevalence rates of non‐IHG‐I grades compared to females in both control cohorts and the VA‐CLC cohort (Figure 6d,e and Figure S27a).

During convalescence, IHG distributions gradually shifted back toward patterns observed in age‐ and sex‐matched controls (Figure 6c; see Figure S27a for complete recovery dynamics), defining three distinct trajectories: IR‐preservers, IR‐reconstituters, and IR‐degraders. Individuals who maintained baseline IHG‐I (optimal IR) emerged as IR‐preservers, while those starting with non‐IHG‐I grades but achieving IHG‐I during recovery represented IR‐reconstituters (Figure 6c,f). Notably, IHG‐I reconstitution rates followed a clear hierarchy based on baseline IHGs, with the sequence IHG‐IIa > IHG‐IIb > IHG‐IIc > IHG‐IV (Figure 6f and Figures S27b,c and S28).

We classified patients presenting with IHG‐IIc or IHG‐IVc as extreme IR‐degraders based on three compelling lines of evidence. First, these grades occurred exceptionally rarely in controls (0.4%) but reached a striking 18.3% prevalence during acute COVID‐19, with particularly high frequency in nonsurvivors (Figure 6c,d). Second, they exhibited a severely impaired capacity to reconstitute IHG‐I, revealed through longitudinal analyses (Figure 6f and Figures S27b,c and S28). Third, these patients with IHG‐IIc or IHG‐IVc at presentation demonstrated strong links to the SAS‐1^low^‐MAS‐1^high^ profile, a marker of extreme IR degradation (Figure 6g).

The baseline SAS‐1^low^‐MAS‐1^high^ profile emerges as a hallmark of nonsurvivors (detected in all cases) but is not observed among non‐hospitalized patients (Figure 6h). Our multi‐cohort validation confirms this pattern: SAS‐1^low^‐MAS‐1^high^ is significantly elevated and SAS‐1^high^‐MAS‐1^low^ markedly underrepresented in nonsurvivors versus ICU survivors from New York University Medical Center (Figure 6i, Table S7). In contrast, the SAS‐1^high^‐MAS‐1^low^ profile predominates in individuals with IHG‐I and nonhospitalized cohorts but is absent in patients with IHG‐IIc/IHG‐IVc or fatal outcomes (Figure 6g,h and Figure S29). This polarized pattern positions IHG‐I and SAS‐1^high^‐MAS‐1^low^ as hallmarks of optimal IR, while IHG‐IIc/IHG‐IVc and SAS‐1^low^‐MAS‐1^high^ signal extreme IR degradation (Figure 6a [right], Figure 6g [bottom], Figure 6j [right]).

##### 2.11.7.3 Case Study 8c: IR‐Preservers Restrict Adverse Acute‐ and Post‐COVID‐19 Outcomes

IR‐preservers show significantly lower hospitalization rates across all ages, with 80‐year‐old IR‐preservers demonstrating reduced rates compared to extreme IR‐degraders of any age (Figure 6j; Figure S30a). This protective effect extends to mortality rates (Figure 7a; Figure S30b–e). Extreme IR‐degraders consistently show higher hazard ratios (HRs) for acute mortality across all ages and higher HRs for post‐acute mortality during the warranty period (< 70 years) compared to IR‐preservers (Figure 7a; Figure S30b). Notably, extreme IR‐degrader status exerts the strongest effect on acute mortality in adults under 60 years (Figure S30d).

IHG‐I presentation confers protection against multiple adverse outcomes (Figure 7b), including acute mortality (9% of cases; *n* = 174) (Figure S30b–d), hospitalization (65%; *n* = 1318), acute respiratory distress syndrome (31%; *n* = 528), serious non‐acute respiratory complications (44%; *n* = 884) (Figure S30f), higher viral loads (74%; *n* = 433; inferred via cycle threshold values; Figure S31a–d), post‐acute conditions (29%; *n* = 531) (Figure S31e,f), and post‐acute mortality (7%; *n* = 114). These associations remained robust after adjusting for confounders such as age, sex, seven blood biomarkers measured as part of standard‐of‐care protocols (e.g., IL‐6, CRP; Table S8), viral load, pandemic eras, vaccine status, and cytomegalovirus serostatus (Table S9). Importantly, both baseline IHG status and improved IR status within the first 5‐days of presentation were associated with improved acute and post‐acute survival, even after adjusting for age and biomarkers (Figure S32 and Table S10).

IR‐preservers demonstrate functional immune responses through the presence of neutralizing antibodies (nAbs) to SARS‐CoV‐2. The absence of nAbs (45%; *n* = 489) suggests impaired immunity and correlates with higher mortality risk after adjusting for confounding variables (Figure S33a). Non‐IHG‐I individuals had higher age‐adjusted odds of failing to produce nAbs (Figure 7b and Figure S33b,c). A COVID‐19 vaccine cohort corroborated these findings, demonstrating stronger nAb persistence after primary vaccination in IHG‐I individuals after adjusting for age, sex, prior infection, and vaccination timing (Figure S33d,e and Table S11). Additionally, IHG‐I correlated with gene expression modules associated with vaccine responsiveness, such as plasma cell‐related modules (Arunachalam et al. 2021; Li et al. 2014) (Figure S34). Together, these findings highlight IR status's significant impact on both acute and post‐acute COVID‐19 outcomes, immune function, and vaccine responsiveness.

Four key findings highlight mechanisms underlying the multifaceted protection observed in IR‐preservers (IHG‐I). First, extreme IR‐degraders showed significantly higher odds of two or more pre‐existing comorbidities compared to IR‐preservers after adjusting for age (Figure 7b). Additionally, IR‐preservers consistently maintained lower comorbidity burdens across ages compared to extreme IR‐degraders (Figure S35 and Table S5a). Second, IR‐preservers (IHG‐I, SAS‐1^high^‐MAS‐1^low^) displayed lower triad burdens, while extreme IR‐degraders (IHG‐IIc/IHG‐IVc, SAS‐1^low^‐MAS‐1^high^) exhibited high triad burdens (Figure 7c; Figure S36). Third, the reduced triad burdens in IR‐preservers stemmed from their tightly controlled biomarker responses during acute COVID‐19 (Figure 7d,e; Figures S37 and S38). Specifically, standard‐of‐care biomarkers displayed age‐related trends, with some markers increasing (e.g., IL‐6, hs‐CRP, NK cells) and others decreasing (e.g., B‐cells). However, IR‐preservers consistently showed superior biomarker profiles—characterized by lower inflammation and higher immunocompetence—across all ages compared to extreme IR‐degraders (Figure 7d and Figure S37).

Fourth, IR‐preservers demonstrated clinically significant advantages even in high‐risk subgroups (≥ 4 comorbidities [Figure S39] or obesity [Figure S40]). These patients showed lower age‐adjusted inflammatory biomarker levels [including high sensitivity CRP (hs‐CRP)] compared to extreme IR‐degraders (Figure 7e). While hs‐CRP levels in IHG‐I patients tracked with comorbidity burden, extreme IR degradation subgroups (IHG‐IIc/IHG‐IVc) displayed persistently elevated hs‐CRP that remained uniform across all comorbidity levels (Figure 7e). These findings demonstrate that IR status independently modulates inflammatory profiles, with effects persisting after adjustment for both age (Figure 7d) and comorbidity burden (Figure 7e). This conclusion was reinforced by the observation that IHG‐I correlated with reduced acute and post‐acute mortality hazards after adjusting for age, comorbidities, and biomarkers (Figure S41 and Table S12). The consistency of these associations across subgroups and analytical approaches underscores the robustness of protective mechanisms in IR‐preservers, highlighting their critical relevance for understanding and managing COVID‐19 outcomes in diverse populations.

##### 2.11.7.4 Case Study 8d: TCF7^high^ Hallmarks IR‐Preservers and Tracks Immunoprotective Traits

The preservation of optimal IR with concurrent high *TCF7* expression defines IR‐preservers in acute COVID‐19, revealing a novel mechanism for multifaceted protection. *TCF7* levels were significantly elevated in IR‐preservers (IHG‐I, SAS‐1^high^‐MAS‐1^low^), non‐hospitalized individuals, and survivors (Figure 7c; Figure S36). Strikingly, *TCF7* expression correlated positively with {+}‐salutogenesis markers (e.g., IMM‐AGE) and inversely with {−}‐salutogenesis markers (e.g., Age_IL6^up^) (Figure 7f, left). To investigate *TCF7*'s role, we analyzed 65 immune traits and 21 cytokines (age‐adjusted, multiple comparison‐corrected), identifying 10 traits/cytokines fulfilling three criteria: (1) *TCF7* association, (2) linkage to IHG‐I (optimal IR), and (3) ties to favorable outcomes (non‐hospitalization/survival) (Figure 7f; Figures S42–S43; Table S13). Seven immune traits—notably CD4^+^ mucosal‐associated invariant T (MAIT) cells—were elevated, while HLA‐DR^++^ conventional dendritic cells and two cytokines (TNFα and ITAC [CXCL11]) were reduced (Figure 7f, left; Figures S42 and S43). These data illuminate the dynamic interplay between *TCF7*, IR preservation, and immune signatures in COVID‐19 outcomes (Figure 7f).

To investigate mechanisms sustaining optimal IR, we identified transcription factors linked to this longevity‐associated status. Using an unbiased approach, we screened 1380 transcription factors (Lambert et al. 2018) and identified seven showing elevated expression in SAS‐1^high^‐MAS‐1^low^ individuals from the Framingham Heart Study: *TCF7, LEF1, ZBTB25, GATA3, SATB11, ETS1*, and *KLF12* (Figure 7f, right; Figure S44a, Supporting Information). These transcription factors exhibited higher expression in females (Figure S44b), with three—*SATB11, ETS1*, and *KLF12*—showing significant associations with increased lifespan (Figure S44c). During acute COVID‐19, their expression levels were elevated in individuals preserving IR compared to extreme degraders and in survivors compared to nonsurvivors (Figure S44d). These findings establish a connection between a core set of transcription factors, optimal IR, and enhanced health outcomes, implicating their role in immune balance and longevity.

#### 2.11.8 Case Study 9: IR Status in Brain Aging and Alzheimer's Disease (AD)

T‐cells are central players in brain aging, neurodegeneration, and AD (Chen et al. 2023; Dias‐Carvalho et al. 2024; Nachun et al. 2019). We found that SAS‐1^high^‐MAS‐1^low^ profiles, compared to SAS‐1^low^‐MAS‐1^high^ profiles, are linked to two key protective features: elevated *LRRN3* expression—a factor positively correlated with cortical gray matter thickness (CGMT), a marker of brain health and slower aging (Case Study 9a; Figure 8a)—and lower AD burden (Case Study 9b; Figure 8b–d). Our results imply that the SAS‐1^high^‐MAS‐1^low^ profile may protect against neurodegeneration, providing mechanistic insights into brain aging and AD pathogenesis.

##### 2.11.8.1 Case Study 9a: IR Status and Transcriptomic Marker of Brain Aging (CGMT)

Kochunov et al. (Kochunov et al. 2013) identified transcriptomic correlates of cortical gray matter thickness (CGMT) and demonstrated that normal cerebral aging results from progressive declines in regenerative capacity and increased neuroinflammation. They identified eight genes whose expression levels were associated with CGMT at the transcriptome‐wide level. Among these, *LRRN3* expression showed the strongest positive correlation with CGMT, mirroring the expression patterns of *CCR7* and *TCF7* (Cano‐Gamez et al. 2020), both components of SAS‐1 (Figure 1f). *LRRN3* expression correlated with indicators of superior immune status, including lower levels of activated CD8^+^ T‐cells and higher levels of CD8^+^ T‐cells expressing CD28, a critical costimulatory receptor necessary for optimal T‐cell activation and preventing T‐cell senescence acquisition (Chou et al. 2013). Given these associations, we examined the relationship between *LRRN3* and IR status. *LRRN3* expression levels were higher in females and decreased with age (Figure 8a, top). Notably, FHS participants with SAS‐1^high^‐MAS‐1^low^ showed higher *LRRN3* expression across all age groups compared to SAS‐1^low^‐MAS‐1^high^ individuals (Figure 8a, bottom).

##### 2.11.8.2 Case Study 9b: IR Status Associates With AD

Building on previous work by Nachun et al. (Nachun et al. 2019), which linked innate immunity to AD through peripheral blood transcriptomic analysis, our analysis of this dataset revealed that SAS‐1^low^‐MAS‐1^high^ profiles were significantly more prevalent in AD patients than in controls, while SAS‐1^high^‐MAS‐1^low^ profiles showed the opposite trend (Figure 8b). Notably, this pattern was absent in comparisons between mild cognitive impairment patients and controls, with no statistically significant differences observed (Figure 8b).

Pathogenic triad burdens exhibited age‐dependent increases, with AD patients carrying higher burdens than controls until advanced age (Figure 8c, Figure S45a). Mirroring observations from the Framingham Heart Study (Figure 3a), AD patients with SAS‐1^low^‐MAS‐1^high^ profiles displayed elevated pathogenic triad burdens compared to those with SAS‐1^high^‐MAS‐1^low^ profiles (Figure 8d, Figure S45b). These results suggest that interactions between the pathogenic triad and SAS‐1/MAS‐1 imbalance may drive AD progression.

#### 2.11.9 Case Studies 10–11: Immune Monitoring and IR‐Reconstitution

##### 2.11.9.1 *Case Study 10a: Immune Monitoring Paradigms Using IR Metrics*—*COVID‐19*

Based on our findings cases 1 to 10, we evaluated IR metrics monitoring as a clinical management tool, using COVID‐19 infection as a case study implemented at the VA‐CLC during the pandemic's early stages (Figure 8e,f). Comparative biomarker analysis between survivor groups—IR‐preservers (IHG‐I) maintaining stable status versus extreme IR‐degraders (IHG‐IIc/IVc) who showed IR improvement during acute infection (Figure 8e and Figure S46)—revealed three key findings: (1) hs‐CRP levels decreased significantly in extreme IR‐degraders who improved their IR status post‐baseline, aligning with stable IHG‐I cases; (2) B‐cell counts showed upward trends, while NK cell depletion remained persistent; and (3) D‐dimer levels remained notably elevated in extreme IR‐degraders compared to IR‐preservers, even after IR improvement. Persistent D‐dimer elevations may contribute to the observed increase in prothrombotic events in COVID‐19 (Cryer et al. 2022). Comparative analysis of baseline and convalescent IHG status showed that only 30% of patients with severe IR decline (IHG‐IIc/IVc) achieved IHG‐I status, indicating prolonged immune suppression (Figure 8f). Thus, prospective IR monitoring during COVID‐19 reveals varying capacities to restore optimal IR (IHG‐I) and identifies residual immunological damage that may have either predated the infection or been induced by it.

##### 2.11.9.2 *Case Study 10b: Immune Monitoring Paradigms Using IR Metrics*—**Biomarkers**

We utilized the monogenic immunodeficiencies (MID) multiomics cohort assembled by Sparks et al. (Sparks et al. 2024) to identify plasma protein biomarkers correlating with transcriptomic (SAS‐1/MAS‐1) metrics of IR (Figure 9a and Figure S47). Sparks et al. quantified 1304 proteins using SomaLogic panels. After adjusting for age and sex, we identified 374 SAS‐1‐associated and 299 MAS‐1‐associated proteins (FDR < 0.05; Table S14). Among these, 76 biomarkers (43 SAS‐1 associated and 33 MAS‐1 associated) had corresponding gene symbols represented in the pathogenic triad readout gene expression signatures (Table S14). Of these, 25 proteins were shared and showed significant associations with both SAS‐1 and MAS‐1 (FDR < 0.05; Figure 9a and Table S14), defining a core set of IR‐associated biomarkers. Selected biomarkers are depicted in the MID cohort stratified by SAS‐1/MAS‐1 profiles (Figure 9b and Figure S47). Pro‐inflammatory biomarkers such as IL‐6 progressively increased from optimal IR (SAS‐1^high^‐MAS‐1^low^) to extreme IR degradation (SAS‐1^low^‐MAS‐1^high^), whereas immunocompetence‐associated and trophic markers such as CD23—a B‐cell marker—exhibited the opposite pattern.

##### 2.11.9.3 **Case Study 11**: *Reconstitution of Optimal IR‐* **TCF7** ^*high*^ *in Persons With Extreme IR Degradation*

Our proof‐of‐principle studies explored whether IR degradation—driven by inflammatory mechanisms—could be reversed. Anti‐inflammatory agents, including TNFα and anti‐α_4_β_7_ integrin antagonists, effectively restored optimal IR‐*TCF7* ^high^ expression in inflammatory bowel disease patients who exhibited a MAS‐1^high^ profile at baseline (Figure 9c and Figure S48). Notably, preemptive TNFα antagonist administration significantly mitigated IR degradation while reducing pathogenic triad burden following experimental lipopolysaccharide challenge in younger adults (Figure S49). The findings from these exploratory studies demonstrate the potential of immunomodulatory agents in addressing IR dysfunction.

## 3 Discussion

### 3.1 Evolutionary Framework of Inflammatory Stress and Immune Resilience

Inflammatory stresses have shaped human evolutionary history by acting as selective pressures that drove the development of salutogenic traits—multi‐layered protective mechanisms enhancing immune resilience and facilitating recovery from inflammatory stress. Our analysis reveals a unifying principle: inflammatory stress responses exist along a continuum of immune robustness (IR‐preservers), plasticity (IR‐reconstituters), and maladaptation (IR‐degraders) (Figure 4a). This framework explains protection states from preserved to failed salutogenesis, consistent across age, inflammatory stressors, diverse populations, and health contexts—including infections, vaccinations, and myocardial ischemia (Figure 9d). Among individuals of comparable ages, these distinctive IR phenotypes align with minimal, moderate, and severe proinflammatory‐immunodeficiency states as well as depletion of trophic factors. Within our evolutionary‐salutogenesis model (Figures 1a,b and Figure 9d), these distinct IR phenotypes represent evolved adaptive strategies that directly influence healthspan, lifespan, and provide crucial metrics for clinical monitoring throughout human life.

We examined protective effects by analyzing connections between IR metrics and a spectrum of health outcomes, including infection risks and severity, immune biomarkers (including vaccine responsiveness), comorbidities (cardiovascular, metabolic, and neurological diseases), and mortality (Figure 1b). The IR‐preserver phenotype consistently demonstrated optimal integration of innate and adaptive immunity across datasets, ages, and sexes. This resilience—characterized by sustained optimal IR levels and elevated *TCF7* expression during inflammatory stressors—points to an evolutionarily conserved mechanism that confers substantial survival advantage.

### 3.2 Evolutionary Origins and Protective Mechanisms of TCF7‐Associated Immunity

Evidence supports the ancient origins and protective functions of the IR‐*TCF7* ^high^ phenotype. First, the canonical Wnt pathway—mediated by TCF/LEF proteins—plays a conserved role in animal biology that predates human evolution (J. Liu et al. 2022). This pathway's fundamental involvement in development and tissue homeostasis underscores its importance in basic biological processes across species. Second, *TCF7* is one of only four genes consistently conserved in T‐cells across species (Jiao et al. 2024) and is uniquely linked to lifespan in the Framingham Heart Study (Figure 9e and Figure S50). Third, the TCF1 protein (encoded by *TCF7*) directly regulates essential T‐cell functions including stemness, fate determination, and stress responses—all critical components for maintaining immune resilience (Sturmlechner et al. 2023; Zhao et al. 2022). Fourth, our unbiased analyses identified five additional transcription factors (*ZBTB25*, *GATA3*, *SATB11*, *ETS1*, *KLF12*) crucial for lymphocyte and natural killer cell function that strongly correlate with optimal IR (Figure 7f) (Harly et al. 2022; Hosokawa and Rothenberg 2021; Kang and Malhotra 2015; Lambert et al. 2018; Marioni and Arendt 2017; Saini et al. 2022; Sturmlechner et al. 2023; Zhao et al. 2022). The observed sex‐specific differences in these transcription factors, with higher levels in females, align with the female longevity advantage documented across species, suggesting evolutionary conservation of these protective mechanisms (Ahuja et al. 2023; Lemaitre et al. 2020). Together, these findings define multi‐protection as a transcriptionally coordinated program integrating the optimal IR‐*TCF7* ^high^ phenotype with stress adaptation and healthspan/lifespan regulation.

### 3.3 Failed Salutogenesis: Mechanisms and Consequences

Failed salutogenesis—the inability to sustain health‐promoting mechanisms under stress—occurs when inflammatory stressors trigger a shift to extreme IR‐degrader status, with low SAS‐1 and elevated MAS‐1 gene signatures. We identified two protein groups: immune competence/trophic proteins (positive correlation with SAS‐1, inverse with MAS‐1) and inflammation‐associated proteins (opposite pattern). This creates a dual state of proinflammatory activation and immunodeficiency, increasing susceptibility to infections, comorbidities, and mortality. MAS‐1 correlating proteins are linked to inflammatory processes (e.g., IL‐6, chemokines, complement) and programmed cell death [e.g., Cathepsin B (Xie et al. 2023)]. SAS‐1 correlating biomarkers are linked to immune functions (e.g., CD23) as well as trophism (e.g., ERBB1, SPINT2, Fibronectin).

Notably, the IR program links to trophic factors, specifically components of the insulin‐like growth factor (IGF) system (Figure 9a), which are intrinsically tied to immunosuppression, aging, and cognitive declines (Conover and Oxvig 2024; Salminen et al. 2021). IGF‐1 receptor and IGFBP‐7 associate positively with SAS‐1, and IGFBP‐2 associates positively with MAS‐1 (Figure 9a). The insulin‐like signaling pathway controls growth, development, and metabolism and regulates lifespan in organisms (Conover and Oxvig 2024; Hu et al. 2009; van den Beld et al. 2012; van den Beld et al. 2019; Vitale et al. 2019). The positive correlation between IGFBP‐2 and MAS‐1 expression is noteworthy, as higher IGFBP‐2 has been correlated with increased all‐cause mortality risk in multiple human aging cohorts (Hu et al. 2009; van den Beld et al. 2012; van den Beld et al. 2019) and predicts an increased rate of incident AD and dementia (McGrath et al. 2019; Quesnel et al. 2022; Quesnel et al. 2024).

Linking IR metrics to the IGF‐1/insulin system reinforces that failed salutogenesis differs from inflammaging by arising from impaired stress adaptation rather than chronological or biological aging. This distinction has implications for addressing age‐associated decline. As inflammatory stress accumulates, the ability to sustain optimal IR‐*TCF7* ^high^ status and trophic factors diminishes alongside rising triad burden. Triad burden and *TCF7* expression correlate more strongly with IR status than age, challenging the age‐centric view that pathogenic triad burden and deficiency of *TCF7* and trophic factors are primarily consequences of aging, thereby exposing an ecological fallacy in conventional aging models (Figure 3d).

### 3.4 Challenging Age‐Centric Models: Evidence From Extreme Aging and Alzheimer's

Findings from the Vitality 90+ and Alzheimer's cohort validate the ecological fallacy, which occurs when group‐level data are misapplied to individual outcomes (Piantadosi et al. 1988). Individual‐level analyses revealed that even among nonagenarians and centenarians (Vitality 90+ cohort), those with failed salutogenesis exhibited greater triad burden and elevated cell‐free DNA levels—with corresponding increases in mortality risk. The observation that IR status predicts health outcomes even at extreme ages supports the centrality of IR mechanisms in determining lifespan and healthspan. Analysis of Alzheimer's cohorts revealed two findings (Figure 8c,d): First, while patients showed higher triad burden than controls, this disparity decreased with age, suggesting mechanisms beyond chronological aging. Second, patients maintaining optimal IR markers exhibited a reduced triad burden regardless of disease status, indicating the protective effects of preserved IR. These results position impaired salutogenic processes—rather than aging itself—as drivers of pathological trajectories. This shift suggests a reevaluation of aging biomarkers and highlights IR‐focused strategies as promising clinical approaches.

### 3.5 Mechanistic Insights Into IR and Pathogen Defense

Historical pandemics—including COVID‐19—highlight the significance of stress resilience in shaping health outcomes. The 1918 influenza pandemic triggered CVD mortality epidemics through inflammatory pathways (Azambuja 2004), mirrored in post‐COVID‐19 CVD risks (Hilser et al. 2024; Krishna et al. 2024). Our research reveals a gradient of outcomes tied to IR status at COVID‐19 presentation. Extreme IR‐degraders exhibited adverse events across three distinct timescales (Figure 6a): (1) pre‐COVID‐19 (higher comorbidity burdens, suggesting a link between susceptibility to degrade IR in response to inflammatory challenges before COVID‐19 and comorbidity burden); (2) acute COVID‐19 (higher risk for hospitalization; serious complications including respiratory failure; increased viral load; and a proinflammatory‐immunodeficient state [regardless of age], and mortality); and (3) post‐acute phase (persistent mortality). In contrast, IR‐preservers exhibited a lower comorbidity burden before COVID‐19 and near‐complete protection against hospitalization and mortality across all age groups during acute infection.

Mechanistically, individuals with preserved optimal IR demonstrated three synergistic advantages that explain their superior disease outcomes. First, IR‐preservers exhibited robust cross‐pathogen defense, evidenced by reduced symptomatic responses in controlled influenza and typhoid challenge studies, suggesting conserved protective immune mechanisms across diverse pathogens. Second, they displayed distinctive COVID‐19 survival‐associated immune/inflammatory signatures, particularly elevated mucosal‐associated invariant T cells that effectively bridge innate and adaptive immunity systems (Parrot et al. 2020). Third, IR‐preservers demonstrated superior humoral optimization, characterized by stronger neutralizing antibody responses against SARS‐CoV‐2 during both natural infection and post‐vaccination, alongside immune profiles correlating with enhanced influenza and SARS‐CoV‐2 vaccine efficacy. These findings collectively suggest that optimal IR represents an evolutionary adaptation with dual protective benefits: simultaneously reducing acute infectious mortality while limiting chronic cardiometabolic sequelae.

### 3.6 Resolving Longevity Paradoxes Through Immune Resilience Framework

While optimal IR‐*TCF7* ^high^ confers survival benefits, these are constrained by evolutionarily relevant timeframes across the lifespan. In aging and post‐acute COVID‐19 contexts, these benefits are limited to the biological “warranty period” (less than 70 years) and help mitigate premature mortality (Figure 1a). In a general population cohort (the Framingham Heart Study), 40‐year‐olds with extreme IR‐degrader status faced a 9.7‐fold increase in mortality risk—equivalent to the risk observed in 55.5‐year‐olds with optimal IR. This disparity highlights a 15.5‐year survival gap favoring those with optimal IR. Survival benefits peak in mid‐adulthood (ages 40–70; with a 69% reduced mortality rate)—a critical intervention window—after which mortality rates converge between resilient and non‐resilient individuals, suggesting a biological ceiling for longevity interventions beyond this period.

Beyond the warranty period, the escalation of mortality risk linked to age reduces the protective effects of optimal IR‐*TCF7* ^high^, paralleled by a rise in IR‐degrader prevalence (Figure 2b). This reduction places constraints on human longevity, aligning with established lifespan limitations (Olshansky et al. 2024; Vaupel 2010). Therefore, failed salutogenesis—marked by progressive accumulation of IR‐degraders—drives these boundaries, highlighting the tension between conserved adaptations and age‐related decline.

James Vaupel's two longevity paradoxes (Vaupel 2010) offer insights into health trajectories linked to aging processes. The first paradox, termed the ‘longevity riddle,’ questions why evolutionary forces permit adjustments to health levels but not debilitation rates—a reflection of longevity arising through better health maintenance rather than slower aging rates. The second paradox highlights substantial variation in health levels across age groups despite consistent aging rates. Our research resolves these paradoxes through three interconnected findings: evolutionary selection for *TCF7‐*linked salutogenic traits promoting resilience; individual differences sustaining these traits driving health variations; reprogramming IR status enabling selective malleability of health maintenance without altering aging rates. This framework reconciles conflicting observations by positioning IR status as a key determinant of health outcomes.

In summary, our research identifies IR degradation as an age‐independent, inflammatory stress‐dependent accelerator of aging phenotypes. We advance understanding of lifespan limits through our framework, demonstrating the necessity of preserving health‐promoting IR mechanisms (salutogenesis) and countering disease drivers (pathogenesis). This approach conceptualizes aging as a dynamic interplay between resilience and decline, aligning with geroscience priorities (Ferrucci et al. 2024). Our findings establish *TCF7*‐linked IR status as a biomarker for longevity thresholds, enabling assessment and modulation of aging trajectories.

### 3.7 Therapeutic Implications and Future Directions

Our analysis identified proteomic targets in the IR‐associated program (Figure 9a) with therapeutic potential: modulation of SAS‐1 sustains immunocompetence and promotes trophic factors, while targeting MAS‐1 attenuates inflammatory mediators, revealing key pathways for healthspan extension. TNFα blockers effectively counteract the inflammatory component of IR, underscoring their translational potential. The efficacy of senolytic agents (Guo et al. 2022; Kennedy et al. 2014; Lopez‐Otin et al. 2023) under extreme IR degradation warrants investigation. Our findings highlight mid‐adulthood as a critical, malleable window for modulating in IR. This research transitions from pathogenesis‐focused models to IR optimization strategies, providing actionable pathways to extend healthspan and lifespan through precision interventions aligned with emerging geroscience priorities and proactive preventive approaches.

## 4 Methods

All studies were approved by the institutional review boards (IRBs) at the University of Texas Health Science Center at San Antonio and institutions participating in this study. All studies adhered to ethical and inclusion practices approved by the local IRB. The cohort details (Tables S5–S7 and S15), experimental methods, analytical plan, and detailed statistical methods used to test for significant differences shown in the figures and tables are detailed in the Supporting Information. FDR‐adjusted *P* values are shown where adjustment for multiple corrections was performed. The number of samples per group (*n*) and statistical methods used are briefly mentioned in the figure legends and are detailed in the Supporting Information.

## Author Contributions

S.K.A. conceived of the idea of salutogenesis and immunologic resilience and its metrics; designed, supervised, and coordinated the study; interpreted the data; and wrote the manuscript. M.S.M. and J.A.M. analyzed publicly available data and performed biostatistical analyses. M.S.M., J.A.M., K.A., and M.N. assembled the Supporting Information. G.C.L. assisted in assembling the COVID‐19 and SAIL cohorts; assisted in the interpretation of the data; and provided conceptual contributions. N.H. performed all statistical analyses for the COVID‐19 and SARS‐CoV‐2 vaccine cohorts. M.M. performed additional statistical analyses. F.J. designed and performed the immunologic assessments with assistance from A.P.B. F.J., N.Z., and A.A.G. interpreted immunologic data. F.J., A.P.B., L.P., and W.H. performed additional experiments and biobanking. M.I.R., G.C.L., A.P.B., J.A.M., A.M.S., L.P., M.S.M., D.T., H.D.T., S.S.‐R., A.G.M., C.B., J.F.O., and W.H. assisted in the assembly of the cohorts along with members of the STVHCS COVID‐19 clinical, vaccine, and convalescent teams and members of the Center for Personalized Medicine. C.B., L.A., and L.N.S. assisted in developing the NYU COVID‐19 cohort. All authors provided editorial suggestions with key inputs from G.C.L., M.S.M., J.A.M., N.H., S.K., K.A., M.N., S.N., R.A.C., and J.F.O. The order of the authors, including co‐first and co‐second authorships, was determined by the relative contributions to this study.

## Consent

All work presented in the research paper is original and no permission is required. Figures adapted from others' work are cited with appropriate references.

## Conflicts of Interest

The authors declare no conflicts of interest.

## Supporting information


Appendix S1.

Appendix S2.

Appendix S3.

## Data Availability

All data, code, and materials used in the analysis will be available to any researcher for the purposes of reproducing or extending the analysis. The phenotype and processed gene expression data generated are available in the Figshare database for the VA‐CLC baseline (https://figshare.com/projects/Ahuja_Lab_COVID‐19_whole_blood_baseline_dataset_n_48/158732) and VA‐CLC longitudinal (https://figshare.com/projects/Ahuja_Lab_COVID‐19_Whole_Blood_longitudinal_dataset_VA‐CLC_n_330_/194474) NYU cohort (https://figshare.com/projects/NYU_COVID‐19_Whole_Blood_Longitudinal_dataset_NYU_COVID‐19_WB_n_265_/239717). The data for VA‐CLC longitudinal and NYU cohort will be made publicly available upon acceptance and publication. Individual level raw data files of the VA‐CLC and NYU COVID‐19 cohorts cannot be shared publicly due to data protection and confidentiality requirements. South Texas Veterans Health Care System (STVHCS) at San Antonio, Texas, is the data holder for the VA‐CLC and SAIL data used in this study. New York University is the data holder for the NYU data used in this study. Data can be made available to approved researchers for analysis after securing relevant permissions via review by the IRB for use of the data collected under this protocol. Inquiries regarding data availability should be directed to the corresponding author. All other patient/individual‐level raw data underlying this article cannot be shared publicly due to data protection and confidentiality requirements. Data analysis was performed in R and the R scripts are available from the corresponding author on request.

## Appendix A

Additional members of the **STVHCS Center for Personalized Medicine**:

Cody R. Butler, Andrew Carrillo, Chanda Dhami, Gaeun Jo, Krupa Jiva, Peter Melby, Ernesto Robinson, Erin Stewart, Caitlyn A. Winter, Lauryn A. Winter, Joseph M. Yabes.

Additional members of the **STVHCS COVID‐19 Clinical team**:

Mohamed I. Abdalla^a^, Sandra G. Adams^a^, Yemi Adebayo^a^, Joseph Agnew^a^, Saleem Ali^a^, Gregory Anstead^a^, Antonio Anzueto^a^, Marichu Balmes^a^, Jennifer Barker^a^, Raymond Benavides^b^, Velma Bible^a^, Angela Birdwell^a^, Stacy Braddy^a^, Stephen Bradford^a^, Heather Briggs^a^, Jose A. Cadena‐Zuluaga^a^, Judith Marin‐Corral^a^, Jennifer J. Dacus^a^, Patrick J. Danaher^a^, Scott A. DePaul^a^, Jill Dickerson^a^, Jollynn Doanne^a^, Samantha Elbel^a^, Miguel Escalante^a^, Corina Escamilla^a^, Valerie Escamilla^a^, Robert Farrar^a^, David Feldman^a^, Debra Flores^a^, Julianne Flynn^a^, Delvina Ford^a^, Joanna D. Foy^a^, Megan Freeman^a^, Samantha Galley^a^, Jessica Garcia^a^, Maritza Garza^a^, Sherraine Gilman^a^, Melanie Goel^a^, Jennifer Gomez^a^, Varun K. Goyal^a^, Sally Grassmuck^a^, Susan Grigsby^a^, Joshua Hanson^a^, Brande Harris^a^, Audrey Haywood^a^, Joan M. Hecht^a^, Cecilia Hinojosa^a^, Tony T. Ho^a^, Teri Hopkins^a^, Aneela N. Hussain^a^, Ali Jabur^a^, Pamela Jewell^a^, Thomas B. Johnson^a^, Austin C. Lawler^a^, Monica Lee^a^, Chadwick S. Lester^a^, Stephanie M. Levine^a^, Haidee V. Lewis^a^, Angel Louder^a^, Charmaine Mainor^a^, Rachel Maldonado^a^, Celida Martinez^a^, Yvette Martinez^a^, Chloe Mata^a^, Neil McElligott^a^, Laura Medlin^a^, Myra Mireles^a^, Joanna Moreno^a^, Kathleen Morneau^a^, Julie Muetz^a^, Samuel B. Munro^a^, Charlotte Murray^a^, Anoop Nambiar^a^, Daniel Nassery^a^, Robert Nathanson^a^, Kimberly Oakman^a^, Jane O'Rorke^a^, Cheryl Padgett^a^, Sergi Pascual‐Guardia^a^, Marisa Patterson^a^, Graciela L. Perez^a^, Rogelio Perez^a^, Rogelio Perez III^a^, Jay I. Peters^a^, Robert E. Phillips^a^, Patrick B. Polk^a^, Michael A. Pomager^a^, Kristy J. Preston^a^, Kevin C. Proud^a^, Jacqueline A. Pugh^a^, Michelle Rangel^a^, Temple A. Ratcliffe^a^, Renee L. Reichelderfer^a^, Evan M. Renz^a^, Jeanette Ross^a^, Teresa Rudd^a^, Maria E. Sanchez^a^, Tammy Sanders^a^, Kevin C. Schindler^a^, David Schmit^a^, Raj T. Sehgal^a^, Claudio Solorzano^a^, Nilam Soni^a^, Win S. Tam^a^, Edward J. Tovar^a^, Sadie A. Trammell Velasquez^a^, Anna R. Tyler^a^, Anjuli Vasquez^a^, Maria C. Veloso^a^, Steven G. Venticinque^a^, Jorge A. Villalpando^a^, Melissa Villanueva^a^, Lauren Villegas^a^, Megan Walker^a^, Andrew Wallace^a^, Maria Wallace^a^, Emily Wang^a^, Stephanie Wickizer^a^, Andreia Williamson^a^, Andrea Yunes^a^, Katharine H. Zentner^a^

^a^South Texas Veterans Health Care System, San Antonio, TX, USA 78229; ^b^Department of Pharmacy, University of Texas Health San Antonio, San Antonio, TX, USA 78229.

Additional members of the **STVHCS COVID‐19 Vaccine team**:

Azaneth Arellanes, Ashley B. Banfield, Stephanie N. Bolan‐Reding, Roxanne Colazo, Katherine S. DeLeon, Norma G. Diaz, Samantha Elbel, Mario A. Garza, Raed Kadhume, Hue Mang, Erwin Paleracio, Robert F. Quitta, Laura J. Ramirez, Marzieh Salehi, Cynthia J. Varela.

South Texas Veterans Health Care System, San Antonio, TX, USA 78229.

Additional members of the **STVHCS COVID‐19 Convalescent team**:

Yemi Adebayo, Marichu Balmes, Velma Bible, Stacy Braddy, Valerie Escamilla, Miguel Escalante, Debra Flores, Jessica Garcia, Maritza Garza, Melanie Goel, Susan Grigsby, Aneela N. Hussain, Ali Jabur, Thomas B. Johnson, Chloe Mata, Joanna Moreno, Julie Muetz, Charlotte Murray, Rogelio Perez III, Anjuli Vasquez, Megan Walker, Maria Wallace, Stephanie Wickizer.

South Texas Veterans Health Care System, San Antonio, TX, USA 78229.
