# Supplementary material for: The 15‐Year Survival Advantage: Immune Resilience as a Salutogenic Force in Healthy Aging
Source: Aging Cell. 2025 Apr 23;24(7):e70063. doi: 10.1111/acel.70063 (PMC12266754; doi:10.1111/acel.70063)
Supplement: Supplementary file 1 — Appendix S1. [file ACEL-24-e70063-s002.pdf]

AGING CELL AUTHOR CHECKLIST. ***Authors should submit this checklist together with their manuscript. Please ensure that you have read the Author Guidelines in detail before submission.***

**Title**	**The 15-Year Survival Advantage: Immune Resilience as a Salutogenic Force in Healthy Aging**						
**Authors**	Manoharan et al						
**Manuscript Type**	Research article						
**Total Character Count (including spaces)[1]**	92,125						
**Word count of Summary[2]**	251						
**Number of papers cited in the References[3]**	76						
**Listing of all Tables (Table1, Table 2 etc)[4]**	None						
**Figure specifications (please complete one row per figure)[5]**<br><br>***Figure no.***	Colour<br>***(yes/no)***	Greyscale<br>***(yes/no)***	Black and white<br>***(yes/no)***	Single column (80mm)<br>***(yes/no)***	Double column (180mm)<br>***(yes/no)***	Size of figure at full scale (mm x mm)<br>***(insert details)***	Smallest font size used in the figure at full scale (minimum 6pt)<br>***(insert details)***
1	yes				yes	177 x 228	6pt
2	yes				yes	178 x 215	6pt
3	yes				yes	174 x 216	6pt
4	yes				yes	176 x 209	6pt
5	yes				yes	177 x 211	6pt
6	yes				yes	179 x 208	6pt
7	yes				yes	177 x 219	6pt
8	yes				yes	158 x 216	6pt
9	yes				yes	162 x 221	6pt

[1] The maximum character count allowed is 50,000 (incl. spaces) for Primary Research Papers and Reviews, 10,000 for Short Takes.

[2] Summary should not exceed 250 words.

[3] Primary Research Papers can contain a maximum of two tables. If more are needed they should replace some of the Figures or can be placed in the Supporting Information.

[4] A maximum of 45 references is allowed for Primary Research Papers and 20 references for Short Takes.

[5] A Primary Research Paper may contain up to 6 figures and a Short Take up to 2 figures. Authors are encouraged to provide figures in the size they are to appear in the journal and at the specifications given.
