# Supplementary material for: The 15‐Year Survival Advantage: Immune Resilience as a Salutogenic Force in Healthy Aging
Source: Aging Cell. 2025 Apr 23;24(7):e70063. doi: 10.1111/acel.70063 (PMC12266754; doi:10.1111/acel.70063)
Supplement: Supplementary file 2 — Appendix S2. [file ACEL-24-e70063-s003.pdf]

Supplementary Information

The 15-Year Survival Advantage: Immune Resilience as a Salutogenic Force in Healthy Aging

Muthu Saravanan Manoharan, M.S.[1,2*†], Grace C. Lee, PharmD., Ph.D.[1,3,4,5*†‡§], Nathan Harper, M.S.[1,5*†], Justin A. Meunier, B.S.[1,5*†], Marcos I. Restrepo, M.D., MSc, Ph.D.[1,2,5,6¶†‡§], Fabio Jimenez, B.S.[1,5¶†], Sreenath Karekatt Ph.D.[1,2¶†], Anne P. Branum, B.S.[1,5¶†], Alvaro A. Gaitan, M.D.[1,5¶†], Kian Andampour, B.S.[1,2†], Alisha M. Smith, Ph.D.[1,5,6†§], Michael Mader, M.S.[7], Michelle Noronha, B.S.[1,2†], Devjit Tripathy, M.D.[2.7†§], Nu Zhang, Ph.D.[1,6†], Alvaro G. Moreira, M.D.[1,8†], Lavanya Pandranki, M.S.[1,2†], South Texas Veterans Health Care System (STVHCS) COVID-19 Clinical team[‡], STVHCS COVID-19 Vaccine team[§], STVHCS COVID-19 Convalescent care team[#], STVHCS Center for Personalized Medicine[†], Sandra Sanchez-Reilly, M.D.[2,7‡§#], Hanh Trinh, M.D.[7‡§#], Clea Barnett, M.D.[9], Luis Angel, M.D.[9], Leopoldo N. Segal, M.D.[9], Susannah Nicholson, M.D.[10], Robert A. Clark, M.D.[1,2,7†], Weijing He, M.D.[1,5¶†], Jason F. Okulicz, M.D.[11], Sunil K. Ahuja, M.D.[1,2,6,7†‡§**]

[1]Veterans Affairs Center for Personalized Medicine, South Texas Veterans Health Care System, San Antonio, TX, USA 78229

[2]Department of Medicine, University of Texas Health Science Center at San Antonio, San Antonio, TX, USA 78229

[3]Pharmacotherapy Education and Research Center, School of Medicine, University of Texas Health Science Center at San Antonio, San Antonio, TX, USA 78229

[4]College of Pharmacy, The University of Texas at Austin, Austin TX, USA 78712
[5]The Foundation for Advancing Veterans' Health Research, San Antonio, TX, USA 78229
[6]Department of Microbiology, Immunology & Molecular Genetics, University of Texas Health Science Center at San Antonio, San Antonio, TX, USA 78229
[7]South Texas Veterans Health Care System, San Antonio, TX, USA 78229
[8]Department of Pediatrics, University of Texas Health Science Center at San Antonio, San Antonio, TX, USA 78229
[9]Division of Pulmonary and Critical Care Medicine, Department of Medicine, New York University Grossman School of Medicine, NYU Langone Health, New York, NY, USA 10016
[10]Department of Surgery, University of Texas Health Science Center at San Antonio, San Antonio, TX, USA 78229
[11]Gilead Sciences, Foster City, CA, USA 94404

*Contributed equally as first authors
¶Contributed equally as second authors
†South Texas Veterans Health Care System (STVHCS) Center for Personalized Medicine
‡STVHCS COVID-19 Clinical team
§STVHCS COVID-19 Vaccine team
#STVHCS COVID-19 Convalescent care team
**Address correspondence to:
ahujas@uthscsa.edu; Sunil K. Ahuja, M.D., Department of Medicine, University of Texas Health Science Center at San Antonio, 7703 Floyd Curl Drive, San Antonio, TX, USA 78229. Phone: 210-450-7277

Contents

Abbreviations ***7***

Conflict of Interest ***10***

STVHCS Center for Personalized Medicine ***10***

STVHCS COVID-19 clinical team ***10***

STVHCS COVID-19 vaccine team ***11***

STVHCS COVID-19 convalescent team ***11***

Disclaimer ***12***

Acknowledgments ***12***

Author contributions ***13***

Supplementary materials and methods ***15***

1. Cohorts studied **15**

1.1. Veterans Affairs COVID-19 Longitudinal Cohort (VA-CLC) developed at the South Texas Veterans Health Care System (STVHCS), San Antonio 15

1.1a. Acute COVID-19 (ac-COVID-19) component of the cohort 15

1.1b. Data collection and sources 16

1.1c. Demographic and clinical characteristics and definitions 16

1.1d. VA-CLC post-acute lifespan subset 19

1.1e. VA-CLC with available pre-COVID-19 IHG data 19

1.2. STVHCS acute COVID-19 outbreak cohort 19

1.3. San Antonio Immunologic Resilience Longitudinal (SAIL) cohort: pre-booster vaccine cohort of persons without acute COVID-19 20

1.4. Non-acute: VA-convalescence program subset 20

1.5. Reference populations for IR assignments of IHGs 21

1.5a. SardiNIA 22

1.5b. HIV− cohort from the University of California San Diego (termed HIV− UCSD cohort) 22

1.5c. The Kenya Majengo Observational Cohort Study cohort (MOCS) 22

1.5d. US Military HIV+ Natural History Study (NHS) Cohort 23

1.6. Primary Infection HIV+ cohort from the University of California San Diego (termed HIV+ PIC cohort) 24

2. Sample processing methods **24**

2.1. Peripheral blood mononuclear cell (PBMC) isolation 24

2.2. Surrogate virus neutralization test (sVNT) methods and evaluations 25

2.3. SARS-CoV-2 Viral Load 25

2.4. Flow cytometry 26

2.5. Transcriptomic profiling 27

3. Data download and normalization **28**

3.1. Microarray datasets 28

3.2. RNA-seq datasets 28

3.3. Probe-Gene information 29

3.4. Single-cell RNA-seq (scRNA-seq) 29

3.5. Quality control of the dataset and interpretation 30

4. Transcriptomic signatures and analyses 30
4.1. SAS and MAS 30
4.1a. SAS/MAS profiles 32
4.2. Methods for IMM-AGE transcriptomic signature score 32
4.3. Extreme Longevity (EL) signatures 33
4.4. SenMayo signature 33
4.5. Age_IL6 signatures 34
4.6. InflammΔage signatures 34
4.7. Tri-partite T-cell health (t-TCH) signatures 35
4.8. Blood transcription modules 35
4.9. Interferon-stimulated genes (ISG) signatures 36
4.10 Gene expression datasets 36
4.11. Transcription factor analysis 43

5. Statistical approach and plots 46
5.1. Clustering 46
5.2. Timing of assessments 46
5.3. Associations of %inhibition levels 46
5.4. Multiomic associations 47
5.5. General statistical methods 48
5.6. Plots 48
5.7. Statistical approach: Principles 48
5.7a. Rationale for studying distinct cohorts and the comparisons made 49
5.7b. Biological plausibility and statistical approach 50
5.7c. Multiple comparisons 50

6. Statistical analyses 51
6.1. Main figure statistical methods used in each figure panel 51
6.2. Supplementary figure statistical methods used in each figure panel 62

7. Supplementary Note: Rationale for derivation of IHGs. 85

8. Supplementary Figures 92
Figure S1. 92
Figure S2. 93
Figure S3. 94
Figure S4. 105
Figure S5. 107
Figure S6. 113
Figure S7. 115
Figure S8. 119
Figure S9. 121
Figure S10. 124
Figure S11. 126
Figure S12. 130
Figure S13. 134
Figure S14. 138

Figure S15. 142
Figure S16. 146
Figure S17. 151
Figure S18. 156
Figure S19. 161
Figure S20. 166
Figure S21. 171
Figure S22. 173
Figure S23. 175
Figure S24. 177
Figure S25. 178
Figure S26 179
Figure S27. 181
Figure S28. 183
Figure S29. 184
Figure S30 185
Figure S31. 187
Figure S32. 189
Figure S33. 191
Figure S34. 193
Figure S35. 195
Figure S36. 197
Figure S37. 199
Figure S38. 202
Figure S39. 206
Figure S40. 209
Figure S41. 211
Figure S42. 212
Figure S43. 213
Figure S44. 217
Figure S45. 219
Figure S46. 222
Figure S47. 223
Figure S48. 225
Figure S49 227
Figure S50. 229

9. Supplementary Tables 231
Table S1. 231
Table S2. 231
Table S3. 231
Table S4. 231
Table S5. 231
Table S6. 232
Table S7. 232
Table S8. 232

Table S9. 232
Table S10. 232
Table S11. 232
Table S12. 232
Table S13. 233
Table S14. 233
Table S15. 233

10. References 234

Abbreviations

ac, acute
AD, Alzheimer's disease
adj, model adjustment
AF, attributable fraction AIC, Akaike information criterion
ANOVA, analysis of variance
AR, absolute risk
AR(1), autoregressive 1 (correlation structure)
ARDS, acute respiratory distress syndrome
BMI, body mass index
C19, COVID-19 (coronavirus cisease-2019)
CAD, coronary artery disease
cDC, conventional dendritic cells
cfDNA, cell-free DNA
CHF, congestive heart failure
CI, confidence interval
CKD, chronic kidney disease
CM, central memory
CMV, cytomegalovirus
COPD, chronic obstructive pulmonary disease
CRP, c-reactive protein
CS, comorbidity score
Ct, SARS-CoV-2 cycle threshold
CVD, cardiovascular disease

dbGaP: Database of Genotypes and Phenotypes
DC, dendritic cells
DE, differentially expressed
DENV, dengue virus
DHF, dengue hemorrhagic fever
DoS, days since onset of symptoms
DSS, dengue shock syndrome
EDS, estimate date of seroconversion
EL, extreme longevity
EM, effector memory
FDR, false discovery rate
FHS, Framingham Heart Study
GALT, gut-associated lymphoid tissue
gd, gamma-delta
GEE, generalized estimating equation

GLM, generalized linear model
GO-BP, gene ontology biological process
Grp, group
GSEA, gene set enrichment analysis
H, hospitalized
H-L, SAS-1^{high}-MAS-1^{low}
H-S, hospitalized survivors
HCW, healthcare workers
HIV, human immunodeficiency virus
HR, hazard ratio
hRV, human rhinovirus
HTN, hypertension
IC, immunocompetence
IF, inflammation
IFN, interferon
IHG, immune health grade
ILD, interstitial lung disease
Int, intercept
IQR, interquartile range
IR, immune resilience
ISG, interferon-stimulated genes
JIA, juvenile idiopathic arthritis
KD, Kawasaki disease
KM, Kaplan-Meier plot
L-H, SAS-1^{low}-MAS-1^{high}
LRT, likelihood ratio test
LTBI, latent tuberculosis infection
MAIT, mucosal-associated invariant T cell
MAS, mortality-associated signature
MID, monogenic immunodeficiency
MIQ, median interquartile range
MOCS, Majengo Observational Cohort Study
MTF, military treatment facilities
MV, mechanical ventilation
MZ, marginal zone
nAb, neutralizing antibody
NCBI, National Center for Biotechnology Information
NE, neutrophils
NGE, normalized gene expression
NH, nonhospitalized

NHS, Natural History Study HIV+ cohort
NK, natural killer cells
NS, nonsurvivors (30-days)
NTM, non-tuberculosis mycobacteria
NYU, New York University
OR, odds ratio
PAF, partially attributable fraction
PB, peripheral blood
PVD, peripheral vascular disease
r, correlation coefficient
RBC, red blood cells
REDCap, Research Electronic Data Capture
RNA-Seq, RNA sequencing
RR, rate ratio
RSV, respiratory syncytial virus
SAIL, San Antonio Immunologic Resilience Longitudinal cohort
SAS, survival-associated signature
SAFHS, San Antonio Family Heart Study
SCS, serious complication score
SEM, standard error of mean
SLE, systemic lupus erythematosus
SLEDAI, SLE disease activity index
STVHCS, South Texas Veterans Health Care System
TB, tuberculosis
TD, typhoid diagnosis
t-TCH, tripartite T-cell health
TEMRA, effector memory T-cells re-expressing CD45RA
TF, transcription factor
TLR, toll-like receptor
UCSD, University of California San Diego
VA, Department of Veterans Affairs
VHA, Veterans Healthcare Administration
VL, viral load

VTE, venous thromboembolism

Conflict of Interest

The authors have declared that no conflict of interest exists.

STVHCS Center for Personalized Medicine

Additional members of the **STVHCS Center for Personalized Medicine** include:

Cody R. Butler, Andrew Carrillo, Chanda Dhami, Gaeun Jo, Krupa Jiva, Peter Melby, Ernesto Robinson, Erin Stewart, Caitlyn A. Winter, Lauryn A. Winter, Joseph M. Yabes

STVHCS COVID-19 clinical team

Additional members of the **STVHCS COVID-19 Clinical team** include:

Mohamed I. Abdalla, Sandra G. Adams, Yemi Adebayo, Joseph Agnew, Saleem Ali, Gregory Anstead, Antonio Anzueto, Marichu Balmes, Jennifer Barker, Raymond Benavides***, Velma Bible, Angela Birdwell, Stacy Braddy, Stephen Bradford, Heather Briggs, Jose Cadena Zuluaga, Judith Marin Corral, Jennifer J. Dacus, Patrick J. Danaher, Scott A. DePaul, Jill Dickerson, Jollynn Doanne, Samantha Elbel, Miguel Escalante, Corina Escamilla, Valerie Escamilla, Robert Farrar, David Feldman, Debra Flores, Julianne Flynn, Delvina Ford, Joanna D. Foy, Megan Freeman, Samantha Galley, Jessica Garcia, Maritza Garza, Sherraine Gilman, Melanie Goel, Jennifer Gomez, Varun K. Goyal, Sally Grassmuck, Susan Grigsby, Joshua Hanson, Brande Harris, Audrey Haywood, Joan M. Hecht, Cecilia Hinojosa, Tony T. Ho, Teri Hopkins, Aneela N. Hussain, Ali Jabur, Pamela Jewell, Thomas Brent Johnson, Austin C. Lawler, Monica Lee, Chadwick S. Lester, Stephanie M. Levine, Haidee V. Lewis, Angel Louder, Charmaine Mainor, Rachel Maldonado, Celida Martinez, Yvette Martinez, Chloe Mata, Neil McElligott, Laura Medlin, Myra Mireles, Joanna Moreno, Kathleen Morneau, Julie Muetz, Samuel B. Munro, Charlotte Murray, Anoop Nambiar, Daniel Nassery, Robert Nathanson, Kimberly Oakman, Jane O'Rorke, Cheryl Padgett, Sergi Pascual-Guardia, Marisa Patterson, Graciela L. Perez, Rogelio Perez, Rogelio Perez III, Jay I. Peters, Robert E. Phillips, Patrick B. Polk, Michael A. Pomager, Kristy J. Preston, Kevin C. Proud, Jacqueline A. Pugh, Michelle Rangel, Temple A. Ratcliffe, Renee L. Reichelderfer, Evan M Renz, Jeanette Ross, Teresa Rudd, Maria E. Sanchez, Tammy Sanders, Kevin C.

Schindler, David Schmit, Raj T. Sehgal, Claudio Solorzano, Nilam Soni, Win S. Tam, Edward J. Tovar, Sadie A. Trammell Velasquez, Anna R. Tyler, Anjuli Vasquez, Maria C. Veloso, Steven G. Venticinque, Jorge A. Villalpando, Melissa Villanueva, Lauren Villegas, Megan Walker, Andrew Wallace, Maria Wallace, Emily Wang, Stephanie Wickizer, Andreia Williamson, Andrea Yunes, Katharine H. Zentner

Affiliations: South Texas Veterans Health Care System, San Antonio, TX, USA 78229; ***Department of Pharmacy, University of Texas Health San Antonio, San Antonio, TX, USA 78229.

STVHCS COVID-19 vaccine team

Additional members of the **STVHCS COVID-19 Vaccine team** include:

Azaneth Arellanes, Ashley B. Banfield, Stephanie N. Bolan-Reding, Roxanne Colazo, Katherine S. DeLeon, Norma G. Diaz, Samantha Elbel, Mario A. Garza, Raed Kadhume, Hue Mang, Erwin Paleracio, Robert F. Quitta, Laura J. Ramirez, Marzieh Salehi, Cynthia J. Varela

Affiliation: South Texas Veterans Health Care System, San Antonio, TX, USA 78229

STVHCS COVID-19 convalescent team

Additional members of the **STVHCS COVID-19 Convalescent team** include:

Yemi Adebayo, Marichu Balmes, Velma Bible, Stacy Braddy, Valerie Escamilla, Miguel Escalante, Debra Flores, Jessica Garcia, Maritza Garza, Melanie Goel, Susan Grigsby, Aneela N. Hussain, Ali Jabur, Thomas B. Johnson, Chloe Mata, Joanna Moreno, Julie Muetz, Charlotte Murray, Rogelio Perez, Anjuli Vasquez, Megan Walker, Maria Wallace, Stephanie Wickizer

Affiliation: South Texas Veterans Health Care System, San Antonio, TX, USA 78229

Disclaimer

The views expressed herein are those of the authors and do not reflect the official policy or position of Brooke Army Medical Center, the U.S. Army Medical Department, the U.S. Army Office of the Surgeon General, the Department of the Army, the Department of the Air Force, or the Department of Defense or the U.S. Government.

Acknowledgments

The two main sources of funding for the data presented herein are those awarded to SKA, JFO, and MIR. SKA was supported by grants from the Veterans Affairs (VA) [VA Research Center for AIDS and HIV Infection, VA Center for Personalized Medicine (IP1 CX000875-01A1); the National Institutes of Health (NIH) MERIT award (R37AI046326); the Doris Duke Distinguished Clinical Scientist Award; the Elizabeth Glaser Pediatric AIDS Foundation; and the Burroughs Wellcome Clinical Scientist Award in Translational Research. The work was also supported, in part, by an award jointly funded by the National Institute of Allergy and Infectious Diseases (NIAID)/NIH (#AAI20042-001) and the Department of Veterans Affairs (COVID19-8100-01) awarded to SKA and MIR. A portion of the data presented is based on research sponsored by the U.S. Air Force under agreement number FA8650-17-2-6816 (United States Air Force 59th Medical Wing Intramural Award to JFO). This work was also supported by NIH grant 1UL1 TR002645 (Clinical and Translational Science Award to RAC). GCL was supported by the NIH K23-AG066933. AMS was supported by the NIH T32DE014318 COSTAR institutional research training grant and Joe and Patty Robles Endowed Post-Doctoral Fellow in Military Health. LNS was supported by the NIH R21 GM147800 award.

Framingham Heart Study dbGaP Acknowledgement Statement: The Framingham Heart Study is conducted and supported by the National Heart, Lung, and Blood Institute (NHLBI) in collaboration with Boston University (Contract No. N01-HC-25195 and HHSN268201500001I). This manuscript was not prepared in collaboration with investigators of the Framingham Heart Study and does not necessarily reflect the opinions or views of the Framingham Heart Study, Boston University, or NHLBI.

Additional funding for SABRe was provided by Division of Intramural Research, NHLBI, and Center for Population Studies, NHLBI.

MID cohort was supported by the National Heart, Lung, and Blood Institute (NHLBI; ZIA-HL006089-12, protocol: 04-H-0012); National Human Genome Research Institute (NHGRI; 1ZIAHG200371, 1ZIAHG200373, and 1ZIAHG200374); National Institute for Allergy and Infectious Diseases (NIAID; protocols: 00-I-0159, 01-I-0202, 07-I-0033, 13-I-0157, 15-I-0162, 91-I-0140, and 93-I-0119) and the corresponding NIH grant numbers associated with this program (Z-01-A-00647, Z-01-A-00646, 1 ZIA AI001202-07, Z01 AI000825-24 LIR, Z-01-A-00646). The original data description for the NIH Division of Intramural Research Multiomic Monogenic Disease Study can be found here: (Journal Citation: Nat Med. 2024 Sep;30(9):2461-2472. doi: 10.1038/s41591-024-03092-6. Epub 2024 Jul 3.) (Sparks et al., 2024). The original data used for the analysis in this publication can be found in the NIH database of Genotypes and Phenotypes (dbGaP) under the accession numbers: phs001860.v1.p1 and phs002732.v1.p1.

Author contributions

SKA conceived the idea of salutogenesis and immunologic resilience and its metrics; designed, supervised, and coordinated the study; interpreted the data; and wrote the manuscript. MSM and JAM analyzed publicly available data and performed biostatistical analyses. MSM, JAM, KA, and MN assembled the supplementary materials. GCL assisted in assembling the COVID-19 and SAIL cohorts; assisted in interpretation of the data; and provided conceptual contributions. NH performed all statistical analyses for the COVID-19 and SARS-CoV-2 vaccine cohorts. MM performed additional statistical analyses. FJ designed and performed the immunologic assessments with assistance from APB. FJ, NZ, and AAG interpreted immunologic data. FJ, APB, LP, and WH performed additional experiments and biobanking. MIR, GCL, APB, JAM, AMS, LP, MSM, DT, HT, SS-R, AGM, CB, JFO, and WH assisted in the assembly of the cohorts along with members of the STVHCS COVID-19 clinical, vaccine, and convalescent teams and members of the Center for Personalized Medicine. CB, LA, and LNS assisted in developing the NYU COVID-19 cohort. All authors provided editorial suggestions with key

inputs from GCL, MSM, JAM, NH, SK, KA, MN, SN, RAC, and JFO. The order of the authors, including co-first and co-second authorships, was determined by the relative contributions to this study.

Supplementary materials and methods

1. Cohorts studied

All studies were approved by the institutional review boards at the University of Texas Health San Antonio and institutions participating in this study.

1.1. Veterans Affairs COVID-19 Longitudinal Cohort (VA-CLC) developed at the South Texas Veterans Health Care System (STVHCS), San Antonio

Characteristics and features of the components of the VA-CLC are in **Table S5**.

1.1a. Acute COVID-19 (ac-COVID-19) component of the cohort

This is an observational, single-center, prospective cohort of individuals with at least one positive test for SARS-CoV-2 from the beginning of the pandemic through December 31, 2021 (*n*=2,042). This period spanned the timeframe in Texas in which pre-Delta and Delta strains were prevalent. Some characteristics of the COVID-19 cohort and the nature of the biological sampling from patients have been described previously (Lee et al., 2021); characteristics of the cohort studied herein are summarized in **Table S5a**. Patients were monitored for outcomes until death or loss to follow-up until October 31, 2022, allowing for a minimum of 10 months of follow-up. Standard laboratory methods in the Flow Cytometry Core of the Central Pathology Laboratory at the Audie L. Murphy VA Medical Center were used to determine peripheral blood $CD4^+$ and $CD8^+$ T-cell levels, as well as levels of total B cells ($CD19^+$ cells) and NK cells ($CD16^+CD57^+$ cells). $CD4^+$ and $CD8^+$ T-cell counts were used to derive the immune health grades (IHGs), a metric of immune resilience (IR), as described previously (Ahuja et al., 2023; Lee et al., 2021) and shown in **Figure 6a-d** and Figures S26b, S27. Please see previous report (Lee et al., 2021) regarding clinical and laboratory monitoring, cytomegalovirus (CMV) testing (BioVision CMV IgG Elisa kit, Milpitas, CA), and therapies. Mechanistic studies (flow cytometry, RNA-seq, plasma cytokine analysis) were performed on a subset of the overall cohort (*n*=48; **Tables S5f and g**). Neutralizing antibody assessments (see Section 2.2 below) were performed at baseline and longitudinally in 1,098 patients from the overall cohort.

1.1b. Data collection and sources

Study data were collected and managed using REDCap electronic data capture tools hosted at the University of Texas Health San Antonio, Texas (Lee et al., 2021). REDCap is a secure, web-based software platform designed to support data capture for research studies (Harris et al., 2019; Harris et al., 2009).

Participant data were obtained from routine review of electronic medical records by study investigators and the VHA Corporate Data Warehouse. Demographic characteristics and diagnostic information for complications and post-acute sequalae were provided on the following VHA domains: demographics, vital, inpatient, and outpatient domains. VHA mortality information contains both in-hospital and nonhospital deaths collected from the Veterans Affairs and non-Veterans Affairs sources. Death information was cross-validated with review of the electronic health record. Survival status (right censor) date was defined by last record of use of outpatient/inpatient encounters, prescription fill, or laboratory service within the VHA system.

1.1c. Demographic and clinical characteristics and definitions

Race and ethnicity were recorded in the electronic medical record and were classified as European, African American or Black, Hispanic, Asian/Pacific Islander, and American Indian. Individuals with more than 1 race category were designated as ‘mixed’. 'Other' represents Asian Americans, Native Americans, mixed race/ethnicity, and are not reported herein.

Key VA-CLC features (**Table S5**) were age (median: 62; IQR: 48-72 years); sex (89% [*n*=1,822] male); race/ethnicity (43% [*n*=885] Hispanic American, 34% [*n*=689] European American, and 12% [*n*=236] African American). Sixty-five percent (*n*=1,318) were hospitalized and 9% (*n*=177) died within 30 days. The median interval between the (i) onset of symptoms and patient encounter was 5 (IQR: 1-14) days, (ii) patient encounter and first IR (IHG) measure was 1 (IQR: 0-5) day, and (iii) the median interval between baseline and post-acute measures of IR was 95 (IQR: 59-131) days. In this study, baseline was indexed to the time of the first IR measure.

Nonhospitalized vs. hospitalized, survivors vs. nonsurvivors during ac-COVID-19

Patients who tested positive for SARS-CoV-2 and were managed as outpatients were classified as nonhospitalized (NH). These included those who tested positive in the emergency department and/or in a skilled nursing facility but did not require hospital admission. Patients who tested positive for SARS-CoV-2 and were hospitalized for at least 1 day and survived at least 30 days were considered hospitalized 30-day survivors (H-S). Patients who tested positive for SARS-CoV-2 who died within 30 days were considered nonsurvivors (NS); all nonsurvivors required hospitalization. Note: none of the nonhospitalized patients died within the 30-day window.

Comorbidities

Data on the following 19 comorbidities were collected at baseline: asthma, asplenia, chronic obstructive pulmonary disease (COPD), chronic kidney disease (including end-stage renal disease), liver disease or cirrhosis, interstitial lung disease, diabetes mellitus, congestive heart failure, hypertension, coronary artery disease, connective tissue disease, atrial fibrillation, peripheral vascular disease, cerebrovascular accident, active malignancy, past malignancies, HIV, dementia, and obesity (based on body mass index ≥30 kg/m^2). Baseline patient characteristics stratified by IHG and comorbidity status are detailed in **Table S5a**.

Respiratory status

The severity of acute respiratory distress syndrome (ARDS) was determined according to the Berlin definition, which uses the ratio of arterial oxygen tension to fraction of inspired oxygen (PaO_2/FiO_2) (Force et al., 2012). To capture early ARDS due to COVID-19, the first arterial measurement taken during the encounter was analyzed, with a median of 1 day from baseline IHG. Patients with PaO2/FiO2 > 300 and no requirement for oxygen support were classified as ‘No ARDS’. These ARDS groups were compared to a non-ARDS control group consisting of a subset of the VA-CLC who were clinically deemed to

have no respiratory parameters warranting arterial blood gas monitoring, including those who were not hospitalized.

Ac-COVID-19 complications

Incident acute complications were evaluated within the first 30 days of diagnosis (ICD-10-CM code U07.1). Complications were defined as extra-pulmonary organ-specific diagnoses, including cardiovascular (thromboembolism, heart failure, myocarditis, arrhythmia, cardiomyopathy, myocardial infarction, and cardiac arrest), acute renal failure, acute liver injury, and sepsis/septic shock (multiorgan failure). Severe respiratory failure (ARDS), a characteristic of severe ac-COVID-19, was evaluated separately and not regarded as a complication. In-hospital complications were determined from routine clinical records and cross-validated with documented ICD-10-CM codes during the hospital encounter (see table below). The serious complication score (SCS) is the composite sum of these complications. Characteristics of complications by IHG and outcomes are detailed in **Table S5a**.

Table. Ac-COVID-19 complication ICD-10-CM Codes

Condition	ICD-10-CM Code(s)
Acute hepatic failure	K72
Acute renal failure	N17.X
Acute myocarditis	I40.X
Cardiac arrhythmia	I47-I49.X
Viral cardiomyopathy	B33.24
Viral pericarditis	B33.23
Acute pulmonary embolism	I26.X
Venous embolism and thrombosis	I82.X
Shock	R57.0, R57.1, R57.8, R57.9, R65.21
Sepsis	R65.2X, A41.X

Improvement in IR status during ac-COVID-19

Early improvements in IR were assessed among participants with 2 or more IHG measurements within 5 days after baseline (**Table S10**). Improvement in IR status was designated by a ≥+1-point difference according to the following IHG hierarchy point scale: IHG-I (8), IHG-III (7), IHG-IIa (6), IHG-IVa (5), IHG-IIb (4), IHG-IVb (3), IHG-IIc (2), and

IHG-IVc (1). For those with baseline IHG-I, improvement was considered if subsequent measurements were also IHG-I.

1.1d. VA-CLC post-acute lifespan subset

Post-acute outcomes were evaluated in a subset of VA-CLC participants who were still alive 120 days after presentation with ac-COVID-19 (*n*=1,738) and followed to death or until October 31, 2022 (median follow-up 591 days, IQR: 447-976). This group was designated the post-acute lifespan subset of the overall VA-CLC. The 120-day period to define the start of the post-acute lifespan window was specified to conservatively distinguish from ac-COVID-19 outcomes by 90 days. Characteristics of the VA-CLC post-acute subset are detailed in **Table S5b**. Covariates examined included age, sex, preexisting comorbidity burden, baseline and post-acute standard-of-care biomarkers (neutrophils, IL-6, CRP, B-cells, and NK cells [D-dimer and ferritin were not monitored in the post-acute lifespan subset]).

The primary outcome in the post-acute lifespan subset was 2-year mortality from an index date of 120 days following presentation with ac-COVID-19. The secondary outcome was sequelae of COVID-19. We examined the ICD-10-CM diagnosis code for ‘post COVID-19 condition, unspecified’ (U09.9) after at least 30 days from index COVID-19 diagnosis (ICD-10-CM code U07.1). Characteristics of those with and without a diagnosis of post-acute condition are detailed in **Table S5e**.

1.1e. VA-CLC with available pre-COVID-19 IHG data

Approximately 27 participants had IHG measurements across 3 timepoints (prior to index COVID-19, baseline IHG during ac-COVID-19, and post-acute). Characteristics of these participants are detailed in **Table S5d** and **Figure S27c**.

1.2. STVHCS acute COVID-19 outbreak cohort

The acute COVID-19 outbreak cohort comprised veterans residing at a state Veterans Affairs skilled nursing facility who tested positive for SARS-CoV-2 (*n*=77) during two outbreak windows (March 2020, *n*=14 and January 2021, *n*=63). Sequential daily testing

of all residents and staff was performed until containment. Most (98%) were males with a median age of 78 (73-88) years; 42% required hospitalization, and 16% did not survive past 30 days of infection (**Table S5h**). IHG and SARS-CoV-2 neutralizing antibody (nAb) levels (estimated through percent inhibition by the surrogate virus neutralization test (sVNT) (Jeewandara et al., 2021; C. W. Tan et al., 2020; Taylor et al., 2021)) were measured longitudinally (170 samples) through 400 days from the time of the positive test.

1.3. San Antonio Immunologic Resilience Longitudinal (SAIL) cohort: pre-booster vaccine cohort of persons without acute COVID-19

The SAIL pre-booster vaccine cohort is an ongoing prospective cohort developed at the Audie L. Murphy VA Medical Center, STVHCS, San Antonio, Texas. The cohort includes healthcare workers, veterans, and an otherwise healthy general population aged 18 years and older from within San Antonio, Texas, and surrounding counties. The cohort includes 1319 participants with IHG data (IHG distributions in **Supplementary Note**). IHGs were determined using $CD4^+$ and $CD8^+$ T-cell counts obtained using standard procedures at LabCorp (CD4:CD8 Ratio Profile, LOINC 505271). A subset of 760 participants who completed their primary COVID-19 vaccine series (received 2 doses of either Pfizer/BioNtech or Moderna or a single dose of J&J/Jansen COVID-19 vaccine) but had not received additional doses ("pre-booster") and were considered as part of the pre-booster subset. Demographic comorbidities, and vaccine and infection history were collected.

Key SAIL cohort features were age (median: 53, IQR: 43-63 years), sex (56% [*n*=740] male), and race/ethnicity (36% [*n*=473] Hispanic American, 34% [*n*=444] European American, and 10% [*n*=138] African American).

1.4. Non-acute: VA-convalescence program subset

Patients diagnosed with COVID-19 who were deemed post-acute and discharged from the hospital or outpatient program (home telehealth program) at the STVHCS South Texas Veterans Health Care System were enrolled in the VA convalescence monthly

interdisciplinary monitoring program, which aimed to identify and address post-acute effects. The program involved monthly virtual clinic visits and referrals to specialized healthcare professionals. Entry into the program typically occurred around one month after the initial diagnosis of post-acute COVID-19. Throughout the program, immune profiles and standard-of-care biomarkers, such as $CD4^+$ and $CD8^+$ T-cells, B-cells, NK cells, complete blood cell count, IL-6, and CRP, were assessed on a monthly basis. A total of 2,419 immune profile samples from 1,418 unique patients were analyzed from May 2020 through September 2023, with 45% (*n*=643) of these samples originating from patients in the VA-CLC cohort.

1.5. Reference populations for IR assignments of IHGs

We used several reference populations to define the distribution of IHGs vs. $CD4^+$ counts and/or CD8:CD4 ratios and assign IR status (**tables in Supplementary Note**). Cohort characteristics have been described by us previously (Ahuja et al., 2023; Okulicz et al., 2015). To control for the extensive inter-individual and inter-cohort (condition) variability in $CD4^+$ and $CD8^+$ count, we derived IHGs to serve as a uniform metric that describes the relative balance between $CD8^+$ and $CD4^+$ T-cell counts, without regard to age, sex, or disease/conditions. We benchmarked IHG distributions in an ac-COVID-19 population versus these non-ac-COVID-19 cohorts, accounting for age and sex, as well as severity of and recovery from ac-COVID-19. This benchmarking revealed that IHG distribution, degradation, and reconstitution patterns were non-stochastic (Ahuja et al., 2023; Le et al., 2013; Lee et al., 2021; Okulicz et al., 2015). A brief overview of these reference cohorts is presented in **table below**.

Reference Cohort	*n*	Median Age (IQR)	Description	Most prevalent IHGs
1. SardiNIA	3898	49 (36-62)	Healthy aging cohort	IHG-I
2. HIV− UCSD	759	39 (30-48)	Healthy cohort	IHG-I
3. HIV− Majengo Sex Worker cohort	449	31 (27-37)	Highly-exposed to HIV	IHG-I
4. HIV+ PIC UCSD	592	33 (26-40)	Therapy-naïve acute	IHG-IV
5. US Military HIV Natural History Study				
Cohort entry	1110	29 (24-35)	HIV+	IHG-IV
Pre-ART	1110	31 (26-37)	Therapy-naïve HIV+	IHG-IV
During ART	1110	34 (29-40)	Aviremic HIV+	IHG-IV and IHG-III

1.5a. SardiNIA

<u>Cohort description:</u> The SardiNIA study investigates genotypic and phenotypic aging-related traits in a longitudinal manner. The main features of this project have been described in detail previously (Orru et al., 2013; Pilia et al., 2006; Pistis et al., 2015). All residents from 4 towns (Lanusei, Arzana, Ilbono, and Elini) in a valley in Sardinia (Italy) were invited to participate. Since November 2001, a total of 6,921 participants were recruited and phenotyped, male and female, age 14 years and older. This corresponds to approximately >60% of the population eligible for recruitment in the area. The SardiNIA participants studied (Orru et al., 2013) comprised 3,898 individuals (age 18 – 102 years) in whom immune marker sets were available and were included in the analyses. Age ranges were from 15 to 103 years, and 57% were females. The median $CD8^+$ and $CD4^+$ T-cell counts and median CD4:CD8 ratio of these individuals are reported in the **Supplementary Note**.

1.5b. HIV− cohort from the University of California San Diego (termed HIV− UCSD cohort)

<u>Cohort description:</u> The HIV-seronegative UCSD cohort was derived from the following three resources: (a) those who enrolled as a normative population for ongoing studies funded by the National Institute of Mental Health; (b) those who enrolled as a normative population for studies funded by the National Institute on Drug Abuse; (c) those who enrolled as HIV– users of recreational drugs for studies funded by the National Institute on Drug Abuse. In the present study, we evaluated 759 participants, pooled from the 3 abovementioned sources, as described previously (Ahuja et al., 2023; Okulicz et al., 2015). The median (IQR) age of these 759 participants was 39 (30-48) years. The corresponding values according to IHG grades are in **Supplementary Note tables**.

1.5c. The Kenya Majengo Observational Cohort Study cohort (MOCS)

<u>Cohort description:</u> The Majengo female sex worker (FSW) cohort (Bandewar et al., 2010) is an open cohort dedicated to better understanding the natural history of HIV infection, including defining immunologic correlates of HIV acquisition and disease progression. The MOCS cohort was established in 1985 in the Majengo area of Nairobi, Kenya.

Biannually (3- to 6-month intervals), all FSWs participated in a resurvey where repeat HIV/STI testing and $CD4^+$ and $CD8^+$ T-cell counts were assessed. The association of risk behavior (e.g., duration of sex work, frequency of condom use, clients per week) with the prevalence of CD8-CD4 disequilibrium grades IHG-III and IHG-IV, as well as future HIV seroconversion, were evaluated in these 762 FSWs and are reported elsewhere (Ahuja et al., 2023). To mitigate confounding, we show data for the 449 women (Figure S26b) who met the following criteria: had concurrent $CD4^+$ T-cell count and CD4:CD8 ratio measurements, as well as risk behavior data and at least 2 HIV seronegative follow-up visits 3 months apart (Ahuja et al., 2023).

1.5d. US Military HIV+ Natural History Study (NHS) Cohort

Cohort description: The US Military HIV Natural History Study is an ongoing, continuous-enrollment, prospective, multicenter, observational cohort study conducted through the Uniformed Services University of the Health Sciences Infectious Disease Clinical Research Program. The NHS has enrolled about 5,723 active-duty military service members and beneficiaries since 1986 at 7 military treatment facilities (MTFs) throughout the US. The US military medical system provides comprehensive HIV education, care, and treatment, including the provision of antiretroviral therapy (ART) and regular visits with clinicians with expertise in HIV medicine at MTFs, at no cost to the patient. Mandatory periodic HIV screening according to Department of Defense policy allowed treatment initiation to be considered at an early stage of infection before it was recommended practice. Eighty-eight percent of the participants since 1995 have documented seroconversion (i.e., a documented negative HIV test preceding a positive HIV test), with a median seroconversion window of approximately 15 months. The median $CD4^+$ T-cell count at diagnosis was approximately 500 cells/mm^3. A total of 1,110 participants with estimated dates of seroconversion who initiated ART between 1/3/1996 and 8/8/2012 (with clinical data collected until 4/7/2014) and met other inclusion criteria as described previously (Krantz et al., 2011; Marconi et al., 2011; Marconi et al., 2010; Okulicz et al., 2015; Okulicz et al., 2009) were evaluated. A subset of the NHS cohort was used for evaluation of correlation with functional immune markers (%$CD3^+$pSTAT5$^+$ T-cells after IL-7 stimulation as a biomarker of T-cell responsiveness and %PD-1$^+$CD4$^+$ T-cells as a

biomarker of T-cell dysfunction) as detailed in Ahuja et al. (Ahuja et al., 2023; Camargo et al., 2009).

1.6. Primary Infection HIV+ cohort from the University of California San Diego (termed HIV+ PIC cohort)

The PIC cohort comprised 723 HIV+ participants (Le et al., 2013). These participants were recruited between June 1996 and June 2010 and then followed prospectively. Details of the cohort were as described previously (Le et al., 2013). We evaluated only those participants in whom an estimated date of infection could be calculated through a series of well-defined stepwise rules that characterize stages of infection based on our previously described serologic and virologic criteria. Of the 723 participants, 685 were evaluated in the present study while they were therapy-naïve.

2. Sample processing methods

2.1. Peripheral blood mononuclear cell (PBMC) isolation

PBMC isolation from EDTA-treated whole blood was performed using Ficoll-paque PLUS (Cytiva) according to standard protocols. After PBMC isolation, PBMCs were resuspended in freezing media (90% fetal bovine serum + 10% DMSO) and cryopreserved in liquid nitrogen for further use.

2.2. Surrogate virus neutralization test (sVNT) methods and evaluations

The SARS-CoV-2 sVNT is based on a serological test that mimics the virus-host interactions at the protein-to-protein level (C. W. Tan et al., 2020). This test is a competitive assay that quantifies the presence of antibodies that target the receptor-binding domain (RBD) of the viral spike (S) protein of SARS2-CoV-2 and disruption of the RBD binding to the hACE2 receptor. This test is highly correlated with the cellular VNT (r^2=0.8591) and the pseudotyped VNT (r^2=0.8374) (C. W. Tan et al., 2020). The sVNT was performed using the SARS-CoV-2 sVNT kit (GenScript) in plasma samples according to the manufacturer's protocol. In its qualitative form, the test provides results in percent inhibition (%inhibition). Inhibition ≥30% represents the detection of nAb against SARS-CoV-2 and inhibition <30% is considered absence of nAb. In order to establish quantitative levels of nAb in plasma (U/mL), a standard curve (adjacent figure) was generated within the sVNT assay using 559 samples (from 145 unique individuals) and the neutralizing antibody standard (GenScript A02087) following the manufacturer's protocol. The quantitative levels (% inhibition) were then used to determine its correlation with nAb concentration as depicted above.

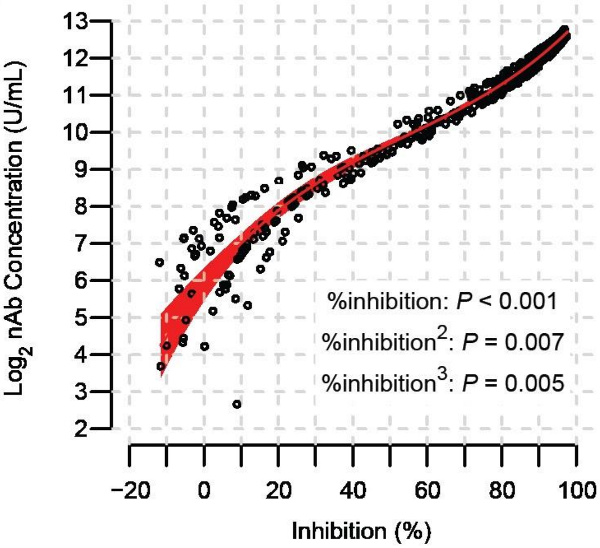

2.3. SARS-CoV-2 Viral Load

Viral load (VL) was gauged by the cycle threshold (Ct) values derived from COVID-19 RT-qPCR testing in nasopharyngeal swabs using as a biologically relevant proxy for VL (Waudby-West et al., 2021). Ct values for N2 genes were analyzed. A Ct value of < 30 was used as a threshold to indicate higher viral load. Ct values from samples within ±7 days from baseline (first IHG measurement) were included in the analysis; the time (days) between Ct date and baseline date were adjusted for in models evaluating VL.

2.4. Flow cytometry

Antibodies used for the T, B, and innate cell panels are described in the **table below**. For each panel, antibodies were used at optimal concentrations according to titrations obtained during panel optimization. Cryopreserved PBMCs were thawed and washed 2X in 10mL of warm complete RPMI 1640 (10% FBS + 1X Glutamax + 1X pen/strep) containing 10U/mL benzonase (Millipore-Sigma) and cells were rested overnight. The next day, cells were collected and clumps removed with a 70-µm strainer. Then cells were washed 2X in PBS and counted. Cells were stained with the BD horizon viability stain 780 (BD Biosciences) according to the manufacturer's directions. After washing cells with staining buffer (0.5% BSA/PBS + 0.08% NaN_3), cell pellet was treated with Human Fc blocking (BD Biosciences) and incubated at 4ºC for 15min. A master mix for each panel was made and added to each corresponding tube and incubated at 4ºC for 15min. PBMCs were washed in staining buffer (SB) and resuspended in 250µL of SB for acquisition. Cells were acquired with an LSR-Fortessa (BD Biosciences), and data were analyzed with FlowJo v10 (BD Biosciences). To assess and maintain data reproducibility, an electronic check of the flow cytometer by applying the CS&T System (BD Biosciences) was performed daily. Fluorescent minus one (FMO) controls were applied to each panel to determine positivity boundaries. Compensation controls for each panel were used with every run using UltraComp eBeads for antibodies (ThermoFisher) and ArC beads (ThermoFisher) for the viability dye, following the manufacturer's directions.

Panel	Antibodies (Clone)	Manufacturer
T cells	CD3 BUV395 (UCHT1)	BD Biosciences
	CD4 APC-R700 (RPA-T4)	BD Biosciences
	CD8 V500 (SK1)	BD Biosciences
	CD25 PE (MA-A251)	BD Biosciences
	CD28 BUV737 (CD28.2)	BD Biosciences
	CD38 BV421 (HIT2)	BD Biosciences
	CD39 APC (TU66)	BD Biosciences
	CD45RA FITC (HI100)	BD Biosciences
	CD95 PE-CF594 (DX2)	BD Biosciences
	CD127 PE-Cy7 (HIL-7R-M21)	BD Biosciences
	CD197/CCR7 PerCP-Cy5 (150503)	BD Biosciences
	CD279/PD1 BV650	Biolegend
	HLA-DR BV711 (L243)	Biolegend

Innate	CD3 BUV395 (UCHT1)	BD Biosciences
	CD8 V500 (SK1)	BD Biosciences
	CD11c PE (B-Ly6)	BD Biosciences
	CD14 BB700 (MoP9)	BD Biosciences
	CD16 FITC (3G8)	BD Biosciences
	CD19 APC-R700 (HIB19)	BD Biosciences
	CD56 BV650 (NCAM16.2)	BD Biosciences
	CD62L BV421 (DREG-56)	Biolegend
	CD123 PE-Cy7 (7G3)	BD Biosciences
	CD141 BV786 (1A4)	BD Biosciences
	HLA-DR BUV737 (G46-6)	BD Biosciences
	MAIT/TCR-Va7.2 APC (3C10)	Biolegend
	iNKT/TCR-Va24-Ja18 PE (6B11)	Biolegend
B cells	CD5 PE (UCTH2)	Biolegend
	CD10 PE-CF594 (HI10a)	BD Biosciences
	CD19 BUV737 (SJ25C1)	BD Biosciences
	CD20 BUV395 (2H7)	BD Biosciences
	CD21 BV510 (1048)	BD Biosciences
	CD27 APC-R700 (M-T271)	BD Biosciences
	CD38 PE-Cy7 (HB7)	Biolegend
	CD95 BV421 (DX2)	BD Biosciences
	IgM BV711 (G20-127)	BD Biosciences
	IgG BB515 (G18-145)	BD Biosciences
	IgD BV785 (IA6-2)	Biolegend
	IgA APC (IS11-8E10)	Miltenyi Biotec

2.5. Transcriptomic profiling

Methods for transcriptomic profiling by RNA-Seq are as described previously (Ahuja et al., 2023; Lee et al., 2021). Briefly, RNA was isolated from peripheral blood samples collected from 330 samples from 48 COVID-19 patients, a subset of the VA-CLC. Characteristics of this subset are detailed in **Tables S5f and g**. RNA isolation, sequencing, and alignment were performed as described previously (Lee et al., 2021; Smith et al., 2021). Methods and bioinformatics analysis were similar to those reported previously (Lee et al., 2021; Smith et al., 2021) and are summarized below.

Normalization and quality control were performed as previously described (Lee et al., 2021). Briefly, genes with a gene count of 0 across all samples were removed; the remaining 0's were changed to 1's. Using the R package DESeq v1.39.0 (Anders & Huber, 2010), the size factors were estimated using the gene-expression matrix taking library sizes into account; these size factors were used to normalize the gene counts. Genes with expression levels <25% of total expression from all samples were removed. Gene signature scores were calculated as described in Section 4.

3. Data download and normalization

All download, normalization, and score analysis was performed in R statistical environment, as described previously (Ahuja et al., 2023). The following packages were used: GEOquery, ArrayExpress, Biobase, limma, preprocessCore, stringr, geepack, corrplot, gplots, DESeq, reshape, downloader, lmtest, forestplot, broom, and others. A modified version of GEO2R script sourced from NCBI GEO was used for GEO datasets and custom script based on ArrayExpress R package was used for ArrayExpress datasets. These scripts were used to download and normalize the data.

3.1. Microarray datasets

For microarray datasets, whenever possible, log_2-transformed and quantile normalization was performed before computing transcriptomic signature scores. If both normalizations were not feasible, either log_2-transformed (if the data were already quantile normalized) or quantile (if the data were already log_2-transformed normalized or >5% of the data had negative values) normalization was performed. For datasets having values provided as log_2 ratios of samples, scores for signatures were computed after quantile normalization.

3.2. RNA-seq datasets

Gene counts were normalized, and dispersion values were estimated using the R package DESeq v1.39.0 (Anders & Huber, 2010). The design matrix (row – samples; column – experimental variables) used in DESeq, along with gene-expression matrix (row – genes; column – gene counts in each sample), and the personal identification number where available.

Genes with a gene count of 0 across all samples were removed; the remaining 0s were changed to 1s and these genes were used in the gene-expression matrix in DESeq. The size factors were estimated using the gene-expression matrix taking library sizes into account; these were used to normalize the gene counts. Genes with expression levels <25% of total expression from all samples were removed. Note: the filtered genes are expressed at low levels across all samples and would not be differentially expressed

(FDR<0.05) in comparisons. The dispersion factors were estimated using the options: method=blind and sharingMode=fit-only, as there were too many variables (due to personal identification numbers) to use the default. Cross-sectional differences between the groups were assessed.

3.3. Probe-Gene information

For microarray datasets, the gene symbol information for probes were determined from the feature data associated with platforms on which the gene expression data were generated. In cases where gene symbol information was not available as part of feature data, they were manually annotated using standard resources: HUGO Gene Nomenclature Committee (HGNC) at the European Bioinformatics Institute (https://www.genenames.org).

In instances where there were multiple probes or transcripts representing a single gene, mean values were determined and used for computing the scores for signatures.

3.4. Single-cell RNA-seq (scRNA-seq)

The standard workflow in Seurat (Butler et al., 2018; Stuart et al., 2019) was utilized for scRNA-seq analyses (Figure 2a and Figure S3). Processing and clustering were performed based on parameters listed to match the clusters presented in the original publications (Table S15b)**.** Example for Figure 2a, we downloaded pre-processed and clustered scRNA-seq dataset of PBMCs ~10k cells using the standard workflow in Seurat, provided at Satija Lab webpage [https://www.dropbox.com/s/3f3p5nxrn5b3y4y/pbmc_10k_v3.rds?dl=1]. The processed dataset consisted of 9,432 cells and 19,089 genes and clustered into 13 different clusters of immune subsets based on gene expression. We calculated the expression of gene signature scores (as explained in Section 4) for each of the cells and overlaid the expression of these scores on clusters by color coding them from blue (low) to red (high) for aggregation of scores.

3.5. Quality control of the dataset and interpretation

We stress that expression of gene signatures is a relative term within a dataset, and it is challenging to compare the score across different datasets.

We are mindful that different RNA microarray or RNA-seq platforms have differences in the availability of gene probes corresponding to the genes in a given gene signature. This could affect the interpretation of the findings. Where applicable, the percent of available genes per signature within a dataset is shown in figures depicting gene signatures. In addition, we stress that transcriptomic signature scores were defined in relative terms and caution is needed for cross-dataset comparisons.

4. Transcriptomic signatures and analyses

Details and derivations of gene expression signatures are outlined below and in Tables S1-S2.

4.1. SAS and MAS

As previously described (Lee et al., 2021), the expression levels of 52 GO-BP (gene ontology biological process) terms associated with categorical COVID-19 severity outcomes (FDR<0.05; i.e., hospitalization vs. nonhospitalization, survivors vs. nonsurvivors) are reported as gene signature expression levels (z-scores) of genes representing the aggregate expression of genes in a specific GO-BP term. To generate the z-score, the log_2-normalized expression of each gene is z-transformed (mean centered then divided by standard deviation) across all samples and then averaged. The grouping of the 52 signatures into 4 categories is depicted below.

After adjusting for age and sex and correcting for multiple comparisons (FDR), 29 signatures and 16 signatures out of the 52 signatures significantly associated (FDR<0.05) with hazard of mortality in the ac-COVID-19 cohort and Framingham Heart Study (FHS), respectively (**Figure below**: Category 1). Ten signatures overlapped between both cohorts and were further examined. Of these 10 signatures, the three signatures that associated (independently of age/sex) with lower and the seven signatures that

associated with higher mortality hazards in both cohorts were termed as Survival-Associated Signature (SAS) and Mortality-Associated Signature (MAS), respectively. As described previously (Ahuja et al., 2023), SASs and MASs were numbered according to their prognostic capacity for predicting survival or mortality, respectively in the FHS [lowest to highest Akaike information criteria (AIC); SAS-1 to SAS-3 and MAS-1 to MAS-7]. The top associated signatures in each category were SAS-1 (GO-BP term: immune response) and MAS-1 (GO-BP term: defense response to gram-positive bacterium) and were used in this study as z-scores to metricize IR status.

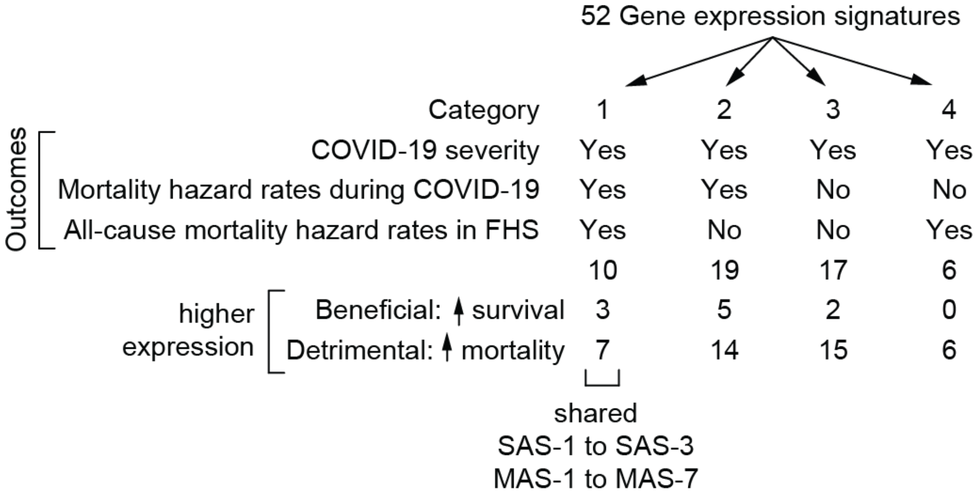

The genes in these signatures are listed below.

SAS-1 genes (*n*=21)			
CCL4L2	*CXCR5*	*ICOS*	*TCF7*
CCR4	*ETS1*	*IL24*	*TNFRSF25*
CCR7	*GPR183*	*IL7R*	*VPREB3*
CD27	*HLA-DQA1*	*MS4A2*	
CD40LG	*HLA-DRB1*	*PTGDR2*	
CXCL8	*HLA-DRB5*	*SUSD2*	

MAS-1 genes (*n*=22)			
ADAM17	*DEFA3*	*HIST1H2BF*	*RNASE3*
ADM	*DEFA4*	*HIST1H2BG*	*TBK1*
ANG	*DEFB1*	*HIST1H2BK*	*TLR2*
C5AR1	*HAVCR2*	*HIST2H2BE*	*TNFSF8*
CAMP	*HIST1H2BC*	*HMGB2*	
CD36	*HIST1H2BD*	*MYD88*	

To generate the z-scores, the normalized expression of each gene is z-transformed (mean centered then divided by standard deviation) across all samples and then

averaged. Categorical score bins (high/low) of SAS-1 and MAS-1 were determined using the calculated median score values relative to each cohort.

4.1a. SAS/MAS profiles

Four theoretical SAS-1/MAS-1 profiles are possible by combining the high/low expression of SAS-1 and MAS-1 scores based on median values in the entire dataset (which is dataset specific) (**table below**). High indicates expression of the score in the sample greater than the median expression of the score in the dataset, whereas low indicates expression of the score in the sample less than or equal to the median expression of the score in the dataset.

SAS-1	MAS-1	SAS-1/MAS-1 profiles
Low	Low	$SAS\text{-}1^{Lo}\text{-}MAS\text{-}1^{Lo}$
Low	High	$SAS\text{-}1^{Lo}\text{-}MAS\text{-}1^{Hi}$
High	Low	$SAS\text{-}1^{Hi}\text{-}MAS\text{-}1^{Lo}$
High	High	$SAS\text{-}1^{Hi}\text{-}MAS\text{-}1^{Hi}$

4.2. Methods for IMM-AGE transcriptomic signature score

A list of 57 genes reported by Alpert et al. (Alpert et al., 2019) as immune-aging transcriptomic signature (IMM-AGE) was used to derive this signature. The genes significantly and consistently correlated with both the age and cell-based IMM-AGE score that predicted all-cause mortality in the FHS offspring cohort (Alpert et al., 2019). Note: The directionality of association of IMM-AGE (transcriptomic-based) with mortality reported by us (higher IMM-AGE score associated with lower mortality) is opposite to the association of IMM-AGE (cell-based) with mortality reported by Alpert et al. (Alpert et al., 2019), as all 57 genes used in IMM-AGE (transcriptomic-based; reported by us in this study) are inversely correlated with the IMM-AGE (cell-based) score they derived. The IMM-AGE transcriptomic signature score was examined in different datasets to assess its association with survival. To generate the z-score, the $\log_2$ normalized expression of each gene is z-transformed (mean centered then divided by standard deviation) across all samples and then averaged.

4.3. Extreme Longevity (EL) signatures

Signatures derived by Karagiannis et al. (Karagiannis et al., 2023) in single cell RNA-seq (scRNA-seq) of PBMC samples from a cohort of centenarians (extreme longevity [EL]; ages 100-119 y) and young adults (ages 20-59 y). Major lymphocyte and myeloid cell types were identified, and cell proportions were analyzed between the age groups. Transcriptomic analysis between age groups was conducted in aggregate over different cell types, yielding 136 over-expressed and under-expressed genes in the EL cohort vs young adults. Genes provided in Karagiannis et al. [their Supplementary Table S29].

- **EL^{up}**
    - Signature of 136 genes associated with inflammation with higher expression in EL vs. younger adults.
- **EL^{down}**
    - Signature of 251 genes associated with immune responses and B cell activation [GO-BP terms using DAVID 2021 (Huang da et al., 2009; Sherman et al., 2022)], with lower expression in EL vs. younger adults.

4.4. SenMayo signature

A signature was derived by Saul et al. (Saul et al., 2022) through gene set enrichment analysis (GSEA) of senescence-associated genes and previous studies of senescence and/or SASP-secreting cells (verified in human or mouse cells). GSEA was performed in RNA-seq analyses of bone/marrow biopsies and highly enriched osteocyte fractions in two cohorts of young (25-35 years) and elderly (63-79 years) individuals. The *n*=125 gene signature was validated in multiple tissues across species, demonstrated clearance of senescent cells in adipose tissue post-DQ treatment (Dasatinib + Quercetin), identified senescent cells in scRNA-seq datasets, and was further validated in silico. The detrimental signature comprised canonical senescence factors (*CCL24, SEMA3F, FGF2, IGFBP7*) and pro-inflammatory cytokines/chemokines. Genes are provided in Saul et al. [their Supplementary Data 1 (Saul et al., 2022)].

4.5. Age_IL6 signatures

Signatures were derived by Pilling et al. (Pilling et al., 2015) in blood samples from the FHS Offspring and InCHIANTI cohorts. Mediation analysis was performed using IL-6 as a dependent measure and chronological age as the independent variable, where gene transcripts were identified as significantly mediating a proportion of the association of IL-6 and age. There were 102 of 17,324 genes that met the significance threshold (P<0.05 Bonferroni-adjusted) after adjusting for sex, technical covariates (microarray batch, study site), and cell type proportions including lymphocytes, monocytes, eosinophils, and basophils. Genes are provided in Pilling et al. [their Table 2 (Pilling et al., 2015)].

- **Age_IL6^{up}**
  - Detrimental signature of positive-mediating genes (n=45) associated with higher IL-6 levels and age.
- **Age_IL6^{down}**
  - Beneficial signature of negative-mediating genes (n=57) associated with lower IL-6 levels and age.

4.6. InflammΔage signatures

Signatures were derived by Lin et al. (Lin et al., 2017) in blood samples from the FHS Offspring cohort. Chronological age was contrasted with biologic age measures (assay of inflammatory biomarkers CRP, ICAM1, IL6, LP-PLA2 mass and activity, MCP1, osteoprotegrin, p-selectin, and TNFR2) to calculate inflammatoryΔage. Using linear mixed-effect models adjusting for sex, age, imputed cell counts, and technical covariates, 448 genes were significantly associated with inflammatoryΔage. The top 25 genes by P values were provided by Lin et al. [their Table 2 (Lin et al., 2017)]

- **InflammΔage^{up}**
  - Detrimental signature of 19 genes that significantly associated with greater biologic age than chronological age.
- **InflammΔage^{down}**
  - Beneficial signature of 6 genes that significantly associated with lesser biologic age than chronological age.

4.7. Tri-partite T-cell health (t-TCH) signatures

Initial gene sets were derived by Kennedy et al. (Kennedy et al., 2016) and further refined in Ahuja et al. (Ahuja et al., 2023) to derive a signature linking senescent cells associated with immune aging (CD8+CD28– T-cells) and functional immune attributes of T-cell responsiveness and dysfunction. The cohort established by Kennedy et al. comprised 159 older persons receiving 2010-2011 trivalent inactivated influenza vaccine (TIV) vaccines, measuring markers of immunosenescence including CD8+CD28– T-cells. Correlation between baseline gene expression and immunosenescence markers yielded 386 genes associated with %CD8+CD28–.

This gene set was further evaluated by Ahuja et al (Ahuja et al., 2023). in RNA-seq profiling of PBMC samples from the subset of the NHS HIV+ cohort that significantly associated with %$CD3^{+}pSTAT5^{+}$ T-cells after IL-7 stimulation as a biomarker of T-cell responsiveness and %$PD\text{-}1^{+}CD4^{+}$ T-cells as a biomarker of T-cell dysfunction, as described previously (Camargo et al., 2009). The association of these three biomarkers ($CD8^{+}CD28^{-}$ senescent cells, T-cell dysfunction, and T-cell responsiveness) contributed to the derivation of the tripartite T-cell health signature.

- **t-TCH^{high}**
  - Beneficial signature of 20 genes that significantly associated (FDR<0.05) with lower T-cell dysfunction, lower $CD8^{+}CD28^{-}$ senescent cells, and higher T-cell responsiveness in the subset of NHS cohort and the TIV cohort.
- **t-TCH^{low}**
  - Detrimental signature of 12 genes that significantly associated (FDR<0.05) with higher T-cell dysfunction, higher $CD8^{+}CD28^{-}$ senescent cells, and lower T-cell responsiveness in the subset of NHS cohort and the TIV cohort.

4.8. Blood transcription modules

Blood transcription modules data reported by Li et al. (Li et al., 2014) were downloaded and used for this analysis. The following modules were selected for this study for their reported association with vaccine responses by Arunachalam et al. (Arunachalam et al., 2021). The genes in these modules are listed in **Table S2**.

- **M7.1, T cell activation (I)**
  - This module associated with BNT162b2 COVID-19 vaccine response and comprised 50 genes.
- **M11.0, enriched in monocytes (II)**
  - This module associated with both neutralizing antibody response and CD8 IFNγ T-cell response in BNT162b2 COVID-19 vaccine participants and comprised 173 genes.
- **M16, TLR, and inflammatory signaling**
  - This module associated with neutralizing antibody response but not CD8 IFNγ T-cell response in BNT162b2 COVID-19 vaccine participants and comprised 45 genes.
- **M156.0, plasma cells & B cells, immunoglobulins**
  - This module was consistently upregulated with most vaccines except BNT162b2 COVID-19 vaccine and comprised 43 genes.

4.9. Interferon-stimulated genes (ISG) signatures

- **ISG_33**
  - 33-gene signature developed by Arunachalam et al. (Arunachalam et al., 2020) comparing CITE-seq data of dendritic cell-enriched PBMC samples from COVID-19 patients and age-matched healthy controls. Higher expression in COVID-19 vs control subjects and positively correlated with plasma IFNa.
- **ISG_23**
  - 23-gene signature developed by McClain et al. (McClain et al., 2021) comprised interferon-stimulated genes that discriminates COVID-19, influenza, seasonal coronavirus, bacterial pneumonia, and healthy controls in longitudinal whole blood transcriptomes.

4.10 Gene expression datasets

The summary of datasets studied is presented in **Table S15b.**

Briefly, multiple aging cohorts were leveraged to assess distributions of IR status and their associations, as summated in Figure 2c. Aging cohorts were Framingham Heart Study (FHS; *n*=2308); Mexican-American Family Heart Study (E-TABM-305; *n*=1240), Brisbane cohort (GSE53195; *n*=858); Vitality 90+ cohort (GSE65218; *n*=151); SardiNIA cohort (*n*=3898), University of California San Diego HIV negative controls (*n*=759); and San Antonio Immunologic Resilience Longitudinal (SAIL) cohort (*n*=1319). Brief descriptions are described below.

4.10.1 Offspring cohort of the FHS

The FHS is a longitudinal study to identify contributions to cardiovascular disease (CVD) and other chronic diseases of community-dwelling residents from Framingham, MA (Dawber et al., 1951; Kannel et al., 1979). The original FHS cohort was recruited in 1948 as the first generation and followed with biennial physical examinations and lifestyle interviews. The second-generation cohort (Offspring) began enrolling in 1971 and comprised the first-generation participants' adult children with their spouses. The SABRe CVD Initiative performed multi-omic analyses of FHS participants to generate biomarker data to advance personalized medicine. The FHS cohorts were examined every 4 years when participants undergo a detailed cardiovascular-focused medical history, a brief physical examination (standardized anthropometry, electrocardiography, and blood pressure measurements), and laboratory testing for CVD risk factors. All FHS participants provided written informed consent to participate in the core study protocols that were reviewed by the institutional review board of the Boston University Medical Center, Boston, MA.

In the present study, the gene expression profiling of whole blood collected at the FHS Offspring study Exam 8 conducted by the SABRe CVD Initiative in their Project 3 were analyzed. Microarray expression profiles of 2,306 participants of the FHS Offspring study were paired with clinical exam measurements, laboratory diagnostics, and lifestyle evaluations. Data were accessed through dbGaP study accessions phs000007.v30.p11 and phs000363.v17.p11. Information on the cohort phenotypic data is available through https://www.framinghamheartstudy.org/phenotypic-data/vr_soe_2020_a_1340s/.

Endpoints:

- *All-cause mortality*: Genomic signatures, genes, and IR metrics (SAS-1/MAS-1) were evaluated for all-cause mortality. Follow-up time onwards from Exam 8 was calculated for survival (median: 8.163 years, IQR: 7.483 – 8.841, min: 0.178, max: 9.815).
- *Incident CVD*: CVD as a composite diagnosis was defined by coronary heart disease (CHD; myocardial infarction, coronary insufficiency, angina pectoris, sudden death from CHD, non-sudden death from CHD), intermittent claudication, congestive heart failure, or stroke or transient ischemic attack in the absence of a previous manifestation of any of these diseases. A person having more than one cardiovascular manifestation within the follow-up period was counted as an incident case only at the time of the first event. Pre-existing CHD at Exam 1 is identified by one of the following: definite angina pectoris, definite history of myocardial infarction or ECG, doubtful myocardial infarction by ECG, definite coronary insufficiency by ECG and history. Persons with pre-existing CHD at Exam 1 are excluded from the population at risk of developing CHD.

FHS dbGaP Acknowledgment Statement: The FHS is conducted and supported by the National Heart, Lung, and Blood Institute (NHLBI) in collaboration with Boston University (Contract No. N01-HC-25195 and HHSN268201500001I). This manuscript was not prepared in collaboration with investigators of the FHS and does not necessarily reflect the opinions or views of the FHS, Boston University, or the NHLBI. Additional funding for SABRe was provided by the Division of Intramural Research, NHLBI, and Center for Population Studies, NHLBI.

<u>4.10.2 Other aging cohorts</u>

4.10.2.1 San Antonio Family Heart Study (SAFHS) (E-TABM-305; Figure 3c, main)

Transcription profiling of human lymphocytes from 1240 Mexican Americans (E-TABM-305). Cohort/sample description. To date, most human studies have compared gene expression between age classes of participants (e.g., young vs. old),

but such categorical comparisons do not reveal the potential trajectories of changes that may occur during aging. Moreover, while many genes are expected to show changes in expression with age, a substantial proportion of the individual variation in aging may result from genotype × age interaction (G × AI) effects on a smaller number of genes. We sought to understand the expression levels across age of gene signatures that associate with survival/mortality and IHG status in this cohort.

Recruitment of the Mexican American families in the SAFHS began in 1991 with ascertainment on family size rather than any disease state, although the cohort reflects the elevated risk of this ethnic stratum for type 2 diabetes (15.3% at recruitment) and other cardiovascular risk factors. Participants have been recalled up to three times to provide a wealth of genetic and phenotypic data. The *n*=1240 SAFHS participants with gene expression data represent 46 extended families ranging in size from 3 to 87 phenotyped relatives.

4.10.2.2. Brisbane Systems Genetics study (GSE53195; Figure 3c, main)

The Brisbane Systems Genetics Study comprises a total of 862 individuals from 374 families. Families consist of combinations of both MZ and DZ twin pairs, their siblings, and for 72 families, their parents (Chundru et al., 2023).

4.10.2.3. Datasets from Vitality 90+ cohort (GSE65218; Figure 5d-e, main)

Cohort/sample description. PBMC samples from nonagenarian participants (*n*=151; ages: ≥90 years; females: 70%) in the Vitality 90+ Study(Goebeler et al., 2003; Jylhava et al., 2014). The all-cause mortality data (the median follow-up time was 2.55 years) (Jylhava et al., 2014), including the dates of death, were collected from the Population Register Center. The mortality rate during the follow-up was 32.5%; of the 151 individuals, 49 died and 102 survived the follow-up period. There were no losses to follow-up.

<u>4.10.3 Inflammatory conditions and “premature aging” cohorts</u>

Systemic lupus erythematosus (SLE) cohort (GSE65391; Figure 2b, main).

Cohort/sample description. The study recruited pediatric patients with SLE. Gene expression profiling of healthy controls (*n*=48) and SLE patients (*n*=158; median age [IQR] = 14.49 [6-21] years; females: 87%). (Banchereau et al., 2016)

Down syndrome (GSE183701; Permutational immune analysis reveals architectural similarities between inflammaging, Down syndrome, and autoimmunity; Figure 2b, main) (K. Lambert et al., 2022).

Common variable immune deficiency (GSE51405; Interferon Signature in the Blood in Inflammatory Common Variable Immune Deficiency [Training Set]; Figure 2b, main) (Park et al., 2013).

Metabolic syndrome (GSE145412; Evaluation of transcriptomic regulations behind metabolic syndrome in obese and lean subjects; Figure 2b, main) (Paczkowska-Abdulsalam et al., 2020).

ST elevation myocardial infarction (STEMI)/ congestive heart failure (CHF) (GSE59867; Gene expression profiling reveals potential prognostic biomarkers associated with the progression of heart failure; Figure 5b, main; Case 5) (Maciejak et al., 2015).

4.10.4 Infection (non-COVID-19) Cohorts

4.10.4.1 Natural influenza season and other acute respiratory viral infections (GSE68310 (Zhai et al., 2015); Figure 4b-c, main; Case 1).

To understand the molecular basis and network orchestration of host responses, Zhai et al. (Zhai et al., 2015) prospectively enrolled 1,610 healthy adults in the fall of 2009 and 2010, followed the subjects with influenza-like illness (*n*=133) for 3 weeks, and examined changes in their peripheral blood gene expression. About 133 participants completed all study visits and yielded technically adequate peripheral blood microarray gene expression data. Seventy-three (55%) had an influenza virus infection, 64 influenza A and

9 influenza B. The remaining subjects had a rhinovirus infection (*n*=32), other viral infections (*n*=4), or no viral agent identified (*n*=24). They analyzed the global gene expression profiles of peripheral whole blood in the 133 adults with an acute respiratory infection at up to seven time points before, during, and after the occurrence of illness (**adjacent figure**). They discovered distinct phases of the host response spanning 6 days after infection and identified genes that differentiate influenza from non-influenza virus infection. Samples from consecutive influenza seasons (2009-2010 and 2010-2011) were pooled for this analysis.

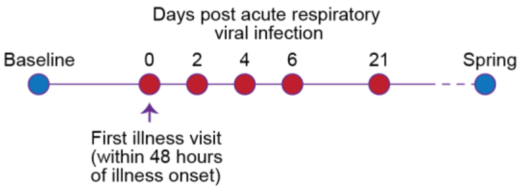

4.9.4.2 Influenza A H1N1 and H3N2 virus infection in adults (GSE52428 (Woods et al., 2013); Figure 4d-f, main; case 2).

Cohort/sample description. Woods et al. (Woods et al., 2013) used microarrays to assay peripheral blood gene expression at baseline and every 8 hours for 7 days following intranasal influenza A H1N1 or H3N2 inoculation in healthy volunteers (**adjacent Figure**).

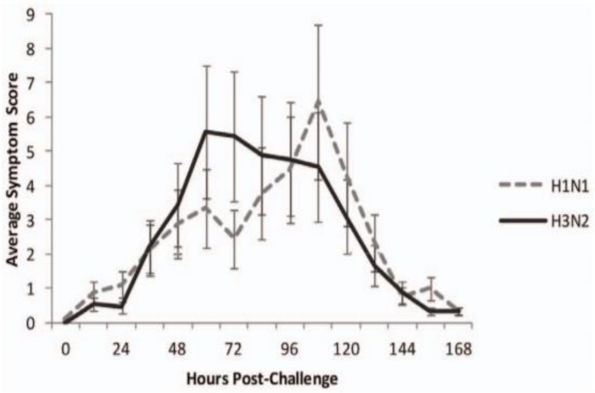

Figure reprinted from Woods et al. *PLoS One* 8, e52198 (2013)

4.10.4.3 Vaccine cohorts

- Influenza vaccine cohorts [SDY67, GSE47353, GSE74816, GSE166545, E-MTAB-2313, SDY296, GSE107990, GSE74813, GSE48023, GSE48018; Figure 4g, main and Figures S11-S20)
- COVID-19 vaccine cohort (GSE169159; Figure S21) (Arunachalam et al., 2021).

<u>4.10.5 Dementia disorders cohort</u>

The cohort developed by Nachun et al. (Nachun et al., 2019) comprised a diverse set of dementia disorders, including Alzheimer’s disease, mild cognitive impairment, and

frontotemporal dementia spectrum to study the effects of peripheral inflammation. Whole blood was collected from patients and controls in a cross-sectional study for microarray expression profiling (Figure 8b-d main; GSE140829).

4.10.6. Monogenic Immunodeficiencies Cohort

We leveraged the monogenic immunodeficiencies (MID) multiomics cohort assembled by Sparks et al. (Sparks et al., 2024) to identify plasma protein biomarkers correlating with transcriptomic (SAS-1/MAS-1) metrics of IR (Figure 9a-b and Figure S47).

Linear regression adjusting for age and sex was used to associate proteomic biomarker levels with expression of SAS-1 and MAS-1 gene expression signatures (Table S14a,b). A total of *n*=1304 plasma protein biomarker probes were available for *n*=233 samples with a paired microarray transcriptomic expression sample. Of the 1304 biomarkers, *n*=374 significantly associated with SAS-1 expression levels and *n*=299 significantly associated with MAS-1 expression levels after FDR correction within each analysis. Gene membership of the pathogenic triad readout gene expression signatures was used to find specific markers associated with salutogenesis, which comprised *n*=690 unique genes (Table S2). Of the 690 genes, *n*=144 genes had corresponding plasma protein biomarkers in the MID cohort multiomics dataset (Table S14b). The pathogenic triad gene membership of SAS-1-associated proteins was *n*=43 and MAS-1-associated proteins was *n*=33. The shared set of SAS-1 and MAS-1-associated proteins contained *n*=25 shared biomarkers with inverse directions (i.e., positive slope with SAS-1 and negative slope with MAS-1, or positive slope with MAS-1 and negative slope with SAS-1), comprising the IR-associated biomarker set (Figure 9a; Table S14a). Selected biomarker levels are depicted in Figure 9b with the full set presented in Figure S47.

This work was supported by the following Intramural Research Programs of the National Institutes of Health (NIH):

1. The National Heart, Lung, and Blood Institute (NHLBI; ZIA-HL006089-12) to Dr. Neal Young, who is an Associate Investigator on NHLBI Protocol 04H0012 –

Tissue Procurement Protocol (NCT00071045), where Dr. Richard Childs from the NHLBI Intramural Program, NIH, is the Principal Investigator.

2. The National Human Genome Research Institute (NHGRI). The Inflammatory Diseases Section (IDS) at the NHGRI is dedicated to the discovery of rare, high-penetrance mutations, as well as more common variants, associated with inflammatory disease susceptibility and phenotypes. NIH grant numbers associated with this program: 1ZIAHG200371, 1ZIAHG200373, and 1ZIAHG200374.
3. The National Institute for Allergy and Infectious Diseases (NIAID; protocols: 00-I-0159, 01-I-0202, 07-I-0033, 13-I-0157, 15-I-0162, 91-I-0140, and 93-I-0119). NIH grant numbers associated with this program: Z-01-A-00647, Z-01-A-00646, 1 ZIA AI001202-07, Z01 AI000825-24 LIR, Z-01-A-00646

The original data description for the NIH Division of Intramural Research Multiomic Monogenic Disease Study can be found here: (Nat Med. 2024 Sep;30(9):2461-2472. doi: 10.1038/s41591-024-03092-6. Epub 2024 Jul 3.) (Sparks et al., 2024).The original data used for the analysis in this publication can be found in the NIH database of Genotypes and Phenotypes (dbGaP) under the accession numbers: phs001860.v1.p1 and phs002732.v1.p1.

4.11. Transcription factor analysis

Based on the membership of *TCF7* in SAS-1 (Figure 1f), we focused on this gene as a possible candidate transcription factor to sustain optimal IR. We used an agnostic approach to bait other transcription factors that may sustain a higher representation of SAS-1^{high}-MAS-1^{low} (figure below, Figure S44). A list of 1639 transcription factors (TF) listed by Lambert et al. (S. A. Lambert et al., 2018) was used in this analysis, of which 1380 TFs were present in the gene expression dataset of FHS. The associations of the median, quantile, and octile groups of these TFs with median-based groups of SAS-1, MAS-1, and its combination were assessed (Figure S44). Based on the following criteria (also listed in the figure below panel c), we identified 7 transcription factors: *TCF7, LEF1*, *ZBTB25*, *GATA3*, *SATB11*, *ETS1*, and *KLF12* genes (Figure S44).

1. Levels of SAS-1^{Hi} is 25% more in M2 of TF compared to M1 of TF, top left in Fig. panel B (# of genes – 11)
2. Q4 of TF contains 25% more SAS-1^{Hi} compared to Q1 of TF, top middle in Fig. panel B (# of genes – 52)
3. Levels of SAS-1^{Hi} consistently higher (up) with octile groups of TF, top right in Fig. panel B (# of genes – 46)
4. Levels of SAS-1^{Hi}/MAS-1^{Lo} (blue) group is consistently higher and levels of SAS-1^{Lo}-MAS-1^{Hi} (yellow) group is consistently lower (down) with median groups of TF, bottom left in Fig. panel B (# of genes – 538)
5. Levels of SAS-1^{Hi}/MAS-1^{Lo} (blue) group is consistently higher and levels of SAS-1^{Lo}-MAS-1^{Hi} (yellow) group is consistently lower with quartile groups of TF, bottom middle in Fig. panel B (# of genes – 289)
6. Levels of SAS-1^{Hi}/MAS-1^{Lo} (blue) group is consistently higher and levels of SAS-1^{Lo}-MAS-1^{Hi} (yellow) group is consistently lower with octile groups of TF, bottom right in Fig. panel B (# of genes – 29)

Overlap of the TFs in criteria 1-6 above resulted in 7 core TFs associated with higher levels of SAS-1^{Hi} and promoting SAS-1^{Hi}-MAS-1^{Lo}. Progressively higher levels of these transcription factors were associated with progressively greater representation of SAS-1^{Hi}-MAS-1^{Lo} in the FHS (Figure S44). In addition to *TCF7*, three other transcription factors were associated with increased lifespan (*SATB1*, *ETS1*, *KLF12*) (Figure S44).

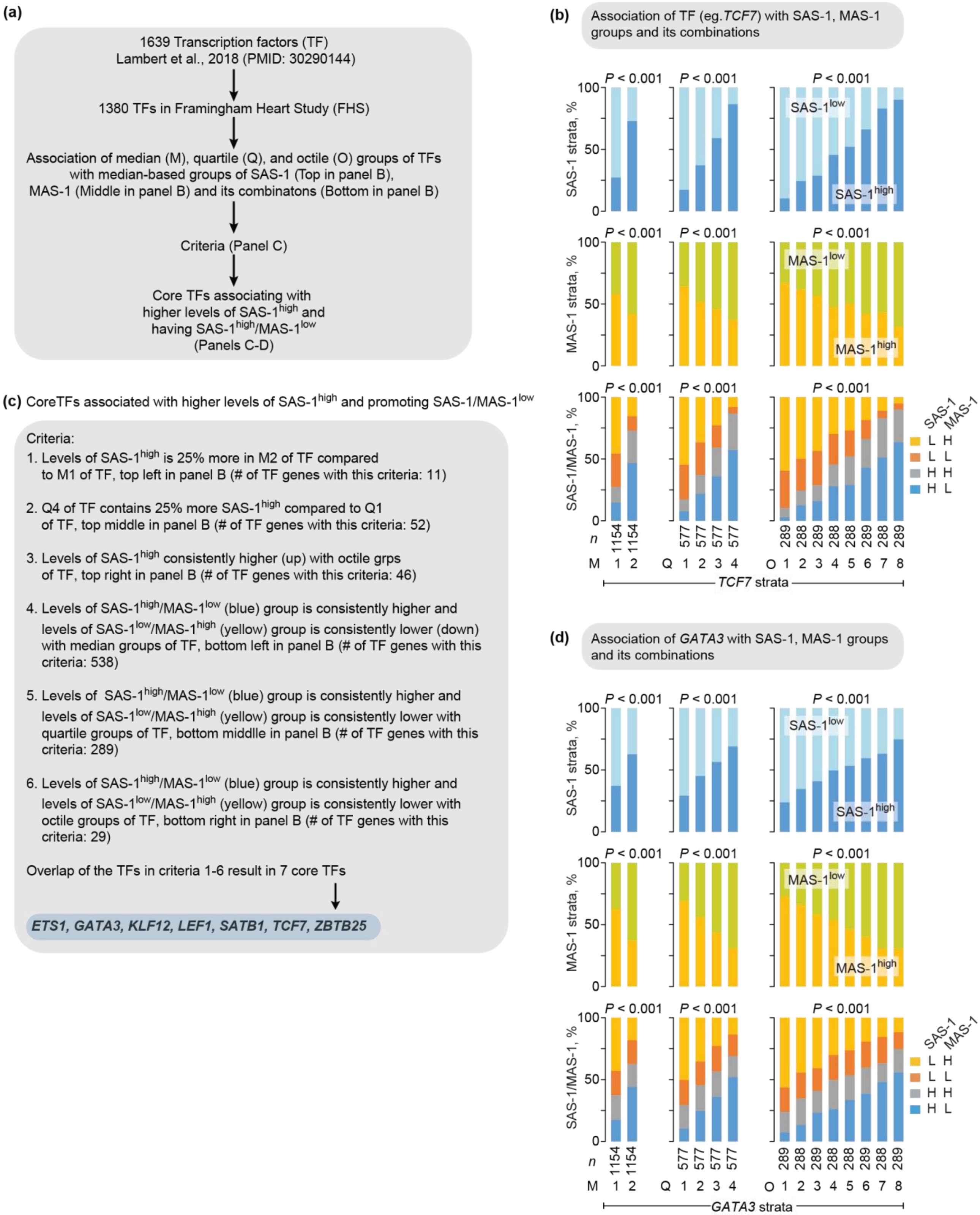

Figure. (a) Flow chart of analysis as described above. **(b)** Stacked barplots depict distribution of SAS-1 strata, MAS-1 strata, and SAS-1/MAS-1 profiles in the FHS cohort by *TCF7* strata (NGE; medians (M),

quartiles (Q), and octiles(O) groups). *P*, by χ^2 test. **(c)** Criteria for selection of transcription factors (TFs) associated with SAS-1^{high} and promoting SAS-1^{high}-MAS-1^{low}. **(d)** Example of core TFs (*GATA3*) associated with SAS-1^{high} and promoting SAS-1^{high}-MAS-1^{low}. Plots similar to panel B. *P*, by χ^2 test.

5. Statistical approach and plots

All analysis was conducted using R (R Foundation for Statistical Computing). Reported significance values are two-sided and set at the 0.05 significance level. The models and significance values were not adjusted for multiple comparisons in the prespecified subgroup analyses, unless otherwise noted. Missing data were random and therefore not imputed.

If not otherwise noted as being FDR or Bonferroni corrected, the *P* values presented are nominal. Best practices were applied when determining when to use an FDR or Bonferroni correction vs. nominal *P* value as described below.

5.1. Clustering

Unsupervised or supervised hierarchical clustering was performed using Euclidean distances with Ward's D linkage. Clustering of gene signatures (Figure 1g and Figure S2) and biomarkers (Figures S42 and S50a-b) was performed.

5.2. Timing of assessments

Baseline indicates the date of the first available IHG measurement. For RNA-Seq, FACS, cytokine, and %inhibition data, the measurement concurrent with the first available RNA-seq measurement was used. The median (IQR) number of days between baseline and first available RNA-seq measurement was 1 day (0-1). By co-indexing the RNA-seq, FACS, cytokine, and %inhibition levels with baseline IHG (IR status), the relationships among concurrent measurements were determined. For longitudinal analysis, days from baseline was used.

5.3. Associations of %inhibition levels

Out of 522 patients in the acute COVID-19 cohort, 454 had a %inhibition assessment at baseline (± 2 days), with baseline being defined as the date of the first IHG measurement.

The %inhibition values were categorized as <30% vs. ≥30% or <50% vs. ≥50% and analyzed using logistic regression to identify predictor and outcomes associations.

Longitudinal analysis was performed (*n*=454) to determine the change in %inhibition levels by IHG status from baseline to 7 days post baseline. In this analysis, the concurrent day IHG and *n*=1,399 %inhibition measurements were evaluated using linear generalized estimating equation (GEE) models with an autoregressive 1 (AR(1)) correlation structure (shown in Figure S33).

Association between %inhibition and nAb concentrations. The relationship between SARS-CoV-2 nAb concentrations (GenScript, A02087) and %inhibition (methods are detailed in Section 2.2) in samples from *n*=145 subjects collected longitudinally was determined. The median (IQR) of the number of measurements taken per subject is 1 (1-5), with two subjects having 15 measurements. Measurements (%inhibition and nAb concentration) were determined between 0 to 14 days from baseline, and the median (IQR) interval between baseline and assessment was 4 (1-8) days. Spearman’s correlation coefficient and a cubic GEE model with an AR(1) correlation structure and adjusted by time was used to examine the relationship. Results are presented in the embedded **figure in Section 2.2**.

5.4. Multiomic associations

The immunologic trait, cytokine levels, and %inhibition values used were the values from the same date as the first available gene signature score used in the clustering analysis. Immunophenotype and cytokine data were $\log_2$-transformed. The association of the immunologic trait and cytokine level data with outcomes (IHG, hospitalization, 30-day survival, %inhibition, and gene signature score clusters) was determined using a linear model with a likelihood ratio test (LRT). The significance values for the immunophenotypes and cytokines were independently FDR corrected on a per outcome basis with an FDR<0.05 considered as significant.

5.5. General statistical methods

Linear models with LRT, quasi-Poisson generalized linear model, logistic regression, Cox proportional hazards model, Fisher's exact test, χ^2 test, and ordinal regression were used where appropriate. Relative risk, attributable fraction (AF), and population attributable fraction (PAF) were used to examine IHGs' association with 30-day acute COVID-19 mortality. Analyses for age were adjusted by IHG and the analysis for IHG was adjusted by age. The PAF was calculated using a logistic regression model with the R package AF (Pace et al., 1989). The non-parametric Kruskal-Wallis test was used where appropriate for continuous data. For longitudinal analysis, linear GEE models with an AR(1) correlation structure were used. FDR corrections were performed where applicable and adjusted *P* values are indicated. Adjustments were made for repeated measures when comparing multiple measurements from the same individuals where applicable. Detailed statistical analyses per-panel are discussed below.

5.6. Plots

Boxplots (median and IQR) were used to plot gene signature scores, cytokines, immune traits, %inhibition levels, and biomarkers by different groups within the datasets. Kaplan-Meier (KM) plots were used to display the proportion of patients who survived over time after admission or indicated timepoints. Dot-and-line plots were used to plot odds ratios, rate ratios with 95% confidence intervals, as well as median (IQR) of *TCF7* gene expression. Stacked barplots were used to plot the distributions of categorical variables. Line plots with confidence interval shadings were used to depict modeling of biomarkers longitudinally or with age. Line plots with error bars (mean and standard error) were used to depict expression levels of gene signature(s) or normalized gene expression (NGE) of indicated gene(s). Dot plots were used to depict relative expression of the gene signatures or NGE of the indicated genes in scRNA-seq datasets.

5.7. Statistical approach: Principles

Our hypothesis is based on the concept that in response to environmental stressors, including infections, individuals may manifest robustness, plasticity, or maladaptation

(**main Figure 4a and 9d**). Without intensive prospective monitoring, it is challenging to distinguish between these responses.

5.7a. Rationale for studying distinct cohorts and the comparisons made

1. While we articulated an omnibus or overarching hypothesis, this hypothesis has distinct facets depending on the clinical or biological context.
2. We tested specific hypotheses relevant to IR within distinct clinical or biological contexts. Each context has no direct relationship with any context other than environmental/antigenic stress.
3. It was impossible to study a single cohort or group of individuals to test whether IR responses influence varied outcomes (CVD, lifespan, COVID-19, biomarkers).
4. For these reasons, we examined IR metrics in distinct cohorts that permitted us to address specific questions that are aligned with the overarching hypothesis. Thus, although conceptual overlaps may exist, each cohort provided a unique biological context.
5. We performed comparisons to account for the effects of age and sex, as during aging some persons may preserve IR, and females preserve optimal IR to a greater extent than men. In our analyses, where possible, we (i) adjusted for age and/or sex, or (ii) performed overall comparisons, followed by stratification by age strata and sex.
6. Additionally, we could not apply the same IR metric or strata of a metric across each of the cohorts. For some analyses, we used IHGs in the context of COVID-19 and in others we used transcriptomic metrics of IR (SAS-1/MAS-1 profiles). However, we validated the relationship between SAS-1/MAS-1 profiles as transcriptomic proxies for IHGs. This was important, as while gene expression studies are commonplace, assessments of $CD4^+$ and $CD8^+$ T-cell counts required for derivations of IHGs are uncommon in most cohort-based studies.
7. Additionally, we define mechanisms by which IR status influences outcomes (e.g., burden of pathogenic triad).
8. <u>Summary</u>: For these reasons, it is not possible to apply an omnibus statistical test across these varied cohorts. Each cohort was distinct, and each comparison

addressed a specific question using the best statistical practices and accounted for multiple comparisons and relevant variables that may influence the results (e.g., age, sex).

5.7b. Biological plausibility and statistical approach

A key goal of our study was to examine the biological plausibility of our overarching hypothesis wherein the general principles of IR are applicable to distinct biological/clinical contexts studied in distinct cohorts. Thus, it was to be anticipated that IR metrics will show statistically significant differences in varied cohorts.

5.7c. Multiple comparisons

We applied the following guidelines to our statistical approach:

1. Our statistical design focused on determining whether the directions and magnitudes of differences (perhaps including some trending with $P>0.05$ and <0.10) fit a biologically coherent pattern vs. chance findings.
2. Our approach to multiple comparisons adheres to best practices. Depending on the question asked and nature of the dataset, we provide (i) an overall omnibus test (e.g., ANOVA, LRT), (ii) FDR or Bonferroni corrections, and (iii) nominal *P* values, without adjustment for multiple testing.
3. Thus, we individualized the analysis of each dataset, adhering to these best practices:
    a) We based interpretations on a synthesis of statistical results with scientific considerations.
    b) We relied on scientific considerations to guard against overinterpreting findings with $P<0.05$.
    c) Where appropriate we determined group level differences followed by post hoc testing.
    d) The assumptions of each statistical test were evaluated to ensure that associated proposed analyses were appropriate (e.g., normality, homogeneity, linearity, and independence).
    e) We provide estimates with confidence intervals.

f) We chose accuracy, scientific judgment, and biological plausibility balanced by appropriate statistical testing for multiple comparisons. We are mindful that conventions for statistical analysis and interpretation have emerged from a formal statistical hypothesis testing paradigm, guarding against chance false-positive results by application of multiple comparison adjustments. However, these paradigms are inherently focused only on *P* values, promoting the use of the *P* value fallacy. These adjustments also have the property that the results of each analysis are automatically assumed to detract from all the others, with no consideration of how well the different results fit together conceptually or scientifically (Bacchetti, 2002; Rothman, 1990). This general approach has been criticized as unreliable and contrary to the original statistical theories that supposedly support it (Armstrong, 2007; Cohen, 1994; Gardner & Altman, 1986; Gigerenzer, 2004; Goodman, 1999), but it remains engrained in research culture (Lecoutre et al., 2001; Silva-Aycaguer et al., 2010).

g) Taken together, our overall statistical approach balances the considerations in points 3a to 3f.

6. Statistical analyses

6.1. Main figure statistical methods used in each figure panel

6.1.1. Figure 1

- Panels a-c:
  - Schema; no statistical analysis was performed.
- Panel d:
  - Hazard ratios (with 95% CI) of mortality in the FHS for the indicated gene signatures (z-scores) and genes (*TCF7*, *LEF1*). Analysis performed using Cox proportional hazards models adjusting for age and sex. FDR correction performed using Benjamini-Hochberg method.
- Panels e-f:
  - Schema; no statistical analysis was performed.
- Panel g:

- Correlation heatmap of indicated gene expression signatures, genes, and chronological age with clustering performed using 1 – Pearson's correlation coefficient as the distance with Ward's D linkage. Color gradient corresponding to correlation coefficient.

6.1.2. Figure 2

- Panel a:
  - PBMC 10k scRNA-seq dataset
  - Dot plots depict the expression of the indicated gene signatures (z-scores) and genes (NGE). Color gradient corresponding to z-score or NGE expression ranging from low to high, blue to white to red. The size of the dot corresponds to the percent expressed in each cluster. No statistical testing was performed.
- Panel b:
  - (*top*) Stacked barplots depict distributions of SAS-1/MAS-1 profiles with *P* values, and (*bottom*) dot-and-line plots depict median (IQR) NGE of *TCF7* in the following datasets (*left to right*):
  - Framingham Heart Study (FHS) Offspring cohort by age strata. *P*, by χ^2 test.
  - Down syndrome registry with controls (GSE183701). *P*, by Fisher's exact test.
  - Common variable immunodeficiency (CVID) with controls (GSE51405). *P*, by Fisher's exact test.
  - Systemic lupus erythematosus (SLE) by total lymphocyte % strata (GSE65391). *P*, by χ^2 test.
  - HIV+ and tuberculosis cohorts (GSE39941). *P*, by Fisher's exact test.
  - Metabolic syndrome cohort (GSE145412). *P*, by Fisher's exact test.
- Panel c:
  - Schema: no statistical testing was performed.
- Panel d:
  - Hazard ratios (with 95% CI) of mortality in the FHS stratified by baseline SAS-1/MAS-1 profiles and the indicated age strata. Analysis performed using Cox proportional hazards models adjusted for sex. General linear hypothesis testing was used to compare the indicated coefficients from the model.
- Panel e:

- Modeled line plots depict the hazard of mortality with age in the FHS for persons with the indicated SAS-1/MAS-1 profiles. Shaded area depicts the 95% confidence bands. Analysis performed using Cox proportional hazards model adjusted for sex, including an interaction term.

- Panel f:
  - Kaplan-Meier plots depict time to mortality in the FHS stratified by the indicated SAS-1/MAS-1 profiles and age strata (split by median age: 66 y). *P*, by log-rank test.
- Panel g:
  - Hazard ratios (with 95% CI) of mortality in the FHS stratified by the SAS-1/MAS-1 profiles within the indicated age strata. Analysis performed using Cox proportional hazards models adjusted for sex.

6.1.3. Figure 3

- Panel a:
  - Linear regression modeling of indicated gene signature expression (z-score) and *TCF7* (NGE) by age in the FHS stratified by (*top*) sex and (*bottom*) SAS-1/MAS-1 profiles at baseline. Shaded area depicts the 95% confidence bands. *P*, by linear regression model testing for association with group, age, and an interaction term.
- Panel b:
  - (*left*) Boxplots depict median (IQR) expression of indicated gene signature expression levels (z-scores) in the FHS stratified by age and SAS-1/MAS-1 profiles. *P*, by linear model with likelihood ratio test (LRT).
- Panel c:
  - Mean difference of association (β values) between age and SAS-1/MAS-1 profiles in the FHS, Mexican American cohort (E-TABM-305), and Brisbane cohort (GSE53195) stratified by age and SAS-1/MAS-1 profiles. *P*, by general linear hypothesis testing if the β values differ between IR comparison (L-H vs. H-L) and age comparison (O vs. Y).
- Panel d:
  - Schema; no statistical testing was performed.

- Panel e:
  - Stacked barplots depict distributions of SAS-1 and MAS-1 high/low (split by median expression) by octiles of *TCF7* expression, overall and within younger and older groups (stratified by median age: 66 y). *P*, by χ^2 test.
- Panel f:
  - Modeling of the odds of having the indicated SAS-1/MAS-1 profile at baseline and age strata by *TCF7* levels (NGE). Shaded area depicts the 95% confidence bands. Analysis performed using logistic regression.

6.1.4. Figure 4

- Panel a:
  - Schema; no statistical testing was performed.
- Panel b:
  - Natural viral infection cohort during influenza season (GSE68310).
  - Stacked barplots depict the distribution of SAS-1/MAS-1 profiles over the indicated timepoints within participants who presented with SAS-1^{high}-MAS-1^{low} at baseline.
- Panel c:
  - Natural viral infection cohort during influenza season (GSE68310).
  - Line plots depict expression (mean ± SEM) of indicated gene signatures (z-score) and *TCF7* (NGE) stratified by IR-degraders, IR-reconstituters, and IR-preservers as indicated in panel B. *P,* by ANOVA for comparing the expression in the three IR groups (preservers, reconstituters and degraders) at final timepoint (T6).
- Panel d:
  - Influenza challenge cohort (GSE54248), *left to right*:
  - Single stacked barplots depict distribution of SAS-1/MAS-1 profiles before virus inoculation (*n*=10, 7, 6 unique subjects).
  - Single stacked barplots depict SAS-1/MAS-1 profiles pooled over time after virus inoculation (*n*=138, 96, 83 measurements excluding baseline).
  - No statistical testing was performed.
- Panel e:

   - Influenza challenge cohort (GSE54248)
   - Stacked barplots depict distribution of SAS-1/MAS-1 profiles over the indicated timepoints (hours). *P*, by Fisher's exact test.
- Panel f:
   - Influenza challenge cohort (GSE54248)
   - Line plots depict expression (mean ± SEM) of indicated gene signatures (z-score) and *TCF7* (NGE) over the indicated timepoints (hours) by baseline SAS-1/MAS-1 profiles and symptom status combination. *P* for group, determined by linear GEE comparing asymptomatic H-L vs symptomatic L-H adjusting for time. *P* for time, determined by linear GEE adjusting for group with ANOVA.
- Panel g:
   - Influenza vaccine cohort (SDY67), *left to right*:
   - Stacked barplots depict the distribution of the SAS-1/MAS-1 profiles by timepoint. *P*, by Fisher's exact test.
   - Line plots depict expression (mean ± SEM) of the indicated gene signatures (z-score) over the indicated timepoints (days) by indicated IR groups. *P*, by Welch's t-test at final timepoint (day 28).

6.1.5. Figure 5

- Panel a:
   - RSV infection cohort (GSE188427)
   - Stacked barplots depict distribution of SAS-1/MAS-1 profiles in controls (one time point) and patients at the indicated timepoints. Dashed lines indicate levels of H-L profile. *P*, by χ^2 test.
- Panel b:
   - STEMI cohort (GSE59867)
   - Stacked barplots depict the distribution of the SAS-1/MAS-1 profiles at the indicated timepoints overall and by heart failure status. *P*, by χ^2 test between the indicated groups.
- Panel c:
   - STEMI cohort (GSE59867)

    - Line plots depict expression (mean ± SEM) of the indicated gene signatures (z-score) and *TCF7* (NGE) over the indicated timepoints by baseline SAS-1/MAS-1 profiles. *P* for group, determined by linear GEE comparing H-L vs. L-H overall adjusting for time. *P* for time, determined by linear GEE adjusting for group with ANOVA.
- Panel d:
    - Vitality 90+ study (GSE65218), *left to right*:
    - Stacked barplots depict the distributions of baseline SAS-1/MAS-1 profiles by quartile groups of circulating cell-free DNA (cfDNA) levels. *P*, by χ^2 test between the indicated groups.
    - Kaplan-Meier plot depict time to death by quartiles of cfDNA levels. *P*, determined by log-rank test.
    - Stacked barplots depict distributions of SAS-1/MAS-1 profiles by *TCF7* expression tertiles within quartile 4 (Q4) of cfDNA levels vs. rest (Q1-Q3 pooled). *P,* by Fisher's exact test comparing tertile strata of *TCF7* levels (tertile 1 vs tertiles 2 & 3 pooled).
- Panel e:
    - Vitality 90+ study (GSE65218)
    - Boxplots depict median (IQR) levels of cfDNA and the indicated gene signature expression (z-scores) by SAS-1/MAS-1 profiles. *P* for cfDNA, determined by Kruskal-Wallis test. *P* for z-scores, determined by ANOVA.
- Panel f:
    - Schema; no statistical testing was performed.
- Panel g:
    - Kaplan-Meier plot depicts time to death in the FHS stratified by Age_IL6^{up} signature strata (split by median expression) and baseline CVD status. *P*, by Cox proportional hazards models adjusted for age, sex, smoking status, diabetes, hypertension, and total cholesterol with likelihood ratio test (LRT).
- Panel h:
    - Stacked barplots depict the distribution of Age_IL6^{up} strata (split by median expression) in the FHS stratified by SAS-1/MAS-1 profiles. *P*, by Cox

proportional hazards models adjusted for age, sex, smoking status, diabetes, hypertension, and total cholesterol with the LRT.

- Panel i:
  - Kaplan-Meier plot depicts time to CVD diagnosis stratified by SAS-1/MAS-1 profiles and Age_IL6^{up} strata (split by median expression). *P*, by Cox proportional hazards models adjusted for age, sex, smoking status, diabetes, hypertension, and total cholesterol with the LRT.
- Panel j:
  - Kaplan-Meier plot depicts time to death in the FHS stratified by SAS-1/MAS-1 profiles and Age_IL6^{up} strata (tertiles) within persons without a CVD diagnosis at baseline. *P*, by Cox proportional hazards models adjusted for age, sex, smoking status, diabetes, hypertension, and total cholesterol with the LRT.
- Panel k:
  - Boxplots depict median (IQR) levels of Age_IL6^{up} gene signature (z-score) and *TCF7* (NGE) by the indicated groups in panel J. *P* for Age_IL6^{up} determined by ANOVA and *P* for *TCF7* determined by the Kruskal-Wallis test.
- Panel l:
  - Stacked barplots depict the distribution of SAS-1/MAS-1 profiles in the FHS subset without baseline diagnosis of CVD stratified by incident CVD diagnosis and all-cause mortality. *P*, by χ^2 test.

6.1.6. Figure 6

- Panel a:
  - Stacked barplots depict the distribution of SAS-1/MAS-1 profiles in the FHS overall, and IHG with subgrades in the control SAIL cohort (left) and acute COVID-19 VA-CLC (right). *P*, by χ^2 test.
- Panel b:
  - Schema; no statistical testing was performed.
- Panel c:
  - Percent of each IHG observed in the indicated cohorts. No statistical testing was performed.
- Panel d:

    - Stacked barplots depict the distribution of IHG with subgrades in the VA-CLC stratified by sex, hospitalization, and survival status. Dashed line indicates level of IHG-I. *P*, by Fisher's exact test.
- Panel e:
    - Modeling line plots with 95% confidence bands depict the probability of having a non-IHG-I grade by age and sex in the non-COVID-19 cohorts (SAIL, SardiNIA, and UCSD cohorts pooled) and ac-COVID-19 (VA-CLC cohort). *P*, by linear regression.
- Panel f:
    - Kaplan-Meier plot depicts time to achieve IHG-I within 30 days from baseline stratified by baseline IHG. *P*, by age-adjusted Cox proportional hazards model.
- Panel g:
    - Stacked barplots depict the distribution of SAS-1/MAS-1 profiles stratified by IHG with subgrades in the VA-CLC. Expression data within 14 days from IHG baseline of 46 participants (228 measurements) were used to determine the SAS-1/MAS-1 profiles and compared to concurrent IHG measurements. IHG baseline is the date of the first available IHG for each participant. Note: There were 2 non-hospitalized individuals with no RNA-Seq measurements within this time window. *P*, by Fisher's exact test.
- Panel h:
    - Stacked barplots depict the distribution of SAS-1/MAS-1 profiles in the VA-CLC stratified by hospitalization and survival status. Expression data from RNA-Seq baseline of 48 participants were used to determine the SAS-1/MAS-1 profiles and compared to COVID-19 outcomes by 30-day survival status. RNA-Seq baseline is the date of the first available RNA-Seq data for each participant. *P*, by Fisher's exact test.
- Panel i:
    - Stacked barplots depict the distribution of SAS-1/MAS-1 profiles in the NYU COVID-19 cohort stratified by survival status. *P*, by χ^2 test.
- Panel j:

- Modeling line plots with 95% confidence bands depict the probability of hospitalization with age stratified by baseline IHG with subgrades in the VA-CLC. Modeling was performed using logistic regression adjusted for sex. *P* for overall age and IHG, determined by logistic regression and LRT. Adjusted odds ratio (aOR) and *P* for individual IHGs, determined by logistic regression.

6.1.7. Figure 7

- Panel a:
  - Point estimates with 95% CI of hazard ratios of mortality in the VA-CLC for acute mortality (30-day mortality) and post-acute mortality (24-month mortality). Analysis performed using Cox proportional hazards models adjusted for sex.
- Panel b:
  - Log_2-transformed odds of the indicated variables and hazard ratios of acute or post-acute mortality by baseline IHG status in the VA-CLC. Lines indicate 95% CI. *P*, by logistic regression and Cox proportional hazards models adjusted for age.
- Panel c:
  - Boxplots depict median (IQR) expression levels of the indicated gene signatures (z-scores) and *TCF7* (NGE) by baseline IHG status in the VA-CLC. *P*, by likelihood ratio test (LRT).
- Panel d:
  - Modeling line plots of the indicated biomarkers with age by baseline IHG status in the VA-CLC. Shaded area depicts the 95% confidence bands. Analysis was performed using linear regression comparing IHG-I vs IHG-IIa and the rest were pooled.
- Panel e:
  - Age-adjusted mean values with 95% CI of log_2-transformed CRP levels by comorbidity score strata and baseline IHG with subgrades. *P*, by linear regression with comorbidity score by indicated IHG.
- Panel f:
  - Pearson's correlation coefficient of indicated gene signatures and immune traits and cytokines with *TCF7* (NGE) in the VA-CLC.

6.1.8. Figure 8

- Panel a:
  - FHS cohort
  - Linear regression modeling of the *LRNN3* (NGE) by age in the FHS stratified by (*top*) sex and (*bottom*) SAS-1/MAS-1 profiles. Shaded area depicts the 95% confidence bands. *P,* by linear regression testing for association with sex or group, age, and an interaction term.
- Panel b:
  - Alzheimer's disease cohort (GSE140829).
  - Stacked barplots depict the distribution of SAS-1/MAS-1 profiles and dot-and-line plots depict median (IQR) levels of *TCF7* stratified by age strata and disease status in the Alzheimer's disease cohort (GSE140829). No statistical testing was performed.
- Panel c:
  - Alzheimer's disease cohort (GSE140829).
  - Linear regression modeling of the expression of indicated gene signatures (z-score) across age stratified by disease status. Shaded area depicts the 95% confidence bands. *P*, by linear regression testing for association with group, age, and an interaction term.
- Panel d:
  - Alzheimer's disease cohort (GSE140829).
  - Linear regression modeling of indicated gene signature expression (z-score) across age stratified by SAS-1/MAS-1 profiles within Alzheimer's disease patients only. Shaded area depicts the 95% confidence bands. *P*, by linear regression testing for association with group, age, and an interaction term.
- Panel e:
  - Modeling line plots of the indicated biomarkers longitudinally (days from baseline) by IR-preserver (maintained IHG-I) and extreme IR-degraders who improved their IHG status (IHG-IIc/IVc to better IHG) in the VA-CLC. Shaded area depicts the 95% confidence bands. Analysis was performed using a linear generalized estimating equations model with an autoregressive 1 correlation

structure. The model includes days from baseline (0 to 14), IHG-I vs IHG-IIc/IVc, and the interaction term between the two to test whether the slopes over time differed by IHG-I vs. IHG-IIc/IVc. A generalized linear hypothesis test was used to compare IHG-I vs. IHG-IIc/IVc at days 0 and 14.

- Panel f:
  - Stacked barplots depict distribution of convalescent IHG with subgrades in the VA-CLC stratified by baseline IHG. Dashed line indicates level of IHG-I. No statistical testing was performed.

6.1.9. Figure 9

- Panel a:
  - MID cohort transcriptomics and proteomics datasets
  - Linear slope β values (with 95% CI) of the indicated proteomic biomarker expression levels versus SAS-1 and MAS-1 expression levels. Analysis was performed using linear regression adjusting for age and sex. FDR correction was performed using the Benjamini-Hochberg method. FDR was calculated in total analyses (**Table S14b**) across *n*=1304 comparisons within SAS-1 and MAS-1 associations. Analysis and derivation outlined in Section 4.10.6.
- Panel b:
  - MID cohort transcriptomics and proteomics datasets
  - Boxplots depict median (IQR) levels of indicated proteomic biomarker expression levels stratified by SAS-1/MAS-1 profiles. *P,* determined by linear regression with ANOVA.
- Panel c:
  - Inflammatory bowel disease cohort (GSE191328).
  - Line plots (mean ± SEM) depict trajectories of the indicated gene signatures (z-scores) and *TCF7* (NGE) over time. *P*, by linear GEE comparing SAS-1/MAS-1 profiles overall adjusting for time.
- Panel d:
  - Schema; no statistical analysis was performed.
- Panel e:

- Schema; no statistical analysis was performed.

6.2. Supplementary figure statistical methods used in each figure panel

6.2.1. Figure S1

- Schema; no statistical testing was performed.

6.2.2. Figure S2

- Correlation plots of age, the indicated gene signatures, and transcription factor genes in the FHS with unsupervised hierarchical clustering performed using 1 – Pearson’s correlation coefficient as the distance with Ward’s D linkage. Correlation values are presented with color gradients and pie charts.

6.2.3. Figure S3

- Panels:
  - scRNA-seq datasets GSE94820, GSE106540, GSE115189, GSE119428, GSE124731, GSE128066, GSE150728, GSE150861, GSE167029, and supercentenarians dataset.
  - Dot plots depict expression of the indicated gene signatures (z-scores) and levels (NGE) of transcription factor genes (*TCF7*, *LEF1*). Color gradient corresponding to z-scores and NGE ranging from low to high, blue to white to red. The size of the dot corresponds to the percent expressed in each cluster. No statistical testing was performed.

6.2.4. Figure S4

- GSE179609:
  - Boxplots depict median (IQR) expression levels of the indicated gene signatures and *TCF7* (NGE) by cell types. *P*, by ANOVA for scores and by Kruskal-Wallis test for *TCF7*.

6.2.5. Figure S5

- ❖ Panel a (aging cohorts)
- E-TABM-305:

    - Stacked barplots depict the distribution of SAS-1/MAS-1 profiles and dot-and-line plots depict median (IQR) of *TCF7* (NGE) by age strata in the San Antonio Family Heart Study cohort. (E-TABM-305). *P*, by χ^2 test.
  - GSE53195:
    - Stacked barplots depict the distribution of SAS-1/MAS-1 profiles and dot-and-line plots depict median (IQR) of *TCF7* (NGE) by age strata in the Brisbane cohort. (GSE53195). *P*, by χ^2 test.
- Panel b (systemic lupus erythematosus; SLE):
  - GSE121239:
    - Stacked barplots depict the distribution of SAS-1/MAS-1 profiles and dot-and-line plots depict median (IQR) of *TCF7* (NGE) by SLEDAI strata (SLE disease activity index) and quartiles of imputed neutrophil percentages. *P*, by Fisher's exact test with 10,000 simulations.
  - GSE72509:
    - Stacked barplots depict the distribution of SAS-1/MAS-1 profiles and dot-and-line plots depict median (IQR) of *TCF7* (NGE) by ISM level (interferon signature metric) and anti-Ro autoantibody levels. *P*, by Fisher's exact test with 10,000 Monte Carlo simulations.
- Panel c (tuberculosis; TB):
  - GSE89403:
    - Stacked barplots depict the distribution of SAS-1/MAS-1 profiles and dot-and-line plots depict median (IQR) of *TCF7* (NGE) by cure status and timepoint (weeks) since starting therapy. *P*, by Fisher's exact test.
  - GSE69581:
    - Stacked barplots depict the distribution of SAS-1/MAS-1 profiles and dot-and-line plots depict median (IQR) of *TCF7* (NGE) by tuberculosis disease stage. *P*, by Fisher's exact test.
  - GSE79362:
    - Stacked barplots depict the distribution of SAS-1/MAS-1 profiles and dot-and-line plots depict median (IQR) of *TCF7* (NGE) by tuberculosis disease progression status. *P*, by χ^2 test.

❖ Panel d (malaria):

- GSE117613:
    - Stacked barplots depict the profiles of SAS-1/MAS-1 profiles and dot-and-line plots depict median (IQR) of *TCF7* (NGE) by infection status and quartiles of parasite density and the indicated biomarkers. *P*, by Fisher's exact test.

❖ Panel e (malaria):

- GSE52166:
    - Stacked barplots depict the distribution of SAS-1/MAS-1 profiles and dot-and-line plots depict median (IQR) of *TCF7* (NGE) by infection status and parasite density. *P*, by Fisher's exact test.

❖ Panel f (dengue):

- GSE28405:
    - Stacked barplots depict the distribution of SAS-1/MAS-1 profiles and dot-and-line plots depict median (IQR) of *TCF7* (NGE) by infection status and timepoint (days/weeks). *P*, by Fisher's exact test.
- GSE43777:
    - Stacked barplots depict the distribution of SAS-1/MAS-1 profiles and dot-and-line plots depict median (IQR) of *TCF7* (NGE) by disease stage. *P*, by Fisher's exact test.
- GSE140809:
    - Stacked barplots depict the distribution of SAS-1/MAS-1 profiles and dot-and-line plots depict median (IQR) of *TCF7* (NGE) by disease stage. (GSE140809). *P*, by Fisher's exact test.
- GSE100299:
    - Stacked barplots depict the distribution of SAS-1/MAS-1 profiles and dot-and-line plots depict median (IQR) of *TCF7* (NGE) by disease severity. *P*, by Fisher's exact test.

❖ Panel g (acute infections and inflammatory conditions):

- GSE157240:

  - Stacked barplots depict the distribution of SAS-1/MAS-1 profiles and dot-and-line plots depict median (IQR) of *TCF7* (NGE) by different viral infections. *P*, by Fisher's exact test with 10,000 simulations.
- GSE42026:
  - Stacked barplots depict the distribution of SAS-1/MAS-1 profiles and dot-and-line plots depict median (IQR) of *TCF7* (NGE) by different infections. *P*, by Fisher's exact test.
- GSE73464:
  - Stacked barplots depict the distribution of SAS-1/MAS-1 profiles and dot-and-line plots depict median (IQR) of *TCF7* (NGE) in persons with the indicated acute inflammatory conditions. *P*, by Fisher's exact test with 10,000 simulations.
- GSE80496:
  - Stacked barplots depict the distribution of SAS-1/MAS-1 profiles and dot-and-line plots depict median (IQR) of *TCF7* (NGE) by infection groups in a meningococcal infection cohort. *P*, by Fisher's exact test.
- GSE60244:
  - Stacked barplots depict the distribution of SAS-1/MAS-1 profiles and dot-and-line plots depict median (IQR) of *TCF7* (NGE) by bacterial and/or viral infection. *P*, by Fisher's exact test.
- GSE113866:
  - Stacked barplots depict the distribution of SAS-1/MAS-1 profiles and dot-and-line plots depict median (IQR) of *TCF7* (NGE) by disease group in an enteric fever cohort. *P*, by Fisher's exact test.
- GSE63881:
  - Stacked barplots depict the distribution of SAS-1/MAS-1 profiles and dot-and-line plots depict median (IQR) of *TCF7* (NGE) by disease timepoint (acute and convalescent) and age strata (4-33 and 34-182 months) in an acute Kawasaki disease cohort. Note: one non-paired sample was excluded from the plot. *P*, by Fisher's exact test with 10,000 simulations.
- GSE97741:

  - Stacked barplots depict the distribution of SAS-1/MAS-1 profiles and dot-and-line plots depict median (IQR) of *TCF7* (NGE) by RSV coinfection status and timepoint (acute and discharge). *P*, by Fisher's exact test with 10,000 simulations.
- GSE113210:
  - Stacked barplots depict the distribution of SAS-1/MAS-1 profiles and dot-and-line plots depict median (IQR) of *TCF7* (NGE) by timepoint (acute and convalescent) in the acute viral bronchiolitis cohort (GSE113210). *P*, by Fisher's exact test.
- GSE115823:
  - Stacked barplots depict the distribution of SAS-1/MAS-1 profiles and dot-and-line plots depict median (IQR) of *TCF7* (NGE) by quartiles of %lymphocytes and %neutrophils at baseline (Visit 0) in a children's asthma cohort. All samples provided on GEO were utilized for normalization and score calculation. Samples with a designation of "Visit 0" were utilized for visualization and statistical testing. *P*, by Fisher's exact test.

❖ Panel h (chronic inflammation):

- GSE205161:
  - Stacked barplots depict the distribution of SAS-1/MAS-1 profiles and dot-and-line plots depict median (IQR) of *TCF7* (NGE) by non-tuberculosis mycobacterial pulmonary disease status in a cystic fibrosis cohort. *P*, by Fisher's exact test.
- GSE3365:
  - Stacked barplots depict the distribution of SAS-1/MAS-1 profiles and dot-and-line plots depict median (IQR) of *TCF7* (NGE) by disease groups and age strata in the indicated study groups. *P*, by Fisher's exact test with 10,000 simulations.
- GSE145412:
  - Stacked barplots depict the distribution of SAS-1/MAS-1 profiles and dot-and-line plots depict median (IQR) of *TCF7* (NGE) by disease and body mass index (BMI) groups in the metabolic syndrome cohort. *P*, by Fisher's exact test.
- GSE45593:

- Stacked barplots depict the distribution of SAS-1/MAS-1 profiles and dot-and-line plots depict median (IQR) of *TCF7* (NGE) by tolerance criteria groups in the renal transplant tolerance cohort. *P*, by Fisher's exact test.

6.2.6. Figure S6

- Panels a-b:
  - Linear regression modeling of levels of the indicated gene signatures (z-score) and *TCF7* expression by age in the FHS stratified by (a) sex and (b) SAS-1/MAS-1 profiles. Shaded area depicts the 95% confidence bands. *P*, by linear regression testing for association with group, age, and an interaction term.

6.2.7. Figure S7

- Panels a-c:
  - (*left*) Boxplots depict median (IQR) levels of indicated gene signatures (z-scores) in the (a) FHS, (b) San Antonio Family Heart Study cohort (E-TABM-305; Mexican American), and (c) Brisbane cohort (GSE53195) stratified by age strata and SAS-1/MAS-1 profiles. *P*, by linear model with likelihood ratio test (LRT).
  - (*right*) Mean differences (β values) in expression levels of the indicated gene signatures in the (a) FHS, (b) San Antonio Family Heart Study cohort (E-TABM-305), and (c) Brisbane cohort (GSE53195) in persons stratified by the indicated age strata and SAS-1/MAS-1 profiles. *P*, by linear model with LRT.

6.2.8. Figure S8

- Panel:
  - Natural viral infection cohort (GSE68310).
  - Line plots depict expression (mean ± SEM) of indicated gene signatures (z-score) and *TCF7* (NGE) in persons classified as IR-degraders, IR-reconstituters, and IR-preservers. *P*, by ANOVA for comparing the expression in the three groups at last timepoint (spring).

6.2.9. Figure S9

- Panel a (GSE89403) and Panel b (GSE67589):
  - Line plots depict expression (mean ± SEM) of indicated gene signatures (z-score) and *TCF7* (NGE) over the indicated timepoints (weeks) in persons with

the indicated tuberculosis outcomes. *P* for group, determined by linear GEE adjusted for time. *P* for time, determined by linear GEE adjusted for indicated groups with ANOVA.

6.2.10. Figure S10

- Panel a:
    - Influenza challenge cohort (GSE54248)
    - Line plots depict expression (mean ± SEM) of the indicated gene signatures (z-score) and *TCF7* (NGE) over indicated timepoints (hours) by baseline SAS-1/MAS-1 profiles and symptom status. Note: data from two participants with asymptomatic L-H status were not included in the plot. *P* for group, determined by linear GEE comparing Asymptomatic H-L *vs.* Symptomatic L-H adjusted for time. *P* for time, determined by linear GEE adjusted for groups (Asymptomatic H-L vs Symptomatic L-H) with ANOVA.
- Panel b:
    - Typhoid challenge study (E-MTAB-3423)
    - Stacked barplots depict the distribution of SAS-1/MAS-1 profiles and dot-and-line plots depict median (IQR) of *TCF7* (NGE) over time stratified by disease status in the typhoid challenge study. *P*, by Fisher's exact test.

6.2.11. Figure S11

- Source: The CHI Consortium influenza vaccine cohort (GSE47353).
    - Panel a: Stacked barplots depict the distribution of SAS-1/MAS-1 profiles and dot-and-line plots depict median (IQR) of *TCF7* (NGE) by timepoint (days) and baseline SAS-1/MAS-1 profiles (at day -7). *P*, by χ^2 test for overall by time and by Fisher's exact test for SAS-1/MAS-1 profiles by time.
    - Panels b and c: Line plots depict expression (mean ± SEM) of the indicated gene signatures (z-score) and *TCF7* (NGE) over the indicated timepoints (days) in the (b) overall cohort and (c) by baseline SAS-1/MAS-1 profiles. *P*, by linear GEE with ANOVA for comparing overall across time (for panel b). *P*, by linear GEE with ANOVA for comparing baseline SAS-1/MAS-1 profiles adjusted for time and for time adjusted for SAS-1/MAS-1 profiles using two separate models (for panel c).

6.2.12. Figure S12

- Source: Emory trivalent influenza vaccine cohort during 2011/12 flu season (GE748
  - Panel a: Stacked barplots depict the distribution of SAS-1/MAS-1 profiles and dot-and-line plots depict median (IQR) of *TCF7* (NGE) by timepoint (days) and baseline SAS-1/MAS-1 profiles. *P*, by χ^2 test for overall by time and by Fisher's exact test for SAS-1/MAS-1 profiles by time.
  - Panels b and c: Line plots depict expression (mean ± SEM) of the indicated gene signatures (z-score) and *TCF7* (NGE) over the indicated timepoints (days) in the (b) overall cohort and (c) by baseline SAS-1/MAS-1 profiles. *P*, by linear GEE with ANOVA for comparing overall across time (for panel b). *P*, by linear GEE with ANOVA for comparing baseline SAS-1/MAS-1 profiles adjusted for time and for time adjusted for SAS-1/MAS-1 profiles using two separate models (for panel c).

6.2.13. Figure S13

- Source: Inactivated influenza vaccination in pregnant women cohort (GSE166545)
  - Panel a: Stacked barplots depict the distribution of SAS-1/MAS-1 profiles and dot-and-line plots depict median (IQR) of *TCF7* (NGE) by timepoint (days) and baseline SAS-1/MAS-1 profiles. *P*, by Fisher's exact test.
  - Panels b and c: Line plots depict expression (mean ± SEM) of the indicated gene signatures (z-score) and *TCF7* levels (NGE) over the indicated timepoints (days) in the (b) overall cohort and (c) by baseline SAS-1/MAS-1 profiles. *P*, by linear GEE with ANOVA for comparing overall across time (panel b). *P*, by linear GEE with ANOVA for comparing baseline SAS-1/MAS-1 profiles adjusted for time and for time adjusted for SAS-1/MAS-1 profiles using two separate models (panel c).

6.2.14. Figure S14

- H1N1 swine flu vaccination cohort (E-MTAB-2313)
  - Panel a: Stacked barplots depict distribution of SAS-1/MAS-1 profiles and dot-and-line plots depict median (IQR) of *TCF7* (NGE) by timepoint (days) and baseline SAS-1/MAS-1 profiles (at day 0). *P*, by Fisher's exact test.

- Panels b and c: Line plots depict expression (mean ± SEM) of the indicated gene signatures (z-score) and *TCF7* levels (NGE) over the indicated timepoints (days) in the (b) overall cohort and (c) by baseline SAS-1/MAS-1 profiles. *P*, by linear GEE with ANOVA for comparing overall across time (panel b). *P*, by linear GEE with ANOVA for comparing baseline SAS-1/MAS-1 profiles adjusted for time and for time adjusted for SAS-1/MAS-1 profiles using two separate models (panel c).

6.2.15. Figure S15

- Source: TIV fluzone influenza vaccine cohort during 2011/12 flu season (SDY296)
  - Panel a: Stacked barplots depict the distribution of SAS-1/MAS-1 profiles and dot-and-line plots depict median (IQR) of *TCF7* (NGE) by timepoint (days) and baseline SAS-1/MAS-1 profiles (at day −7). *P*, by Fisher's exact test.
  - Panels b and c: Line plots depict expression (mean ± SEM) of the indicated gene signatures (z-score) and *TCF7* levels (NGE) over the indicated timepoints (days) in the (b) overall cohort and (c) by baseline SAS-1/MAS-1 profiles. *P*, by linear GEE with ANOVA for comparing overall across time (panel b). *P*, by linear GEE with ANOVA for comparing baseline SAS-1/MAS-1 profiles adjusted for time and for time adjusted for SAS-1/MAS-1 profiles using two separate models (panel c).

6.2.16. Figure S16

- TIV influenza vaccination older cohort during 2010/11 flu season (SDY67)
  - Panel a: Stacked barplots depict the distribution of SAS-1/MAS-1 profiles and dot-and-line plots depict median (IQR) of *TCF7* (NGE) by timepoint (days), baseline SAS-1/MAS-1 profiles (at day 0), and IR groups [SAS-1^{high}/MAS-1^{low} participants were subdivided into four groups based on changes in SAS-1/MAS-1 profile post-vaccination: IR preservers (HL-HL-HL); IR reconstituters (HL-N-HL); Late IR-degraders (HL-HL-N); and Early IR degraders (HL-N-N)]. N, non-HL. *P*, by χ^2 test for overall by time and by Fisher's exact test for SAS-1/MAS-1 profiles and IR groups by time. *P* for inset at the bottom by Fisher's exact.

- Panels b to d: Line plots depict expression (mean ± SEM) of indicated gene signatures (z-score) and *TCF7* levels (NGE) over indicated timepoints (days) (b) overall and by (c) baseline SAS-1/MAS-1 profiles, and (d) IR groups. *P*, by linear GEE with ANOVA for comparing overall across time (panel b). *P*, by linear GEE with ANOVA for comparing baseline SAS-1/MAS-1 profiles adjusted for time and time adjusted for SAS-1/MAS-1 profiles using two separate models (panel c). *P*, determined by Welch's t-test for comparing gene signatures and by Wilcoxon rank-sum test for comparing *TCF7* expression between IR preservers (HL-HL-HL and HL-N-HL) *vs.* IR degraders (HL-HL-N and HL-N-N) at day 28 (panel d).

6.2.17. Figure S17

- Singapore Vaxigrip cohort (GSE107990)
  - Panel a: Stacked barplots depict the distribution of SAS-1/MAS-1 profiles and dot-and-line plots depict the median (IQR) of *TCF7* (NGE) by timepoint (days), baseline SAS-1/MAS-1 profiles (day 0), and IR groups [SAS-1^{high}/MAS-1^{low} participants were subdivided into four groups based on changes in SAS-1/MAS-1 profile post-vaccination: IR-preservers (HL-HL-HL); IR-reconstituters (HL-N-HL); Late IR-degraders (HL-HL-N); and Early IR-degraders (HL-N-N)]. N, non-HL. *P*, by χ^2 test for overall by time and by Fisher's exact test for SAS-1/MAS-1 profiles and IR groups by time.
  - Panels b to d: Line plots depict expression (mean ± SEM) of indicated gene signatures (z-score) and *TCF7* levels (NGE) over indicated timepoints (days) (b) overall and by (c) baseline SAS-1/MAS-1 profiles, and (d) IR groups. *P*, by linear GEE with ANOVA for comparing overall across time (panel b). *P*, by linear GEE with ANOVA for comparing baseline SAS-1/MAS-1 profiles adjusted for time and time adjusted for SAS-1/MAS-1 profiles using two separate models (panel c). *P*, determined by Welch's t-test for comparing z-scores and by Wilcoxon rank-sum test for comparing *TCF7* expression between IR preservers (HL-HL-HL and HL-N-HL) vs. IR degraders (HL-HL-N and HL-N-N) at day 28 (panel d).

6.2.18. Figure S18

- HIPC TIV influenza vaccine cohort during 2010/11 flu season (GSE74813)
  - Panel a: Stacked barplots depict the distribution of SAS-1/MAS-1 profiles and dot-and-line plots depict median (IQR) of *TCF7* (NGE) by timepoint (days), baseline SAS-1/MAS-1 profiles (at day 0), and IR groups [SAS-1^{high}/MAS-1^{low} participants were subdivided into four groups based on changes in SAS-1/MAS-1 profiles: IR-preservers (HL-HL-HL); IR-reconstituters (HL-N-HL); Late IR-degraders (HL-HL-N); and Early IR degraders (HL-N-N)]. N, non-H-L. *P*, by χ^2 test for overall by time and by Fisher's exact test for SAS-1/MAS-1 profiles and IR groups by time.
  - Panels b to d: Line plots depict expression (mean ± SEM) of indicated gene signatures (z-score) and *TCF7* levels (NGE) over the indicated timepoints (days) (b) overall and by baseline SAS-1/MAS-1 profiles, and (d) IR groups. *P*, by linear GEE with ANOVA for comparing overall across time (panel b). *P*, by linear GEE with ANOVA for comparing baseline SAS-1/MAS-1 profiles adjusted for time and time adjusted for SAS-1/MAS-1 profiles using two separate models (panel c). *P*, by linear GEE with ANOVA for comparing IR-preservers (HL-HL-HL and HL-N-HL) vs*.* IR- degraders (HL-HL-N & HL-N-N) adjusted for time and time adjusted for IR groups (IR-preservers vs. IR-degraders) using two separate models (panel d).

6.2.19. Figure S19

- TIV influenza vaccination female cohort (GSE48023)
  - Panel a: Stacked barplots depict the distribution of SAS-1/MAS-1 profiles and dot-and-line plots depict median (IQR) of *TCF7* (NGE) by timepoint (days), baseline SAS-1/MAS-1 profiles (at day 0), and IR groups [SAS-1^{high}/MAS-1^{low} participants were subdivided into four groups based on changes in SAS-1/MAS-1 profiles: IR-preservers (HL-HL-HL); IR-reconstituters (HL-N-HL); Late IR-degraders (HL-HL-N); and Early IR degraders (HL-N-N)]. N, non-HL. *P*, by χ^2 test for overall by time and by Fisher's exact test for SAS-1/MAS-1 profiles and IR groups by time.
  - Panels b to d: Line plots depict expression (mean ± SEM) of indicated gene signatures (z-score) and *TCF7* levels (NGE) over the indicated timepoints

(days) (b) overall and by (c) baseline SAS-1/MAS-1 profiles, and (d) IR groups. *P*, by linear GEE with ANOVA for comparing overall across time (panel b). *P*, by linear GEE with ANOVA for comparing baseline SAS-1/MAS-1 profiles adjusted for time and time adjusted for SAS-1/MAS-1 profiles using two separate models (panel c). *P*, by linear GEE with ANOVA for comparing all IR groups [IR-preservers (HL-HL-HL); IR-reconstituters (HL-N-HL); Late IR-degraders (HL-HL-N); and Early IR-degraders (HL-N-N)] adjusted for time and time adjusted for all IR groups using two separate models) (panel d).

6.2.20. Figure S20

- TIV influenza vaccination male cohort (GSE48018)
  - Panel a: Stacked barplots depict the distribution of SAS-1/MAS-1 profiles and dot-and-line plots depict median (IQR) of *TCF7* (NGE) by timepoint (days), baseline SAS-1/MAS-1 profiles (at day 0), and IR groups [SAS-1^{high}/MAS-1^{low} participants were subdivided into four groups based on changes in SAS-1/MAS-1 profiles: IR-preservers (HL-HL-HL); IR-reconstituters (HL-N-HL); Late IR-degraders (HL-HL-N); and Early IR degraders (HL-N-N)]. N, non-HL. *P*, by χ^2 test for overall by time and by Fisher's exact test for SAS-1/MAS-1 groups and IR groups by time.
  - Panels b to d: Line plots depict expression (mean ± SEM) of indicated gene signatures (z-score) and *TCF7* levels (NGE) over the indicated timepoints (days) (b) overall and by (c) baseline SAS-1/MAS-1 profiles, and (d) IR groups. *P*, by linear GEE with ANOVA for comparing overall across time (panel b). *P*, by linear GEE with ANOVA for comparing baseline SAS-1/MAS-1 profiles adjusted for time and time adjusted for SAS-1/MAS-1 profiles using two separate models (panel c). *P*, by linear GEE with ANOVA for comparing all IR groups [IR-preservers (HL-HL-HL); IR-reconstituters (HL-N-HL); Late IR-degraders (HL-HL-N); and Early IR-degraders (HL-N-N)] adjusted for time and time adjusted for all IR groups using two separate models (panel d).

6.2.21. Figure S21

- Stanford SARS-CoV-2 vaccine cohort (GSE169159)
- Panel a:

  - Schema; no statistical testing was performed.
- Panel b:
  - Stacked barplots depict the distribution of SAS-1/MAS-1 profiles and dot-and-line plots depict median (IQR) of *TCF7* (NGE) by timepoint (days) and baseline SAS-1/MAS-1 profiles (at day 0). *P*, by Fisher's exact test.
- Panels c-d:
  - Line plots depict expression (mean ± SEM) of gene signatures (z-score) and *TCF7* levels (NGE) over the indicated timepoints (days) by baseline SAS-1/MAS-1 profiles. *P*, by linear GEE with ANOVA for comparing baseline SAS-1/MAS-1 profiles adjusted for time and time adjusted for SAS-1/MAS-1 profiles using two separate models.

6.2.22. Figure S22

- RSV children cohort (GSE188427)
  - Panel a: Line plots depict expression (mean ± SEM) of gene signatures (z-score) and *TCF7* levels (NGE) over indicated timepoints (days) in children with RSV vs. controls. *P*, determined by linear GEE with ANOVA comparing RSV group over time.
  - Panel b: Stacked barplots depict the distribution of SAS-1/MAS-1 profiles and dot-and-line plots depict median (IQR) of *TCF7* (NGE) by timepoint (days) and study groups. *P*, by χ^2 test (*left*) and Fisher's exact test (*right*).

6.2.23. Figure S23

- STEMI heart failure cohort (GSE59867)
  - Line plots depict expression (mean ± SEM) of gene signatures (z-scores) and *TCF7* levels (NGE) over the indicated timepoints. *P*, by linear GEE with ANOVA for comparing baseline SAS-1/MAS-1 profiles (at admission) adjusted for time and time adjusted for SAS-1/MAS-1 profiles using two separate models.

6.2.24. Figure S24

- Vitality 90+ study (GSE65218)
  - Boxplots depict median (IQR) expression of gene signatures (z-scores) and *TCF7* levels (NGE) by SAS-1/MAS-1 profiles. *P*, by ANOVA for gene signatures and by Kruskal-Wallis test for *TCF7*.

6.2.25. Figure S25

- Panel:
  - Kaplan-Meier plot depicts time to CVD diagnosis by quartiles of Age_IL6^{up} gene signature in the FHS. *P*, by Cox proportional hazards models adjusted for age, sex, smoking status, diabetes, hypertension, and total cholesterol with likelihood ratio test (LRT).

6.2.26. Figure S26

- Panel a:
  - Schema; no statistical analysis was performed.
- Panel b:
  - IHG distributions and median age (IQR) for each cohort.

6.2.27. Figure S27

- Panel a:
  - Barplots depict distribution of IHG with subgrades by sex, age strata, and age strata within sex in four cohort subsets: *Left top:* SAIL cohort; *Left bottom*: overall ac-COVID-19 cohort (VA-CLC); *Right top*: All post-acute samples; and *Right bottom*: samples obtained at baseline (VA-CLC) paired with the post-acute samples. Dashed lines indicate percent of IHG-I in younger females and older males. *P*, by χ^2 and Fisher's exact test
- Panel b:
  - Barplots depict the distribution of convalescent (post-acute) IHG by the IHG at presentation (baseline) with ac-COVID-19. *P*, by Fisher's exact test.
- Panel c:
  - Barplots depict the distribution of the IHG at baseline (i.e., presentation with ac-COVID-19) and post-acute COVID-19 according to the IHG pre-COVID-19. *P*, by Fisher's exact test.

6.2.28. Figure S28

- Panel a:
  - Kaplan-Meier plots depict time to achieve (*top*) IHG-I or IIa and (*bottom*) IHG-I within 30 days from presentation with ac-COVID-19 stratified by the baseline IHG. *P*, by age adjusted Cox proportional hazards models.

- Panel b:
  - Odds ratios with 95% CIs of post-acute IHG-I based on the baseline IHG with subgrades. Analysis was performed using an unadjusted logistic regression model. Two-sided statistical tests were used.

6.2.29. Figure S29

- Cohort: ac-COVID-19 VA-CLC
  - Line plots depict linear GEE models with pointwise 95% confidence bands of the change in expression levels of SAS-1 and MAS-1 gene signatures during ac-COVID-19 since baseline (0 to 7 days), by (*top*) COVID-19 outcomes and (*bottom*) baseline IHG. Expression data within 7 days from IHG baseline of 45 participants were plotted by baseline IHG and COVID-19 outcomes by 30-day survival status. IHG baseline is the date of the first available IHG for each participant. Note: There were 3 non-hospitalized individuals with no RNA-Seq measurements within this time window. *P*, by linear GEE with an AR(1) correlation structure testing for interaction of groups and time.

6.2.30. Figure S30

- Panel a:
  - (*Top*) Distribution of IHG in the ac-COVID-19 VA-CLC stratified by hospitalization status and age strata.
  - (*Bottom*) Odds ratios of IHG-I (blue) and IHG-II (orange) in the ac-COVID-19 cohort stratified by hospitalization status and age strata. *P*, by logistic regression.
- Panel b:
  - Point estimates with 95% CI of hazard ratios of mortality in the VA-CLC for acute mortality (30-day mortality) and post-acute mortality (24-month mortality). Analysis performed using Cox proportional hazards models adjusted for sex.
- Panel c:
  - Probability of 30-day mortality with age stratified by baseline IHG with subgrades. *P*, by logistic regression.
- Panel d:

    - Absolute risk (AR), relative risk (RR), population attributable fraction (PAF), and adjusted PAF (aPAF) values of 30-day mortality. Analysis for aPAF (for the overall analysis) was performed using logistic regression for IHG adjusted for age and age adjusted for IHG. No *P* values were determined.
- Panel e:
    - Kaplan-Meier plots depict 60-day survival from baseline ac-COVID-19 stratified by IHG with subgrades in (left) subset prior to October 1, 2020, and (*right*) subset on or after October 1, 2020. *P*, by Cox proportional hazards model with likelihood ratio test (LRT).
- Panel f:
    - Rate ratios (RR) with 95% CI of serious non-ARDS complications score (SCS) by age, sex, and baseline IHG with subgrades. Stratifications and adjustments are indicated along y-axes. *P,* by quasi-Poisson GLM.

6.2.31. Figure S31

- Panel a:
    - Kaplan-Meier plot of 30-day survival stratified by SARS-CoV-2 RT-qPCR cycle threshold (Ct ≥30 or <30. *P*, by age-adjusted Cox proportional hazards model with likelihood ratio test (LRT).
- Panel b:
    - Boxplots depict median (IQR) of the indicated biomarkers by Ct ≥30 or <30. *P*, by linear model with LRT.
- Panel c:
    - Odds with 95% confidence bands of SARS-CoV-2 RT-qPCR cycle threshold (Ct) ≥30 with age by IHG bins (IHG-I vs. rest). *P*, by logistic regression.
- Panel d:
    - Adjusted odds ratios of SARS-CoV-2 RT-qPCR cycle threshold (Ct) ≥30 by IHG with subgrades. *P*, by logistic regression adjusted for age and days between baseline and Ct measurement.
- Panel e:
    - Odds ratios with 95% CI of post-acute condition (ICD-10-CM U09.9) by age strata and sex. *P*, by univariate logistic regression models.

- Panel f:
  - Adjusted odds ratios with 95% CI of post-acute condition (ICD-10-CM U09.9) by baseline IHG with subgrades. *P*, by logistic regression adjusted for age and sex.

6.2.32. Figure S32

- Panel a:
  - Kaplan-Meier plots depict time to survival in the acute mortality (*left*) and post-acute mortality (*right*) windows stratified by improvement in IR status. Improvement determined within the first 5 days of ac-COVID-19. *P*, by Cox proportional hazards model adjusted by age and/or number of IHG measures with LRT.
- Panel b:
  - Kaplan-Meier plots depict time to survival in the acute mortality (*left*) and post-acute mortality (*right*) windows stratified by improvement in IR status overall and within age strata. Improvement determined within the first 5 days of ac-COVID-19. *P*, by Cox proportional hazards models adjusted for age and/or number of IHG measurements within the first 5 days of ac-COVID-19.

6.2.33. Figure S33

- Panel a: ac-COVID-19 cohort (VA-CLC)
  - Odds ratio with 95% CI of sVNT %inhibition of <30% by (*left to right*) age ≥60 vs. <60 years, sex, and days since onset of symptoms (DoS) ≤7 vs. >7. *P*, by logistic regression.
- Panel b: ac-COVID-19 cohort (VA-CLC)
  - Age and DoS-adjusted odds ratio with 95% CI of %inhibition of <30% by (*left to right*) baseline IHG, baseline IHG with subgrades, and indicated COVID-19 outcomes. *P*, by logistic regression.
- Panel c: ac-COVID-19 outbreak cohort (subset of VA-CLC)
  - Line plots depict the odds with 95% pointwise confidence band of %inhibition of ≥30% *vs.* time by baseline IHG. Modeling was performed using a logistic GEE model with an AR(1) correlation structure as a quadratic function of days from baseline (COVID-19 diagnosis) by IHG.

- Panel d: SAIL cohort – pre-booster subset
  - Line plots depict the odds with 95% confidence bands of %inhibition of ≥30% *vs.* time by IHG, adjusted for age and previous COVID-19 infection status. Modeling was performed using logistic regression.
- Panel e: SAIL cohort – pre-booster subset
  - Odds ratio with 95% CI of sVNT %inhibition of <30% by (*left to right*) baseline IHG, previous COVID-19 infection status, and age strata. *P*, by logistic regression adjusting for days since last vaccination.

6.2.34. Figure S34

- Source: TIV fluzone influenza vaccine cohort during 2011/12 flu season (SDY296)
  - Panel a: Stacked barplots depict the distribution of SAS-1/MAS-1 profiles and dot-and-line plots depict median (IQR) of *TCF7* (NGE) by timepoint (days) and pre-vaccination IHG status. *P*, by Fisher's exact test.
  - Panel b: Line plots depict expression (mean ± SEM) of the indicated gene signatures (z-score) and *TCF7* levels (NGE) over the indicated timepoints (days) by pre-vaccination IHG status. *P*, by linear GEE with ANOVA for comparing baseline IHG groups (IHG-I vs. IHG-IIa) adjusted for time and for time adjusted for IHG groups using two separate models.

6.2.35. Figure S35

- Panel a:
  - Rate ratio with 95% CI of average comorbidity burden (comorbidities per person) by baseline IHGs among persons in the VA-CLC stratified by strata. *P*, by quasi-Poisson GLM.
- Panel b:
  - Left, schema and no statistical testing was performed.
  - Adjusted comorbidity rates with 95% CI of all 19 recorded conditions and a subset of 8 conditions (associated with post-acute mortality) by baseline IHG with subgrades. Modeling was performed using quasi-Poisson GLM model adjusted for age. To calculate the rates, an age had to be specified and the median age (62 y) was used.
- Panel c:

- Line plots depict the percentage of individuals expected to have 1 or no comorbidities by age and baseline IHG with 95% confidence bands. *P*, by logistic regression.

6.2.36. Figure S36

- Source: ac-COVID-19 cohort (VA-CLC)
    - Boxplots depict median (IQR) expression of indicated gene signatures (z-scores) and *TCF7* levels (NGE) by IHG groupings (rest, IHG-II through IHG-IV pooled), baseline IHG with subgrades, SAS-1/MAS-1 profiles, and COVID-19 outcomes. Expression data from RNA-Seq baseline of 48 participants were used to generate the plots and determine the SAS-1/MAS-1 profiles. RNA-Seq baseline is the date of the first available RNA-Seq data for each participant. COVID-19 outcomes were determined by 30-day survival status. *P,* by linear model with likelihood ratio test (LRT).

6.2.37. Figure S37

- Panel a:
    - Line plots depict the levels of the indicated biomarkers with 95% confidence bands across age stratified by baseline IHG groups. *P* for age and IHG, determined by linear regression.
- Panel b:
    - Line plots depict the levels of the biomarkers with 95% confidence bands across age by baseline IHG bins (IHG-I vs IHG-IIc/IVc). Ct, SARS-CoV-2 qRT-PCR cycle threshold values. The model with Ct was adjusted for the days between baseline and when the Ct measurement was taken. Probability of %inhibition < 30% represents the lower SARS-CoV-2 nAb response. *P*, by linear regression model or logistic regression model (for probability of %inhibition).

6.2.38. Figure S38

- Panel a:
    - Boxplots depict median (IQR) of the indicated biomarkers by baseline IHG status, outcomes, %inhibition, age strata, and sex. *P*, by linear model with likelihood ratio test (LRT).

- Panel b:
  - Line plots depict the modeled data with 95% pointwise confidence bands of indicated log_2-transformed immune traits and log_2-transformed cytokines by (*left to right*) baseline IHG status, baseline IHG with subgrades, COVID-19 outcomes, and age strata over the first 7 days from baseline. Analysis was performed using a linear GEE with an AR(1) correlation structure with ANOVA by time, predictor (IHG, COVID-19 outcomes, or age strata), and interaction term between time and predictor.

6.2.39. Figure S39

- Source: ac-COVID-19 cohort (VA-CLC)
  - Age-adjusted mean values with 95% CI of indicated log_2-transformed biomarker levels by baseline IHG and comorbidity score strata. *P*, by linear regression with contrasts.

6.2.40. Figure S40

- Source: ac-COVID-19 cohort (VA-CLC)
  - Age-adjusted mean values with 95% CI of indicated log_2-transformed biomarker levels by obesity status overall and within IHGs. *P*, by linear regression with contrasts.

6.2.41. Figure S41

- Panel a:
  - Kaplan-Meier plots depict 30-day mortality stratified by IHG grouping and (*left*) comorbidity burden strata and (*right*) IL-6 (low/high strata, determined by median).
  - Adjusted *P* (aP) values determined with age-adjusted Cox proportional hazards model for the indicated comparisons.
- Panel b:
  - Cox proportional hazards model of 30-day mortality with indicated variables, reporting adjusted hazard ratio, 95% CI, and *P* values.

6.2.42. Figure S42

- Source: ac-COVID-19 cohort (VA-CLC)

  - *Left*: Unsupervised hierarchical clustering heatmap of Pearson's correlation coefficient values of indicated biomarkers with gene signatures using Euclidean distances and Ward's D linkage.
  - *Right*: Supervised clustering heatmap of mean levels of indicated biomarkers within IHGs and outcomes. The color code corresponds to the z-score transformed mean values.

6.2.43. Figure S43

- Source: ac-COVID-19 cohort (VA-CLC)
  - Boxplots depict median (IQR) of the indicated $\log_2$-transformed immune traits and $\log_2$-transformed cytokines by (*left to right*) the indicated IHGs, SAS-1/MAS-1 profiles, and COVID-19 outcomes. *P,* by linear regression model with likelihood ratio test (LRT).

6.2.44. Figure S44

- Panel a:
  - Stacked barplots depict the distribution of SAS-1/MAS-1 profiles by octiles of the indicated transcription factor expression levels (NGE) in the FHS. *P*, by χ^2 test.
- Panel b:
  - Linear regression modeling of the indicated transcription factor genes (NGE) with 95% confidence bands by age and sex in the FHS. *P* value determined by linear regression testing for an association of expression of the indicated genes with sex, age, and the interaction term.
- Panel c:
  - Hazard ratios of all-cause mortality by the indicated transcription factor genes in the FHS. Analysis was performed using Cox proportional hazards models adjusted for age and sex. FDR correction was performed using the Benjamini-Hochberg method.
- Panel d:
  - Boxplots depict median (IQR) of the indicated transcription factor genes (NGE) by IHG groups in the VA-CLC (top) and by survival status in the NYU-COVID-

19 cohort (*bottom*). *P,* by linear model with a likelihood ratio test in the VA-CLC cohort and by Wilcoxon rank-sum test in the NYU-COVID-19 cohort.

6.2.45. Figure S45

- Source: Alzheimer's disease cohort (GSE140829)
  - Panels a-b: Linear regression modeling of the expression of the indicated gene signatures (z-score) and *TCF7* levels (NGE) by age and (a) disease status, and (b) SAS-1/MAS-1 profiles in persons with Alzheimer's disease. Shaded area indicates 95% confidence bands. *P* by linear regression testing for association with group, age, and the interaction term.

6.2.46. Figure S46

- Source: VA-CLC
  - Modeling line plots of the indicated biomarkers longitudinally (days from baseline) by IR-preserver (maintained IHG-I) and extreme IR-degraders who improved their IHG status (IHG-IIc/IVc to better IHG) in the VA-CLC. Shaded area depicts the 95% confidence bands. Analysis was performed using a linear GEE model with an autoregressive 1 correlation structure. The model includes days from baseline (0 to 14), IHG-I vs IHG-IIc/IVc, and the interaction term between the two to test whether the slopes over time differed by IHG-I vs. IHG-IIc/IVc. A generalized linear hypothesis test was used to compare IHG-I vs. IHG-IIc/IVc at days 0 and 14.

6.2.47. Figure S47

- Source: MID cohort
  - Boxplots depict median (IQR) levels of indicated proteomic biomarker expression levels stratified by SAS-1/MAS-1 profiles. *P*, determined by linear regression with ANOVA. Analysis and derivation outlined in Section 4.10.6.

6.2.48. Figure S48

- Source: IBD vedolizumab cohort (GSE191328)
  - Line plots depict expression (mean ± SEM) of the indicated gene signatures (z-scores) and *TCF7* levels (NGE) over the indicated timepoints. *P*, by linear GEE with ANOVA for comparing baseline SAS-1/MAS-1 profiles adjusting for time and time adjusting for SAS-1/MAS-1 profiles using two separate models.

6.2.49. Figure S49

- Source: Etanercept cohort (GSE36177)
    - Panel a: Line plots depict expression (mean ± SEM) of indicated gene signatures (z-score) and *TCF7* levels (NGE) by treatment groups over the indicated timepoints (hours). *P*, by Welch's t-test for comparison of z-scores and by Wilcoxon rank-sum test for comparison of *TCF7* expression between the treatment groups at 4h time point.
    - Panel b: Stacked barplots depict the distribution of SAS-1/MAS-1 profiles and dot-and-line plots depict median (IQR) of *TCF7* (NGE) by timepoint (hours) and treatment groups. *P*, by Fisher's exact test.

6.2.50. Figure S50

- Source: Framingham Heart Study (FHS)
- Panel a:
    - Unsupervised hierarchical clustering of correlation heatmap of indicated genes, and age with clustering performed using 1 – Pearson's correlation coefficient as the distance with Ward's D linkage. Color gradient corresponding to correlation coefficient.
- Panel b:
    - Correlation plots of age, the indicated genes in the FHS with unsupervised hierarchical clustering performed using 1 – Pearson's correlation coefficient as the distance with Ward's D linkage. Correlation values are presented with color gradients and pie charts.
- Panels c-d:
    - Linear regression modeling of expression levels of the indicated genes by age in the FHS stratified by (c) sex and (d) SAS-1/MAS-1 profiles. Shaded area depicts the 95% confidence bands. *P*, by linear regression testing for association with group, age, and an interaction term.
- Panel e:
    - Hazard ratios (with 95% CI) of mortality in the FHS for the indicated genes. Analysis performed using Cox proportional hazards models adjusting for age and sex.

7. Supplementary Note: Rationale for derivation of IHGs.

Based on principles discussed previously (Ahuja et al., 2023; Lee et al., 2021), we derived IHGs I to IV as metrics of IR (Figure 6b and Figure S26b). As reported previously, the IHGs were generated by co-indexing the peripheral blood CD4:CD8 T-cell ratio and $CD4^+$ counts and they signify distinct tiers and forms of CD8-CD4 balance/imbalance (Ahuja et al., 2023; Lee et al., 2021) (Figure 6b and Figure S26b). In prior reports, we outlined why the CD8-CD4 balance/imbalance derived by this co-indexing strategy is a superior indicator of immunocompetence vs. single measures of the $CD4^+$ and $CD8^+$ count or the CD4:CD8 ratio (Ahuja et al., 2023; Lee et al., 2021). Additional justifications for the derivation and use of IHGs are as follows.

The CD4:CD8 T-cell ratio correlates negatively with $CD8^+$ T-cell counts and positively with $CD4^+$ T-cell counts (**Note Figure panel a**). However, the extent to which the CD4:CD8 T-cell ratio explained the variability in $CD4^+$ and $CD8^+$ counts and median levels of $CD4^+$ and $CD8^+$ counts differed extensively among cohorts and by age (**Note tables a and b, below**). For example, the baseline ratio values explained 5% and 26% of the variability in baseline $CD4^+$ and $CD8^+$ T-cell counts, respectively, in the VA-CLC (r^2 in **Note Figure panel a**); the corresponding proportions were 10% and 52%, respectively, in the SAIL cohort (**Note table a**). In the n=3898 person aging cohort (SardiNIA (Ahuja et al., 2023; Lee et al., 2021)), the ratio explained approximately 11% of the variability in $CD4^+$ counts in three age groups (<40, 40-59, and ≥60 years), whereas the ratio explained 37%, 48%, and 61%, respectively, of the variability in $CD8^+$ counts in these groups (**Note table b**). Thus, the extensive interindividual differences in the balance between $CD8^+$ and $CD4^+$ T-cell counts across ages and conditions is not adequately captured by assessments of the ratio or $CD8^+$ and $CD4^+$ counts alone. To mitigate this confounder, IHGs were derived to serve as a uniform metric that describes this balance independent of age, sex, or underlying disease/conditions.

IHG features. We used a CD4:CD8 ratio <1.0 as an indicator of disproportionately higher $CD8^+$ counts relative to whether individuals have $CD4^+$ counts higher than the average in healthy adults (800 CD4-cells/mm^3) (Le et al., 2013; Okulicz et al., 2015) (**Note Figure a; Note table c**). Hence, IHG-I and IHG-II track relatively lower $CD8^+$ T-cell levels with higher (IHG-I) or lower (IHG-II) $CD4^+$ counts (**Note Figure a; Note table c**). In contrast, IHG-III and IHG-IV track relatively higher $CD8^+$ T-cell levels with higher (IHG-III) or lower (IHG-IV) $CD4^+$ counts (**Note Figure a; Note table c**). To further monitor immunosuppression, we derived subgrades a, b, and c of IHG-II and IHG-IV (Figure S26b), which track ≥500 to <800, >200 to <500, and ≤200 $CD4^+$ T-cells/mm^3, respectively (Ahuja et al., 2023; Lee et al., 2021). ≤200 $CD4^+$ T-cells/mm^3 signifies AIDS in HIV+ persons, and 500 cells/mm^3 is the median $CD4^+$ during early HIV infection (Le et al., 2013; Okulicz et al., 2015). 500 CD4-cells/mm^3 is a clinically meaningful threshold of immunocompetence, as initiation of antiretroviral therapy below this $CD4^+$ threshold is associated with an increased risk of developing AIDS in HIV+ persons (INSIGHT START Study Group et al., 2015).

IHGs mitigate confounded assessments of immune status. Five examples illustrate that the IHGs are distinct from strata of the ratio and $CD4^+$ or $CD8^+$ count as well as align with distinct features of aging and viral infections. First, IHGs with similar $CD4^+$ strata track distinct relative proportions of $CD4^+$ and $CD8^+$ T-cells (**Note Figure a**; **tables below)**: IHG-I ($CD8^{lower}$-$CD4^{higher}$), IHG-II ($CD8^{lower}$-$CD4^{lower}$), IHG-III ($CD8^{higher}$-$CD4^{higher}$), and IHG-IV ($CD8^{higher}$-$CD4^{lower}$) (**Note Figure a; Note table c**) (Ahuja et al., 2023; Lee et al., 2021). Hence, IHG-I and IHG-II track CD8-CD4 balance, i.e., restrained $CD8^+$ T-cell expansion with higher or lower CD4+ counts, respectively. Conversely, IHG-III and IHG-IV track CD8-CD4 imbalance, i.e., unrestrained $CD8^+$ T-cell expansion with higher or lower $CD4^+$ counts, respectively. Thus, while IHG-I and IHG-III both track $CD4^{higher}$ (≥800 cells/mm^3), and both IHG-II and IHG-IV track $CD4^{lower}$ (<800 cells/mm^3), yet these IHG sets track distinct CD8-CD4 balances/imbalances.

Second, IHGs with similar $CD4^+$ strata may associate with distinct pathophysiologic contexts. For example, IHG-IIa and IHG-IVa track similar $CD4^+$ counts (≥500 to <800 cells/mm^3; **Figure S26b**; **Note table c**). Yet, IHG-IIa is the second-most prevalent grade

in HIV-seronegative individuals (Figure S26b). In contrast, IHG-IVa emerges with advanced age, after exposure to HIV (e.g., HIV-seronegative female sex workers), and in HIV+ persons (Ahuja et al., 2023; Lee et al., 2021) (Figure S26b). IHG-I is the most prevalent grade in HIV-seronegative reference populations (Figure S26b). In contrast, while IHG-III is infrequent (e.g., 2% in the overall SardiNIA aging cohort), its prevalence increases with age (Ahuja et al., 2023; Lee et al., 2021), following exposure to HIV (Figure S26b), and during HIV antiretroviral therapy (2.7% before versus 23.2% during therapy; Figure S26b).

Third, $CD4^+$ lymphopenia, a hallmark feature of both ac-COVID-19 and HIV infection, occurs in the context of distinct CD8-CD4 balances (Figure S26b). The $CD4^+$ lymphopenia of ac-COVID-19 occurs in the context of $CD8^{lower}$-$CD4^{lower}$ (IHG-IIb/c) or $CD8^{higher}$-$CD4^{lower}$ (IHG-IVb/c) (Figure S26b) (Ahuja et al., 2023; Lee et al., 2021). In contrast, the $CD4^+$ lymphopenia of HIV occurs exclusively with $CD8^{higher}$-$CD4^{lower}$ (IHG-IVa/b/c) (Figure S26b) (Ahuja et al., 2023). Hence, while both IHG-IIc and IHG-IVc track ≤200 CD4-cells/mm^3, IHG-IVc signifies classic AIDS in HIV+ individuals ("Panel on Antiretroviral Guidelines for Adults and Adolescents. Guidelines for the Use of Antiretroviral Agents in Adults and Adolescents with HIV. Department of Health and Human Services.
http://www.aidsinfo.nih.gov/ContentFiles/AdultandAdolescentGL.pdf. Accessed July, 26 2019); IHG-IIc is not a feature of HIV disease), whereas IHG-IIc or IHG-IVc signifies an acute AIDS-like immunodeficiency during ac-COVID-19 (Figure S26b). In the ac-COVID-19 cohort, IHG-IIc (13.1%) was 2.5 times more common than IHG-IVc (5.2%) (Figure S26b). In the VA-CLC, ac-COVID-19 patients presenting with IHG-IVc vs. IHG-IIc tend to be older and have a higher comorbidity burden (Table S5a).

Fourth, while a $CD4^+$ T-cell count from 500 to 1,600 cells/mm^3 is viewed as “normal” (HIV.gov), this range conflates four IHGs (IHG-I, IHG-IIa, IHG-III, and IHG-IVa) with contrasting distribution patterns in reference populations (**Note Figure a**). Furthermore, while lymphopenia is a well-documented feature of ac-COVID-19 (L. Tan et al., 2020), total lymphocyte count strata conflate varying proportions of IHGs.

Fifth, use of the CD4:CD8 ratio as a metric of immunocompetence is also potentially confounded. IHG-IVc signifies the triad ≤200 CD4-cells/mm^3 and higher CD8$^+$ counts and therefore an inverted CD4:CD8 ratio; in contrast, IHG-IIc signifies the triad ≤200 CD4-cells/mm^3 but with lower CD8$^+$ counts and therefore a ratio value greater than unity. Yet, both IHG-IIc and IHG-IVc associate with increased 30-day mortality hazards during ac-COVID-19 [(Lee et al., 2021) and findings reported herein]. Thus, while a high ratio value has been reported to associate with severe ac-COVID-19 (De Zuani et al., 2022), we found that both a high and low ratio value may associate with worse outcomes. Furthermore, during ac-COVID-19, levels of total lymphocyte counts as well as B and NK cells were closely aligned with the IHGs, but they did not correlate with the CD4:CD8 ratio (**Note Figure b-c**). Collectively, these five examples underscore that lymphocyte counts or the ratio are not a proxy for the IHGs; instead IHGs track distinctive CD8-CD4 balances/imbalances as well as levels of lymphocytes, B and NK cells. Findings described herein provide additional examples wherein use of the CD4$^+$ count strata and ratio for risk stratification rather than the IHGs would have obscured the disease and mechanistic correlates of ac-COVID-19.

Supplementary Note Figure

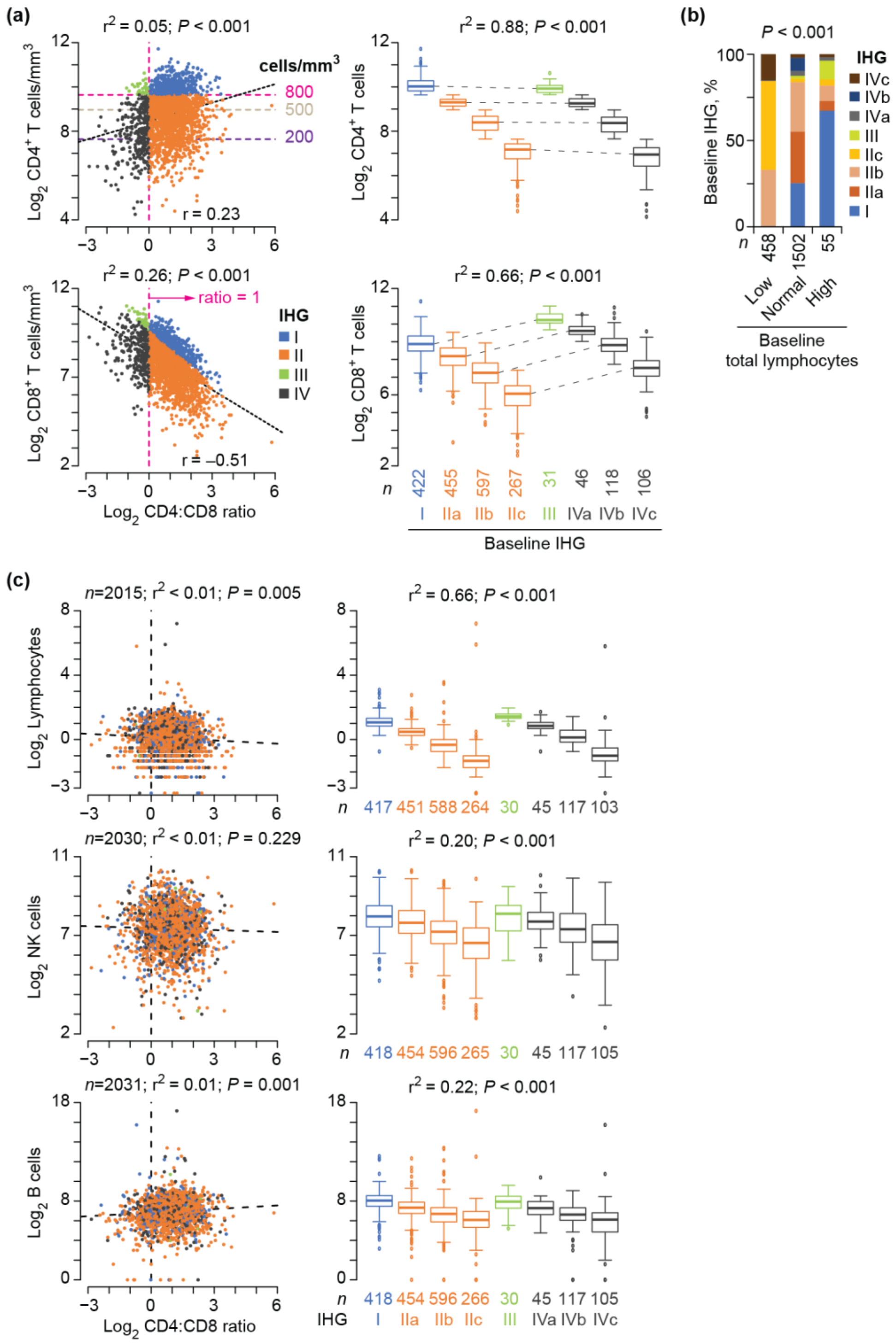

Note figure. (a) (*left*) Scatterplots of peripheral blood CD4⁺ (*top*) and CD8+ (*bottom*) T-cells with CD4:CD8 T-cell ratio at presentation with ac-COVID-19. Colored dots correspond to IHGs: blue, IHG-I; orange, IHG-II; green, IHG-III; black, IHG-IV. (*right*) Boxplots depict median (IQR) of $CD4^+$ counts and $CD8^+$ counts according to the IHG at presentation with ac-COVID-19. Dashed lines connect medians of $CD4^+$ and $CD8^+$ T-cell counts across IHGs. r^2, by linear regression. *P*, by linear regression with LRT. **(b)** Distribution of

IHGs according to baseline total lymphocyte count strata (low, <0.7; normal, 0.7-3.1; high, ≥3.1 x10^3 cells/µL). *P*, by χ^2 test. **(c)** Scatterplots (*left*) and boxplots (*right*) depict median (IQR) of indicated peripheral blood biomarkers at presentation with ac-COVID-19 by baseline IHG. Dashed lines indicating regression line of best fit. r^2, by linear regression. *P*, by linear regression with LRT.

Note table a. Correlation of $CD4^+$ and $CD8^+$ T-cell counts with the CD4:CD8 ratio in cohorts with or without persons with HIV seropositivity.

	Median (IQR)			$CD4^+$ and Ratio		$CD8^+$ and Ratio	
**Cohort**	**$CD4^+$**	**$CD8^+$**	**CD4:CD8**	**r^2**	***P***	**r^2**	***P***
SAIL	858 (666-1086)	449 (308-616)	1.92 (1.42-2.66)	0.10	0.000	0.52	0.000
Acute VA-CLC	462 (247-748)	241 (125-417)	1.96 (1.29-2.80)	0.05	0.000	0.26	0.000
Post-acute VA-CLC	791 (596-1024)	412 (284-588)	1.90 (1.35-2.80)	0.11	0.000	0.54	0.000
SardiNIA	1023 (798-1280)	462 (337-634)	2.22 (1.66-2.93)	0.09	0.000	0.51	0.000
UCSD	917 (740-1128)	514 (376-673)	1.78 (1.41-2.35)	0.08	0.000	0.49	0.000
Female Sex Workers	986 (785-1201)	638 (487-893)	1.50 (1.13-2.02)	0.17	0.000	0.17	0.000
HIV+ Entry	454 (329-609)	869 (623-1173)	0.53 (0.37-0.77)	0.47	0.000	0.25	0.000
HIV+ Pre-ART	360 (273-469)	882 (616-1219)	0.40 (0.28-0.57)	0.41	0.000	0.26	0.000
HIV+ ART	812 (637-1035)	884 (662-1167)	0.94 (0.68-1.24)	0.24	0.000	0.41	0.000

r^2 presented from correlation of indicated value with CD4:CD8 ratio. ART, anti-retroviral therapy; HIV, human immunodeficiency virus; IHG, immune health grade; SAIL, San Antonio Immunological Resilience Longitudinal cohort; UCSD, University of California San Diego cohort; VA-CLC, Veterans Affairs COVID-19 Longitudinal Cohort

Note table b. Correlation of $CD4^+$ and $CD8^+$ T-cell counts with the CD4:CD8 ratio in the SardiNIA cohort by age strata.

Age	*n*	Median (IQR)			$CD4^+$ and Ratio		$CD8^+$ and Ratio	
		**$CD4^+$**	**$CD8^+$**	**CD4:CD8**	**r**	**r^2**	**r**	**r^2**
Overall	3898	1023 (798-1280)	462 (337-634)	2.22 (1.66-2.93)	0.30	0.09	-0.71	0.51
15-39	1244	1103 (860-1397)	539 (414-701)	2.07 (1.62-2.62)	0.34	0.11	-0.61	0.37
40-59	1524	1023 (800-1287)	447 (338-611)	2.25 (1.69-3.01)	0.37	0.13	-0.69	0.48
60+	1130	953 (726-1170)	383 (266-567)	2.36 (1.67-3.42)	0.32	0.10	-0.78	0.61

r^2 presented from correlation of indicated value with CD4:CD8 ratio; IHG, immune health grade; IQR, interquartile range

Note table c. $CD4^+$ and $CD8^+$ counts and CD4:CD8 ratio by IHG in the indicated cohorts.

IHG	SAIL		
	**$CD4^+$**	**$CD8^+$**	**CD4:CD8**
IHG-I	1057 (916-1237)	506 (370-658)	2.14 (1.64-2.92)
IHG-IIa	681 (603-746)	365 (260-472)	1.83 (1.40-2.46)
IHG-IIb	427 (388-471)	234 (163-290)	1.79 (1.52-2.42)
IHG-IIc	193 (193-193)	100 (100-100)	1.93 (1.93-1.93)
IHG-III	920 (844-1127)	1198 (1012-1416)	0.84 (0.70-0.98)
IHG-IVa	616 (578-708)	760 (664-907)	0.82 (0.71-0.93)
IHG-IVb	396 (371-436)	506 (437-602)	0.78 (0.64-0.94)
IHG-IVc	176 (150-182)	274 (234-544)	0.57 (0.42-0.63)

IHG	VA-CLC: Acute subset		
	**$CD4^+$**	**$CD8^+$**	**CD4:CD8**
IHG-I	1040 (906-1244)	469 (355-640)	2.25 (1.71-3.07)
IHG-IIa	633 (563-695)	291 (204-402)	2.14 (1.58-3.04)
IHG-IIb	337 (265-409)	152 (102-223)	2.10 (1.53-3.03)
IHG-IIc	144 (108-172)	67 (42-92)	2.02 (1.44-2.80)
IHG-III	972 (858-1073)	1200 (1063-1518)	0.82 (0.70-0.90)
IHG-IVa	611 (559-709)	780 (679-922)	0.84 (0.68-0.90)
IHG-IVb	332 (252-397)	448 (353-579)	0.76 (0.57-0.88)
IHG-IVc	124 (86-153)	184 (134-245)	0.70 (0.53-0.85)

IHG	VA-CLC: Post-Acute subset		
	**$CD4^+$**	**$CD8^+$**	**CD4:CD8**
IHG-I	1034 (900-1254)	457 (336-651)	2.30 (1.72-3.22)
IHG-IIa	635 (584-711)	342 (250-448)	1.82 (1.47-2.58)
IHG-IIb	418 (357-469)	210 (141-290)	1.77 (1.32-2.76)
IHG-IIc	166 (155-176)	95 (70-116)	1.80 (1.51-2.25)
IHG-III	950 (864-1008)	1306 (1108-1408)	0.79 (0.67-0.90)
IHG-IVa	645 (582-728)	856 (652-964)	0.81 (0.74-0.93)
IHG-IVb	379 (288-446)	563 (444-737)	0.71 (0.44-0.85)
IHG-IVc	99 (99-99)	855 (855-855)	0.12 (0.12-0.12)

Median (IQR) values presented. IHG, immune health grade; SAIL, San Antonio Immunological Resilience Longitudinal cohort; VA-CLC, Veterans Affairs COVID-19 Longitudinal Cohort

8. Supplementary Figures

Note: Figure legends are presented at the top of the figures as panels on some figures span across multiple pages and for ease of access figure numbers are embedded in the figures as well.

Figure S1. Health Cycle associated with Optimal IR-*TCF7*^{high}-associated salutogenesis. IHG, Immune Health Grade. IR, immune resilience; SAS, Survival-associated signature; MAS, Mortality-associated signature; TF, transcription factor; *TCF7*, transcription factor 7; AD, Alzheimer's disease; CVD, cardiovascular diseases.

Figure S1

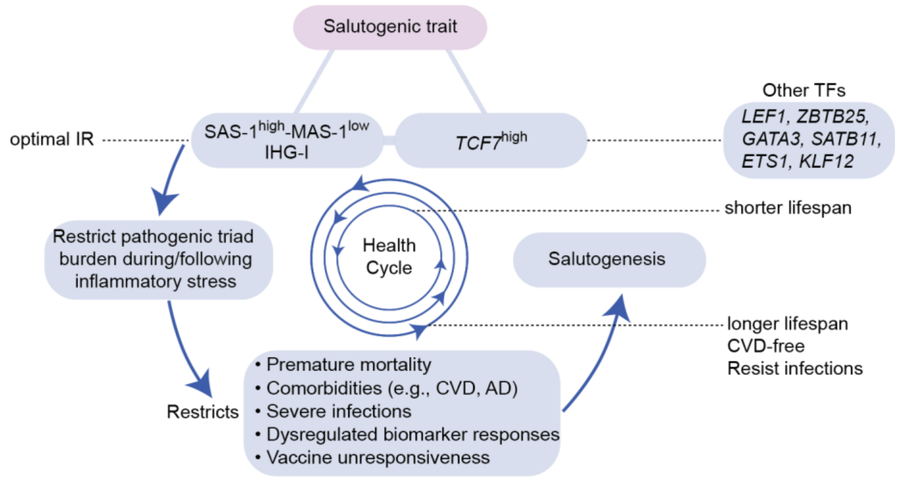

Figure S2. Correlations among the immune resilience metrics, gene signatures tracking the pathogenic triad, transcription factors (*TCF7* and *LEF1*), and age. Correlation plots of indicated genes and gene signatures and age in the Framingham Heart Study presented with color gradient heatmap and pie charts. Color gradient of blue to red, low to high value of Pearson's correlation coefficient (r). Details of signatures are in Figure 1d (main) and Table S1, and gene composition in gene signatures are in Table S2. Higher levels of the IMM-AGE signature were computed to signify an association with fewer senescent T-cells (less immune aging and lower mortality; a {+}-salutogenesis readout), as detailed in Section 4.2. Statistical details in Section 6.2.2. Data correspond to Fig. 1g (main).

Figure S2

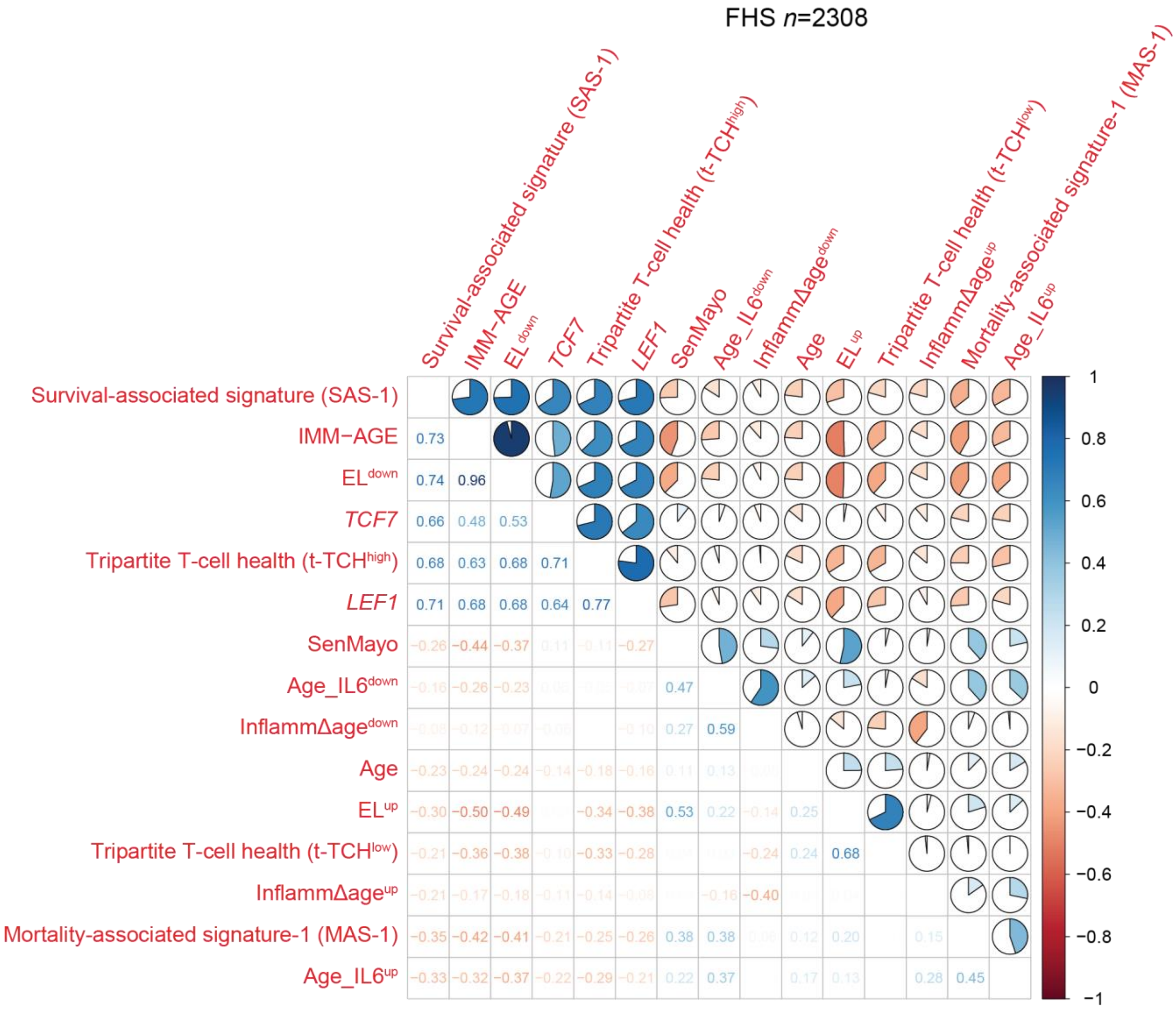

Figure S3. Association of immune resilience metrics, gene signatures tracking the pathogenic triad, and transcription factors (*TCF7* and *LEF1*) in lymphocytes and leukocytes in scRNA-seq datasets. Expression of indicated gene signatures (z-scores) in the following datasets: GSE94820, GSE106540, GSE115189, GSE119428, GSE124731, GSE128066, GSE150728, GSE150861, GSE167029, and supercentenarians dataset. Color gradient of blue to red corresponding to z-score expression ranging from low to high. Size of dot corresponds to % expressed of signature in each cluster. Analysis performed using Seurat R package. Some clusters were not fully ascertained to a specific cell type, and they were labeled as unidentified. Some clusters were partially ascertained to a specific cell type, and they are labelled with a '?' symbol in front of the cell type label. x-axis depicts IR metrics [survival-associated signature 1 (SAS-1), Mortality-associated signature-1 (MAS-1)], the {+} and {–}-salutogenesis readouts, TFs (transcription factors), and other signatures. Details of signatures are in Figure 1d (main) and Table S1, and gene composition in gene signatures are in Table S2. Higher levels of the IMM-AGE signature were computed to signify an association with fewer senescent T-cells (less immune aging and lower mortality; a {+}-salutogenesis readout), as detailed in Section 4.2. Statistical details are in Section 6.2.3.

Figure S3 (corresponds to main Figure 2a)

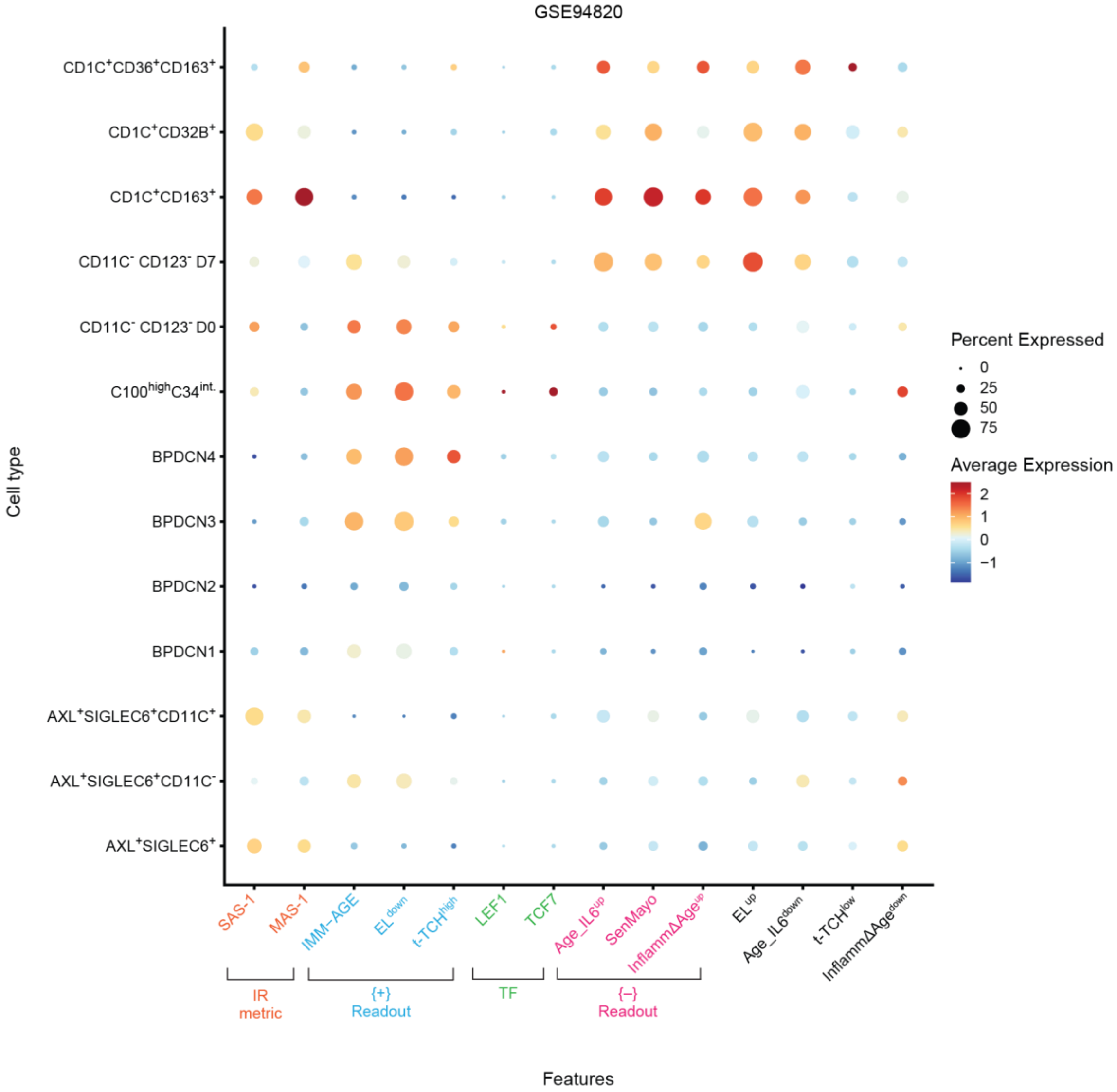

Key	
BPDCN1-4	blastic plasmacytoid dendritic cell neoplasm (CD45+HLA-DR+CD123+ neoplasia patients 1-4)
D	Day

Figure S3 (continued - corresponds to main Figure 2a)

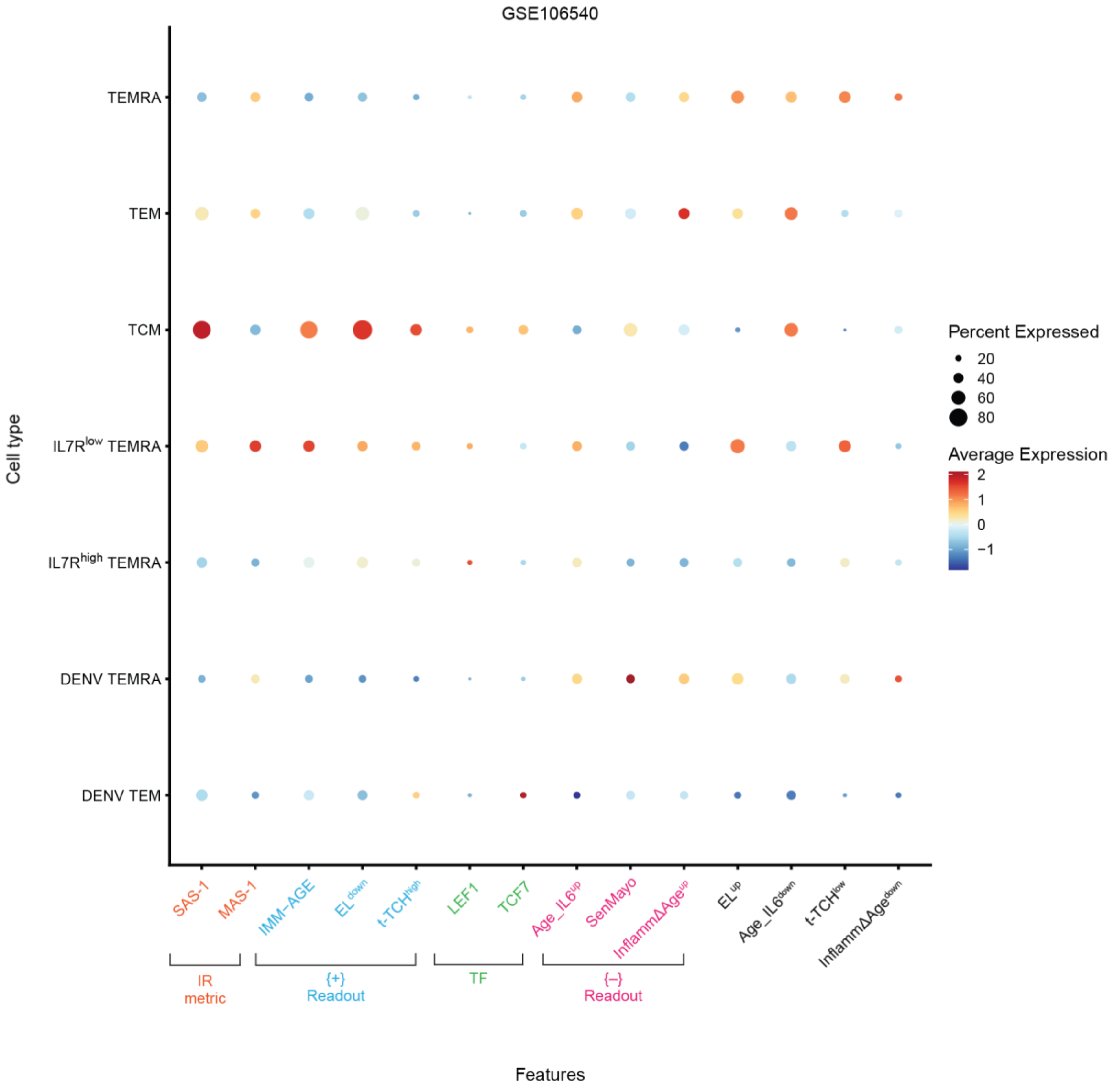

Key

TEMRA	effector memory T cells expressing CD45RA
TEM	effector memory cells T cells
TCM	central memory cells T cells
DENV	dengue virus-specific

Figure S3 (continued - corresponds to main Figure 2a)

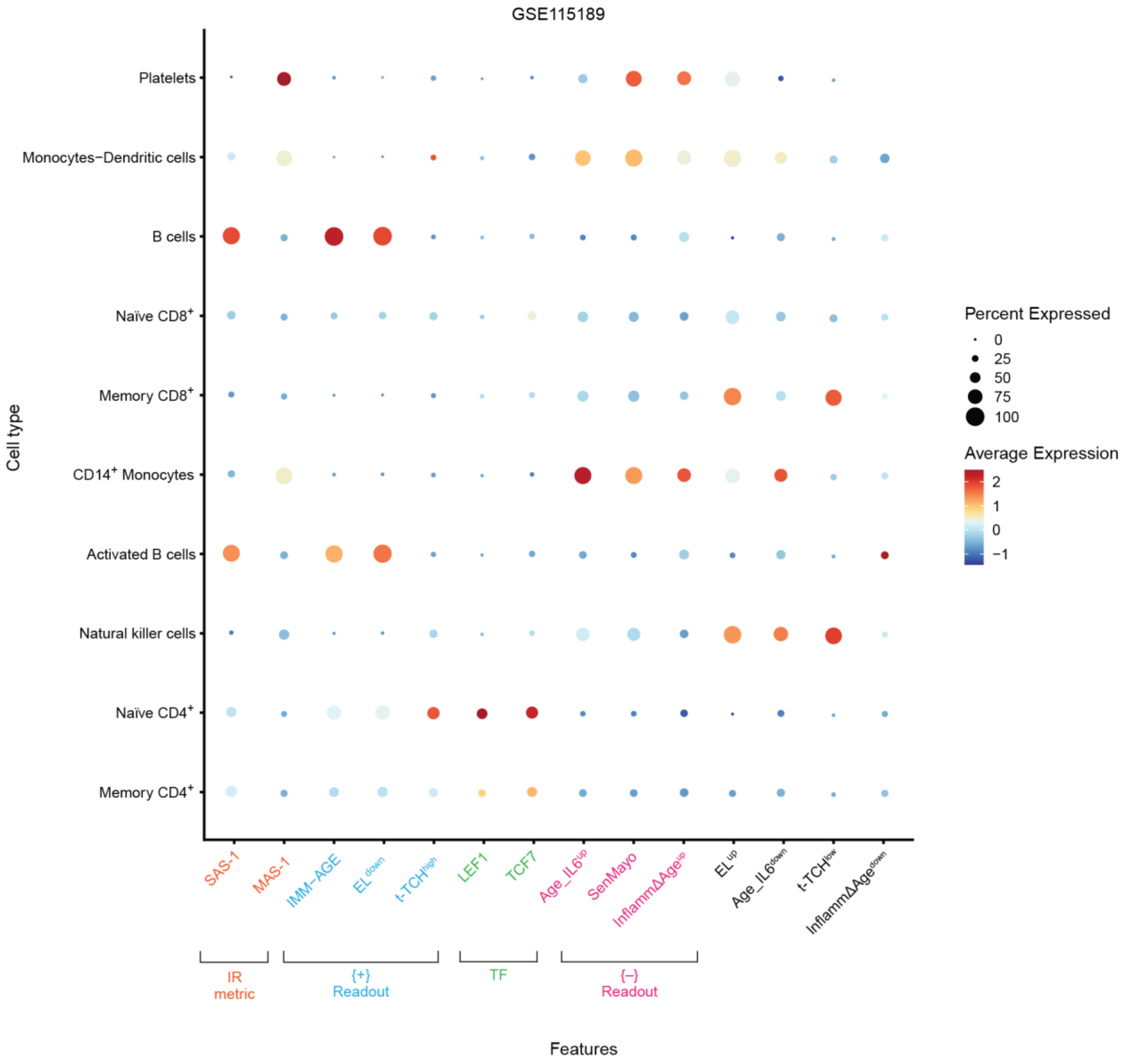

2628

Figure S3 (continued - corresponds to main Figure 2a)

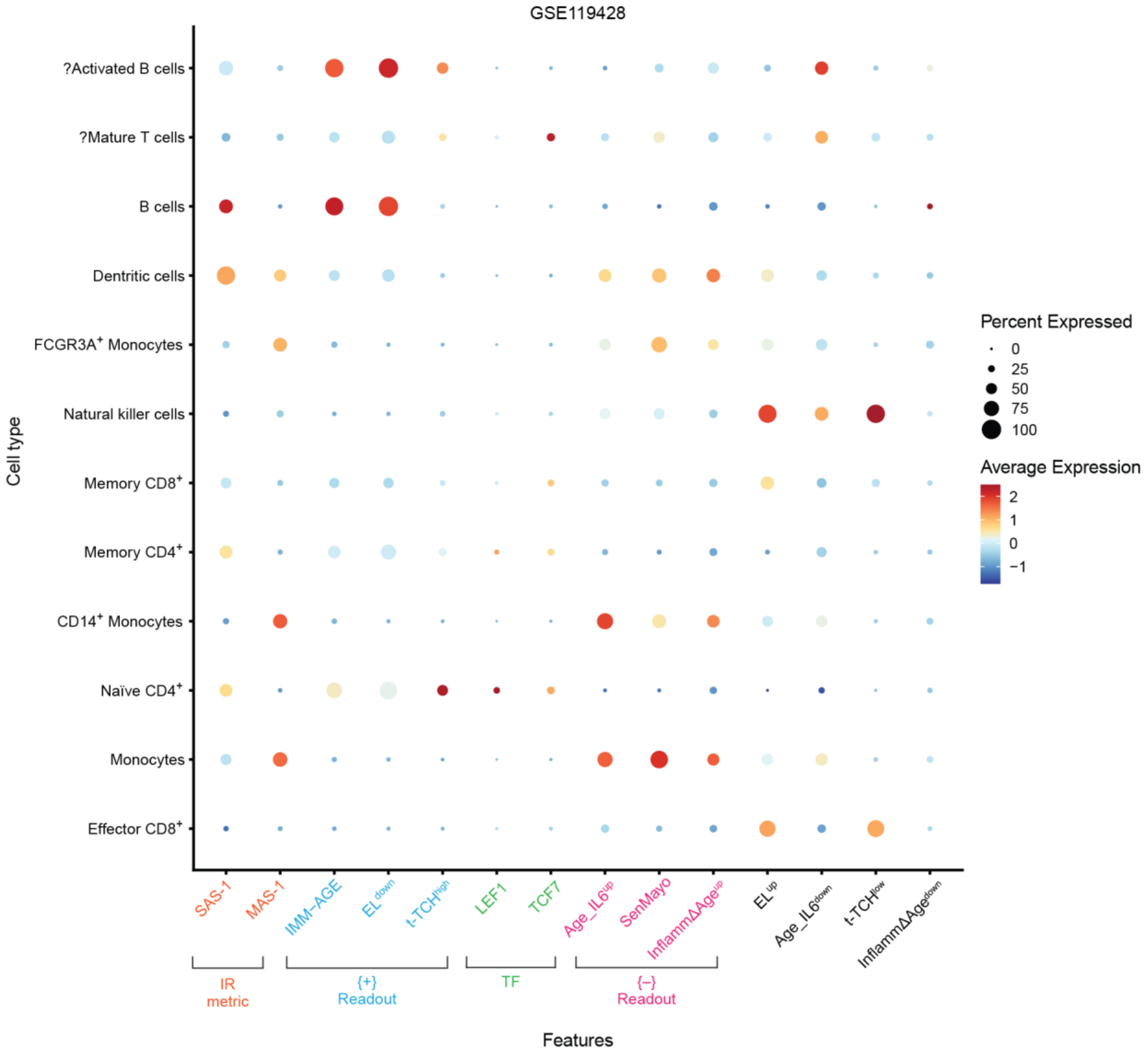

2629

Figure S3 (continued - corresponds to main Figure 2a)

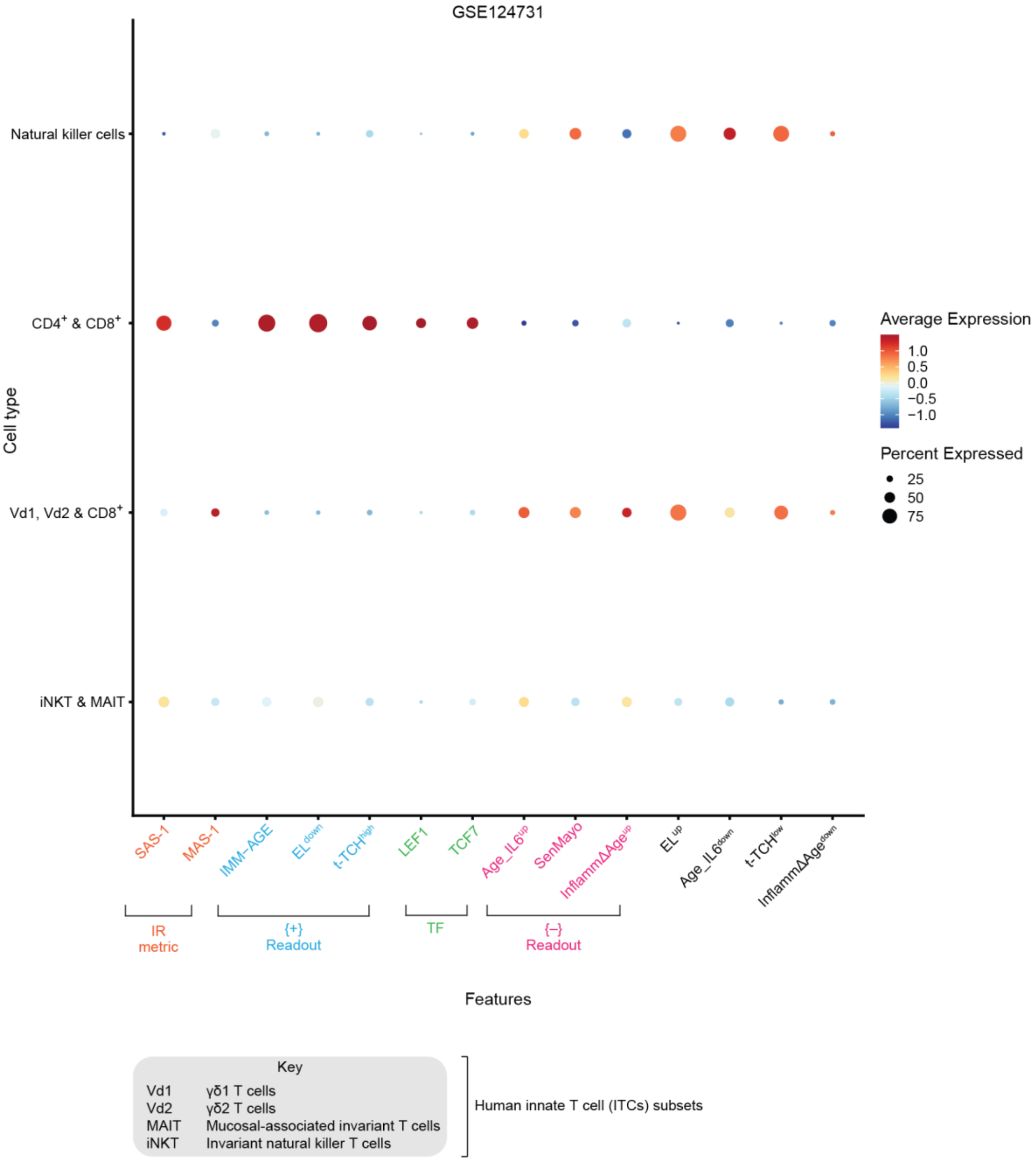

Figure S3 (continued - corresponds to main Figure 2a)

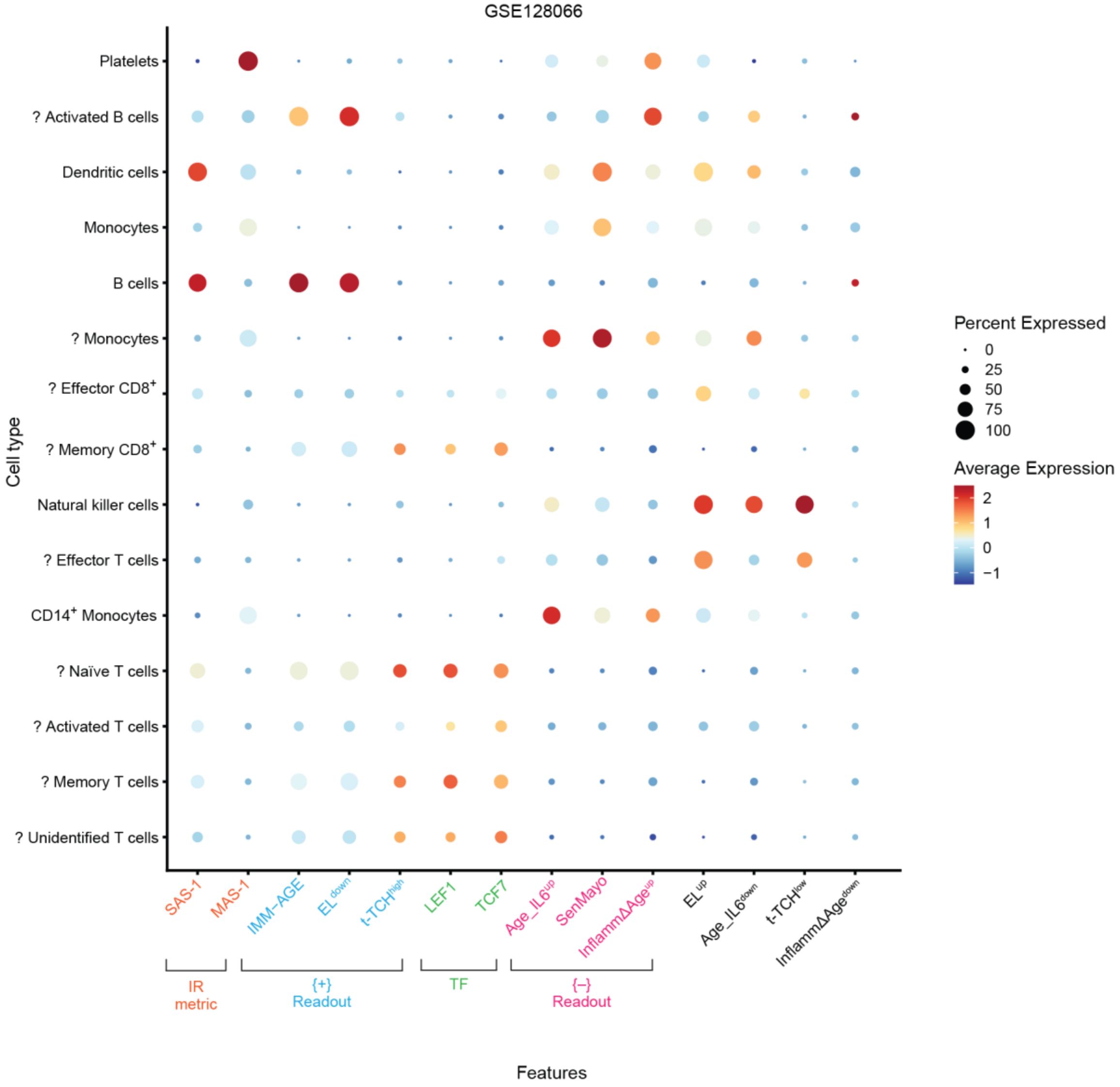

2631

Figure S3 (continued - corresponds to main Figure 2a)

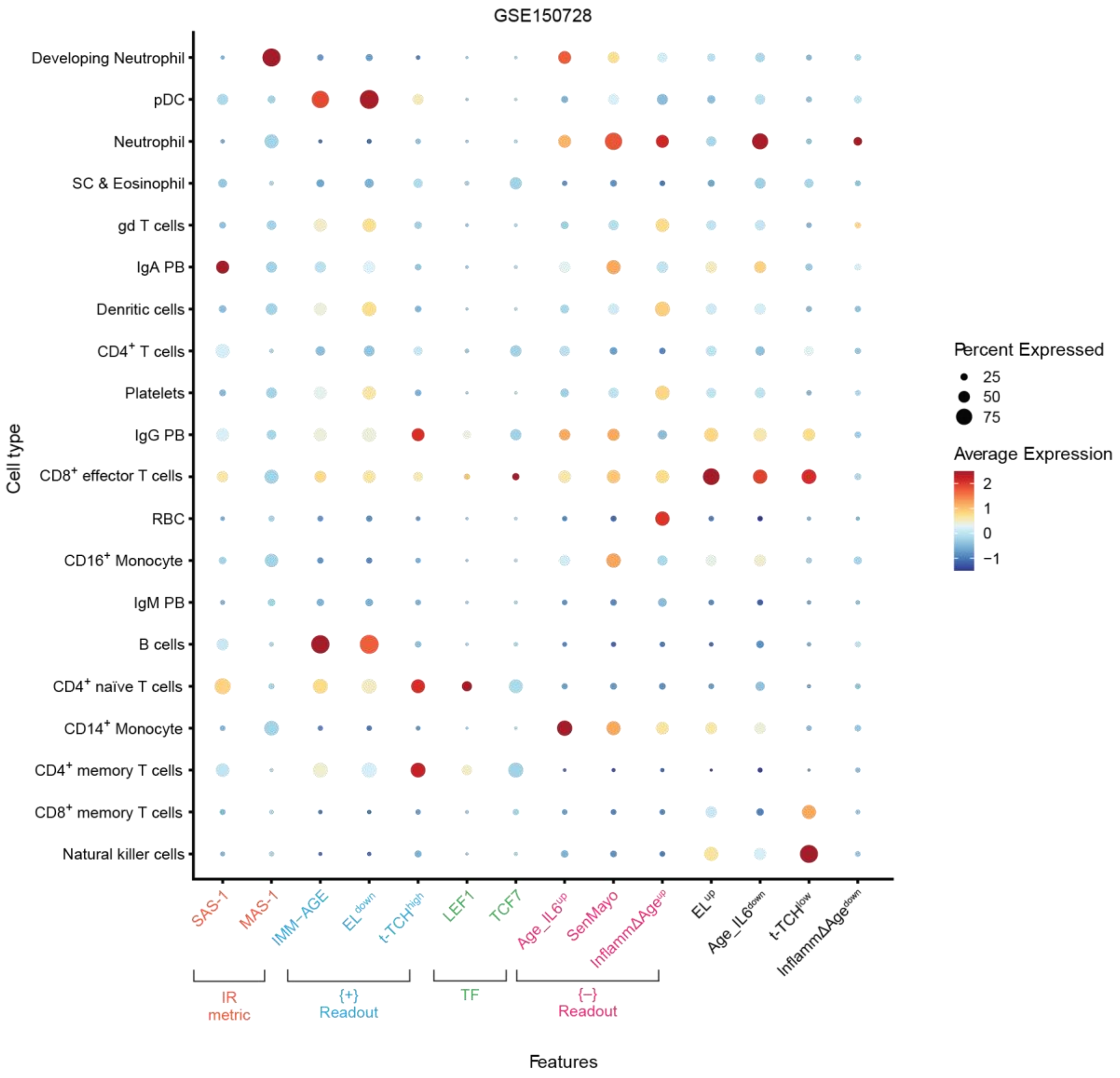

Key

pDC	Plasmacytoid dendritic cells
SC	Stem cells
gd T	γδ T cells
PB	Plasmablasts
DC	Dendritic cells
RBC	Red blood cells
IgA	Immunoglobin A
IgG	Immunoglobin G
IgM	Immunoglobin M

Figure S3 (continued - corresponds to main Figure 2a)

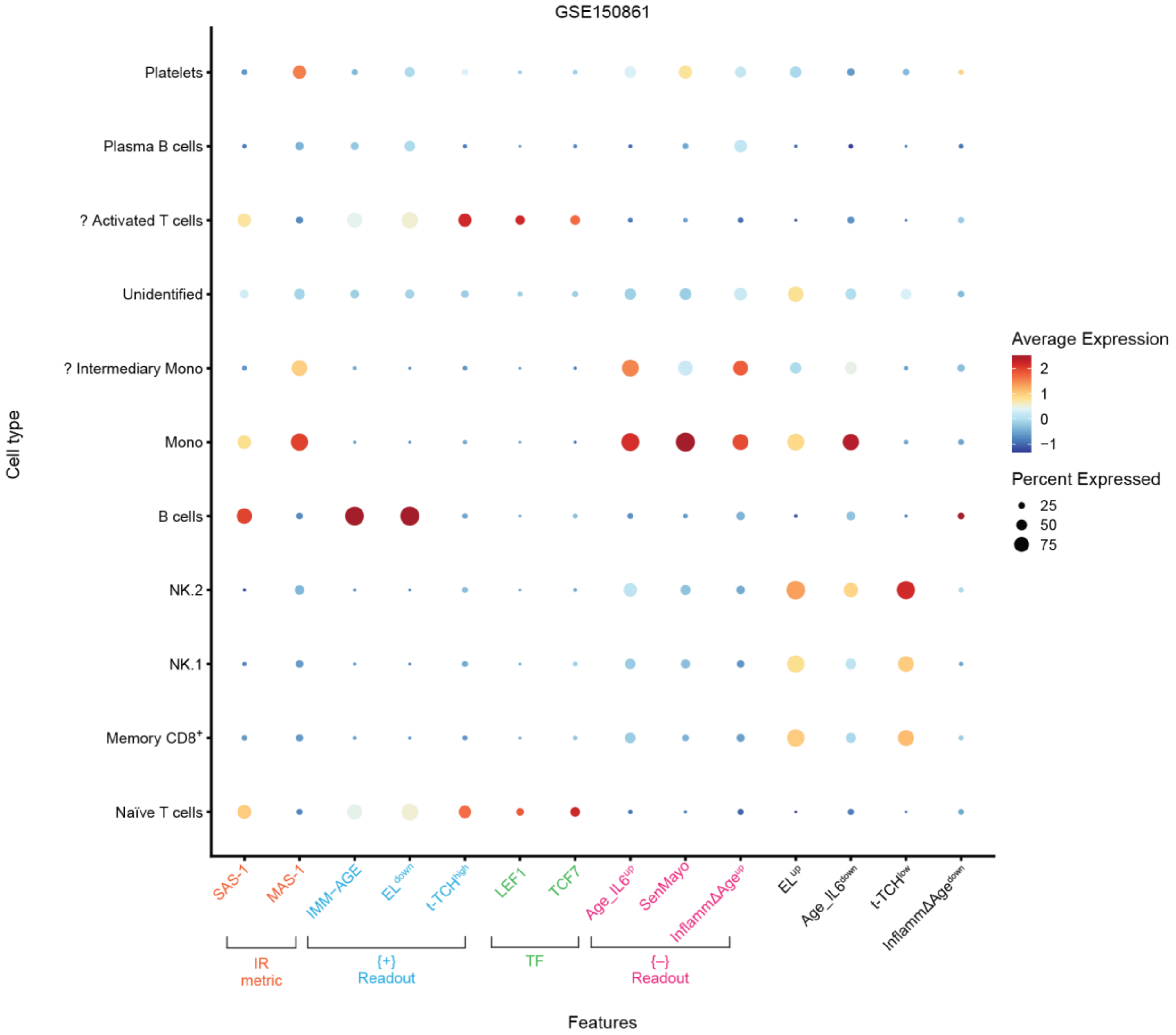

Key

PB	Plasma B cells
Mono	Monocytes
NK	Natural killer cells

Figure S3 (continued - corresponds to main Figure 2a)

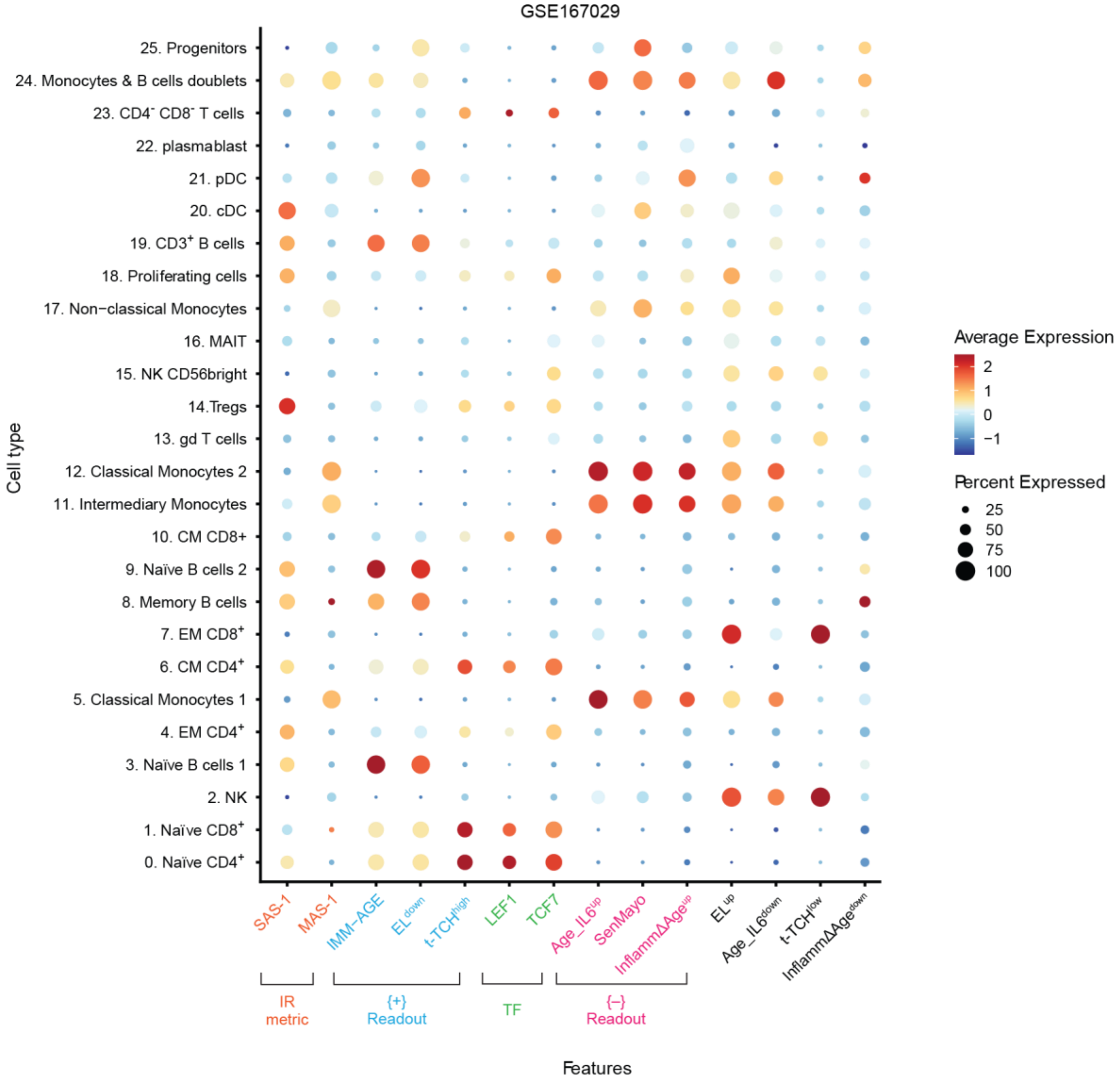

Key	
pDC	Plasmacytoid dendritic cells
cDC	Conventional dendritic cells
MAIT	Mucosal-associated invariant T cells
Tregs	Regulatory T cells (CD25+)
gd	γδ
EM	Effector memory
CM	Central memory
NK	Natural killer cells

Figure S3 (continued - corresponds to main Figure 2a)

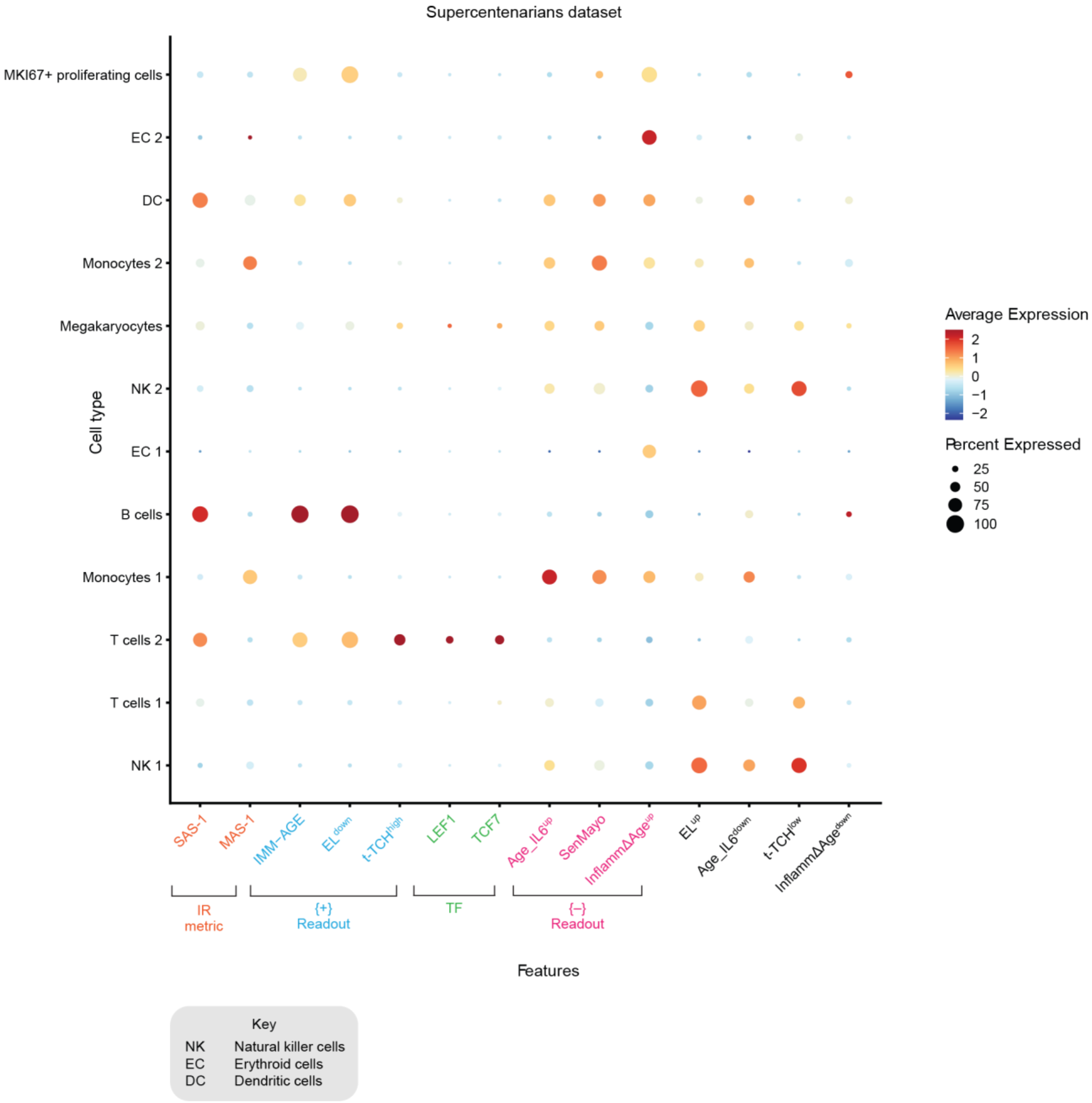

Figure S4. Association of immune resilience metrics, gene signatures tracking the pathogenic triad, and *TCF7* expression levels in sorted T-cells. Boxplots depict median (IQR) of the indicated gene signatures and *TCF7* expression in indicated sorted T cell subsets (GSE179609). EMRA, effector memory RA; CM, central memory; Tfh, T follicular helper cells; SCM, stem cell memory; PD-1, programmed cell death protein 1; R3, CXCR3. NGE, normalized gene expression. Grp, group. Details of signatures are in Figure 1d (main) and Table S1. Higher levels of the IMM-AGE signature were computed to signify an association with fewer senescent T-cells (less immune aging and lower mortality; a {+}-salutogenesis readout), as detailed in Section 4.2. Statistical details are in Section 6.2.4.

Figure S4

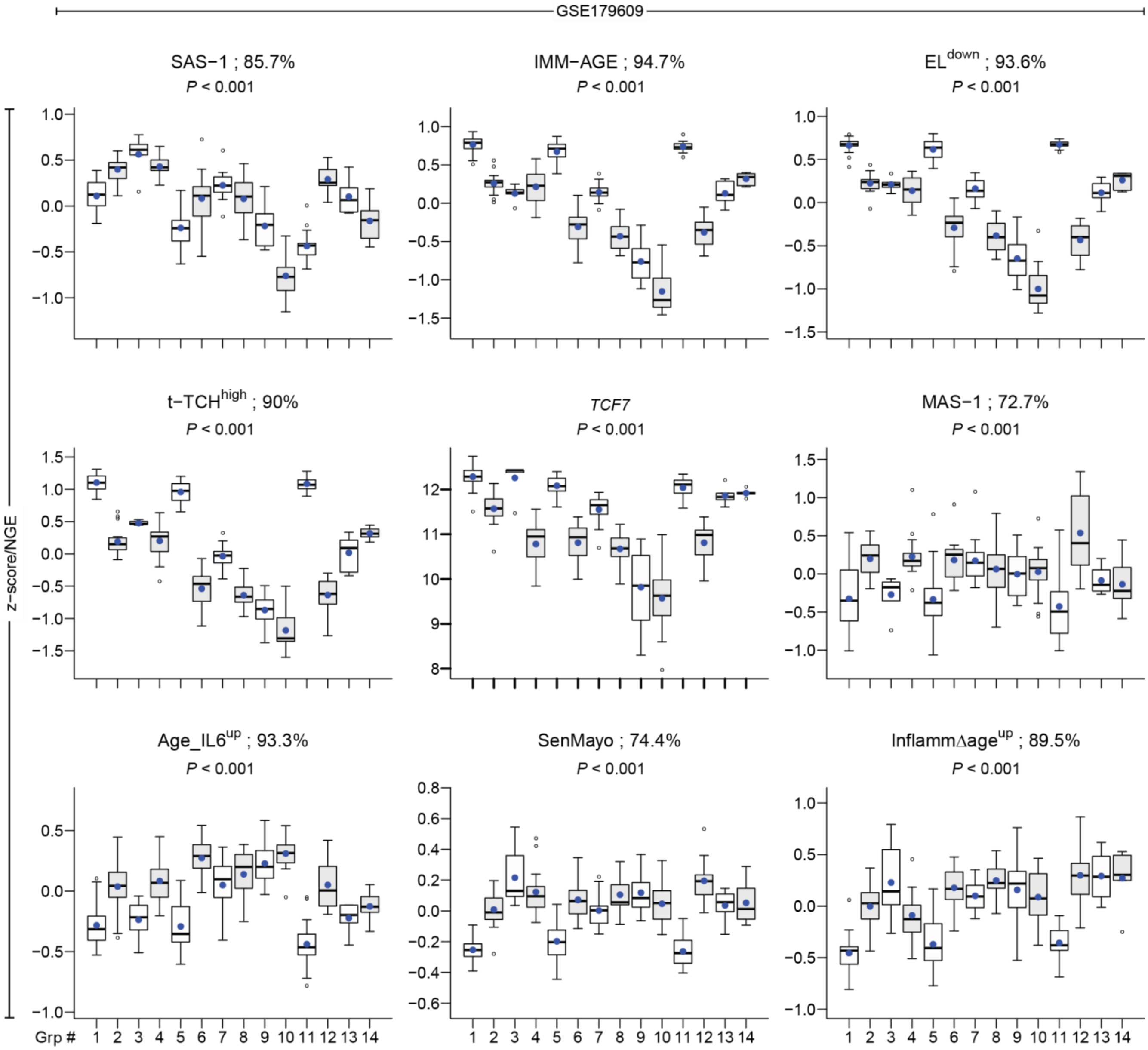

Key

#	Grp	n
1	Bulk naïve CD4⁺	17
2	Bulk non-naïve CD4⁺	18
3	Follicular helpers CD4⁺	6
4	Regulatory CD4⁺	17
5	Bulk naïve CD8⁺	20
6	Bulk non-naïve CD8⁺	21
7	central memory CD8⁺	18
8	effector memory 1 CD8⁺	19
9	effector memory 2 CD8⁺	16
10	effector memory (CD45RA⁺) CD8⁺	19
11	Naïve CD8⁺	17
12	PD1⁺ CD39⁺ CD8⁺	12
13	Stem cell memory (SCM) CXCR3⁻ CD8⁺	6
14	Stem cell memory (SCM) CXCR3⁺ CD8⁺	6

Figure S5. Immune resilience (IR) status and *TCF7* expression levels during aging, infectious diseases, and other acute and chronic inflammatory conditions. IR status is indexed to SAS-1/MAS-1 profiles. Stacked barplots depict distribution of SAS-1/MAS-1 profiles (key, bottom right) and dot-and-line plots depict median (IQR) of *TCF7* expression [normalized gene expression (NGE)] by indicated groups in these cohorts by: **(a)** age strata in San Antonio Family Heart Study cohort (E-TABM-305) and Brisbane cohort (GSE53195); **(b)** SLEDAI, neutrophil quartiles, IFN signature metric, and anti-Ro antibody levels in SLE cohorts (GSE121139 and GSE72509); **(c)** treatment timepoints with treatment outcome (cure vs. not cured), disease stage, and tuberculosis progressor status in tuberculosis cohorts (GSE89403, GSE69581, GSE79362); **(d)** infection status (sev., severe) and quartiles of parasite density values, plasma *Plasmodium falciparum* histidine-rich protein 2 (PfHRP2) titers, and indicated biomarkers in the Uganda malaria cohort (GSE117613); **(e)** infection status and parasite density values in the Mali malaria cohort (GSE52166); **(f)** timepoint from symptom onset and disease stage in dengue virus infection cohorts (GSE28405, GSE43777, GSE140809, GSE100299); **(g)** viral and bacterial infections, enteric fever groups, Kawasaki disease, other inflammatory conditions, disease status (acute, discharged from hospital), and quartiles of baseline %lymphocytes and %neutrophils (GSE157240, GSE42026, GSE73464, GSE80496, GSE60244, GSE113866, GSE63881, GSE97741, GSE113210, GSE115823); and **(h)** nontuberculous mycobacterium (NTM) infection status, chronic inflammatory conditions (ulcerative colitis, Crohn’s disease); metabolic syndrome with obesity status, and renal transplant tolerant group (GSE205161, GSE3365, GSE145412, GSE45593). Note: There is an increase in the prevalence of SAS-1^{low}-MAS-1^{high} by age, disease severity/stage, biomarker levels, treatment outcomes, and parasite density; conversely, the prevalence of SAS-1^{high}-MAS-1^{low} increases during convalescence and with cure status for TB. Statistical details are in Section 6.2.5.

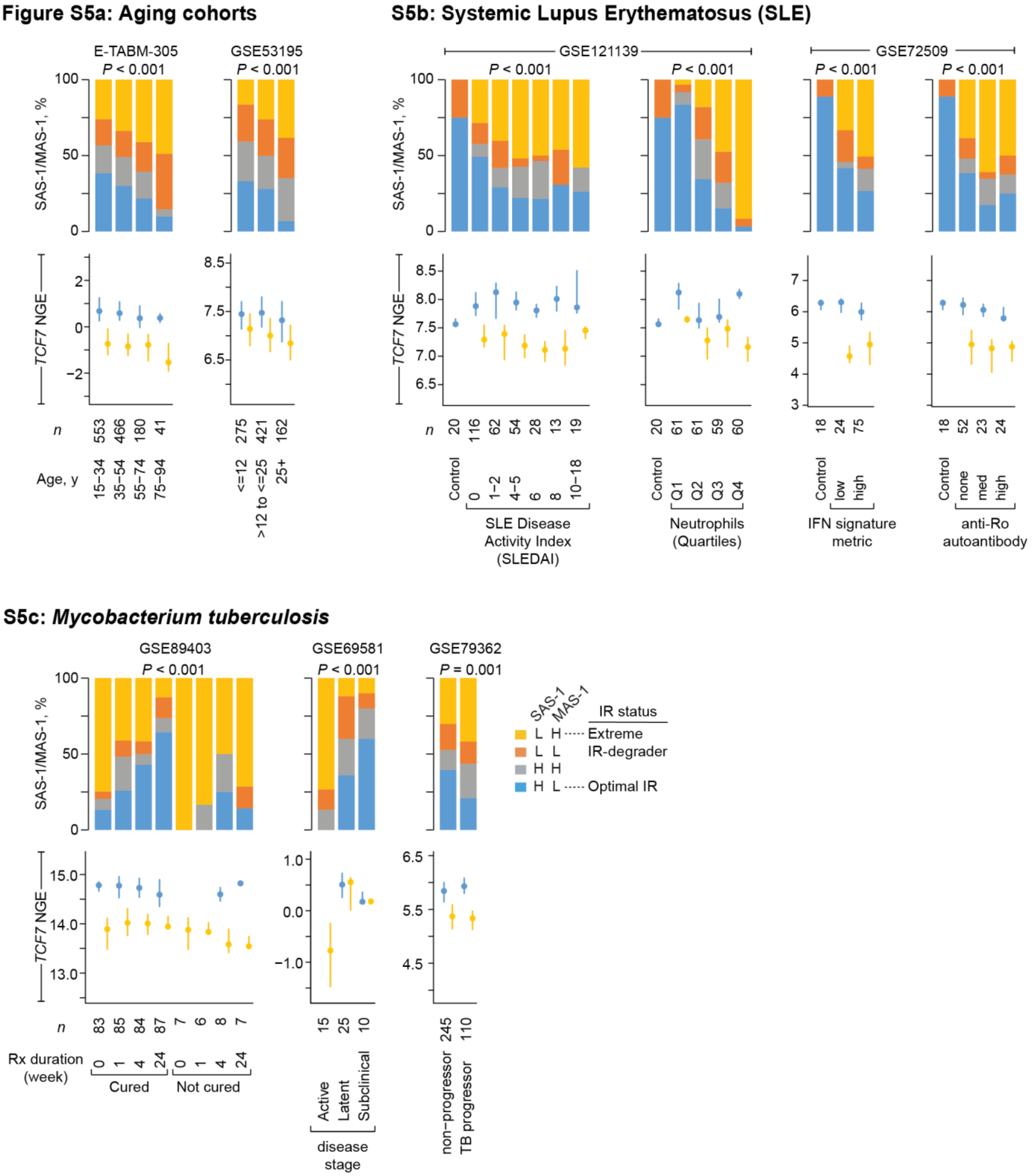
Figure S5a: Aging cohorts
S5b: Systemic Lupus Erythematosus (SLE)
E-TABM-305
GSE53195
GSE121139
GSE72509
P < 0.001
SAS-1/MAS-1, %
TCF7 NGE
n
Age, y
15–34
35–54
55–74
75–94
<=12
>12 to <=25
25+
Control
SLE Disease Activity Index (SLEDAI)
Neutrophils (Quartiles)
IFN signature metric
anti-Ro autoantibody
S5c: Mycobacterium tuberculosis
GSE89403
GSE69581
GSE79362
P = 0.001
Rx duration (week)
Cured
Not cured
Active
Latent
Subclinical
disease stage
non−progressor
TB progressor
SAS-1
MAS-1
IR status
L H Extreme
L L IR-degrader
H H
H L Optimal IR

Figure S5d: Malaria- *Plasmodium falciparum*

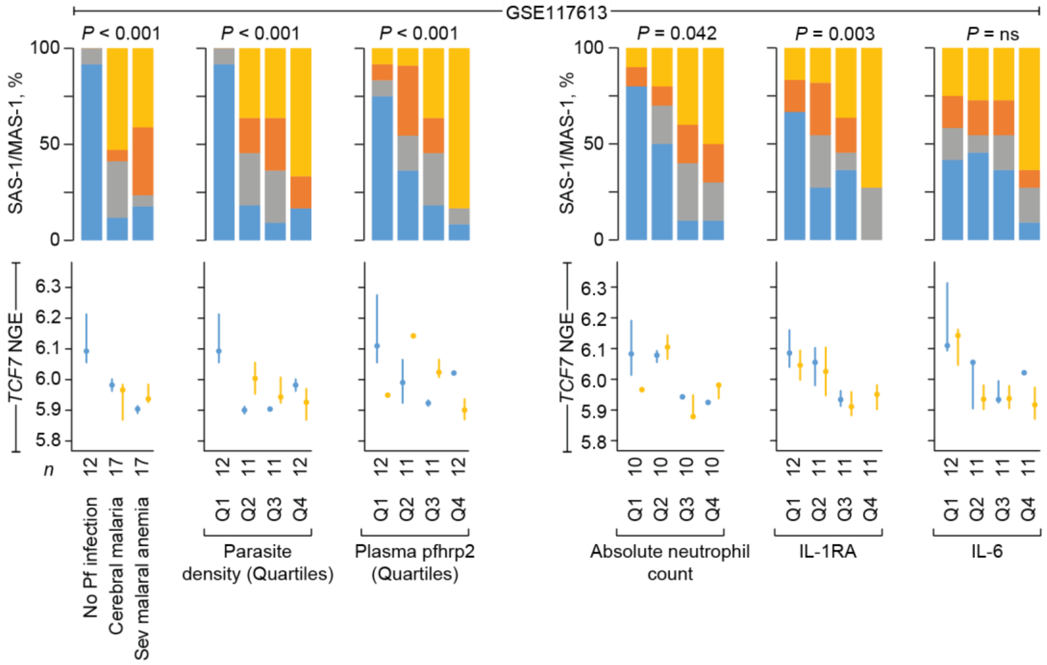

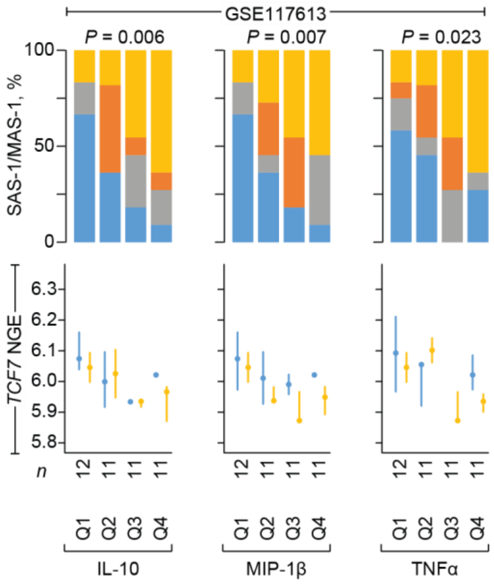

S5e: Malaria- *Plasmodium falciparum*

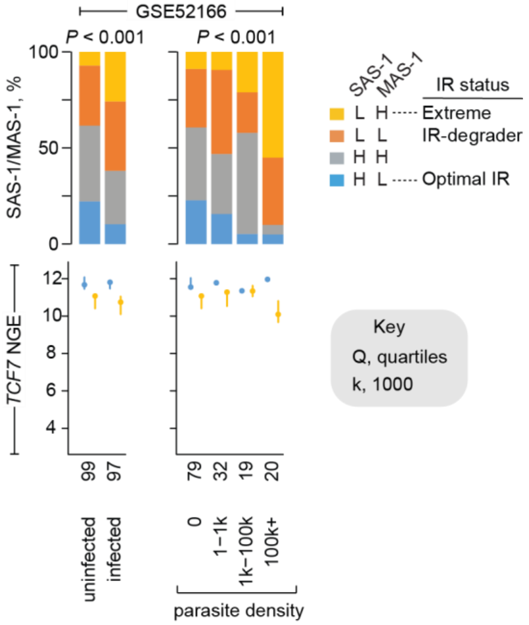

Figure S5f: Dengue

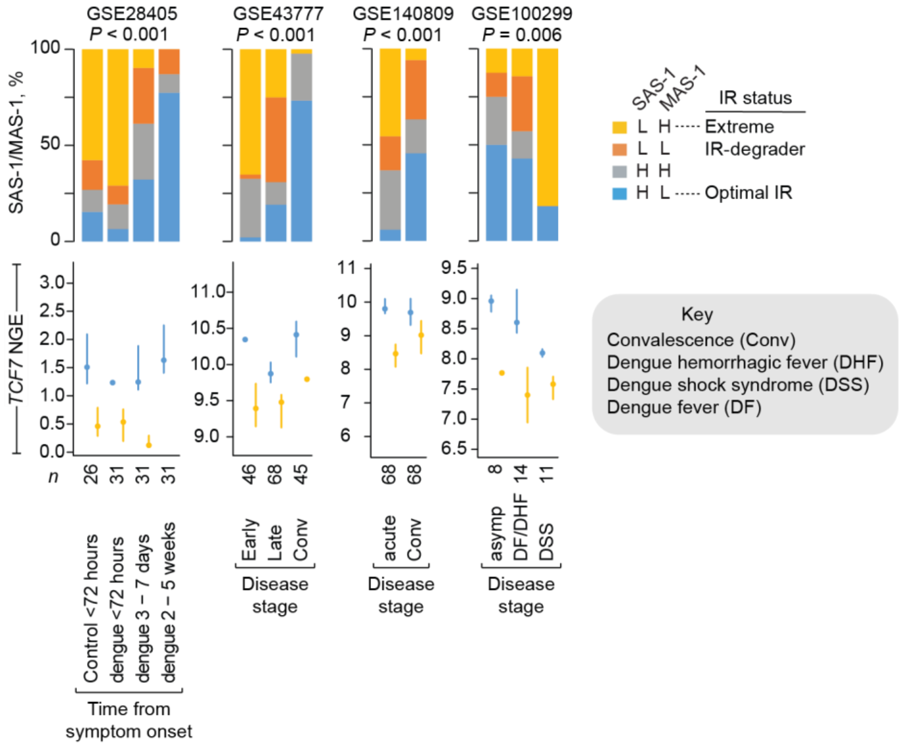

2676

Figure S5g: Miscellaneous acute infections and inflammatory conditions

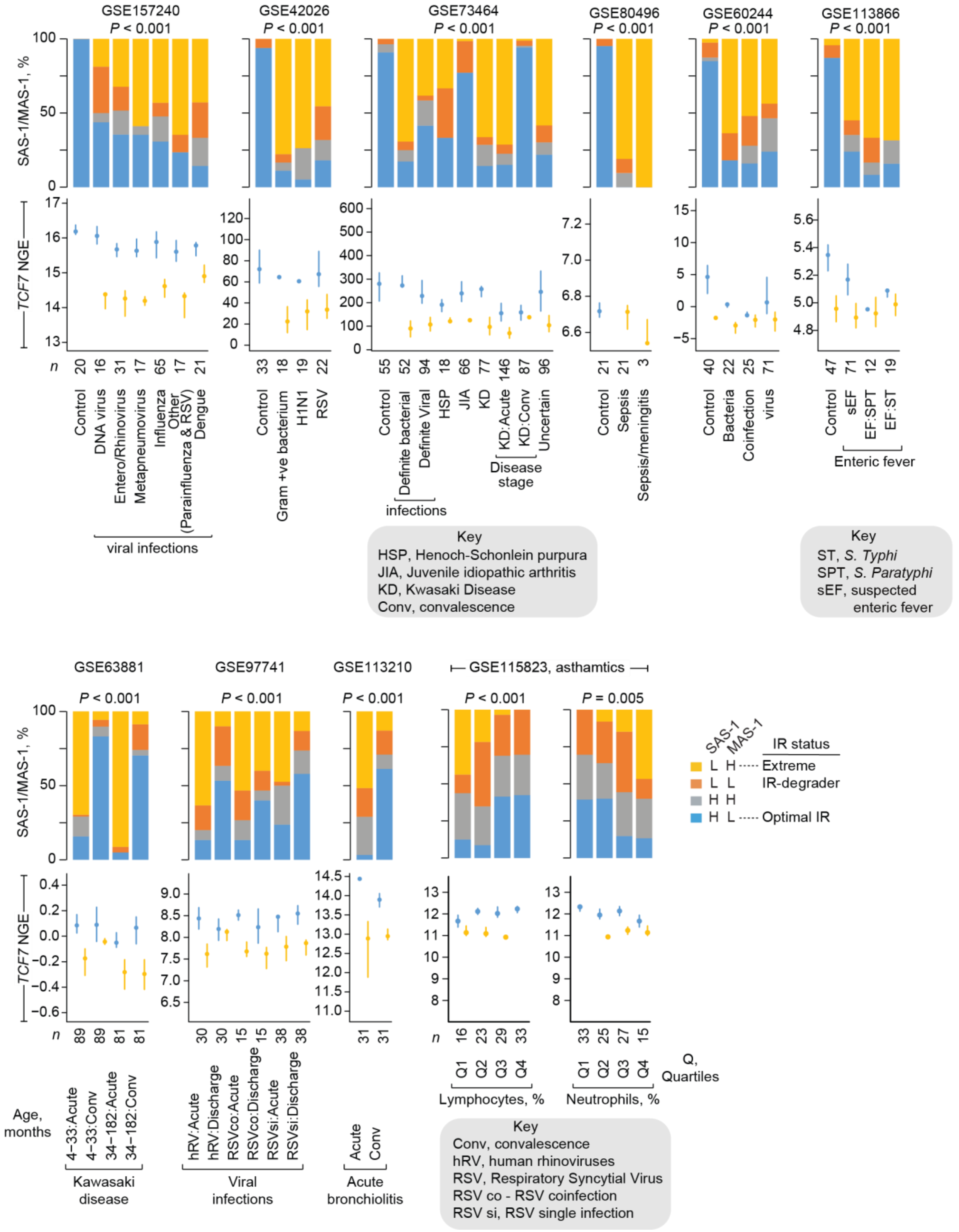

Figure S5h: Chronic inflammatory conditions

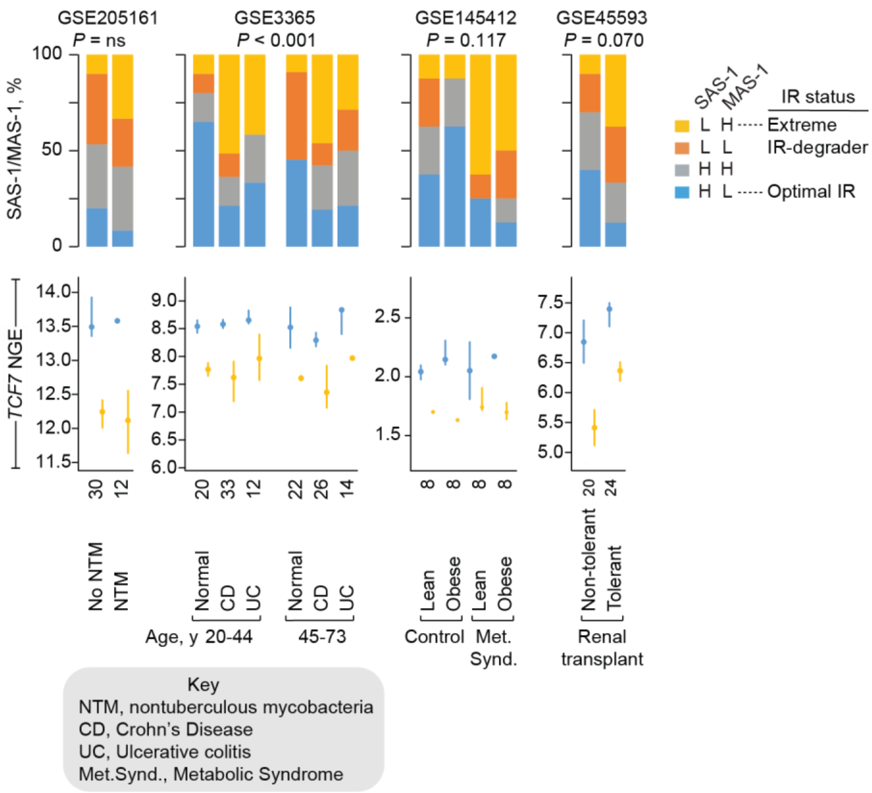

2678

Figure S6. Association of immune resilience (IR) metrics, gene signatures tracking the pathogenic triad, and *TCF7* expression levels by sex and optimal vs. extreme IR degradation status across age in the FHS. Linear regression modeling of the expression of the indicated gene signatures (z-scores) and *TCF7* expression with 95% confidence bands stratified by **(a)** sex and **(b)** SAS-1/MAS-1 profiles tracking optimal IR (SAS-1^{high}-MAS-1^{low}) and extreme IR degradation (SAS-1^{low}-MAS-1^{high}) status. NGE, normalized gene expression. Details of the signatures are in Figure 1d (main) and Table S1. Higher levels of the IMM-AGE signature were computed to signify an association with fewer senescent T-cells (less immune aging and lower mortality; a {+}-salutogenesis readout), as detailed in Section 4.2. Statistical details in Section 6.2.6. Data correspond to Figure 3a (main).

Figure S6a (corresponds to main Figure 3a)

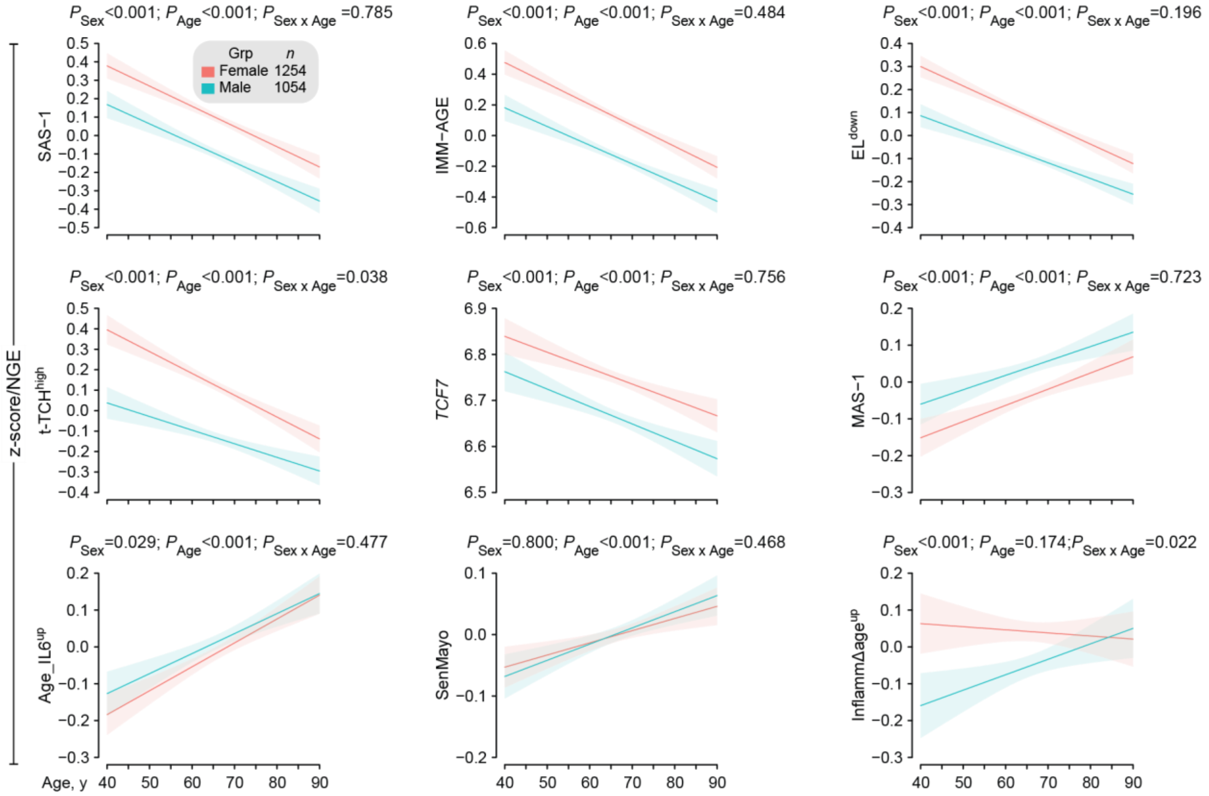

Figure S6b (corresponds to main Figure 3a)

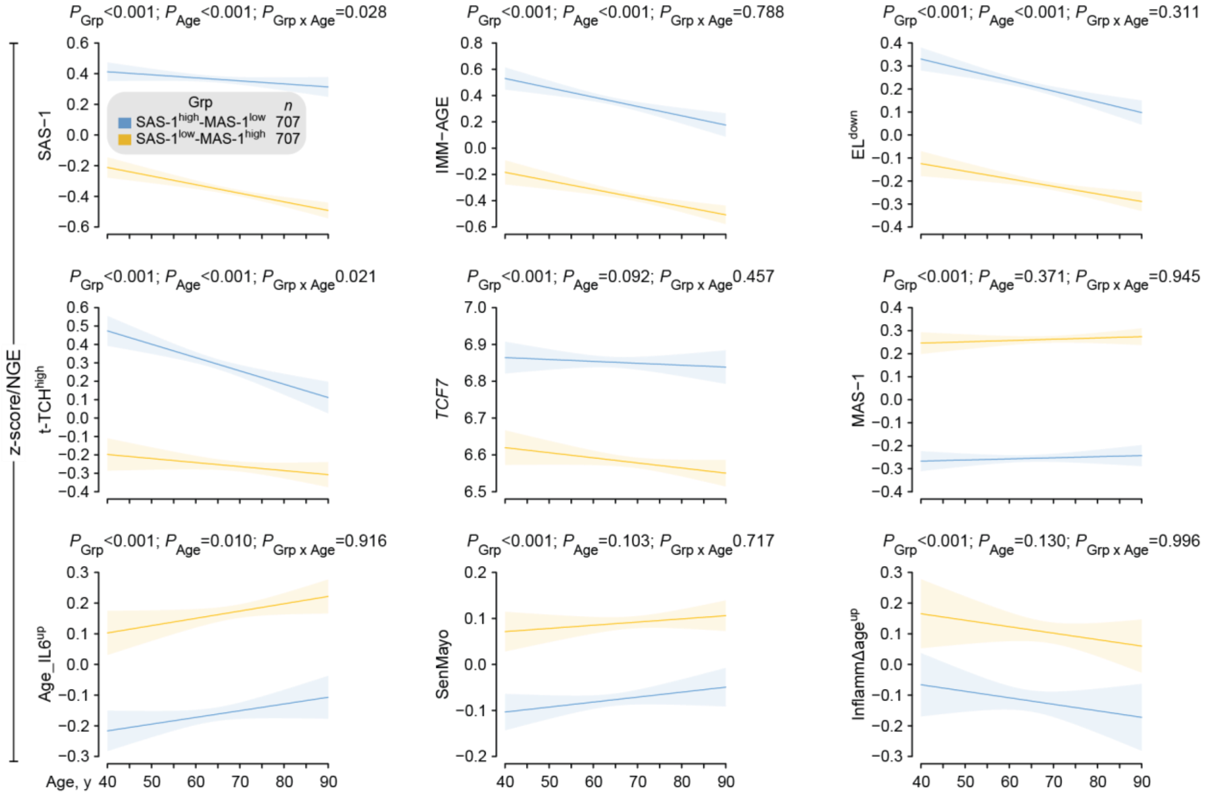

Figure S7. Association of gene signatures tracking the pathogenic triad by age and in persons with optimal vs. extreme IR degradation status in three cohorts. Boxplots depict median (IQR) expression levels of the indicated gene signatures (z-scores) and mean difference beta (β) values by age and SAS-1/MAS-1 profiles in the **(a)** Framingham Heart Study (FHS), **(b)** San Antonio Family Heart Study (Mexican American, E-TABM-305), and **(c)** Brisbane (GSE53195) cohorts. NGE, normalized gene expression. SAS-1^{high}-MAS-1^{low} (H-L) indicates optimal IR; SAS-1^{low}-MAS-1^{high} (L-H) indicates extreme IR degradation status. Younger vs. Older (Y vs. O) age cut-offs [years with median (IQR) within those cut-offs] in FHS: Y<56: 53 (50-54) *vs.* ≥76: 79 (77-82); Mexican American: <25: 20 (15-22) *vs.* ≥50: 60 (53-68); and Brisbane: <20: 14 (12-16) *vs.* ≥40: 47 (44-49). Details of the signatures are in Figure 1d (main) and Table S1. Higher levels of the IMM-AGE signature were computed to signify an association with fewer senescent T-cells (less immune aging and lower mortality; a {+}-salutogenesis readout), as detailed in Section 4.2. Statistical details are in Section 6.2.7. Data correspond to Figure 3b-c (main).

Figure S7a (corresponds to main Figure 3b and Figure 3C left panel)

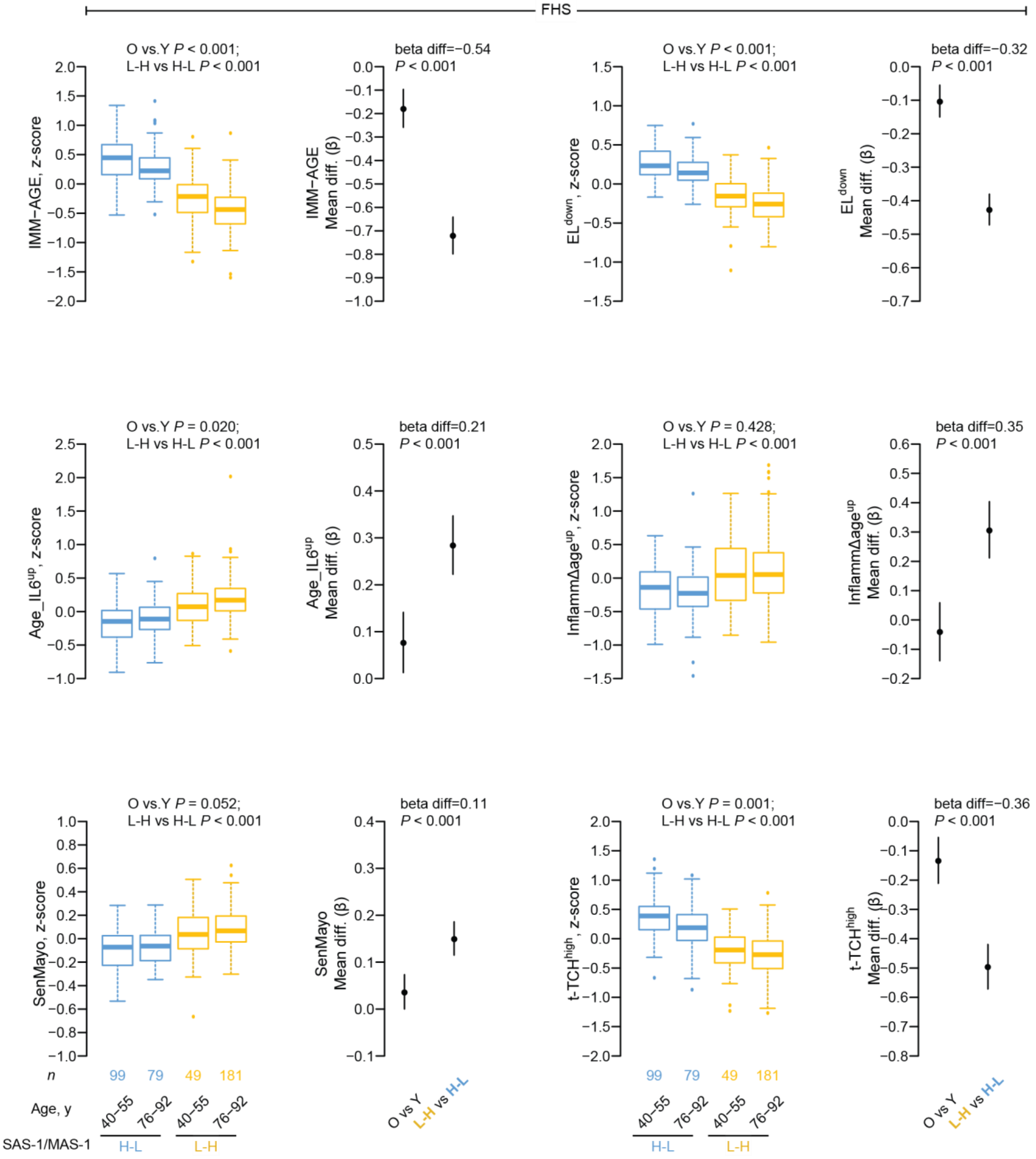

Figure S7b (corresponds to main Figure 3c middle panel)

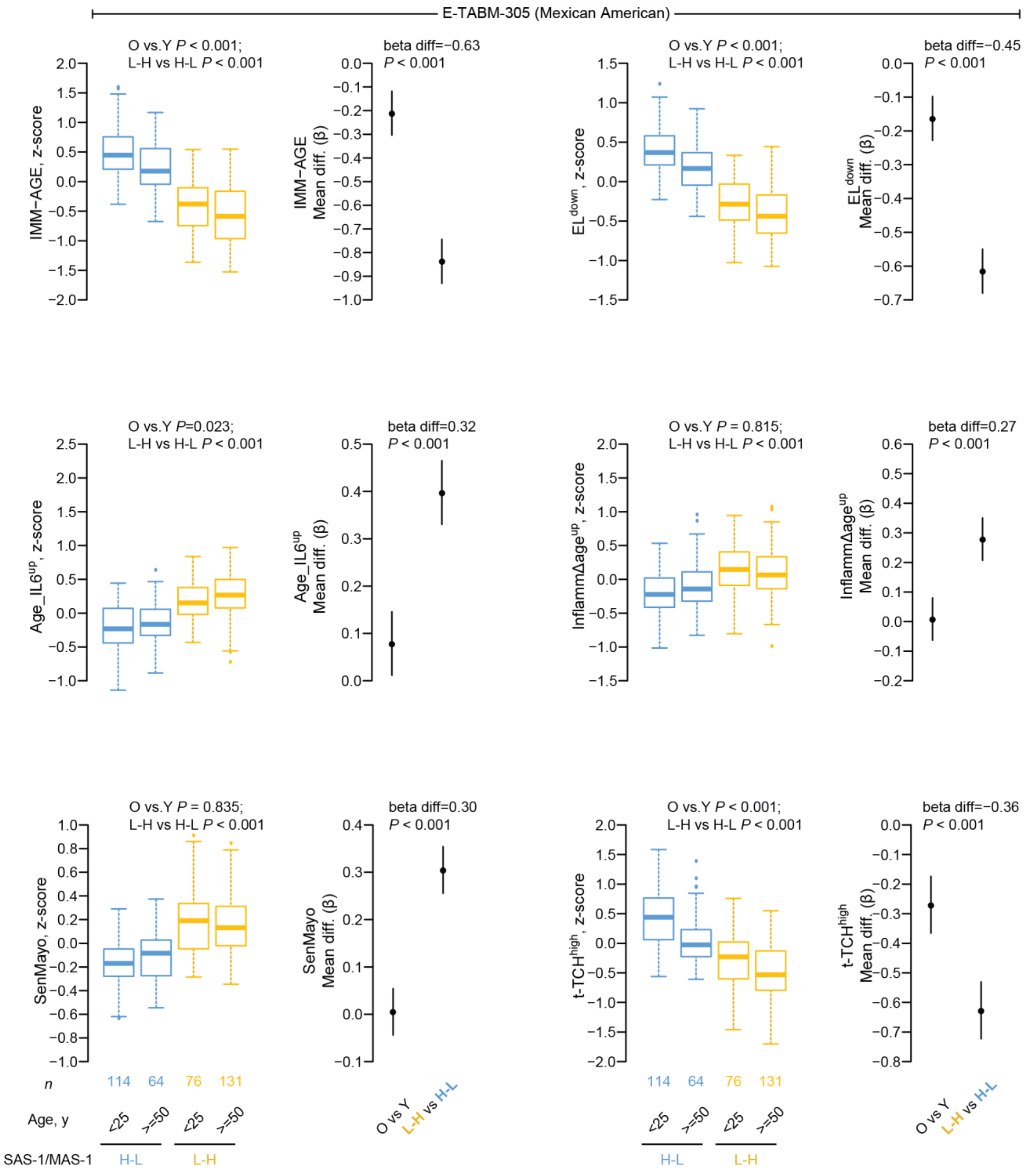

2710

Figure S7c (corresponds to main Figure 3C right panel)

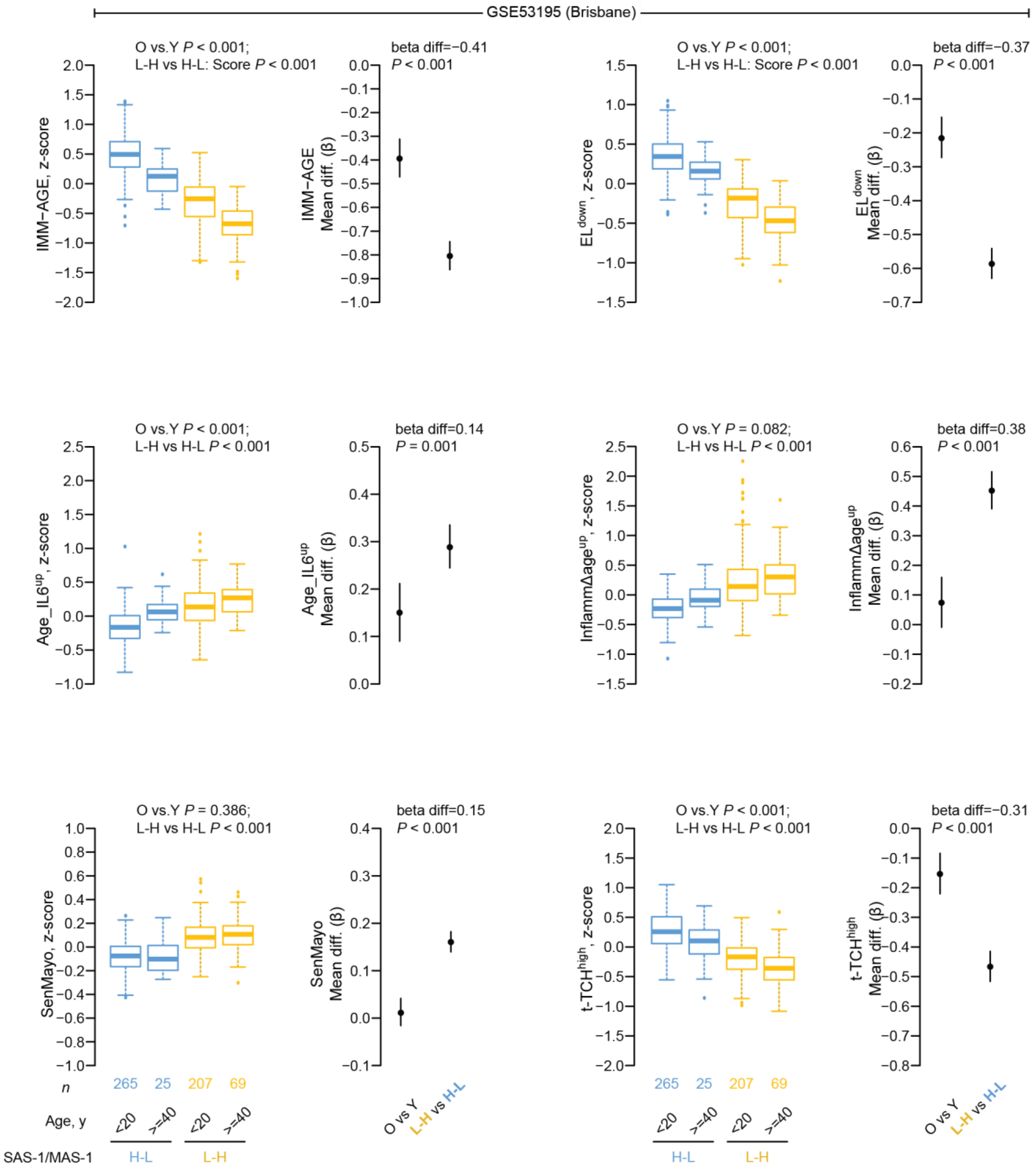

Figure S8. Association of immune resilience (IR) metrics, gene signatures tracking the pathogenic triad and interferon-stimulated genes (ISG), and *TCF7* expression levels in persons with acute respiratory viral infection in the natural season (GSE63810). Analysis was restricted to those with optimal IR status [SAS-1^{high}-MAS-1^{low} (H-L)] before infection. Line plots (mean ± SEM) of indicated gene signatures (z-scores) and *TCF7* expression levels [normalized gene expression (NGE)] stratified by IR-degraders, IR-reconstituters, and IR-preserver status (key at bottom right). ISG_33 corresponds to an ISG signature. Grp, group. Details of the signatures are in Figure 1d (main) and Table S1. Higher levels of the IMM-AGE signature were computed to signify an association with fewer senescent T-cells (less immune aging and lower mortality; a {+}-salutogenesis readout), as detailed in Section 4.2. Statistical details are in Section 6.2.8. Data correspond to Figure 4c (main).

Figure S8 (corresponds to case 1- main Figure 4c)

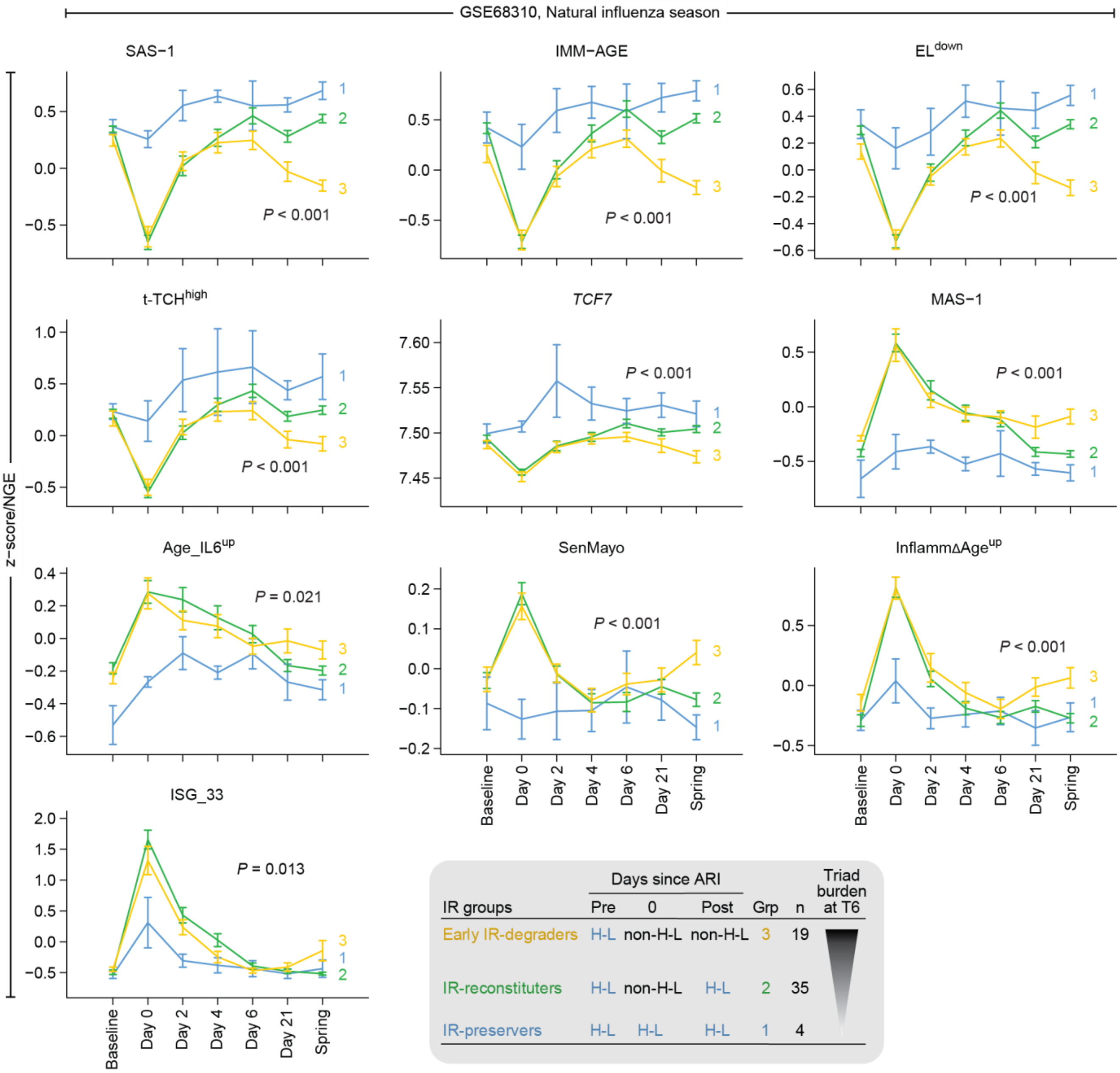

Figure S9. Association of immune resilience (IR) metrics, gene signatures tracking the pathogenic triad, and *TCF7* expression levels by tuberculosis (TB) outcomes. (a-b) Line plots (mean ± SEM) of indicated gene signatures (z-scores) and *TCF7* expression levels (NGE) over the indicated timepoints of TB treatment by **(a)** cure vs. no cure (GSE89403) and **(b)** cure vs. relapse (GSE67589) groups. NGE, normalized gene expression. Details of the signatures are in Figure 1d (main) and Table S1. Higher levels of the IMM-AGE signature were computed to signify an association with fewer senescent T-cells (less immune aging and lower mortality; a {+}-salutogenesis readout), as detailed in Section 4.2. Statistical details are in Section 6.2.9.

Figure S9a: *Mycobacterium tuberculosis*

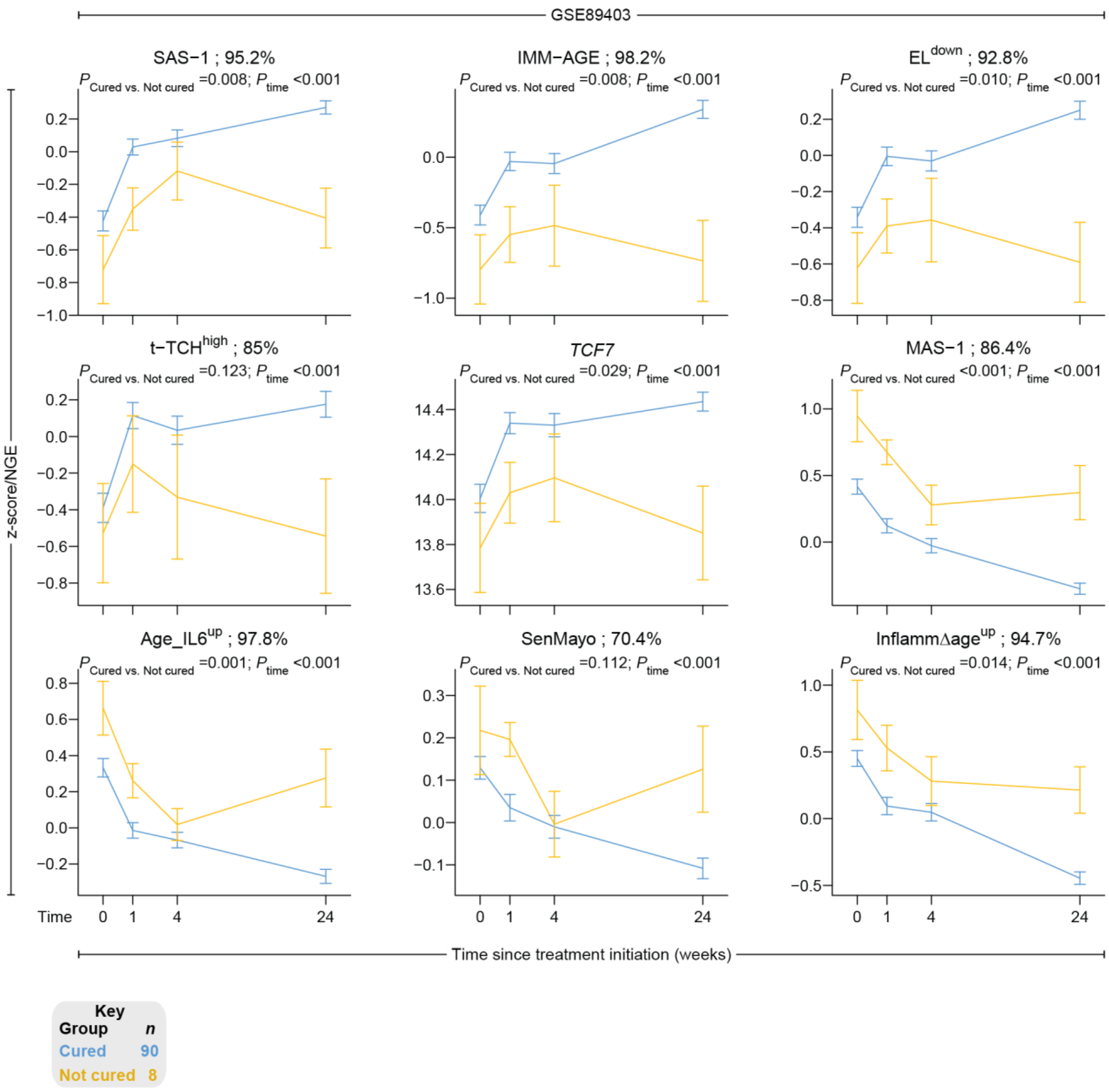

2738

2739

Figure S9b: *Mycobacterium tuberculosis*

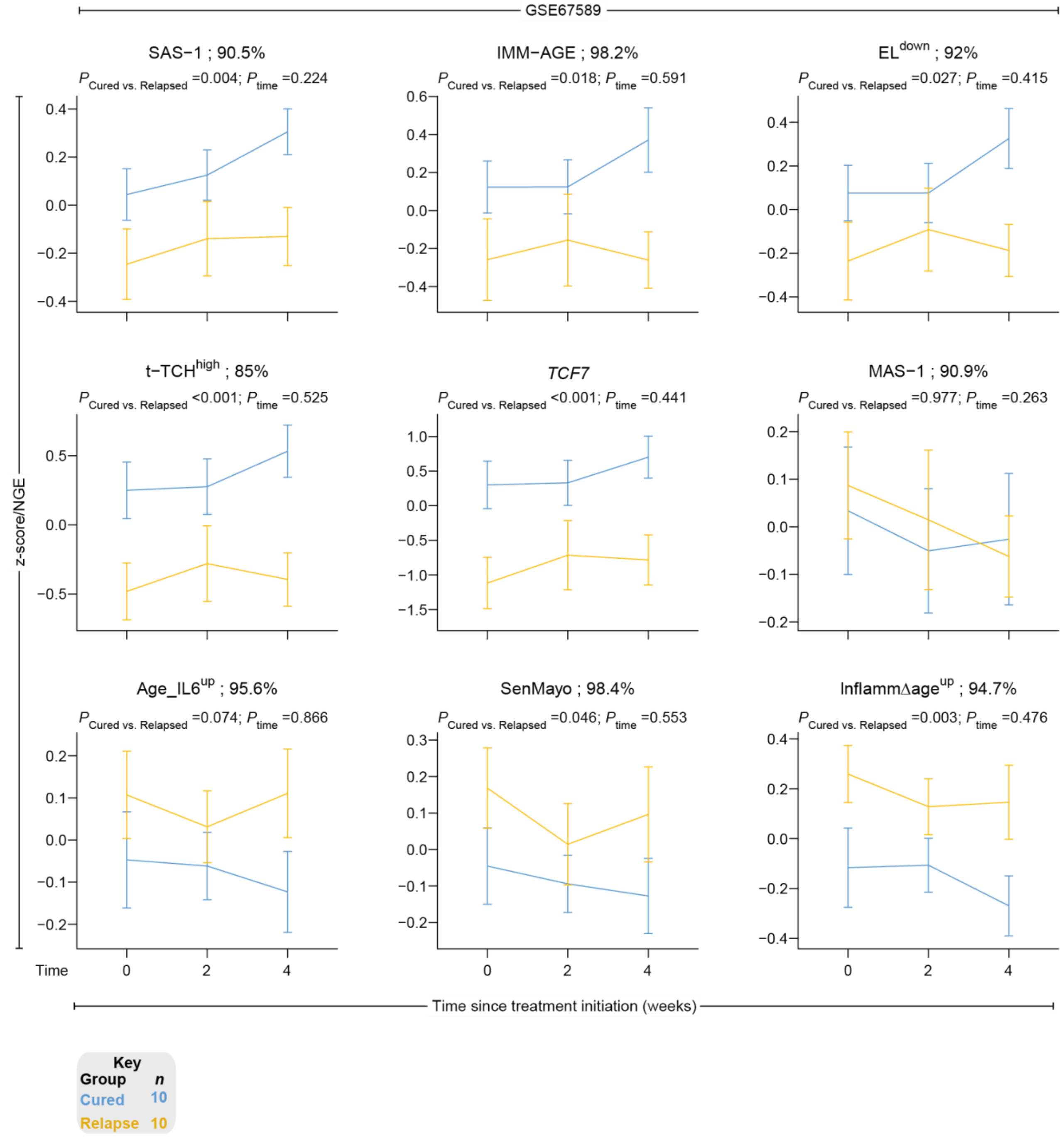

2740

2741

Figure S10. Association of immune resilience (IR) metrics, gene signatures tracking the pathogenic triad, and *TCF7* expression levels in human infection challenge studies. (a) Influenza infection challenge study. Line plots (mean ± SEM) of indicated gene signatures (z-scores) and *TCF7* expression levels (NGE) over the indicated timepoints by SAS-1/MAS-1 profiles and symptom status after human influenza challenge (GSE52428). Data shown for participants with SAS-1^{high}-MAS-1^{low} (H-L) (optimal IR status) who were asymptomatic or symptomatic post challenge, and SAS-1^{low}-MAS-1^{high} (L-H) (extreme IR degradation status) before infection who were symptomatic post challenge. <u>Note:</u> data from two participants with asymptomatic L-H status are not included in the plot. **(b)** Stacked barplots depict distribution of SAS-1/MAS-1 profiles and dot-and-line plots depict median (IQR) of *TCF7* (NGE) over time by infection and control groups in the *Salmonella typhi* challenge study cohort (E-MTAB-3423). NGE, normalized gene expression. *Left*, persons who developed symptomatic typhoid disease (TD) post challenge. *Right*, persons who did not develop TD post challenge. Details of the signatures are in Figure 1d (main) and Table S1. Higher levels of the IMM-AGE signature were computed to signify an association with fewer senescent T-cells (less immune aging and lower mortality; a {+}-salutogenesis readout), as detailed in Section 4.2. Statistical details are in Section 6.2.10. Data in Figure S10a correspond to Figure 4f (main).

Figure S10a: Influenza challenge study (corresponds to case 2- main Figure 4f)

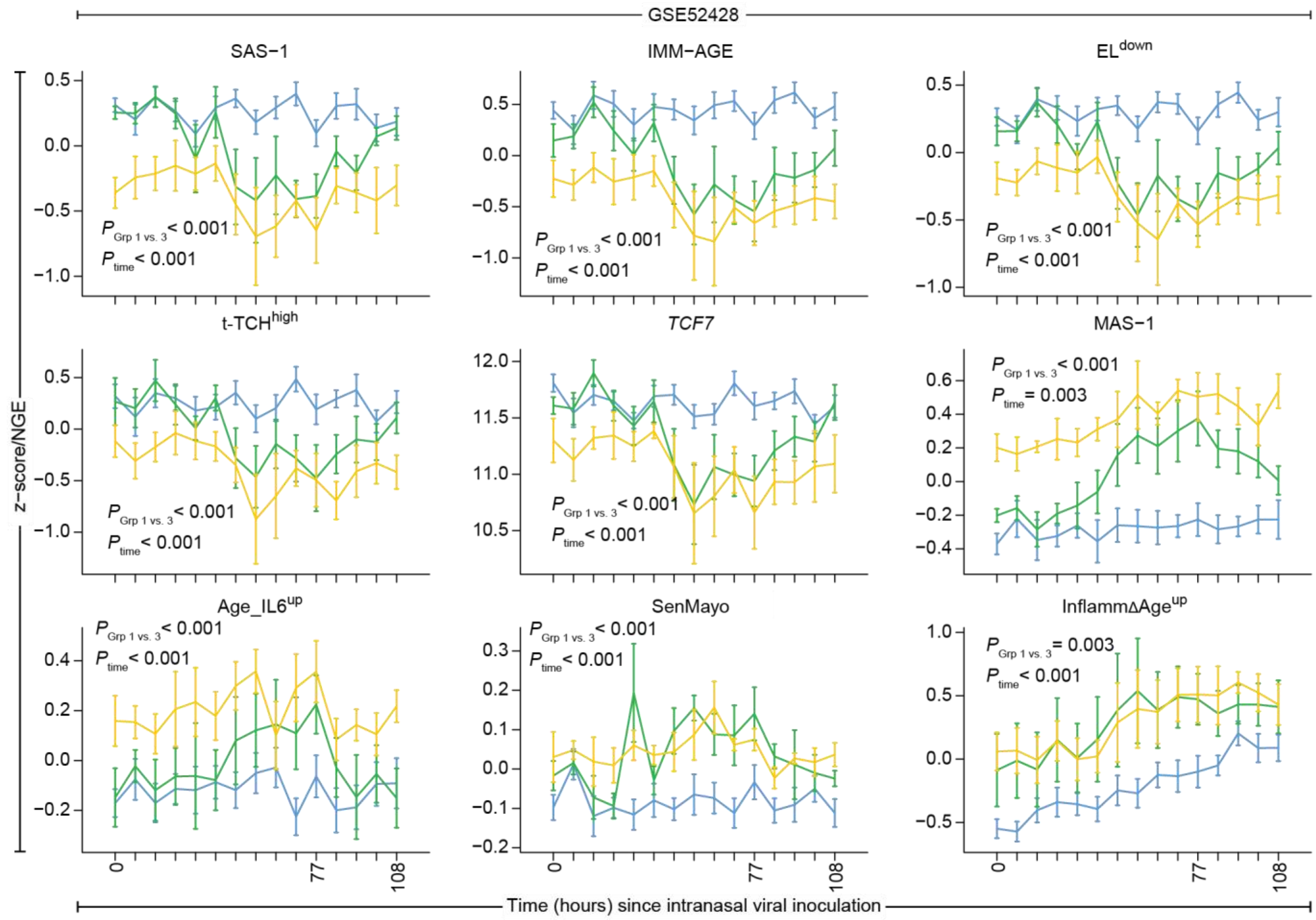

Key for Figure S10a

Group	SAS-1	MAS-1	*n*
1, Asymptomatic	H	L	10
2, Symptomatic	H	L	7
3, Symptomatic	L	H	6

Figure S10b: *Salmonella typhi* challenge study

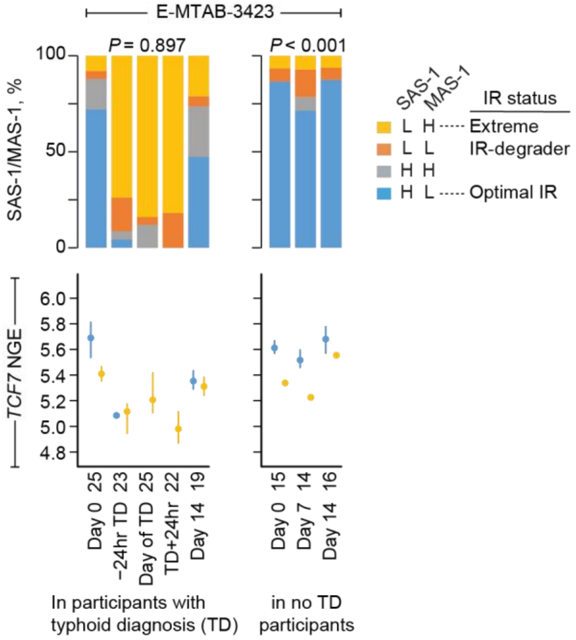

Figure S11. Association of immune resilience (IR) metrics, gene signatures tracking the pathogenic triad and vaccine responses, and *TCF7* expression levels in the CHI consortium influenza vaccine cohort (GSE47353). (a) Stacked barplots depict the distribution of SAS-1/MAS-1 profiles and dot-and-line plots depict median (IQR) of *TCF7* (NGE). *Left*, SAS-1/MAS-1 profiles in the overall cohort at the indicated timepoints from day of receiving vaccine (day 0). Note: Day 1 post-vaccination is associated with an increase in the prevalence of SAS-1^{low}-MAS-1^{high} (extreme IR degrader status) followed by reconstitution of distribution of SAS-1/MAS-1 profiles at day 70 post-vaccination. *Right*, change in the SAS-1/MAS-1 distributions after vaccination in persons whose SAS-1/MAS-1 profiles at baseline (pre-vaccination; day −7) were (*left to right*): SAS-1^{high}-MAS-1^{low} (H-L; optimal IR status); SAS-1^{high}-MAS-1^{high} (H-H); SAS-1^{low}-MAS-1^{low} (L-L); and SAS-1^{low}-MAS-1^{high} (L-H; extreme IR-degrader status). Note: Some persons with SAS-1^{high}-MAS-1^{low} at baseline, failed to reconstitute this profile at day 70 post-vaccination. **(b, c)** Line plots (mean ± SEM) of indicated gene signatures (z-scores) and *TCF7* levels (NGE) over the indicated timepoints **(b)** in the overall cohort and by **(c)** optimal IR (SAS-1^{high}-MAS-1^{low}) vs. extreme IR-degrader (SAS-1^{low}-MAS-1^{high}) status. Included are gene signatures tracking interferon-stimulated genes (ISG_33, ISG_23) and four gene modules tracking vaccine responses (Supplementary section 4.8). NGE, normalized gene expression. Details of the signatures are in Figure 1d (main) and Table S1. Higher levels of the IMM-AGE signature were computed to signify an association with fewer senescent T-cells (less immune aging and lower mortality; a {+}-salutogenesis readout), as detailed in Section 4.2. Statistical details are in Section 6.2.11.

Figure S11a: Influenza vaccination: SAS-1/MAS-1 distributions & *TCF7* levels

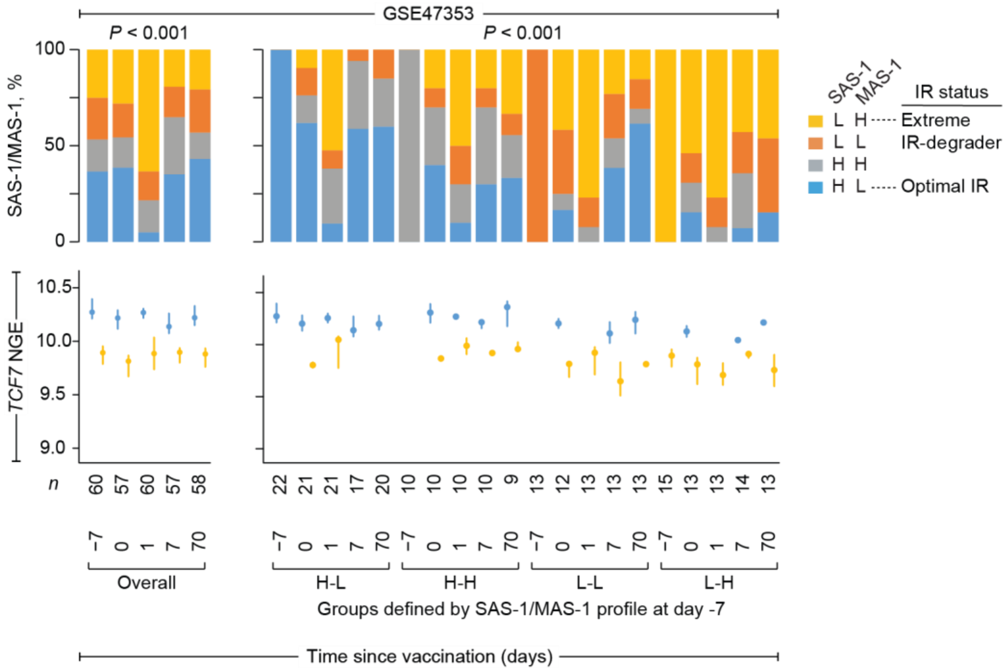

2784

Figure S11b: Influenza vaccination (continued): overall trajectories

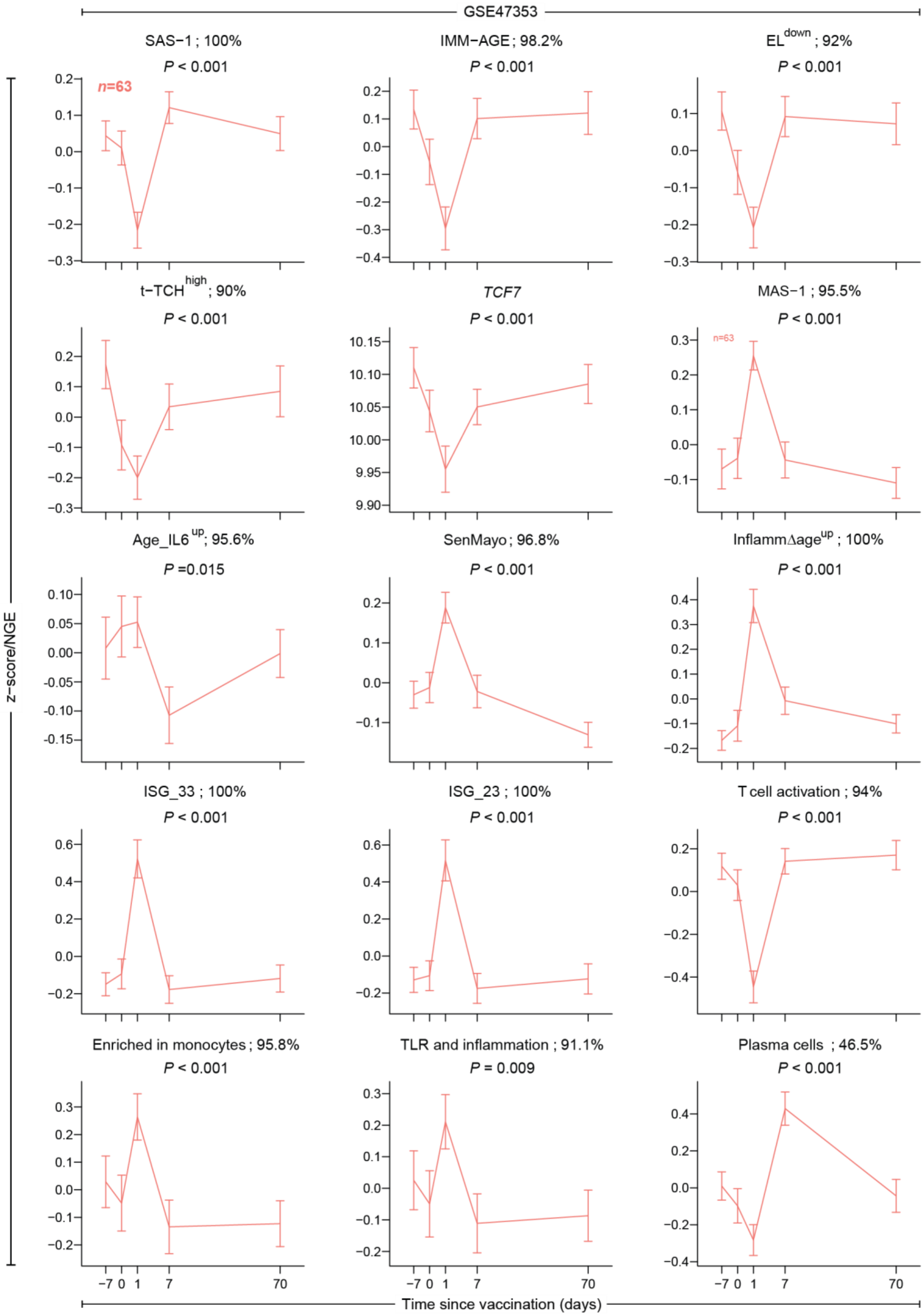

2785

Figure S11c: Influenza vaccination (continued): trajectories by SAS-1^{high}-MAS-1^{low} vs. SAS-1^{low}-MAS-1^{high}

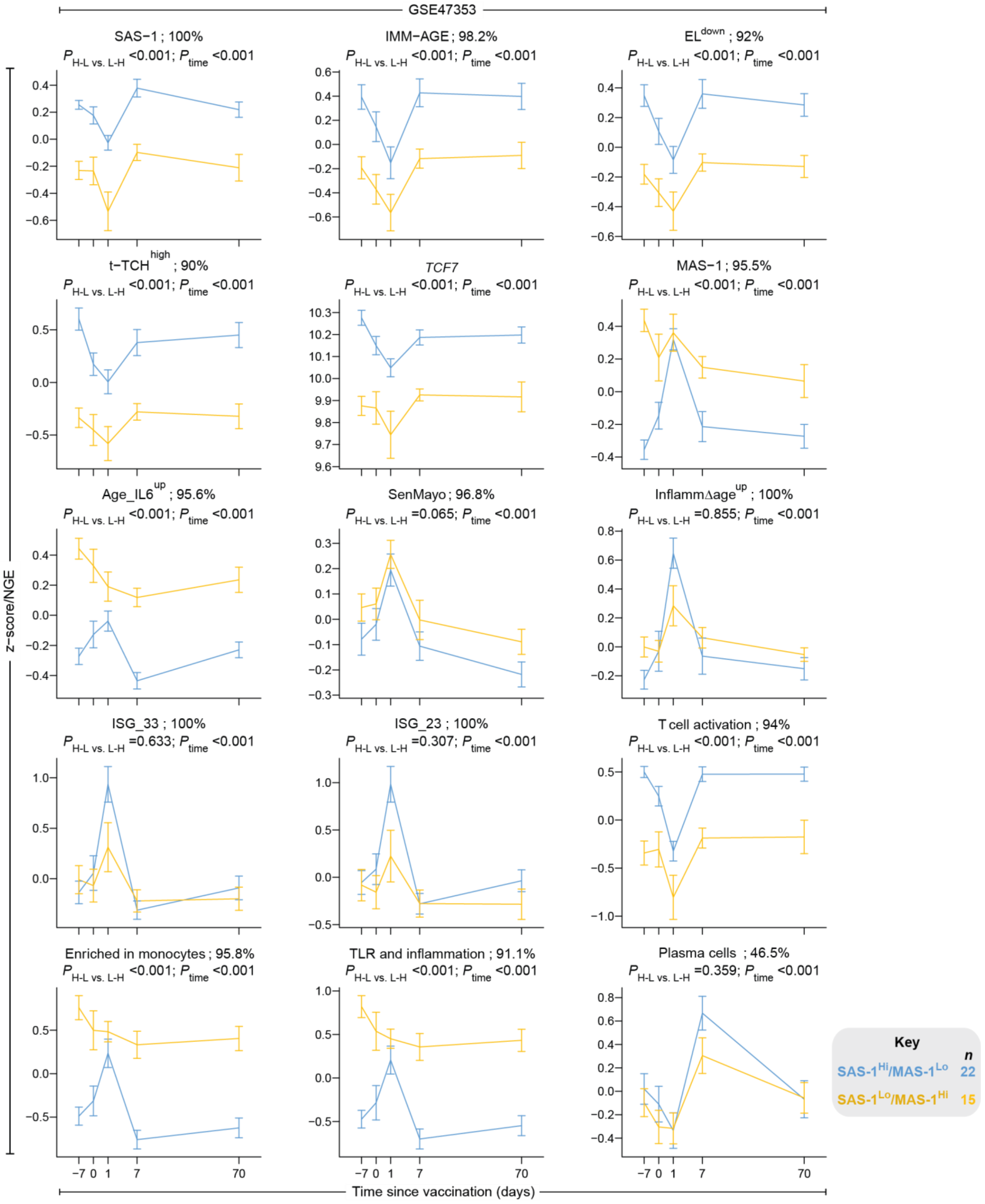

Figure S12. Association of immune resilience (IR) metrics, gene signatures tracking the pathogenic triad and vaccine responses, and *TCF7* expression levels in the Emory trivalent influenza vaccine (TIV) cohort during 2011/12 flu season (GSE74816). (a) Stacked barplots depict the distribution of SAS-1/MAS-1 profiles and dot-and-line plots depict median (IQR) of *TCF7* (NGE). *Left*, SAS-1/MAS-1 profiles in the overall cohort at the indicated timepoints from day of receiving vaccine (day 0). Note: Day 7 post-vaccination is associated with an increase in the prevalence of SAS-1^{low}-MAS-1^{high} (extreme IR-degrader status). The sampling window is not enough to capture reconstitution patterns. *Right*, change in the SAS-1/MAS-1 distributions after vaccination in persons whose SAS-1/MAS-1 profiles at baseline (pre-vaccination) were (*left to right*): SAS-1^{high}-MAS-1^{low} (H-L; optimal IR status); SAS-1^{high}-MAS-1^{high} (H-H); SAS-1^{low}-MAS-1^{low} (L-L); and SAS-1^{low}-MAS-1^{high} (L-H; extreme IR-degrader status). **(b, c)** Line plots (mean ± SEM) of indicated gene signatures (z-scores) and *TCF7* levels (NGE) over the indicated timepoints **(b)** in the overall cohort and by **(c)** optimal IR (SAS-1^{high}-MAS-1^{low}) vs. extreme IR-degrader (SAS-1^{low}-MAS-1^{high}) status. Included are gene signatures tracking interferon-stimulated genes (ISG_33, ISG_23) and four gene modules tracking vaccine responses (Supplementary section 4.8). NGE, normalized gene expression. Details of the signatures are in Figure 1d (main) and Table S1. Higher levels of the IMM-AGE signature were computed to signify an association with fewer senescent T-cells (less immune aging and lower mortality; a {+}-salutogenesis readout), as detailed in Section 4.2. Statistical details are in Section 6.2.12.

Figure S12a: Influenza vaccination: SAS-1/MAS-1 distributions & *TCF7* levels

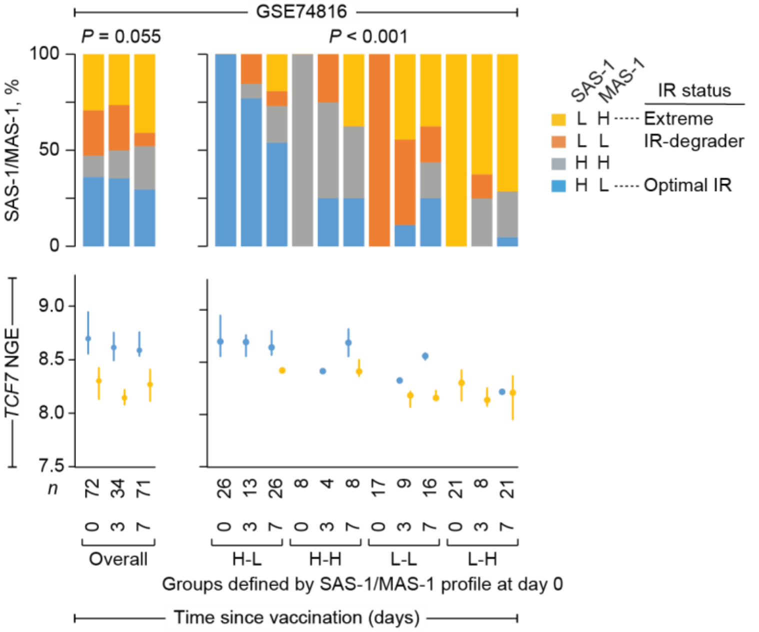

Figure S12b: Influenza vaccination (continued): overall trajectories

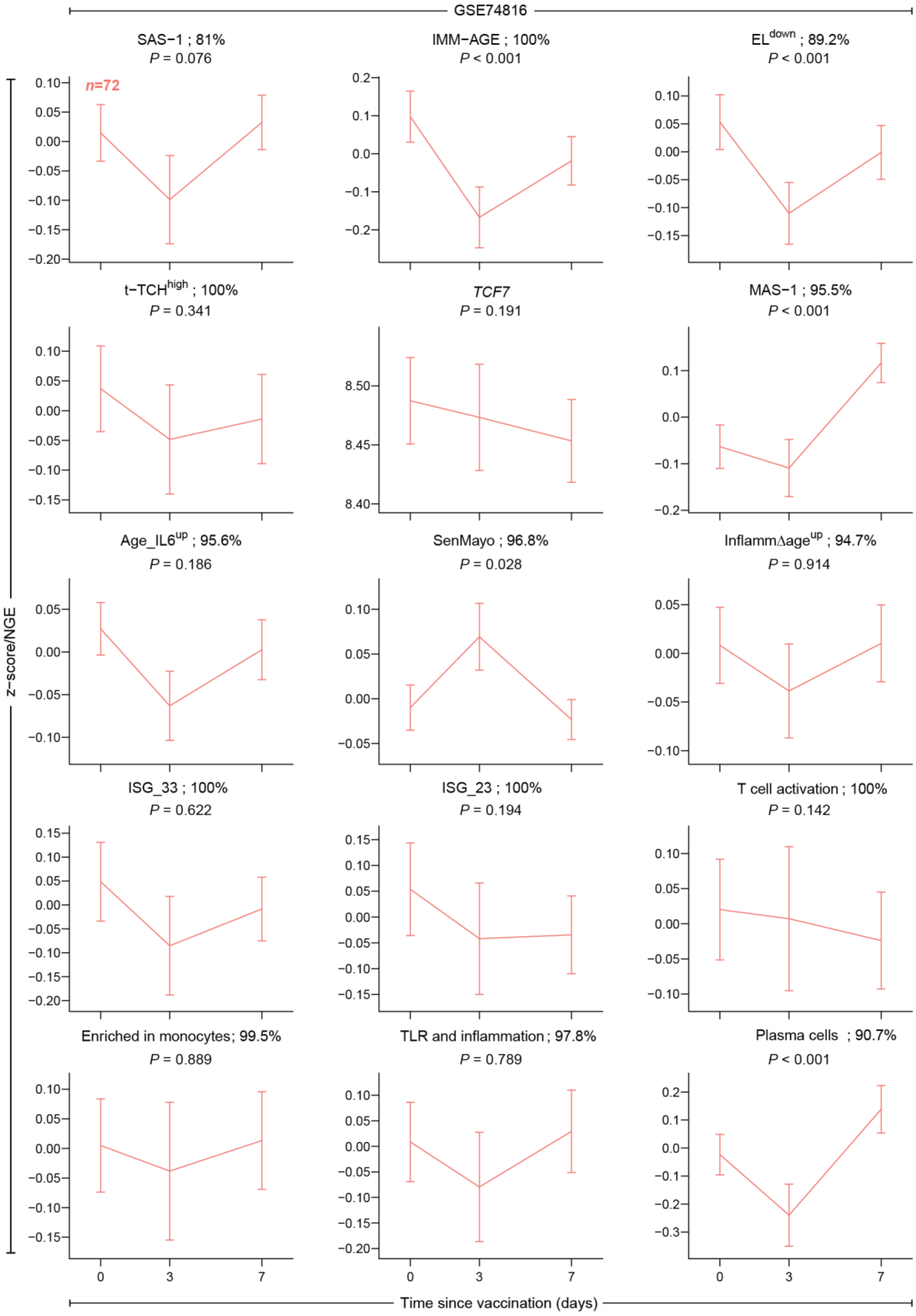

2809

Figure S12c: Influenza vaccination (continued): trajectories by SAS-1^{high}-MAS-1^{low} vs. SAS-1^{low}-MAS-1^{high}

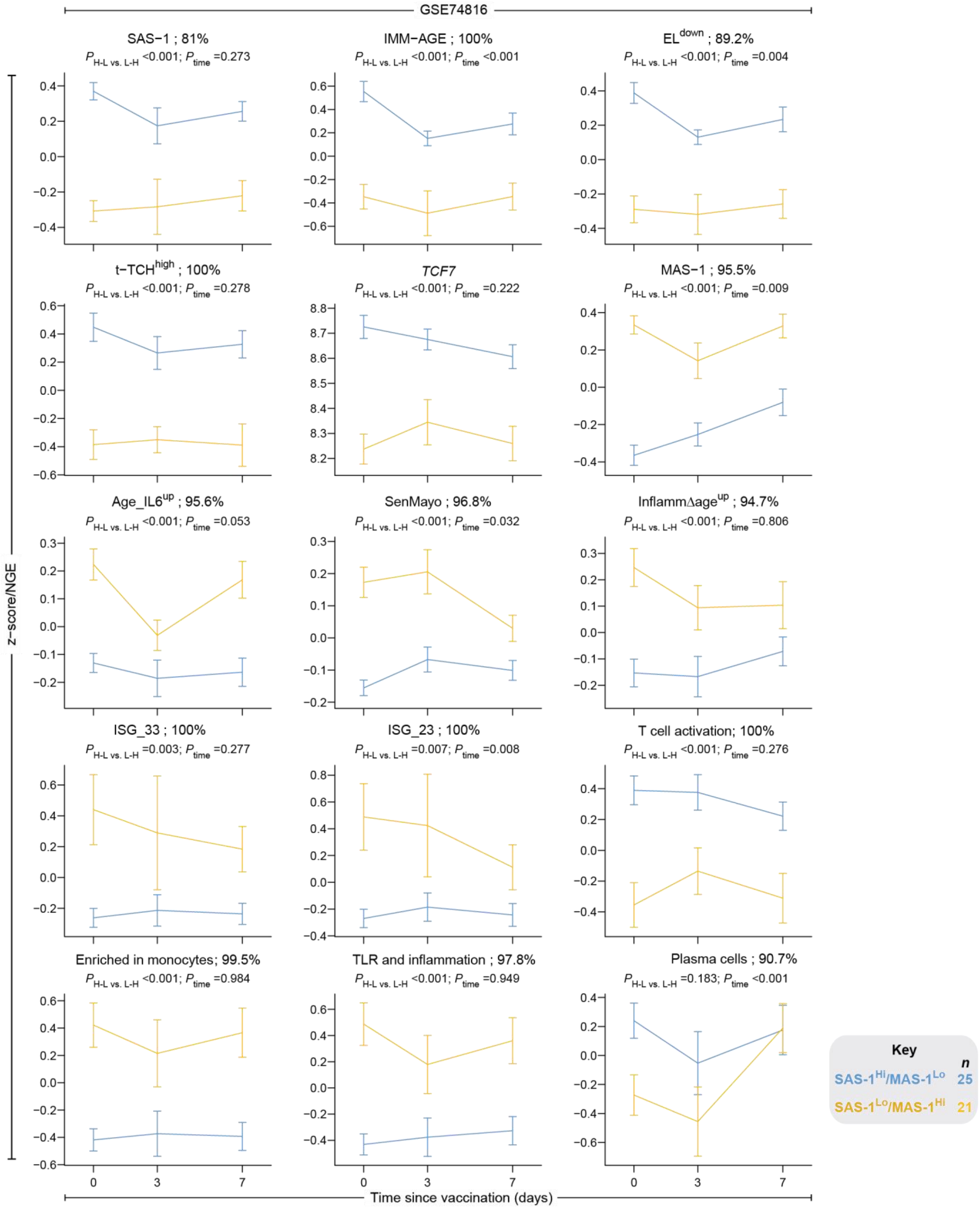

Figure S13. Association of immune resilience (IR) metrics, gene signatures tracking the pathogenic triad and vaccine responses, and *TCF7* expression levels in pregnant women receiving inactivated influenza vaccination (GSE166545). (a) Stacked barplots depict the distribution of SAS-1/MAS-1 profiles and dot-and-line plots depict median (IQR) of *TCF7* (NGE). *Left*, SAS-1/MAS-1 profiles in the overall cohort at the indicated timepoints from day of receiving vaccine (day 0). Note: day 1 post-vaccination is associated with an increase in the prevalence of SAS-1^{low}-MAS-1^{high} (extreme IR- degrader status) followed by reconstitution of the distribution of SAS-1/MAS-1 profiles at day 7 post-vaccination. *Right*, change in the SAS-1/MAS-1 distributions after vaccination in persons whose SAS-1/MAS-1 profiles at baseline (pre-vaccination) were (*left to right*): SAS-1^{high}-MAS-1^{low} (H-L; optimal IR status); SAS-1^{high}-MAS-1^{high} (H-H); SAS-1^{low}-MAS-1^{low} (L-L); and SAS-1^{low}-MAS-1^{high} (L-H; extreme IR-degrader status). Note: Some persons with SAS-1^{high}-MAS-1^{low} at baseline failed to reconstitute this profile at day 7 post vaccination. **(b, c)** Line plots (mean ± SEM) of indicated gene signatures (z-scores) and *TCF7* levels (NGE) over the indicated timepoints **(b)** in the overall cohort and by **(c)** optimal IR (SAS-1^{high}-MAS-1^{low}) vs. extreme IR-degrader (SAS-1^{low}-MAS-1^{high}) status. Included are gene signatures tracking interferon-stimulated genes (ISG_33, ISG_23) and four gene modules tracking vaccine responses (Supplementary section 4.8). NGE, normalized gene expression. Details of the signatures are in Figure 1d (main) and Table S1. Higher levels of the IMM-AGE signature were computed to signify an association with fewer senescent T-cells (less immune aging and lower mortality; a {+}-salutogenesis readout), as detailed in Section 4.2. Statistical details are in Section 6.2.13.

Figure S13a: Influenza vaccination: SAS-1/MAS-1 distributions & *TCF7* levels

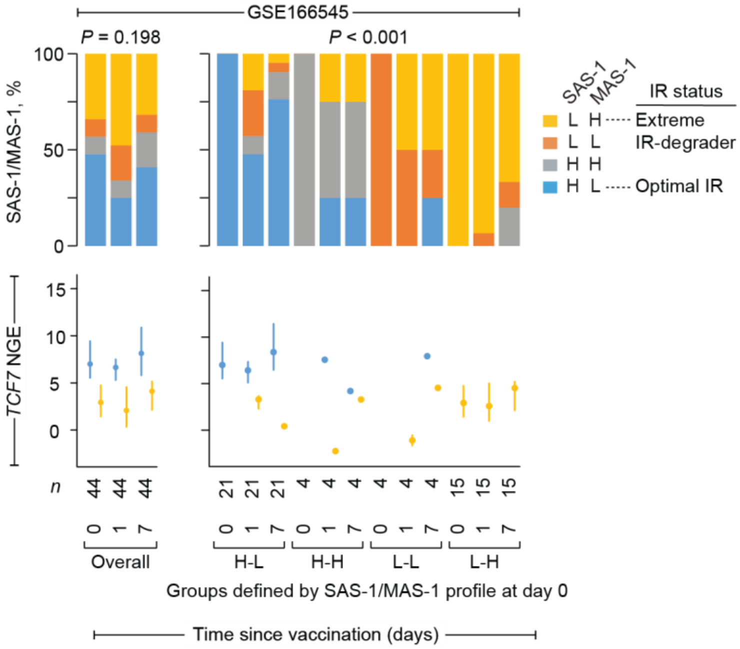

Figure S13b: Influenza vaccination (continued): overall trajectories

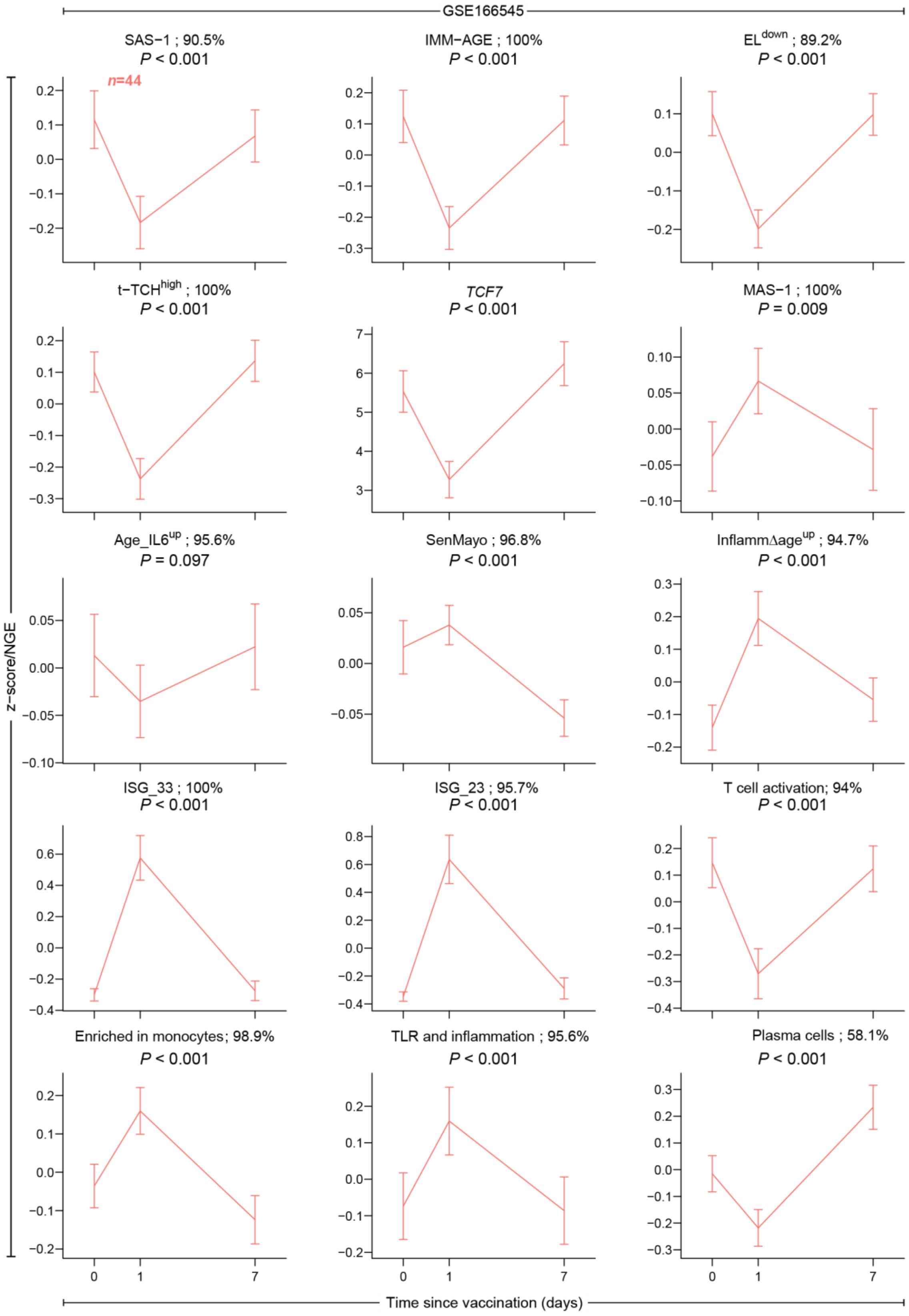

2835

Figure S13c: Influenza vaccination (continued): trajectories by SAS-1^high^-MAS-1^low^ vs. SAS-1^low^-MAS-1^high^

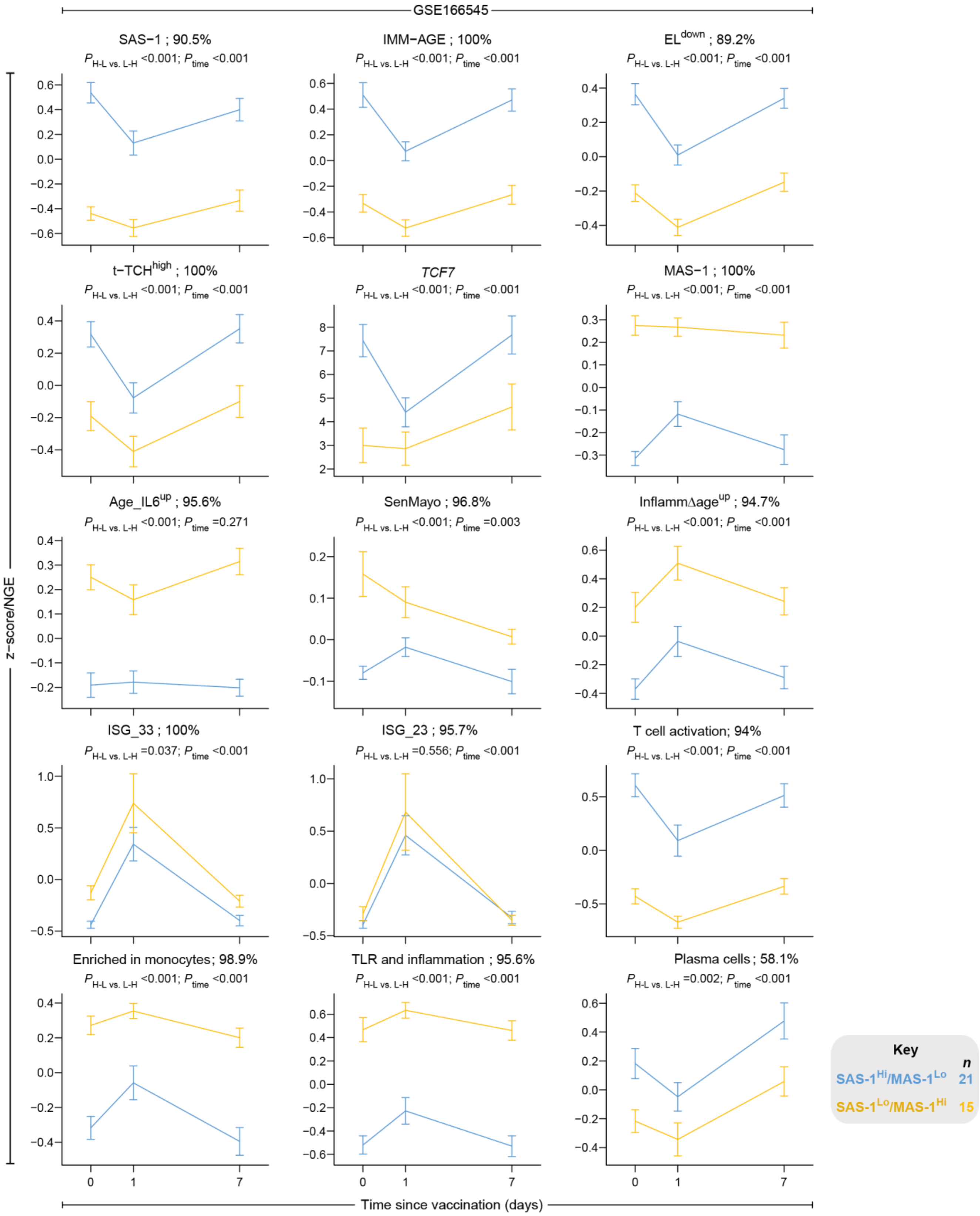

Figure S14. Association of immune resilience (IR) metrics, gene signatures tracking the pathogenic triad and vaccine responses, and *TCF7* expression levels in the H1N1 swine flu vaccine cohort (E-MTAB-2313). (a) Stacked barplots depict the distribution of SAS-1/MAS-1 profiles and dot-and-line plots depict median (IQR) of *TCF7* (NGE). *Left*, SAS-1/MAS-1 profiles in the overall cohort at the indicated timepoints from day of receiving vaccine (day 0). Note day 1 post-vaccination is associated with an increase in the prevalence of the SAS-1^{low}-MAS-1^{high} combination (extreme IR degrader status) followed by reconstitution of SAS-1/MAS-1 profiles at day 7 post-vaccination. *Right*, change in the SAS-1/MAS-1 distributions after vaccination in persons whose SAS-1/MAS-1 profiles at baseline (pre-vaccination, day 0) were (*left to right*): SAS-1^{high}-MAS-1^{low} (H-L; optimal IR status); SAS-1^{high}-MAS-1^{high} (H-H); SAS-1^{low}-MAS-1^{low} (L-L); and SAS-1^{low}-MAS-1^{high} (L-H; extreme IR degrader status). Note: Some persons with SAS-1^{high}-MAS-1^{low} at baseline failed to reconstitute this profile at day 7 post-vaccination. **(b, c)** Line plots (mean ± SEM) of indicated gene signatures (z-scores) and *TCF7* levels (NGE) over the indicated timepoints **(b)** in the overall cohort and by **(c)** optimal IR (SAS-1^{high}-MAS-1^{low}) vs. extreme IR-degrader (SAS-1^{low}-MAS-1^{high}) status. Included are gene signatures tracking interferon-stimulated genes (ISG_33, ISG_23) and four gene modules tracking vaccine responses (Supplementary section 4.8). NGE, normalized gene expression. Details of the signatures are in Figure 1d (main) and Table S1. Higher levels of the IMM-AGE signature were computed to signify an association with fewer senescent T-cells (less immune aging and lower mortality; a {+}-salutogenesis readout), as detailed in Section 4.2. Statistical details in Section 6.2.14.

Figure S14a: Influenza vaccination: SAS-1/MAS-1 distributions & *TCF7* levels

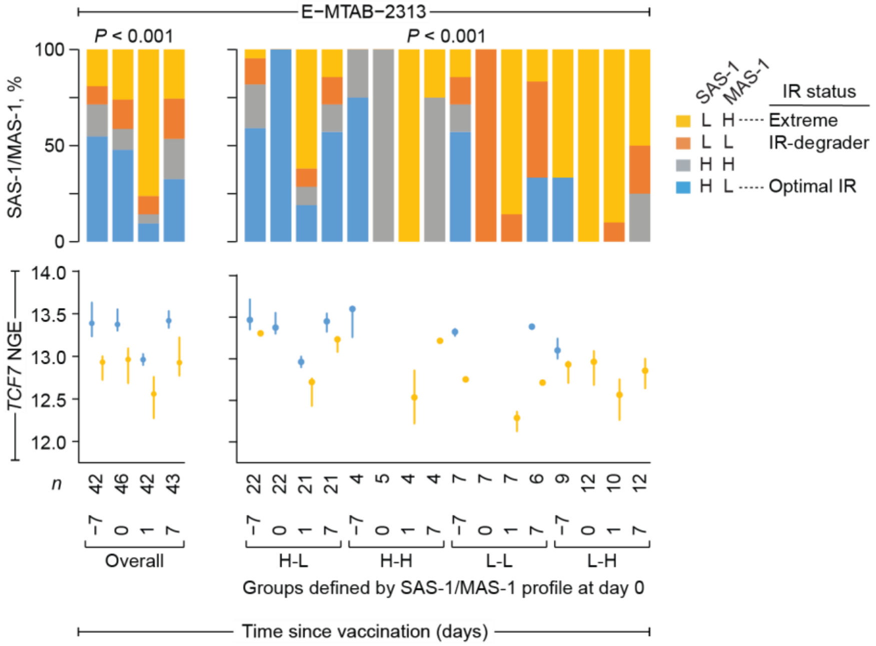

Figure S14b: Influenza vaccination (continued): overall trajectories

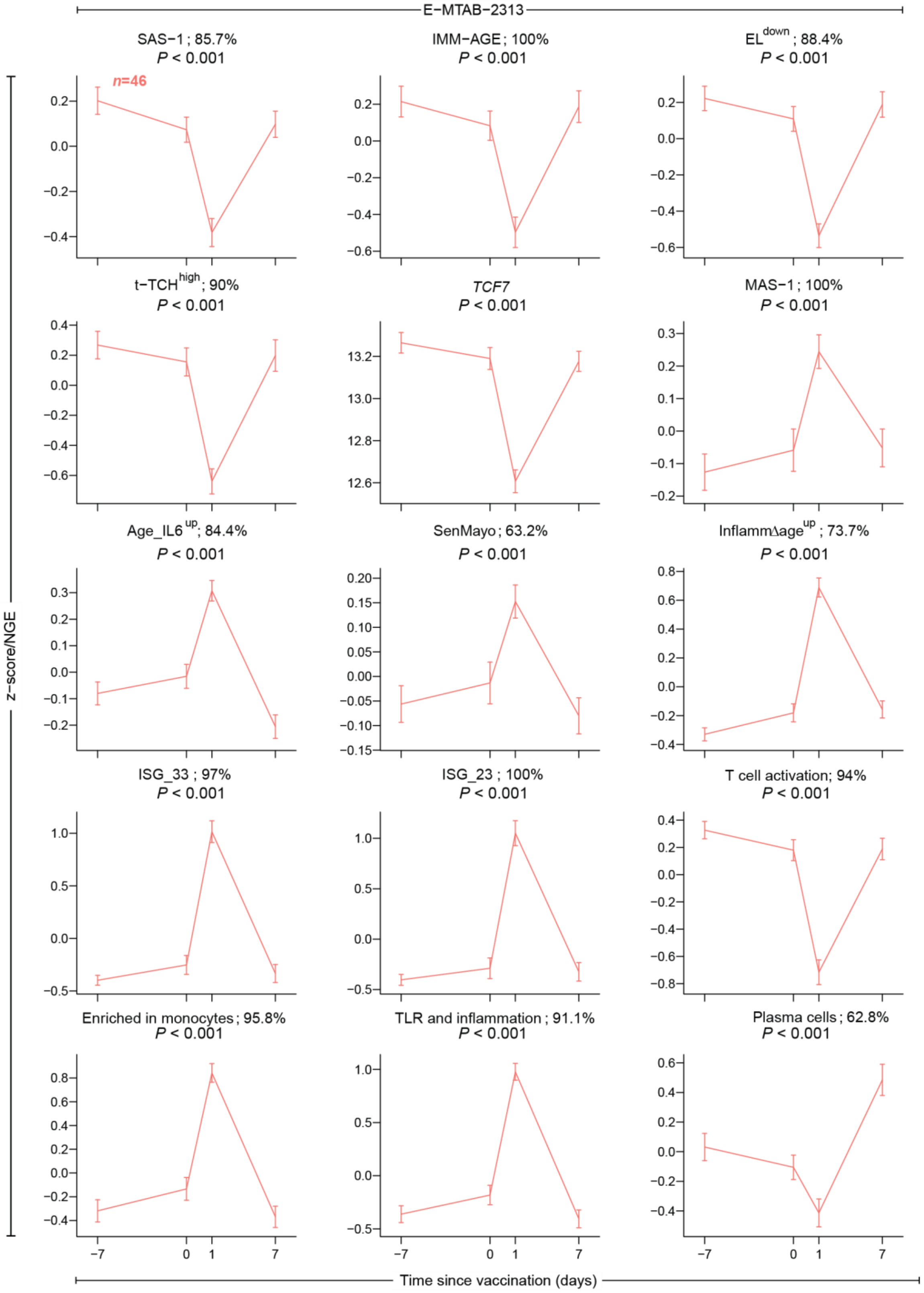

2860

Figure S14c: Influenza vaccination (continued): trajectories by SAS-1^{high}-MAS-1^{low} vs. SAS-1^{low}-MAS-1^{high}

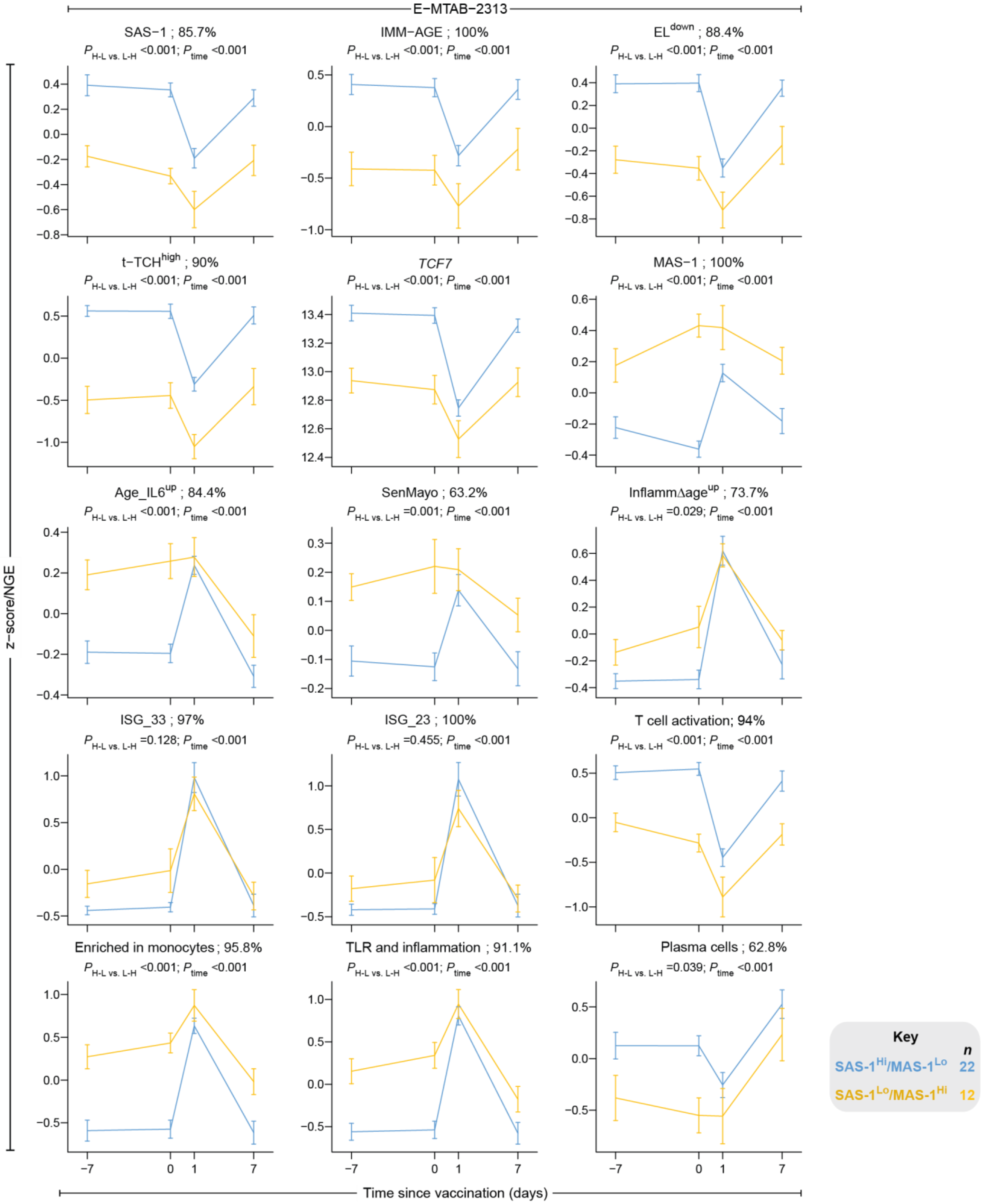

Figure S15. Association of immune resilience (IR) metrics, gene signatures tracking the pathogenic triad and vaccine responses, and *TCF7* expression levels in the fluzone trivalent influenza vaccine (TIV) cohort during 2011/12 flu season (SDY296). (a) Stacked barplots depict the distribution of SAS-1/MAS-1 profiles and dot-and-line plots depict median (IQR) of *TCF7* (NGE). *Left*, SAS-1/MAS-1 profiles in the overall cohort at the indicated timepoints from day of receiving vaccine (day 0). Note: Day 1 post-vaccination is associated with an increase in the prevalence of the SAS-1^{low}-MAS-1^{high} combination (extreme IR-degrader status) followed by reconstitution of SAS-1/MAS-1 profiles at day 7 post-vaccination. *Right*, change in the SAS-1/MAS-1 distributions after vaccination in persons whose SAS-1/MAS-1 profiles at baseline (pre-vaccination, day -7) were (*left to right*): SAS-1^{high}-MAS-1^{low} (H-L; optimal IR status); SAS-1^{high}-MAS-1^{high} (H-H); SAS-1^{low}-MAS-1^{low} (L-L); and SAS-1^{low}-MAS-1^{high} (L-H; extreme IR-degrader status). Note the switch from SAS-1^{high}-MAS-1^{low} to non-SAS-1^{high}-MAS-1^{low} status at day 1 post-vaccination, and some persons with SAS-1^{high}-MAS-1^{low} at baseline failed to reconstitute this profile at day 28 post-vaccination. **(b, c)** Line plots (mean ± SEM) of indicated gene signatures (z-scores) and *TCF7* levels (NGE) over the indicated timepoints **(b)** in the overall cohort and by **(c)** optimal IR (SAS-1^{high}-MAS-1^{low}) vs. extreme IR-degrader (SAS-1^{low}-MAS-1^{high}) status. Included are gene signatures tracking interferon-stimulated genes (ISG_33, ISG_23) and four gene modules tracking vaccine responses (Supplementary section 4.8). NGE, normalized gene expression. Details of the signatures are in Figure 1d (main) and Table S1. Higher levels of the IMM-AGE signature were computed to signify an association with fewer senescent T-cells (less immune aging and lower mortality; a {+}-salutogenesis readout), as detailed in Section 4.2. Statistical details in Section 6.2.15.

Figure S15a: Influenza vaccination: SAS-1/MAS-1 distributions & *TCF7* levels

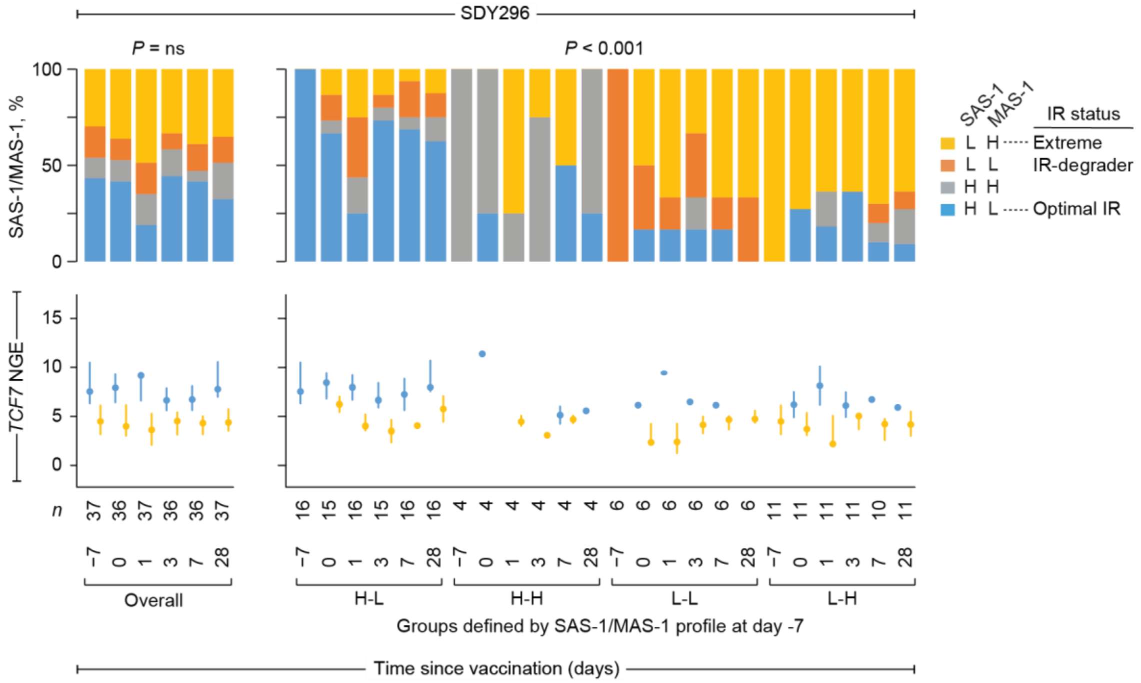

2886

Figure S15b (continued): overall trajectories

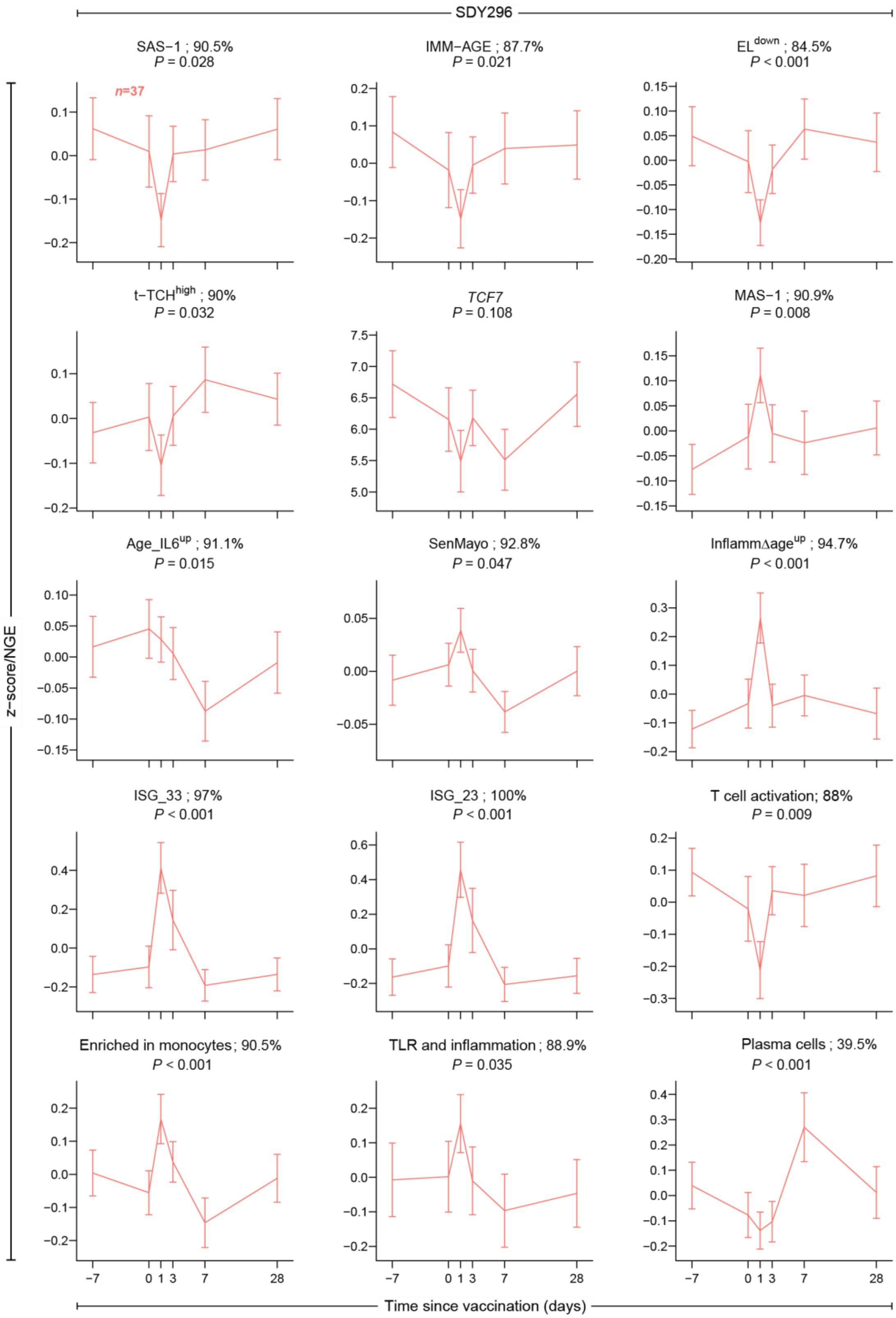

2887

Figure S15c: Influenza vaccination (continued): trajectories by SAS-1^{high}-MAS-1^{low} vs. SAS-1^{low}-MAS-1^{high}

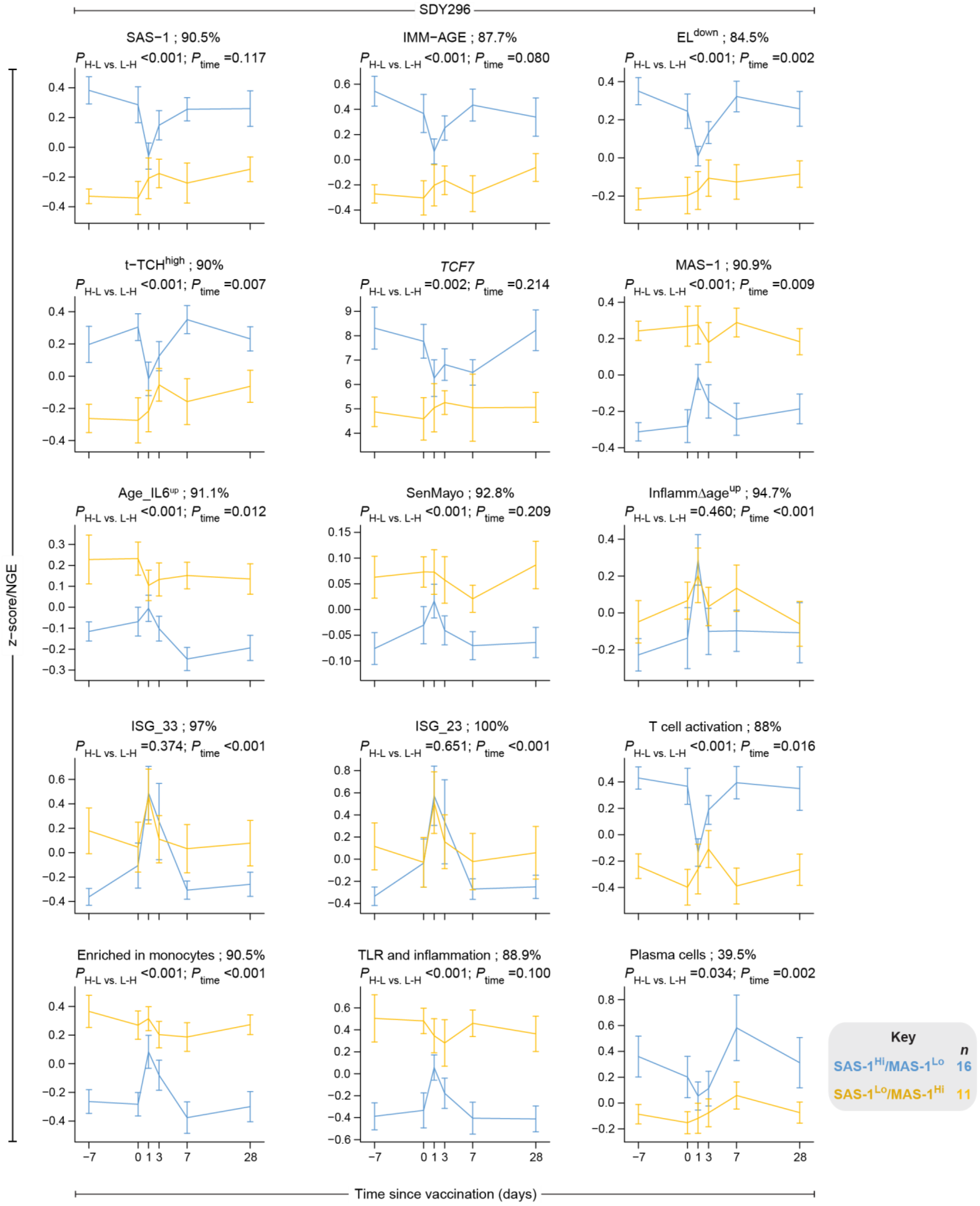

2888

2889

Figure S16. Association of immune resilience (IR) metrics, gene signatures tracking the pathogenic triad and vaccine responses, and *TCF7* expression levels in older participants (age 50-74 years] receiving trivalent influenza vaccination (TIV) during 2010/11 flu season (SDY67). (a) Stacked barplots depict the distribution of SAS-1/MAS-1 profiles and dot-and-line plots depict median (IQR) of *TCF7* (NGE). *Left*, SAS-1/MAS-1 profiles in the overall cohort at the indicated timepoints from day of receiving vaccine (day 0). *Middle*, distributions of SAS-1/MAS-1 profiles after vaccination in persons whose SAS-1/MAS-1 profiles at baseline (pre-vaccination, day 0) were (*left to right*): SAS-1^{high}-MAS-1^{low} (H-L; optimal IR status); SAS-1^{high}-MAS-1^{high} (H-H); SAS-1^{low}-MAS-1^{low} (L-L); and SAS-1^{low}-MAS-1^{high} (L-H; extreme IR-degrader status). Inset: persons with SAS-1^{high}-MAS-1^{low} at baseline (pre-vaccination) were categorized into four IR groups based on their SAS-1/MAS-1 profiles at days 0, 3 and 28 post-vaccination, respectively: IR-preservers (H-L, H-L, H-L), IR-reconstituters (H-L, non-H-L, H-L), late IR-degraders (H-L, H-L, non-H-L), and early IR-degraders (H-L, non-H-L, non-H-L). *Right*, SAS-1/MAS-1 profiles in persons classifying as IR-preservers, IR-reconstituters, and early and late IR-degraders. N, non-SAS-1^{high}-MAS-1^{low} status. **(b, c, d)** Line plots (mean ± SEM) of indicated gene signatures (z-scores) and *TCF7* levels (NGE) over the indicated timepoints **(b)** in the overall cohort, by **(c)** optimal IR (SAS-1^{high}-MAS-1^{low}) vs. extreme IR-degrader (SAS-1^{low}-MAS-1^{high}) status, and **(d)** four IR groups noted in the inset shown in panel A and in the panel key (*bottom right*). In panels to b-d, gene signatures tracking interferon-stimulated genes (ISG_33, ISG_23) and four gene modules associated with vaccine responses (Supplementary section 4.8) are shown. NGE, normalized gene expression. Details of the signatures are in Figure 1d (main) and Table S1. Higher levels of the IMM-AGE signature were computed to signify an association with fewer senescent T-cells (less immune aging and lower mortality; a {+}-salutogenesis readout), as detailed in Section 4.2. Statistical details in Section 6.2.16. Data correspond to Figure 4g (main).

Figure S16a: Influenza vaccination: SAS-1/MAS-1 distributions & *TCF7* levels (corresponds to main Figure 4g left panel)

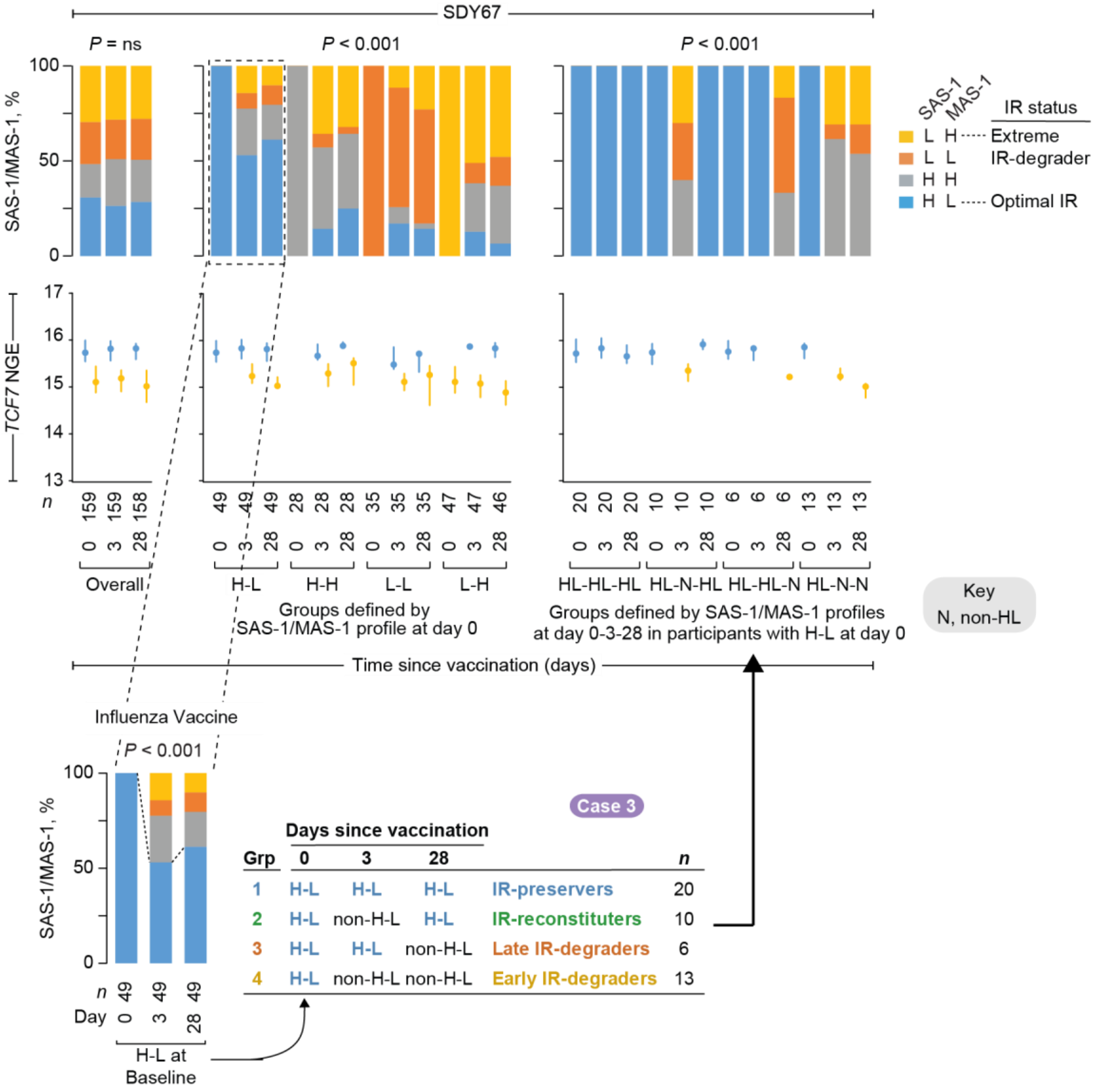

Figure S16b: Influenza vaccination (continued): overall trajectories

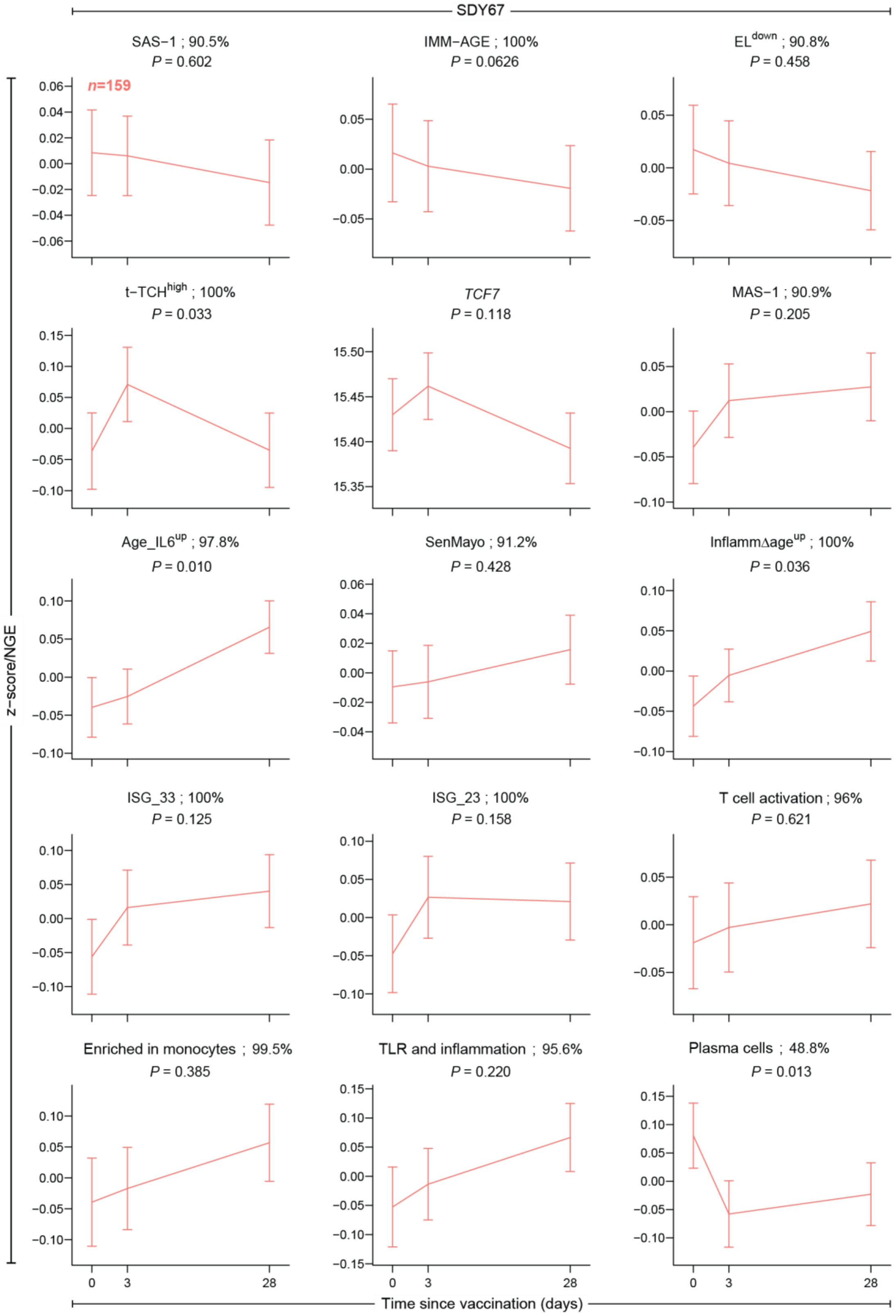

Figure S16c: Influenza vaccination (continued): trajectories by SAS-1^{high}-MAS-1^{low} vs. SAS-1^{low}-MAS-1^{high}

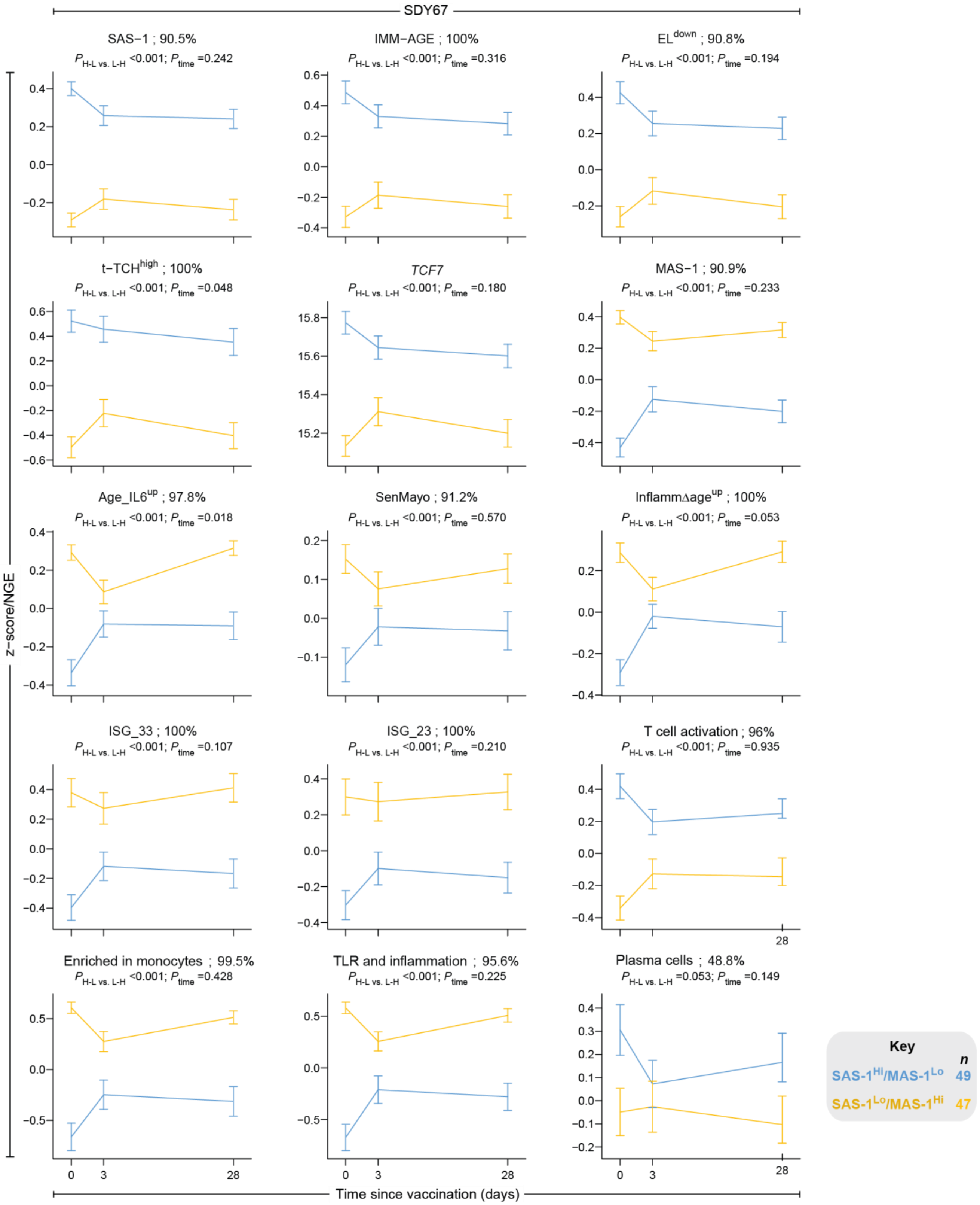

2918

Figure S16d: Influenza vaccination (continued - corresponds to main Figure 4g right panel)

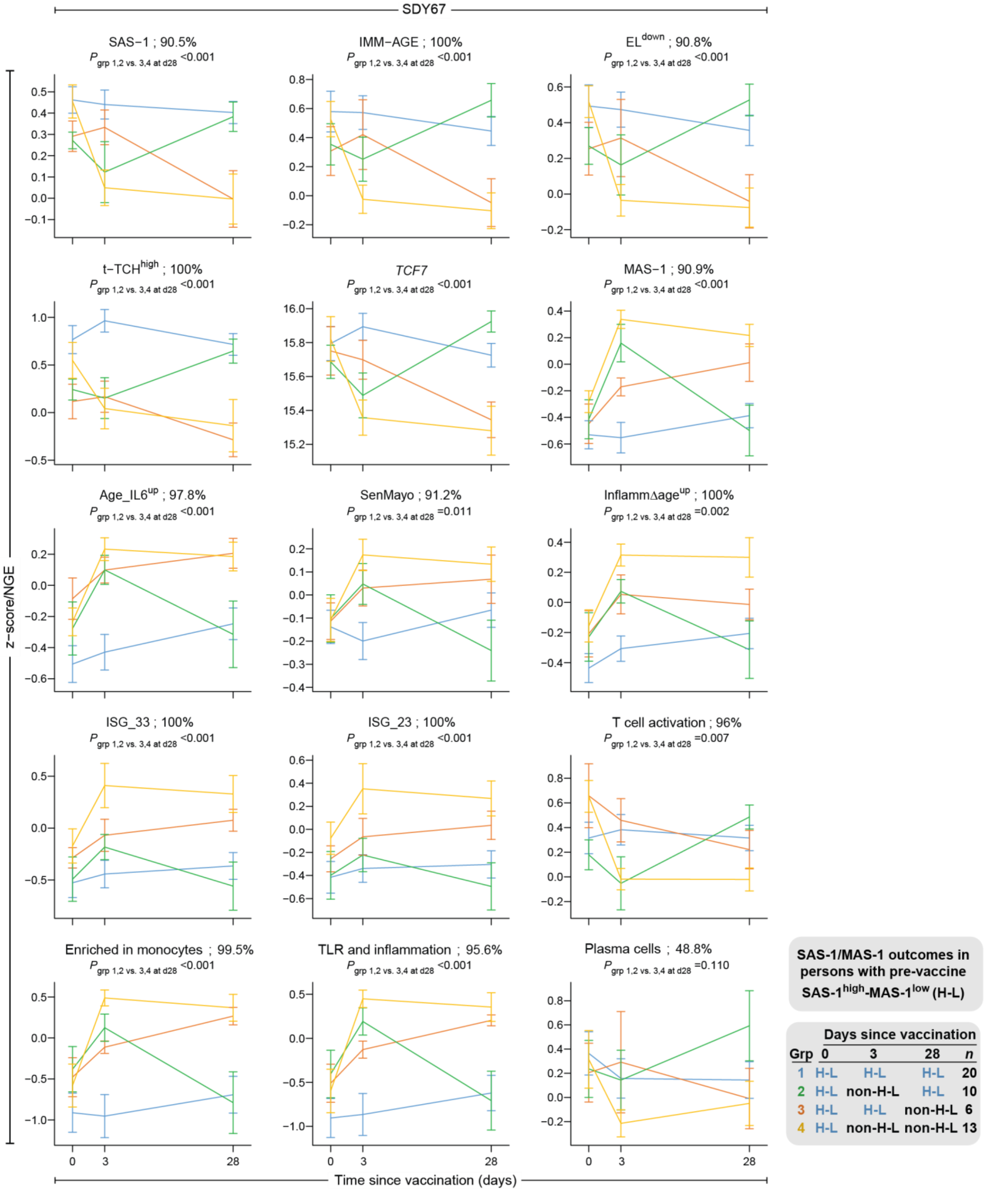

Figure S17. Association of immune resilience (IR) metrics, gene signatures tracking the pathogenic triad and vaccine responses, and *TCF7* expression levels in the Singapore Vaxigrip cohort (GSE107990). (a) Stacked barplots depict the distribution of SAS-1/MAS-1 profiles and dot-and-line plots depict median (IQR) of *TCF7* (NGE). *Left*, SAS-1/MAS-1 profiles in the overall cohort at the indicated time points from the day of receiving vaccine (day 0). *Middle*, distribution of SAS-1/MAS-1 profiles after vaccination in persons whose profiles at baseline (pre-vaccination, day 0) were (*left to right*): SAS-1^{high}-MAS-1^{low} (H-L; optimal IR status); SAS-1^{high}-MAS-1^{high} (H-H); SAS-1^{low}-MAS-1^{low} (L-L); and SAS-1^{low}-MAS-1^{high} (L-H; extreme IR-degrader status). *Right*, SAS-1/MAS-1 profiles in persons with SAS-1^{high}-MAS-1^{low} at baseline (pre-vaccination) who were categorized into four IR groups based on their SAS-1/MAS-1 profiles at days 0, 2 and 28 post-vaccination: IR-preservers (H-L, H-L, H-L), IR-reconstituters (H-L, non-H-L, H-L), late IR-degraders (H-L, H-L, non-H-L), and early IR-degraders (H-L, non-H-L, non-H-L). N, non-SAS-1^{high}-MAS-1^{low}. **(b, c, d)** Line plots (mean ± SEM) of indicated gene signatures (z-scores) and *TCF7* levels (NGE) at the indicated timepoints **(b)** in the overall cohort and by **(c)** optimal IR (SAS-1^{high}-MAS-1^{low}) vs. extreme IR-degrader (SAS-1^{low}-MAS-1^{high}) status. **(d)** Four IR groups in the key shown at the bottom right. In panels b-d, gene signatures tracked interferon-stimulated genes (ISG_33, ISG_23) and four gene modules associated with vaccine responses (Supplementary section 4.8) are shown. NGE, normalized gene expression. Details of the signatures are in Figure 1d (main) and Table S1. Higher levels of the IMM-AGE signature were computed to signify an association with fewer senescent T-cells (less immune aging and lower mortality; a {+}-salutogenesis readout), as detailed in Section 4.2. Statistical details are in Section 6.2.17.

Figure S17a: Influenza vaccination: SAS-1/MAS-1 distributions & *TCF7* levels

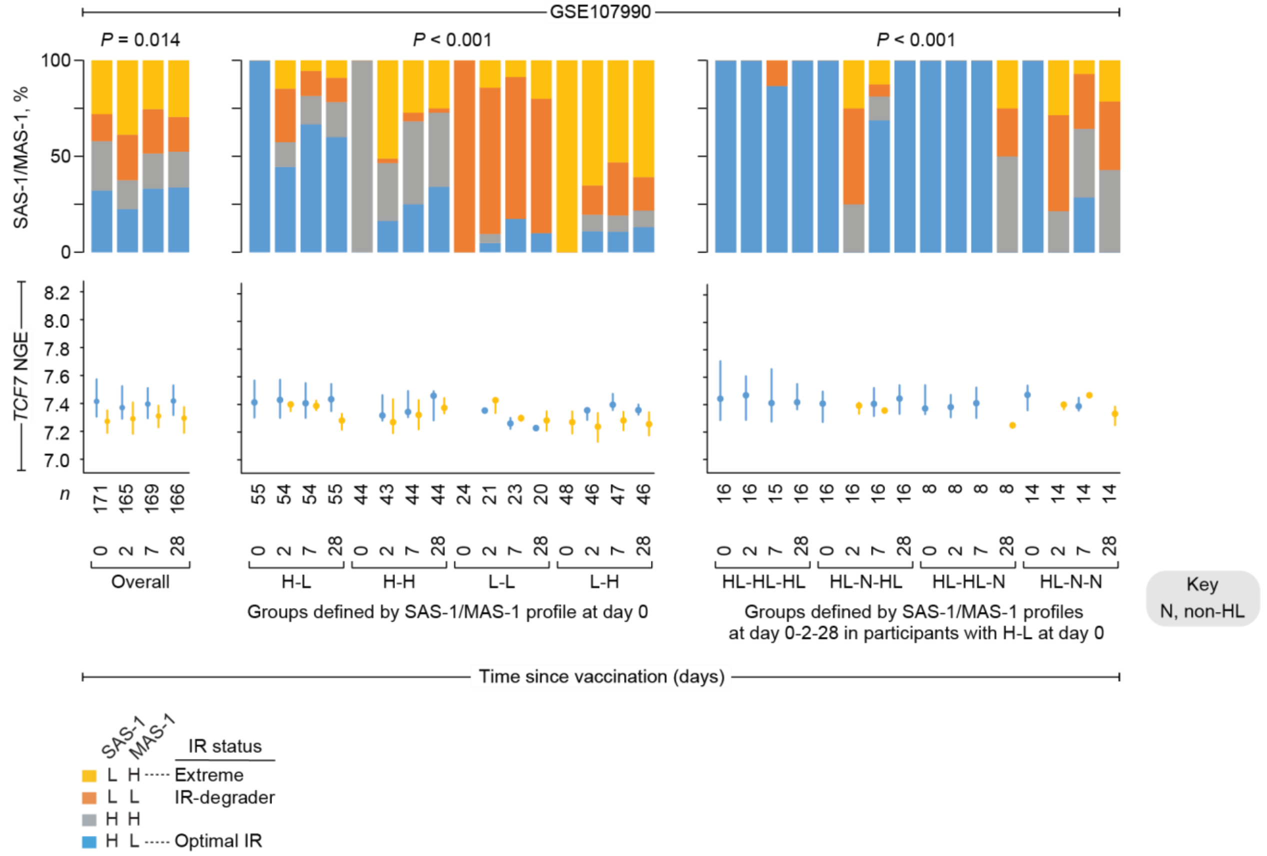

Figure S17b: Influenza vaccination (continued): overall trajectories

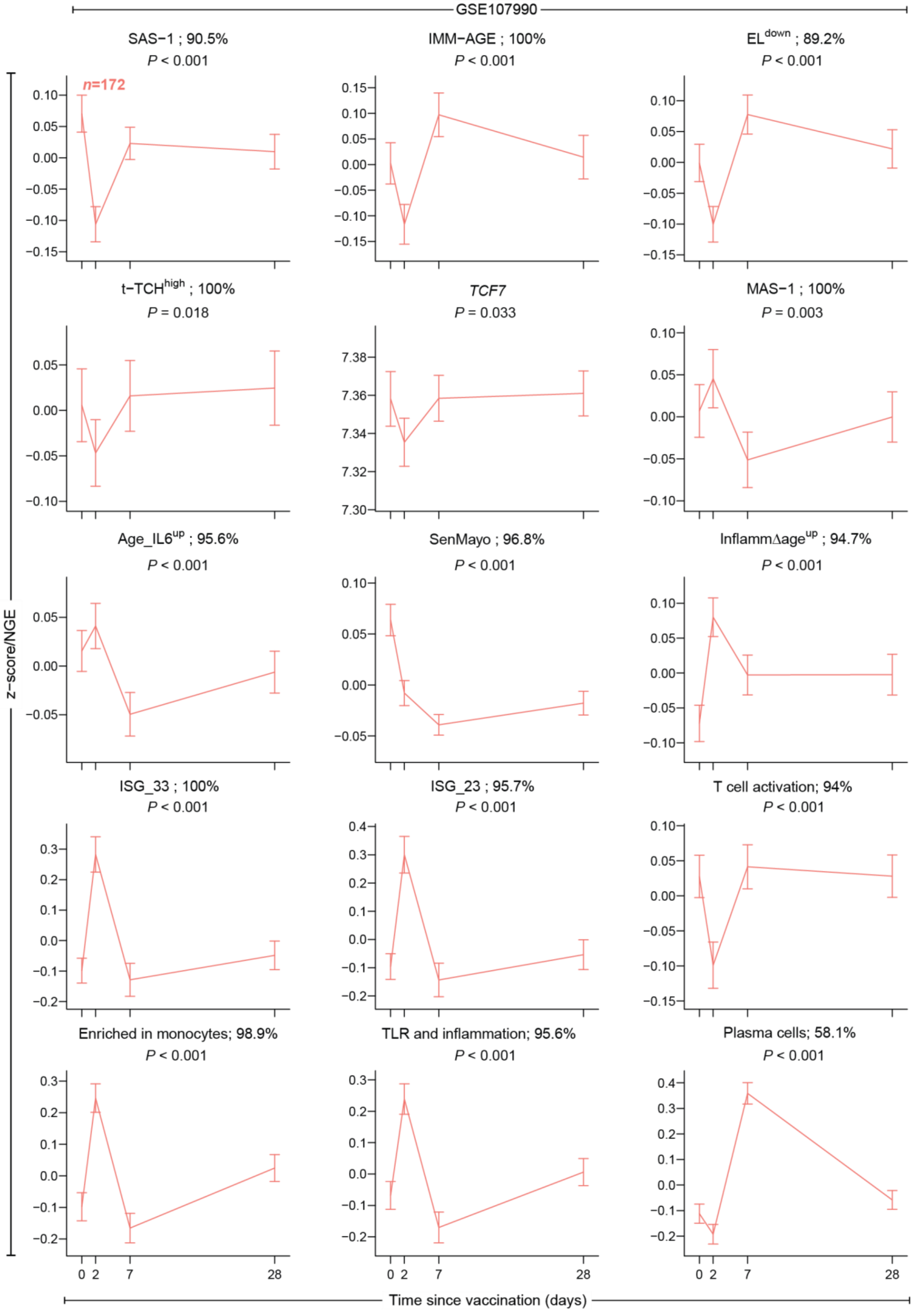

2945

Figure S17d: Influenza vaccination (continued): trajectories by SAS-1^{high}-MAS-1^{low} vs. SAS-1^{low}-MAS-1^{high}

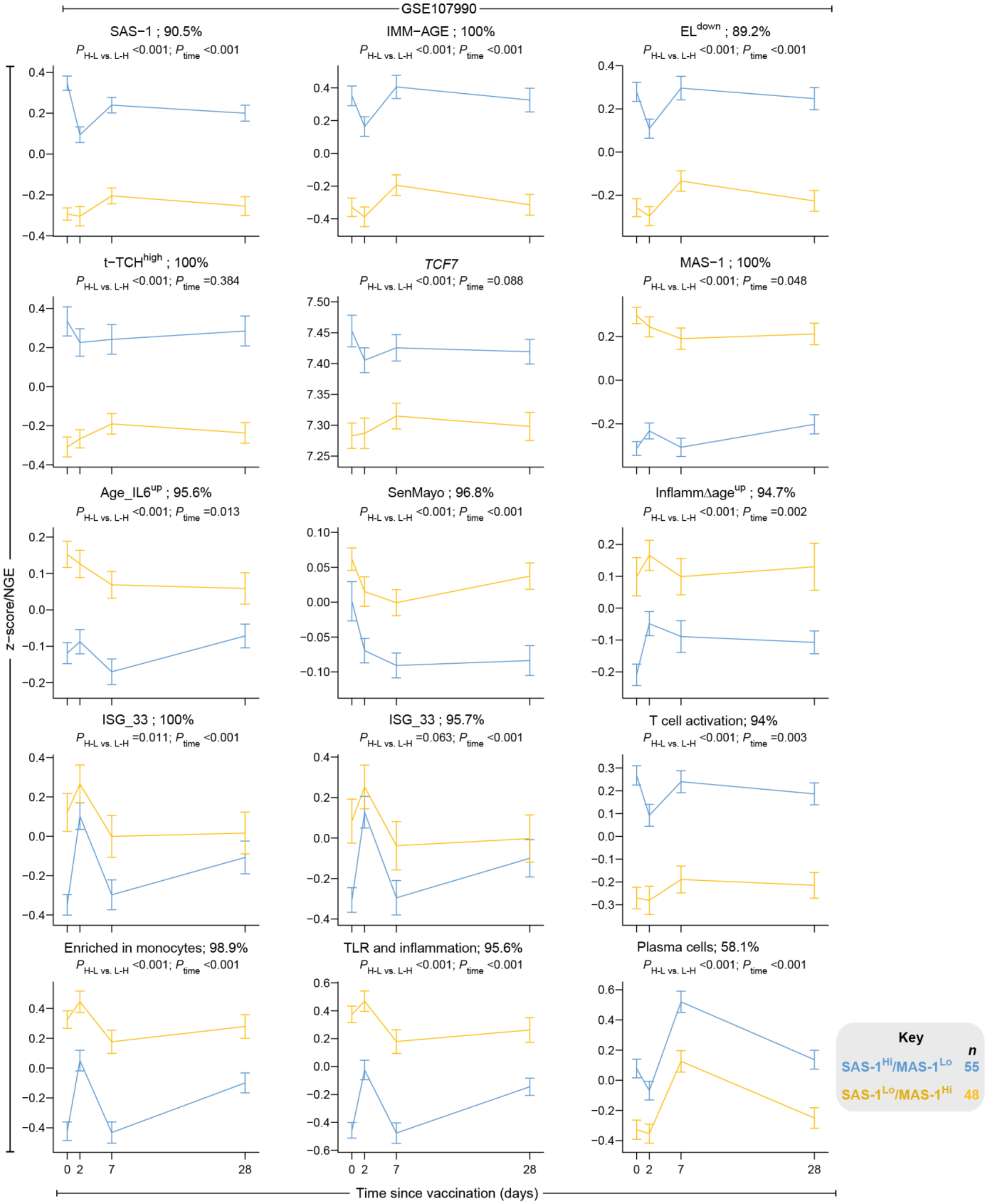

2946

Figure S17d: Influenza vaccination (continued)

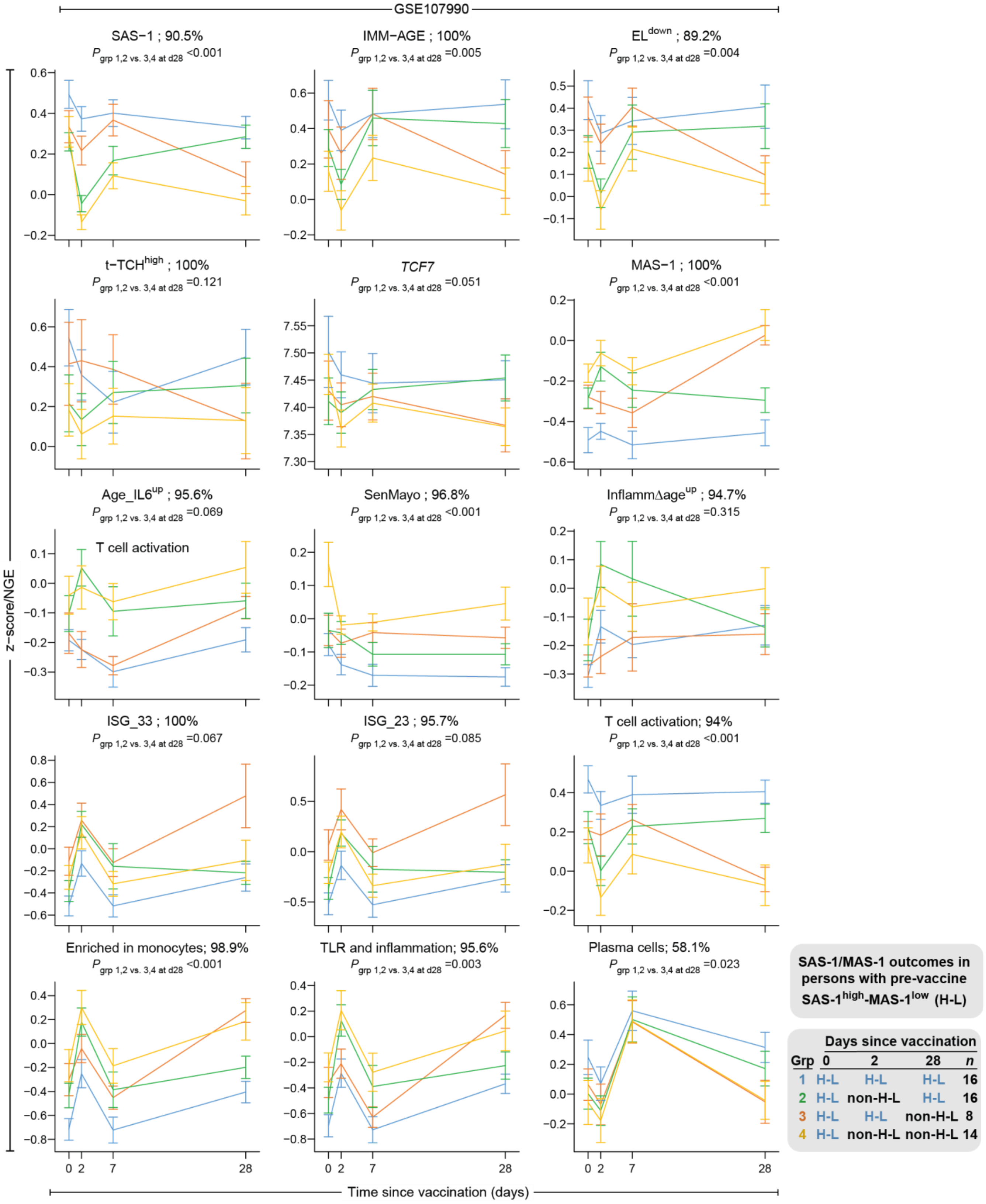

Figure S18. Association of immune resilience (IR) metrics, gene signatures tracking the pathogenic triad and vaccine responses, and *TCF7* expression levels in the Human Immunology Project Consortium (HIPC) trivalent influenza vaccine (TIV) cohort during 2010/11 flu season (GSE74813). (a) Stacked barplots depict the distribution of SAS-1/MAS-1 profiles and dot-and-line plots depict median (IQR) of *TCF7* (NGE). *Left*, SAS-1/MAS-1 profiles in the overall cohort at the indicated time points from the day of receiving vaccine (day 0). *Middle*, distribution of SAS-1/MAS-1 profiles after vaccination in persons whose profiles at baseline (pre-vaccination, day 0) were (*left to right*): SAS-1^{high}-MAS-1^{low} (H-L; optimal IR status); SAS-1^{high}-MAS-1^{high} (H-H); SAS-1^{low}-MAS-1^{low} (L-L); and SAS-1^{low}-MAS-1^{high} (L-H; extreme IR-degrader status). *Right*, SAS-1/MAS-1 profiles in persons with SAS-1^{high}-MAS-1^{low} at baseline (pre-vaccination) who were categorized into four IR groups based on their SAS-1/MAS-1 profiles at days 0, 1 and 14 post-vaccination: IR-preservers (H-L, H-L, H-L), IR-reconstituters (H-L, non-H-L, H-L), late IR-degraders (H-L, H-L, non-H-L), and early IR-degraders (H-L, non-H-L, non-H-L). N, non-SAS-1^{high}-MAS-1^{low}. **(b, c, d)** Line plots (mean ± SEM) of indicated gene signatures (z-scores) and *TCF7* levels (NGE) at the indicated timepoints **(b)** in the overall cohort, and by **(c)** optimal IR (SAS-1^{high}-MAS-1^{low}) vs. extreme IR-degrader (SAS-1^{low}-MAS-1^{high}) status. **(d)** Dour IR groups in the key shown at the bottom right. In panels to b-d, gene signatures tracking interferon-stimulated genes (ISG_33, ISG_23) and four gene modules associated with vaccine responses (Supplementary section 4.8) are shown. NGE, normalized gene expression. Details of the signatures are in Figure 1d (main) and Table S1. Higher levels of the IMM-AGE signature were computed to signify an association with fewer senescent T-cells (less immune aging and lower mortality; a {+}-salutogenesis readout), as detailed in Section 4.2. Statistical details are in Section 6.2.18.

Figure S18a: Influenza vaccination: SAS-1/MAS-1 distributions & *TCF7* levels

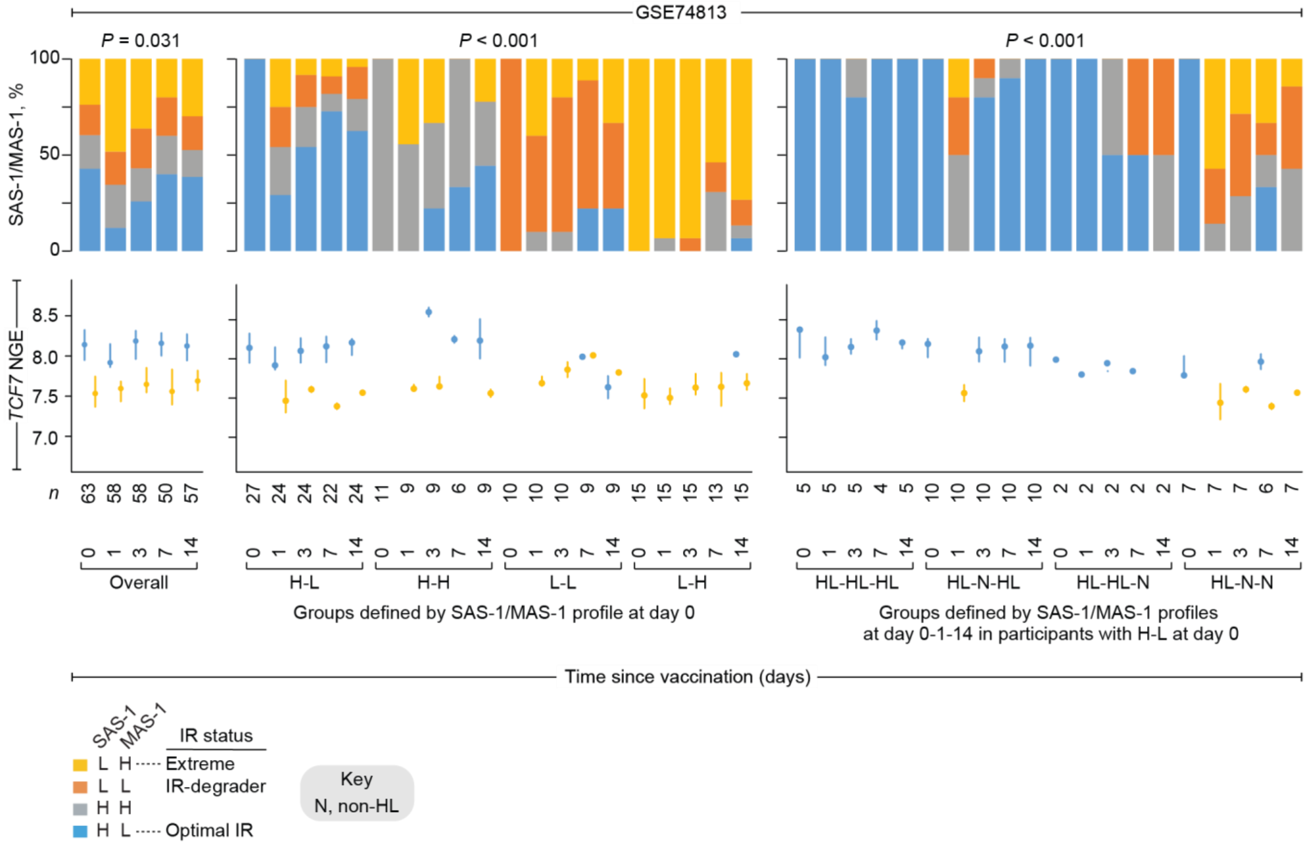

Figure S18b: Influenza vaccination (continued): overall trajectories

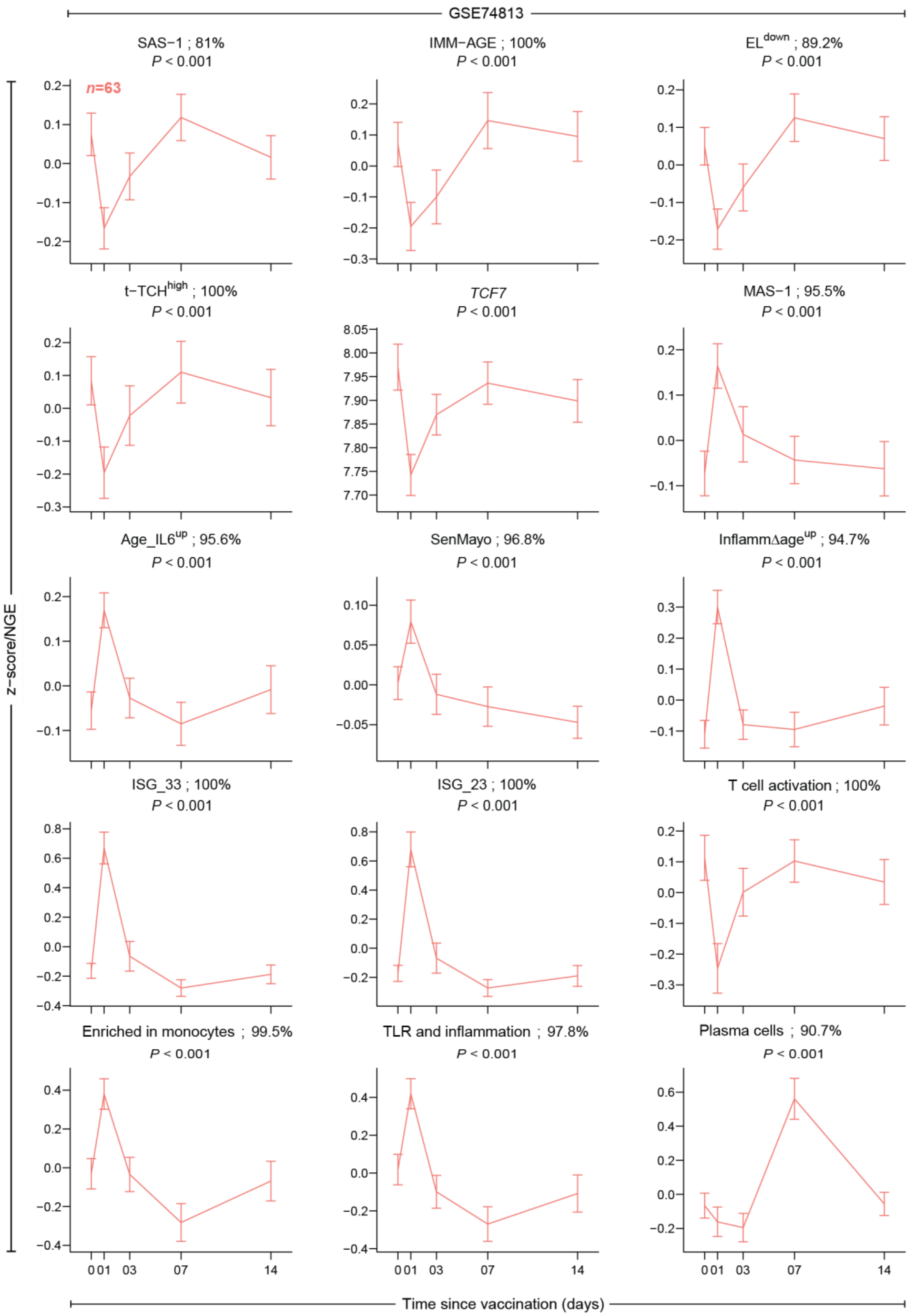

2974

Figure S18c: Influenza vaccination (continued): trajectories by SAS-1^{high}-MAS-1^{low} vs. SAS-1^{low}-MAS-1^{high})

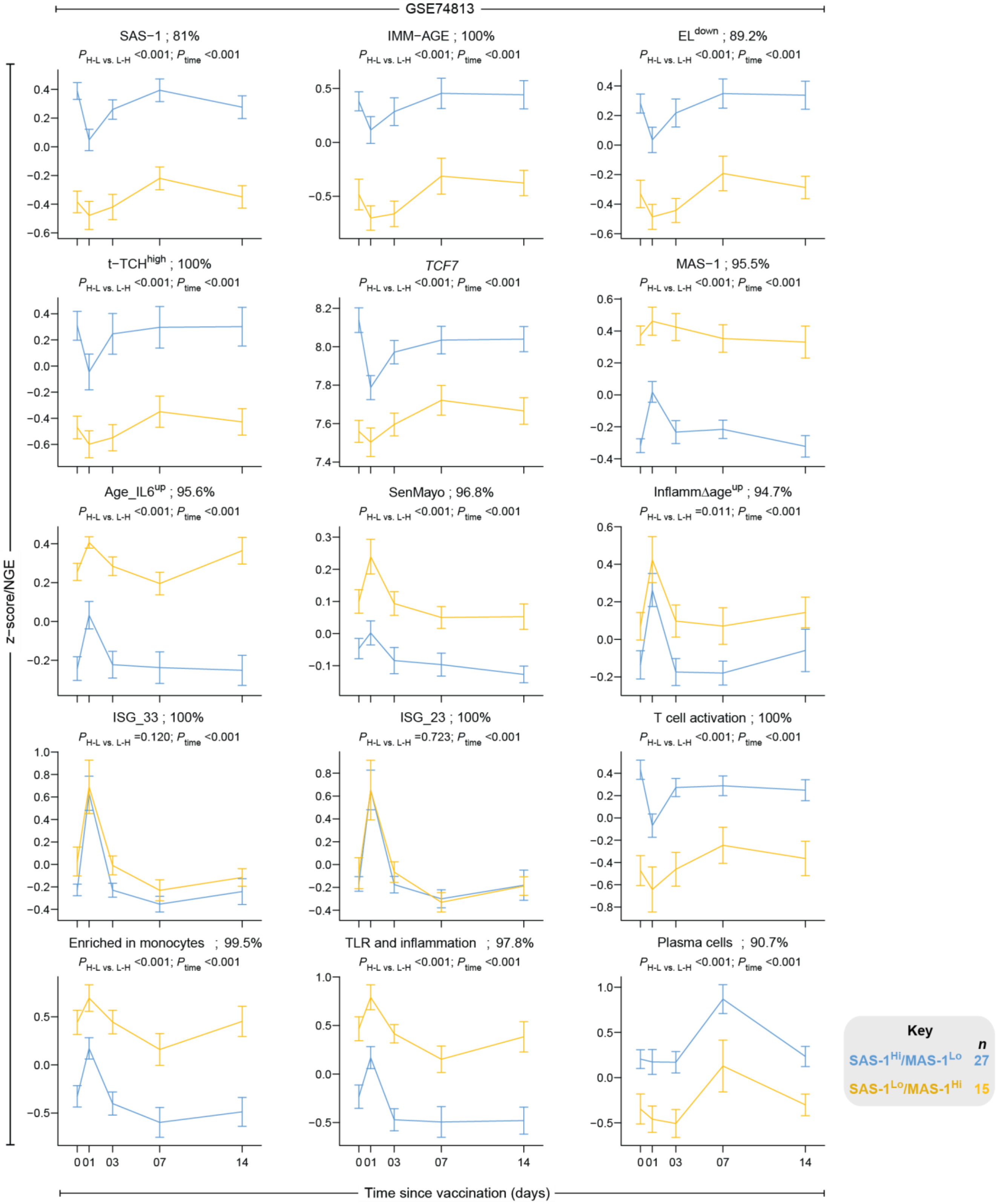

Figure S18d: Influenza vaccination (continued)

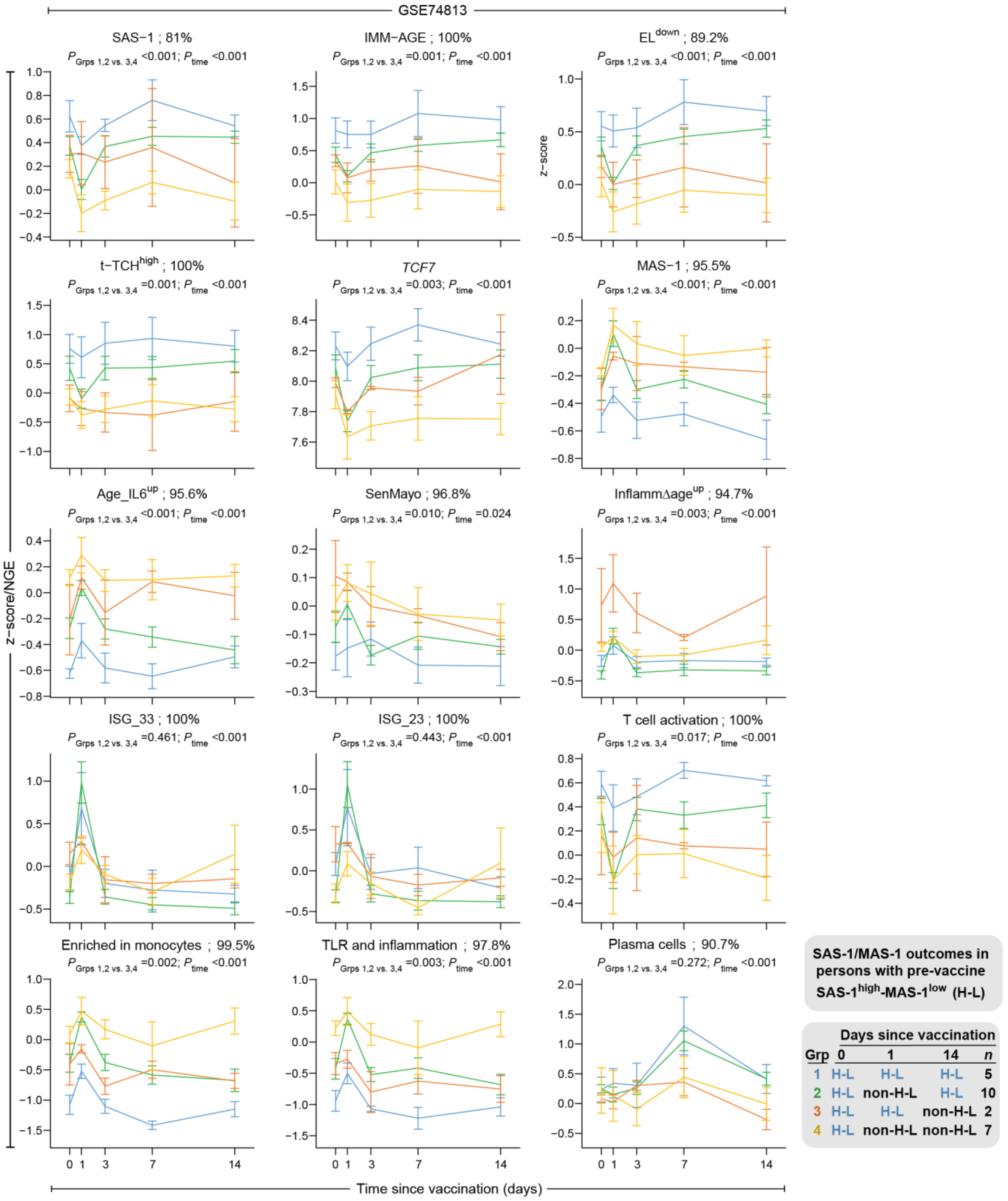

Figure S19. Association of immune resilience (IR) metrics, gene signatures tracking the pathogenic triad and vaccine responses, and *TCF7* expression levels in the trivalent influenza vaccination (TIV) female cohort (GSE48023). (a) Stacked barplots depict the distribution of SAS-1/MAS-1 profiles and dot-and-line plots depict median (IQR) of *TCF7* (NGE). *Left*, SAS-1/MAS-1 profiles in the overall cohort at the indicated time points from the day of receiving vaccine (day 0). *Middle*, distribution of SAS-1/MAS-1 profiles after vaccination in persons whose profiles at baseline (pre-vaccination, day 0) were (*left to right*): SAS-1^{high}-MAS-1^{low} (H-L; optimal IR status); SAS-1^{high}-MAS-1^{high} (H-H); SAS-1^{low}-MAS-1^{low} (L-L); and SAS-1^{low}-MAS-1^{high} (L-H; extreme IR-degrader status). *Right*, SAS-1/MAS-1 profiles in persons with SAS-1^{high}-MAS-1^{low} at baseline (pre-vaccination) who were categorized into four IR groups based on their SAS-1/MAS-1 profiles at days 0, 1 and 14 post-vaccination: IR-preservers (H-L, H-L, H-L), IR-reconstituters (H-L, non-H-L, H-L), late IR-degraders (H-L, H-L, non-H-L), and early IR-degraders (H-L, non-H-L, non-H-L). N, non-SAS-1^{high}-MAS-1^{low}. **(b, c, d)** Line plots (mean ± SEM) of indicated gene signatures (z-scores) and *TCF7* levels (NGE) at the indicated timepoints **(b)** in the overall cohort and by **(c)** optimal IR (SAS-1^{high}-MAS-1^{low}) vs. extreme IR-degrader (SAS-1^{low}-MAS-1^{high}) status, and **(d)** four IR groups in the key shown at the bottom right. In panels to b-d, gene signatures tracking interferon-stimulated genes (ISG_33, ISG_23) and four gene modules associated with vaccine responses (Supplementary section 4.8) are shown. NGE, normalized gene expression. Details of the signatures are in Figure 1d (main) and Table S1. Higher levels of the IMM-AGE signature were computed to signify an association with fewer senescent T-cells (less immune aging and lower mortality; a {+}-salutogenesis readout), as detailed in Section 4.2. Statistical details are in Section 6.2.19.

Figure S19a: Influenza vaccination: SAS-1/MAS-1 distributions & *TCF7* levels

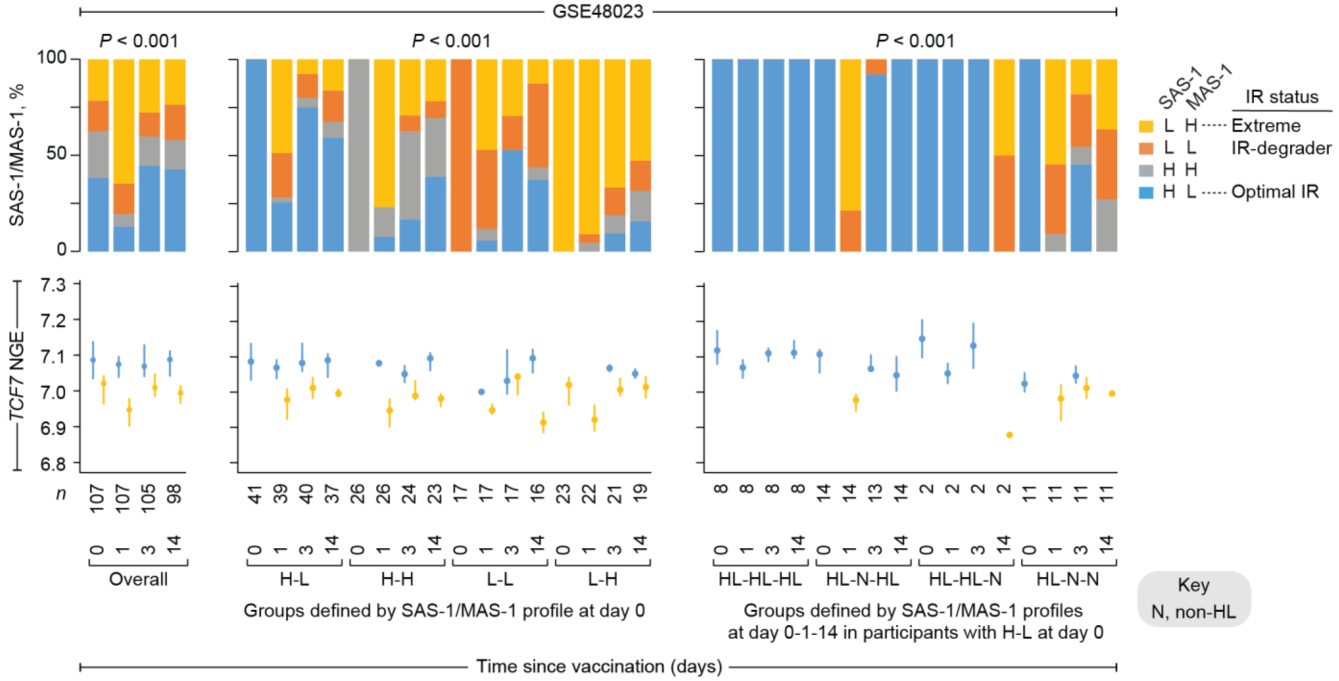

Figure S19b: Influenza vaccination (continued): overall trajectories

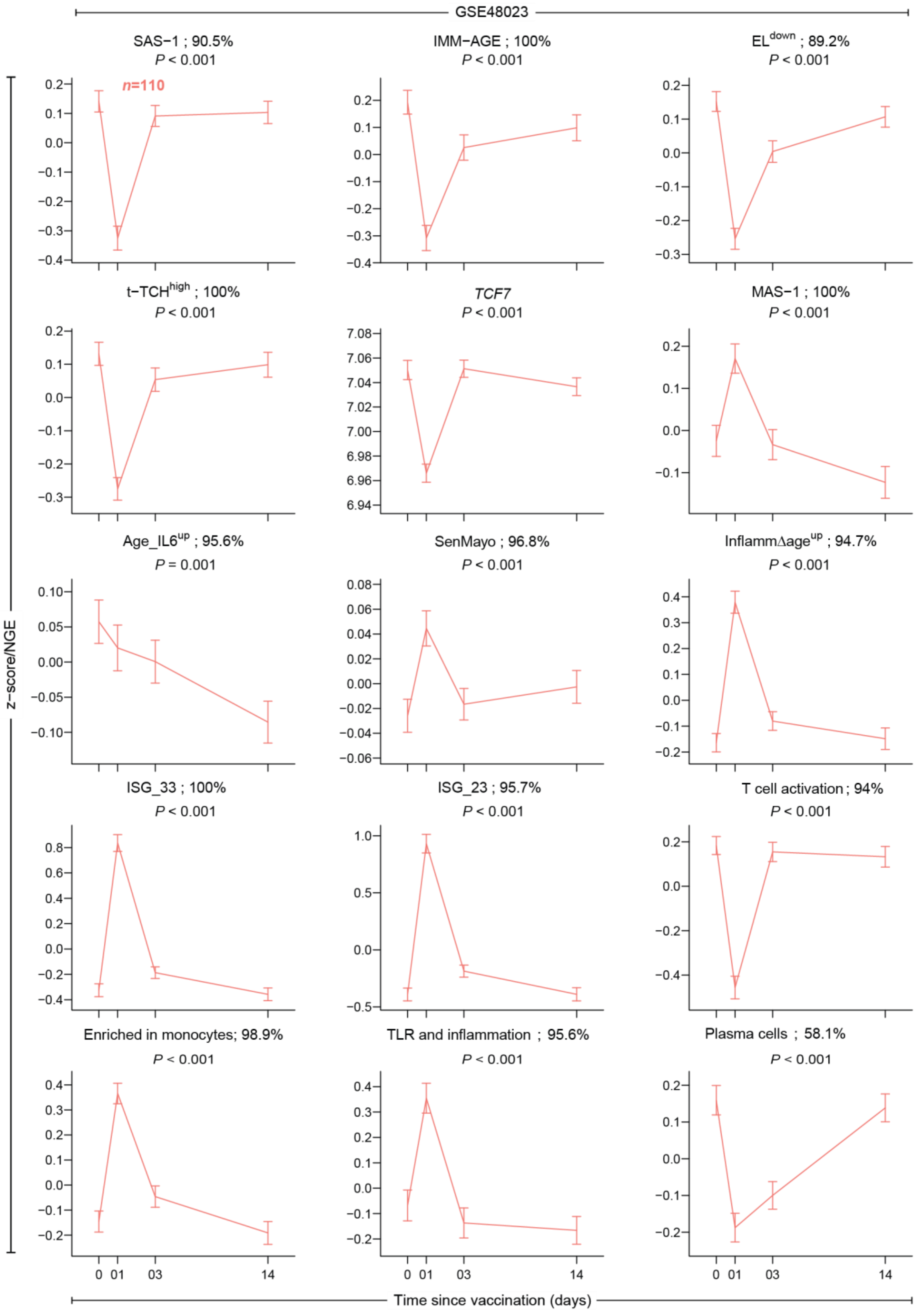

3002

Figure S19c: Influenza vaccination (continued): trajectories by SAS-1^{high}-MAS-1^{low} vs. SAS-1^{low}-MAS-1^{high}

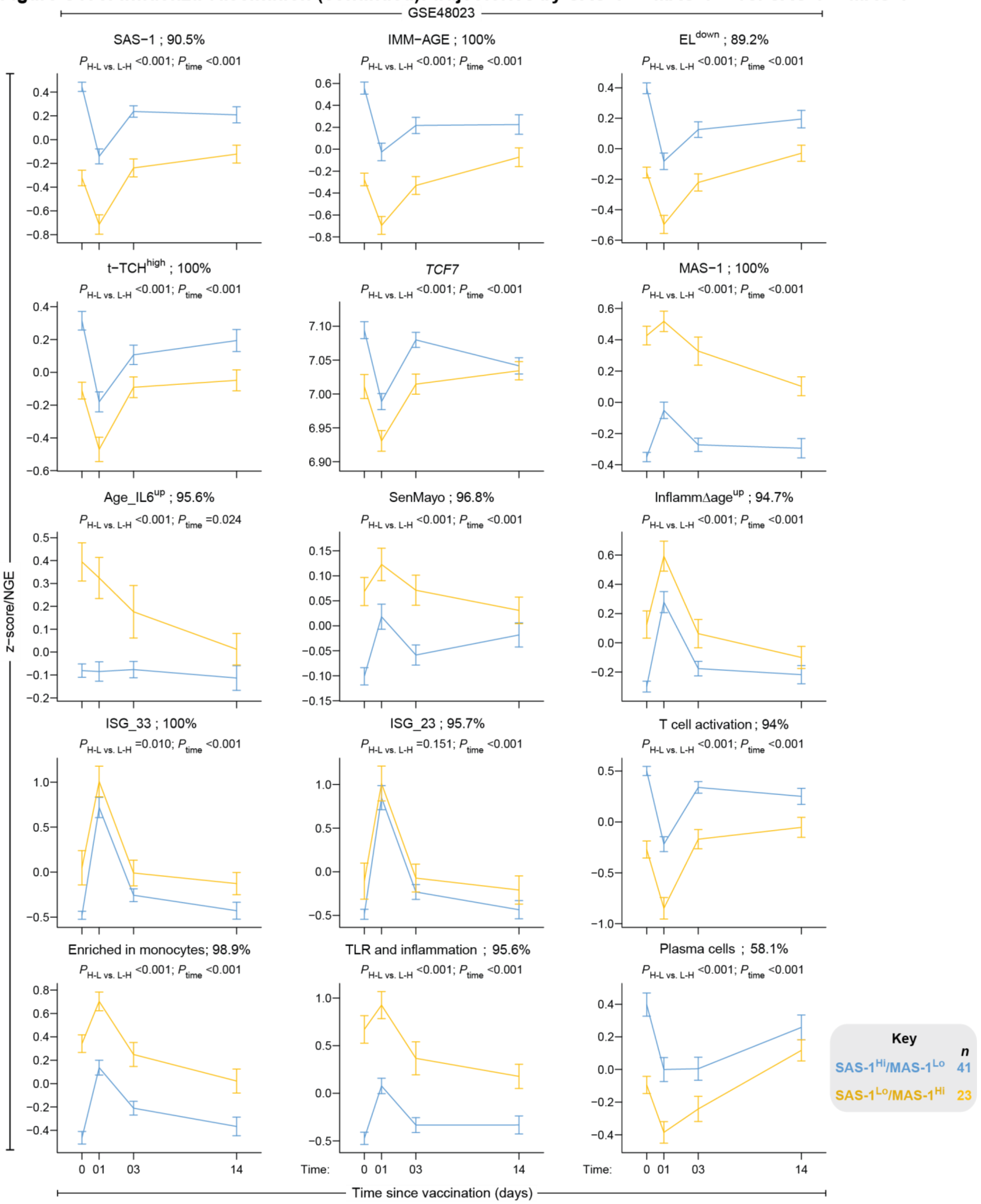

3003

Figure S19d: Influenza vaccination (continued)

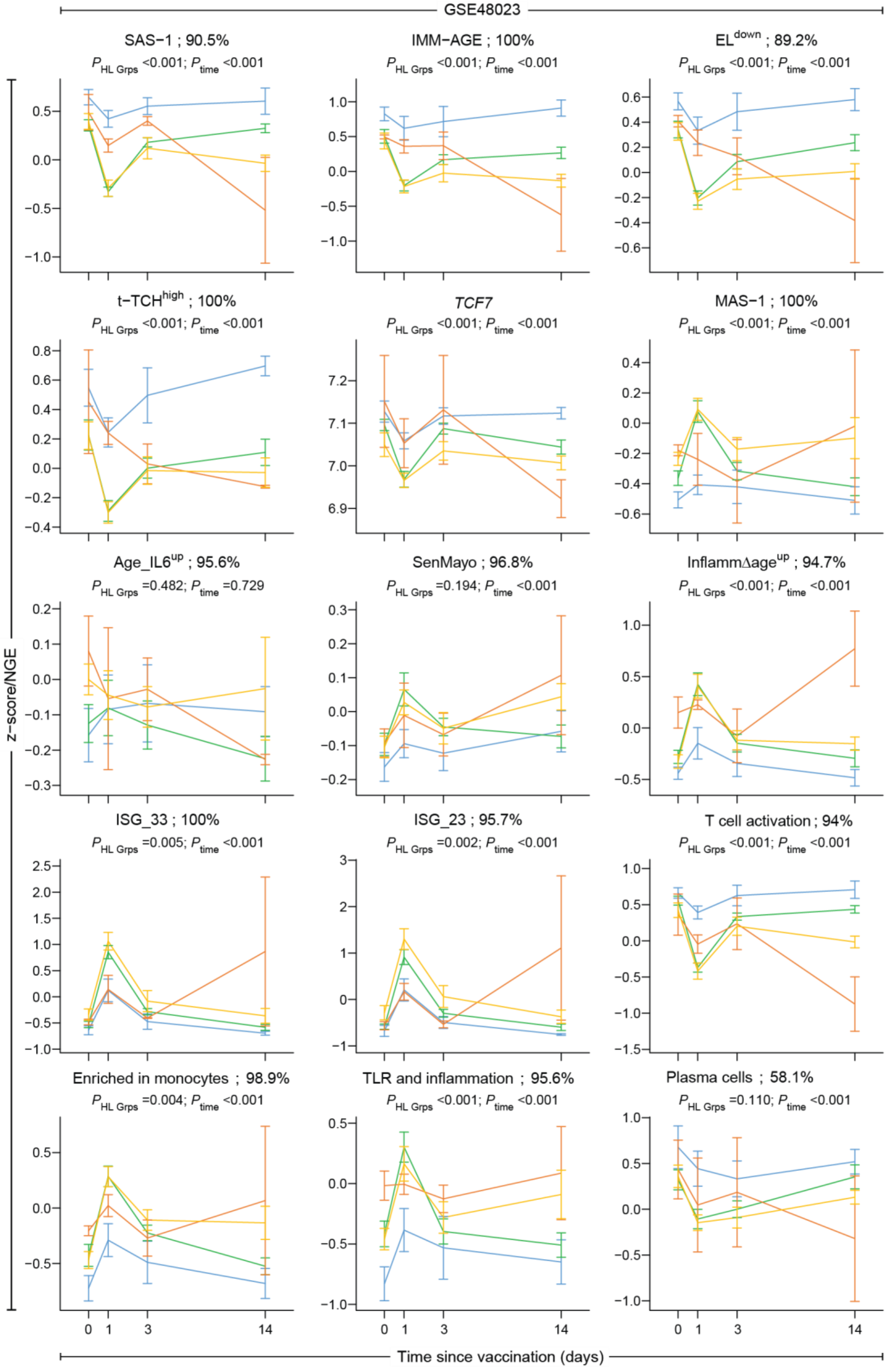

Grp	0	1	14	n
1	H-L	H-L	H-L	8
2	H-L	non-H-L	H-L	14
3	H-L	H-L	non-H-L	2
4	H-L	non-H-L	non-H-L	11

3004

3005

Figure S20. Association of immune resilience (IR) metrics, gene signatures tracking the pathogenic triad and vaccine responses, and *TCF7* expression levels in the trivalent influenza vaccination (TIV) male cohort (GSE48018). (a) Stacked barplots depict the distribution of SAS-1/MAS-1 profiles and dot-and-line plots depict median (IQR) of *TCF7* (NGE). *Left*, SAS-1/MAS-1 profiles in the overall cohort at the indicated time points from the day of receiving vaccine (day 0). *Middle*, distribution of SAS-1/MAS-1 profiles after vaccination in persons whose profiles at baseline (pre-vaccination, day 0) were (*left to right*): SAS-1^{high}-MAS-1^{low} (H-L; optimal IR status); SAS-1^{high}-MAS-1^{high} (H-H); SAS-1^{low}-MAS-1^{low} (L-L); and SAS-1^{low}-MAS-1^{high} (L-H; extreme-IR degrader status). *Right*, SAS-1/MAS-1 profiles in persons with SAS-1^{high}-MAS-1^{low} at baseline (pre-vaccination) who were categorized into four IR groups based on their SAS-1/MAS-1 profiles at days 0, 1, and 14 post-vaccination: IR-preservers (H-L, H-L, H-L), IR-reconstituters (H-L, non-H-L, H-L), late IR-degraders (H-L, H-L, non-H-L), and early IR-degraders (H-L, non-H-L, non-H-L). N, non-SAS-1^{high}-MAS-1^{low}. **(b, c, d)** Line plots (mean ± SEM) of indicated gene signatures (z-scores) and *TCF7* levels (NGE) at the indicated timepoints **(b)** in the overall cohort and by **(c)** optimal IR (SAS-1^{high}-MAS-1^{low}) vs. extreme IR-degrader (SAS-1^{low}-MAS-1^{high}) status, and **(d)** four IR groups in the key shown at the bottom right. In panels to b-d, gene signatures tracking interferon-stimulated genes (ISG_33, ISG_23) and four gene modules associated with vaccine responses (Supplementary section 4.8) are shown. NGE, normalized gene expression. Details of the signatures are in Figure 1d (main) and Table S1. Higher levels of the IMM-AGE signature were computed to signify an association with fewer senescent T-cells (less immune aging and lower mortality; a {+}-salutogenesis readout), as detailed in Section 4.2. Statistical details are in Section 6.2.20.

Figure S20a: Influenza vaccination: SAS-1/MAS-1 distributions & *TCF7* levels

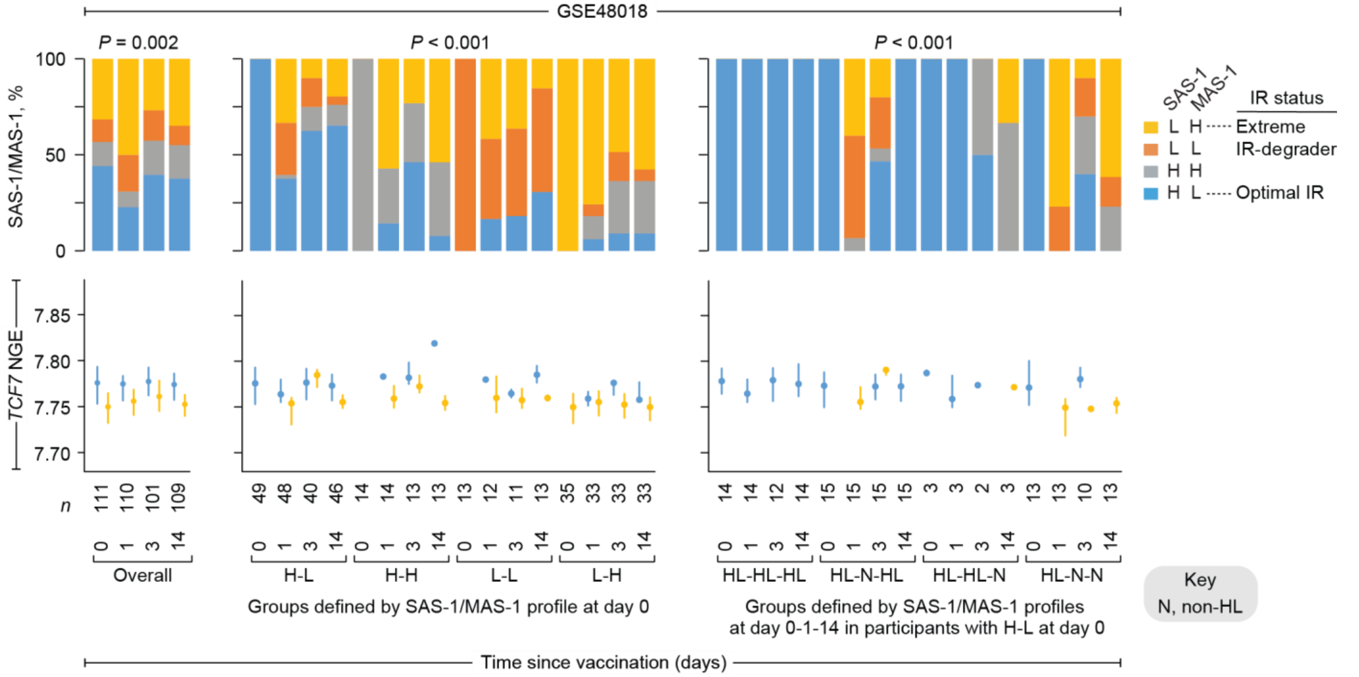

Figure S20b: Influenza vaccination (continued): overall trajectories

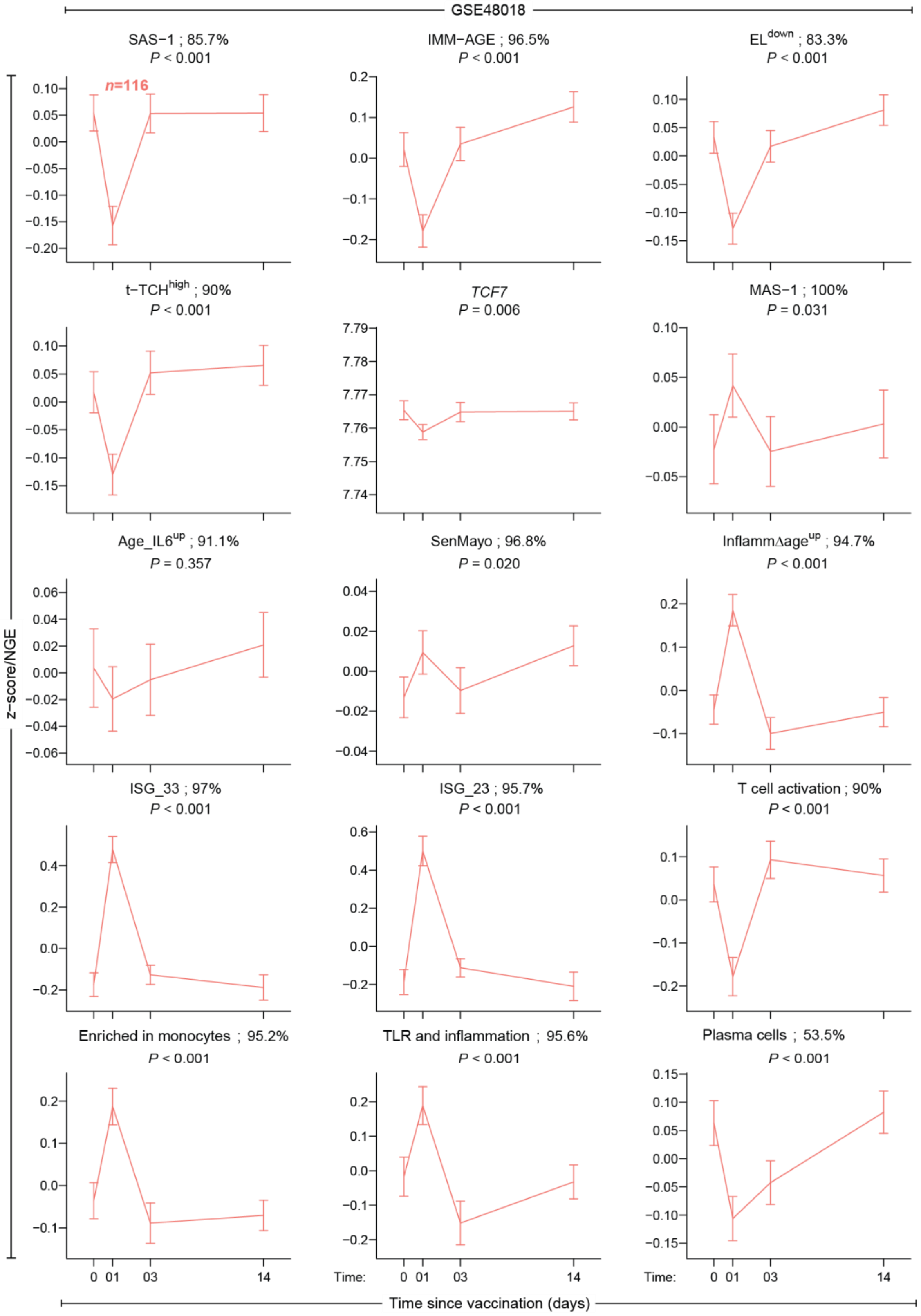

3030

Figure S20c: Influenza vaccination (continued): trajectories by SAS-1^{high}-MAS-1^{low} vs. SAS-1^{low}-MAS-1^{high}

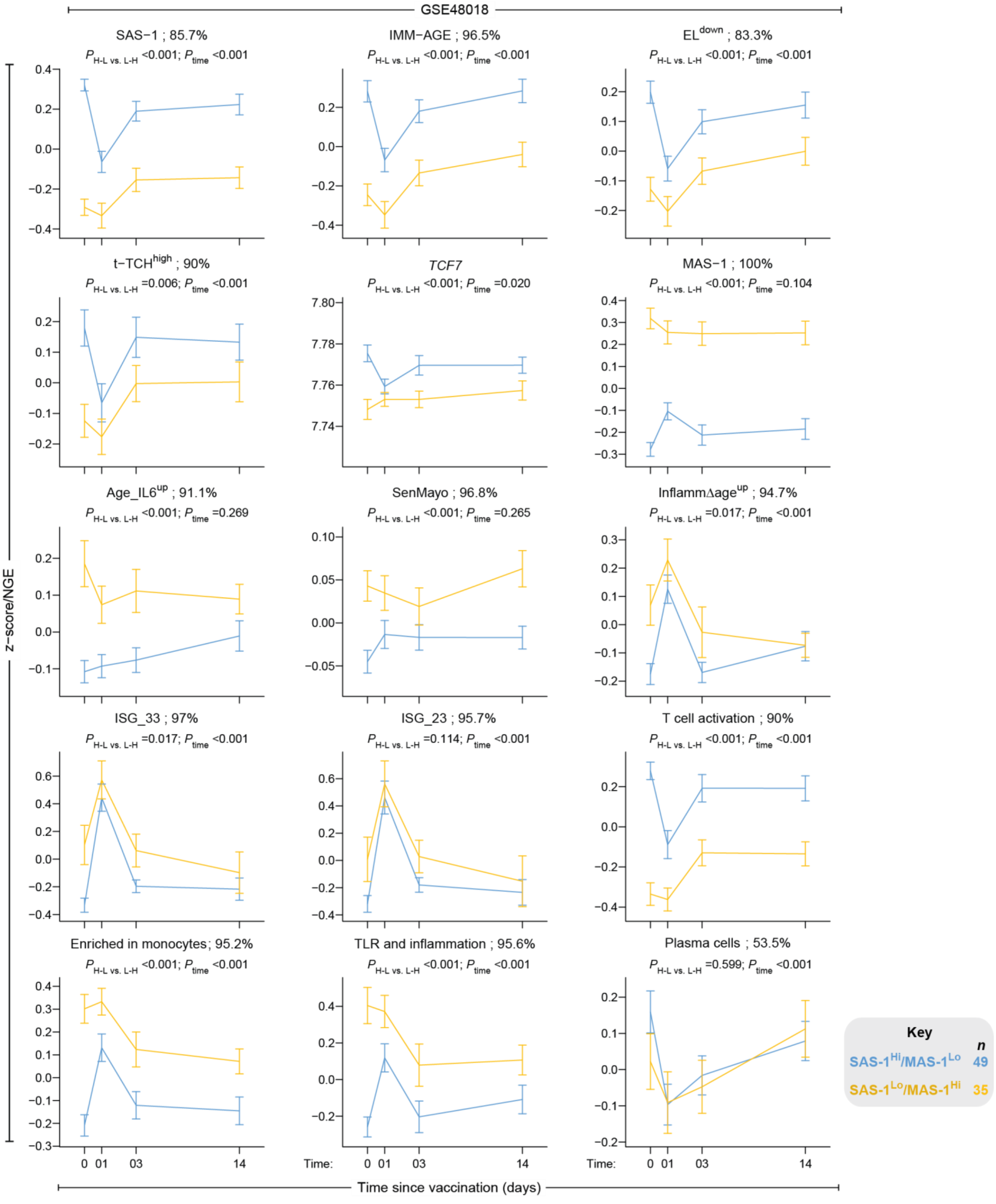

3031

Figure S20d: Influenza vaccination (continued)

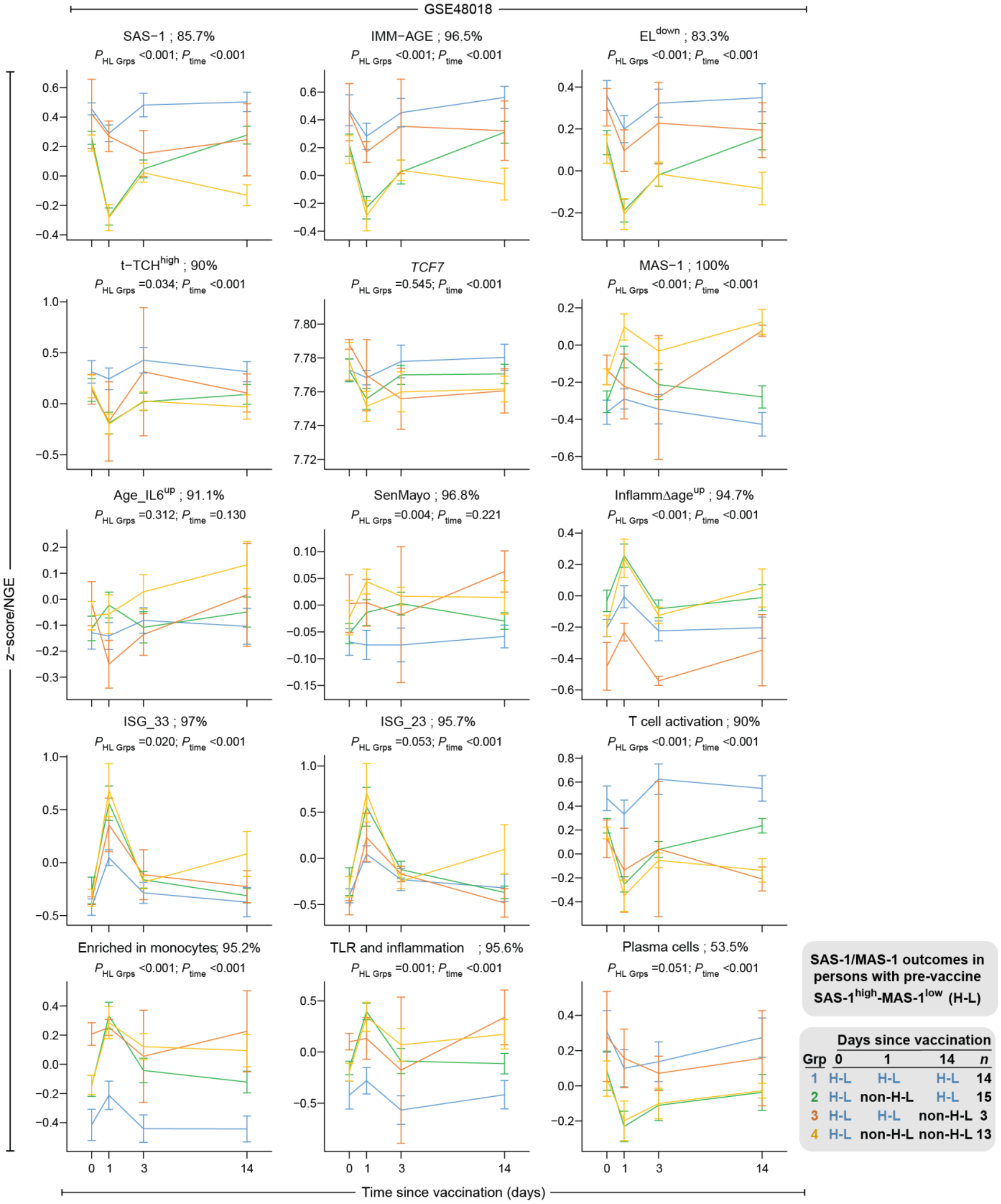

Figure S21. Association of immune resilience (IR) metrics, gene signatures tracking the pathogenic triad and vaccine responses, and *TCF7* expression levels in the Stanford SARS-CoV-2 vaccine cohort (GSE169159). (a) Schema depict the days since receiving the first dose of a SARS-CoV-2 RNA vaccine (Pfizer). Among persons with a SAS-1^{high}-MAS-1^{low} profile before vaccination, the rate of conversion to an SAS-1^{low}-MAS-1^{high} profile after the receipt of the first (10%) and second (50%) doses of the vaccine are noted. **(b)** *Top*, SAS-1/MAS-1 profiles at the indicated timepoints in persons with pre-vaccination (day 0) SAS-1^{high}-MAS-1^{low} and SAS-1^{low}-MAS-1^{high}. *Bottom*, dot-and-line plots depict median (IQR) of *TCF7* (NGE). **(c)** Line plots (mean ± SEM) of the indicated gene signatures (z-scores) and *TCF7* levels (NGE) at the indicated timepoints in persons with optimal IR (SAS-1^{high}-MAS-1^{low}) vs. extreme IR-degrader (SAS-1^{low}-MAS-1^{high}) status before vaccination. (**d)** Line plots (mean ± SEM) of indicated gene modules that associate with vaccine responses (Supplementary section 4.8). NGE, normalized gene expression. Details of the signatures are in Figure 1d (main) and Table S1. Higher levels of the IMM-AGE signature were computed to signify an association with fewer senescent T-cells (less immune aging and lower mortality; a {+}-salutogenesis readout), as detailed in Section 4.2. Statistical details are in Section 6.2.21.

Figure S21: SARS-CoV-2 Vaccination

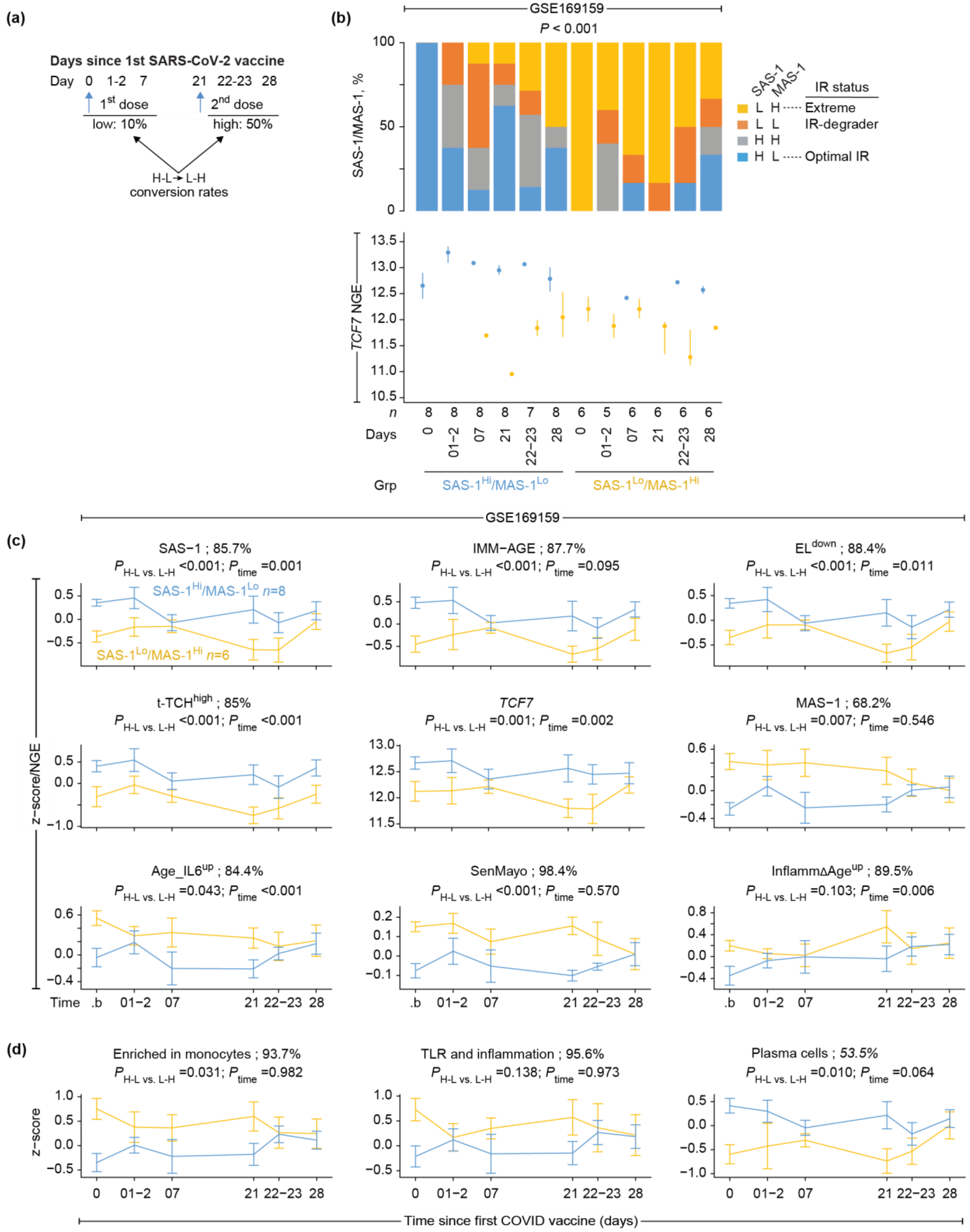

Figure S22. Association of immune resilience (IR) metrics, gene signatures tracking the pathogenic triad, and *TCF7* expression levels in the respiratory syncytial virus (RSV) cohort (GSE188427). (a) Line plots (mean ± SEM) of the indicated gene signatures (z-scores) and *TCF7* levels (NGE) at the indicated timepoints in the control vs. RSV groups. Controls were samples at only one timepoint. **(b)** *Left*, SAS-1/MAS-1 profiles in the overall cohort at the indicated timepoints from the day of diagnosis (day 1). Note: Day 1 is associated with a high prevalence of the SAS-1^{low}-MAS-1^{high} combination (extreme IR-degrader status) followed by reconstitution of the distribution of the SAS-1/MAS-1 profiles at day 30 post-diagnosis. *Right*, Distribution of SAS-1/MAS-1 profiles after diagnosis in the inpatient and outpatient groups at the indicated timepoints. NGE, normalized gene expression. Details of the signatures are in Figure 1d (main) and Table S1. Higher levels of the IMM-AGE signature were computed to signify an association with fewer senescent T-cells (less immune aging and lower mortality; a {+}-salutogenesis readout), as detailed in Section 4.2. Statistical details are in Section 6.2.22. Data in Figure S22b correspond to Figure 5a (main).

Figure S22a: RSV

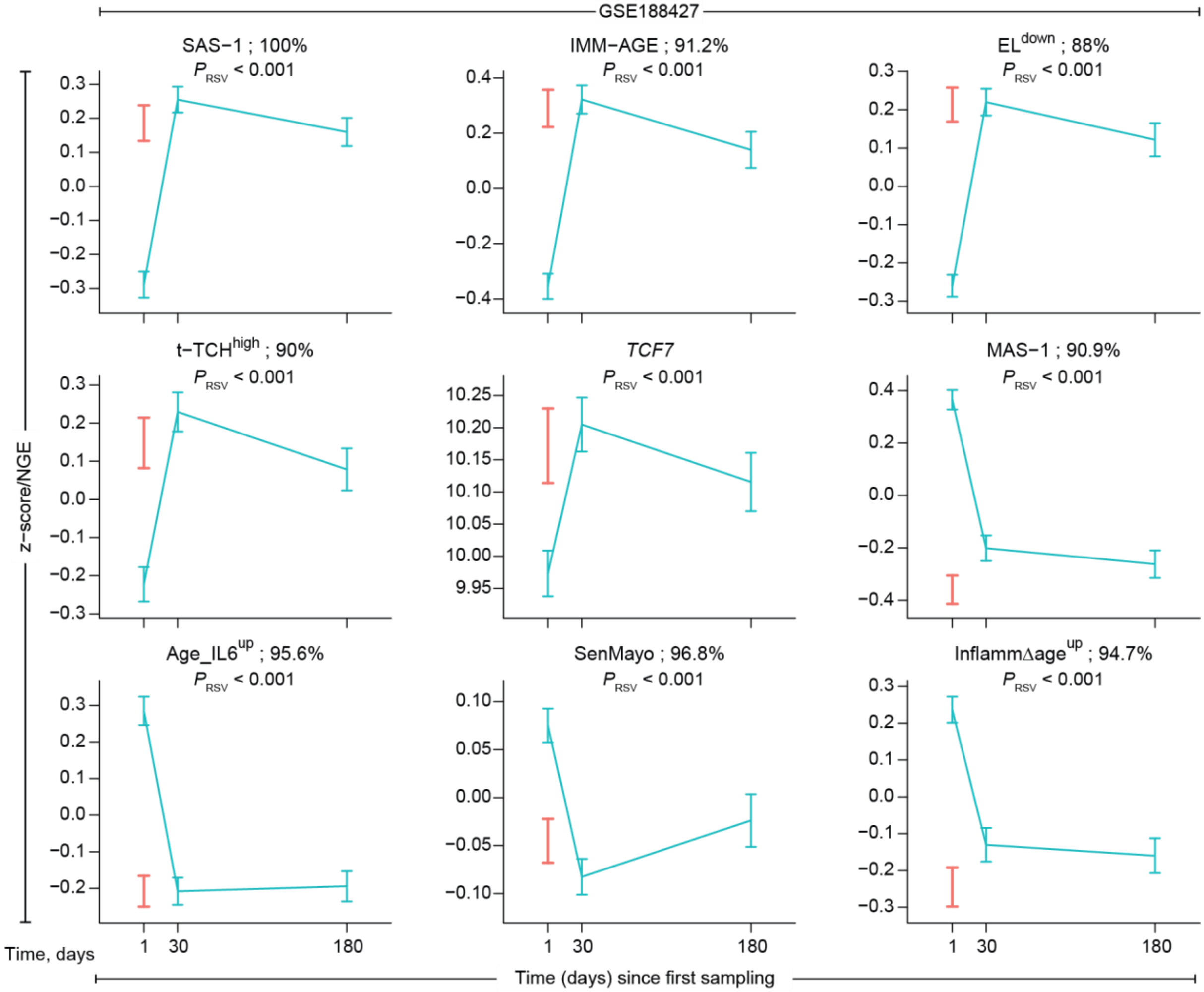

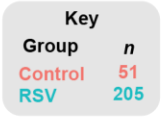

Figure S22b: RSV (corresponds to Case 4 main Figure 5a)

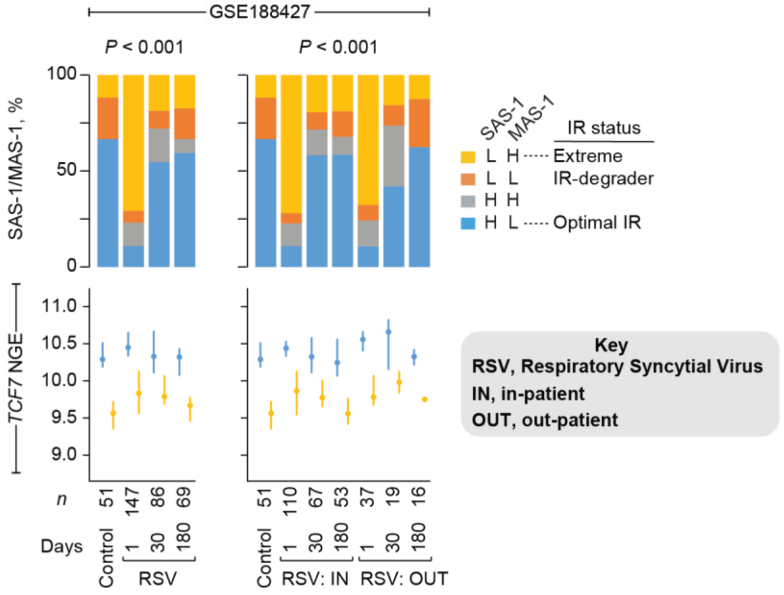

Figure S23. Association of immune resilience (IR) metrics, gene signatures tracking the pathogenic triad, and *TCF7* expression levels in the STEMI (ST-segment elevation myocardial infarction) cohort (GSE59867). Line plots (mean ± SEM) of the indicated gene signatures (z-scores) and *TCF7* levels (NGE) over the indicated timepoints in persons with optimal IR (SAS-1^{high}-MAS-1^{low}) *vs.* extreme IR-degrader (SAS-1^{low}-MAS-1^{high}) status. NGE, normalized gene expression. Details of the signatures are in Figure 1d (main) and Table S1. Higher levels of the IMM-AGE signature were computed to signify an association with fewer senescent T-cells (less immune aging and lower mortality; a {+}-salutogenesis readout), as detailed in Section 4.2. Statistical details are in Section 6.2.23. Data correspond to Figure 5c (main).

Figure S23: STEMI (corresponds to Case 5 main Figure 5c)

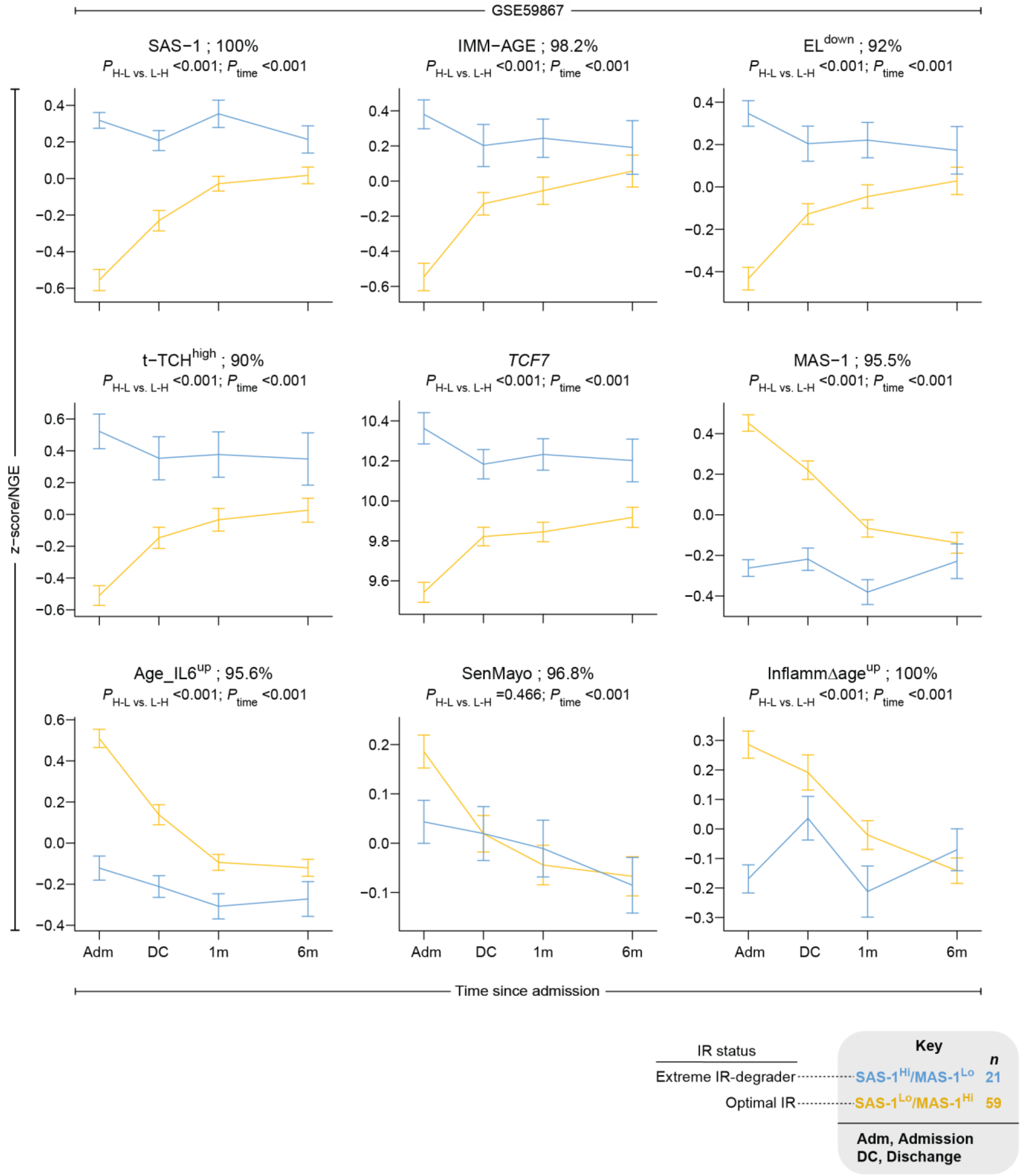

3081

3082

Figure S24. Association of immune resilience (IR) metrics, gene signatures tracking the pathogenic triad, and *TCF7* expression levels in the Vitality 90+ Study (GSE65218). Boxplots depict median (IQR) expression levels of the indicated gene signature (z-scores) and *TCF7* (NGE) by SAS-1/MAS-1 profiles (*left to right*): SAS-1^{low}-MAS-1^{high} (L-H; extreme IR-degrader status); SAS-1^{low}-MAS-1^{low} (L-L); SAS-1^{high}-MAS-1^{high} (H-H); SAS-1^{high}-MAS-1^{low} (H-L; optimal IR status). NGE, normalized gene expression. Details of the signatures are in Figure 1d (main) and Table S1. Higher levels of the IMM-AGE signature were computed to signify an association with fewer senescent T-cells (less immune aging and lower mortality; a {+}-salutogenesis readout), as detailed in Section 4.2. Statistical details are in Section 6.2.24. Data correspond to Figure 5e (main).

Figure S24: Vitality 90+ study (corresponds to Case 6 main Figure 5e)

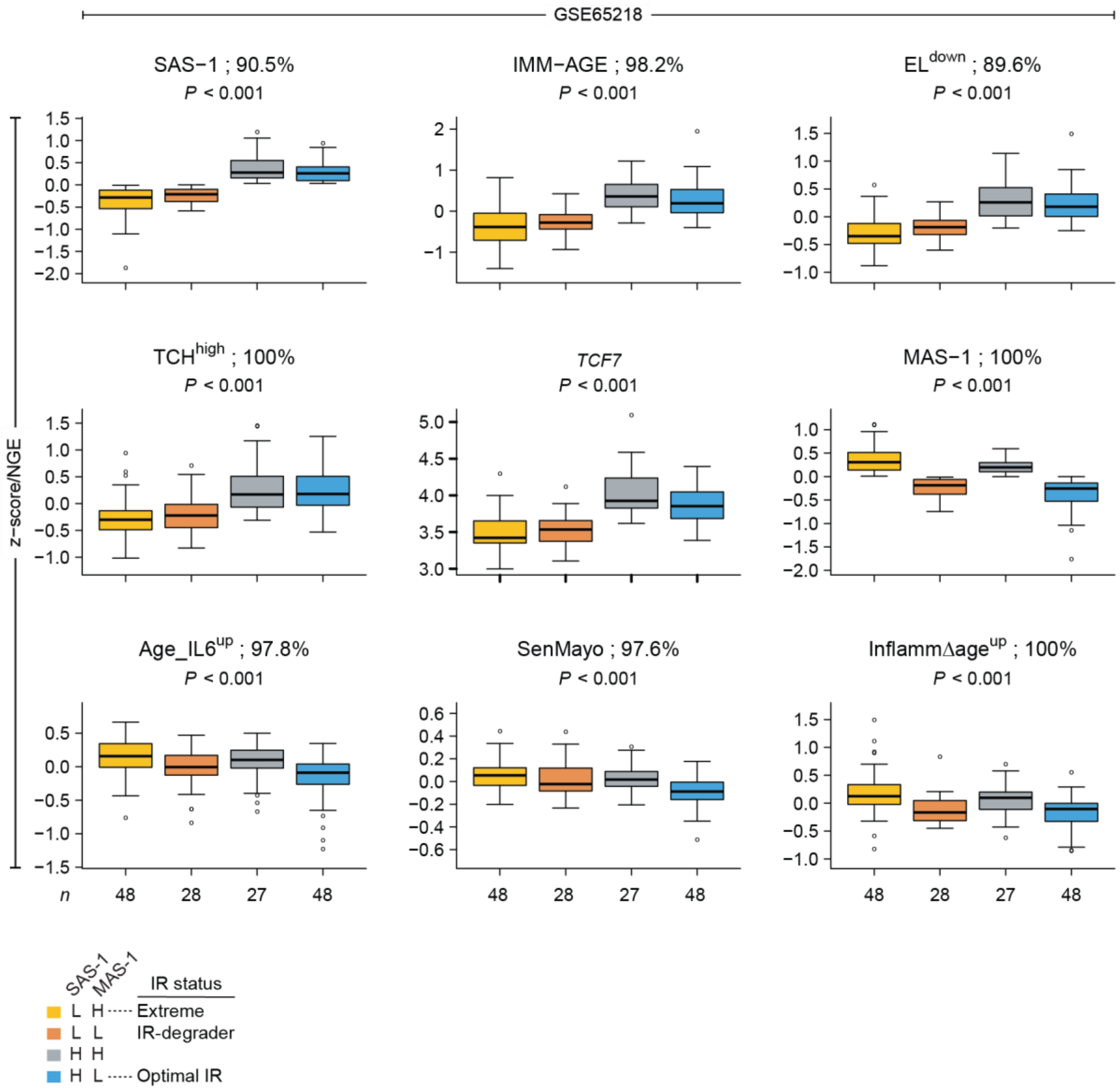

Figure S25. Time to cardiovascular disease (CVD) diagnosis in the Framingham Heart Study (FHS). Kaplan-Meier plots depict time to CVD diagnosis by quartiles of Age_IL6^{up} gene signature in the FHS. Analysis by Cox proportional hazards models adjusted for age, sex, smoking status, diabetes, hypertension, and total cholesterol with likelihood ratio test (LRT). Statistical details are in Section 6.2.25.

Figure S25

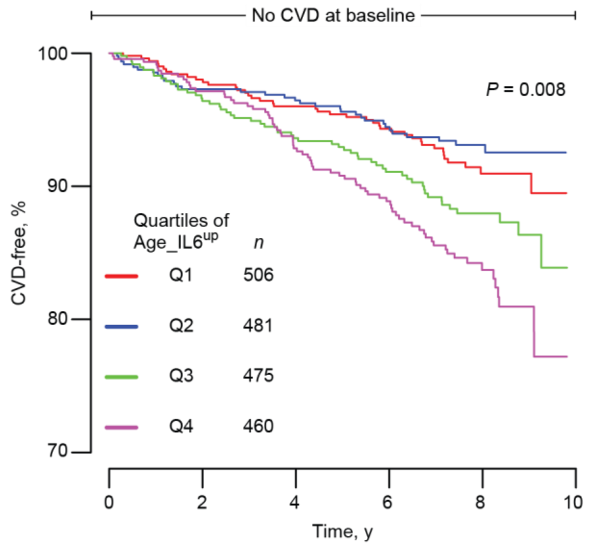

Figure S26. COVID-19 and non-COVID-19 cohorts: features and Immune Health Grades (IHG). **(a)** Study populations developed at the Audie Murphy VA Hospital in San Antonio, Texas (South Texas Veterans Health Care System). I: VA-CLC [**VA** (Veterans Affairs) **C**OVID-19 **L**ongitudinal **C**ohort]. II: SAIL [**S**an **A**ntonio **I**mmune Resilience (IR) **L**ongitudinal cohort]. Outcomes evaluated in the VA-CLC are noted. The subset of the SAIL who had not received a booster and in whom the association between IR status and vaccine responsiveness is noted. ARDS, acute respiratory distress syndrome; IC, immunocompetence; IF, inflammation; CRP, C-reactive protein; nAb, neutralizing antibodies; NK, natural killer. **(b)** Distribution of IHGs in COVID-19 and non-COVID-19 (controls) cohorts. Top, IHG derivation. Bottom, IHG prevalence during acute COVID-19 (ac-COVID-19), convalescent (conv.) COVID-19 and controls. Shaded values, IHG-IIc in non-ac-COVID-19 cohorts; blue boxes, IHG-I in non-ac-COVID-19 and HIV-seropositive cohorts; red boxes, IHG-IV subgrades overrepresented in persons with HIV risk factors and infection; purple boxes, IHGs overrepresented during ac-COVID-19. <u>Note:</u> The acute lymphopenia of ac-COVID-19 is concentrated in persons presenting with IHG-IIc (13.1%) and less so with IHG-IVc (5.2%). In contrast, the lymphopenia of HIV is restricted to IHG-IV grades. ART, HIV antiretroviral therapy; FSW, female sex worker; H, high; L, low; NA, not assigned; NHS, Natural HIV History Study; UCSD, University of San Diego; IR, immune resilience. Statistical details are in Section 6.2.26.

Figure S26

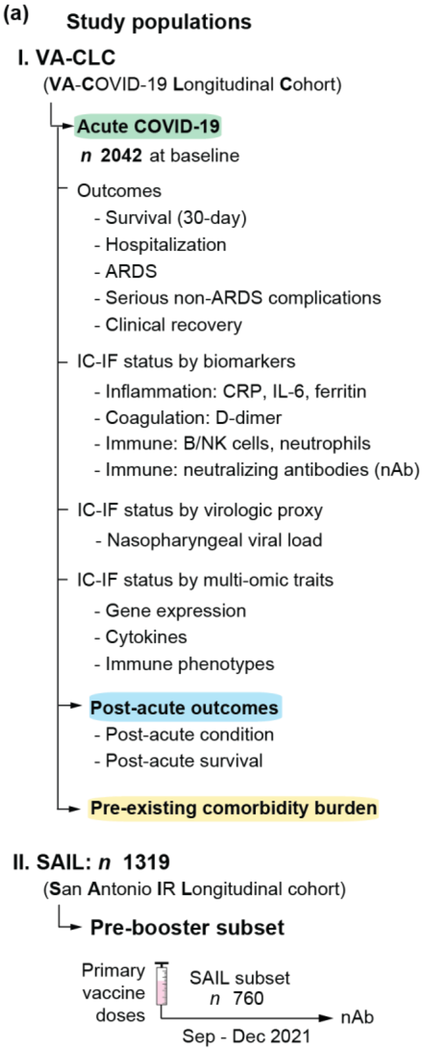

(b)

Immune Health Grade (IHG); Prevalence, %

Group	Population	*n*	age	I	IIa	IIb	IIc	III	IVa	IVb	IVc
	CD4⁺ (cells/mm³)			≥800	≥500 <800	>200 <500	≤200	≥800	≥500 <800	>200 <500	≤200
	CD4:CD8 ratio			≥ 1.0				< 1.0			
	CD8⁺ levels			L	L	L	L	H	H	H	H
	CD4⁺ levels			H	L	LL	LLL	H	L	LL	LLL
Study groups	SAIL	1319	53 (43-63)	56.0	28.4	7.1	0.1	1.8	3.7	2.7	0.3
Study groups	ac-COVID-19	2042	62 (48-72)	20.7	22.3	29.2	13.1	1.5	2.3	5.8	5.2
Study groups	ac-C19 conv.	733	59 (48-70)	45.8	32.6	9.0	0.5	3.1	4.8	4.0	0.1
Non-COVID-19 reference populations, HIV−	SardiNIA	3898	49 (36-62)	72.9	20.3	2.6	0.1	2.0	1.6	0.5	0.1
Non-COVID-19 reference populations, HIV−	UCSD	759	39 (30-48)	64.8	25.7	3.6	0.1	2.5	2.6	0.7	0.0
Non-COVID-19 reference populations, HIV−	Sex workers	449	31 (27-37)	64.8	18.7	1.1	0.0	9.1	4.2	1.6	0.4
Non-COVID-19 reference populations, HIV+	Acute (<10 mo)	592	33 (26-40)	4.1	7.4	5.4	0.5	6.4	31.4	42.6	2.2
Non-COVID-19 reference populations, HIV+, NHS	Cohort entry	1110	29 (24-35)	3.6	6.1	2.4	0.0	6.4	26.0	49.3	6.1
Non-COVID-19 reference populations, HIV+, NHS	Pre-ART	1110	31 (26-37)	0.5	2.7	1.7	0.0	2.7	14.9	67.4	10.2
Non-COVID-19 reference populations, HIV+, NHS	During ART	1110	34 (29-40)	28.8	14.1	1.2	0.0	23.2	24.5	8.1	0.2
				optimal IR			extreme IR degrader				extreme IR degrader

Figure S27. Immune health grade (IHG) distributions in controls and during the acute and post-acute phases of COVID-19. (a) Distribution of IHG by sex, age strata, and age strata within sex in the SAIL cohort without acute COVID-19 (ac-COVID-19) (*top left*), overall VA-CLC ac-COVID-19 cohort (*bottom left*) and paired longitudinal samples from the VA-CLC cohort in the acute (*bottom right*) vs. post-acute stages (*top right*). Dashed lines indicate the highest and lowest percentages of IHG-I in the young (Y) females (F) and old (O) males (M), respectively. *P*, χ^2, and Fisher's exact test. Note: Sex dimorphism and IHG distributions are reconstituted in post-acute samples to mirror that of controls. VA-CLC, VA (Veterans Affairs) COVID-19 Longitudinal Cohort; SAIL, San Antonio Immune Resilience (IR) Longitudinal cohort. **(b)** Distribution of baseline IHGs during post-acute COVID-19 according to the IHGs at presentation with ac-COVID-19. *P*, Fisher's exact test. **(c)** Distribution of IHGs at baseline and convalescence by pre-COVID-19 IHG status in HIV-seropositive persons in the VA-CLC. *P*, Fisher's exact test. Note: The IHG reconstituted during convalescence is dependent on the baseline and/or pre-COVID-19 IHG status. Statistical details are in Section 6.2.27.

Figure S27

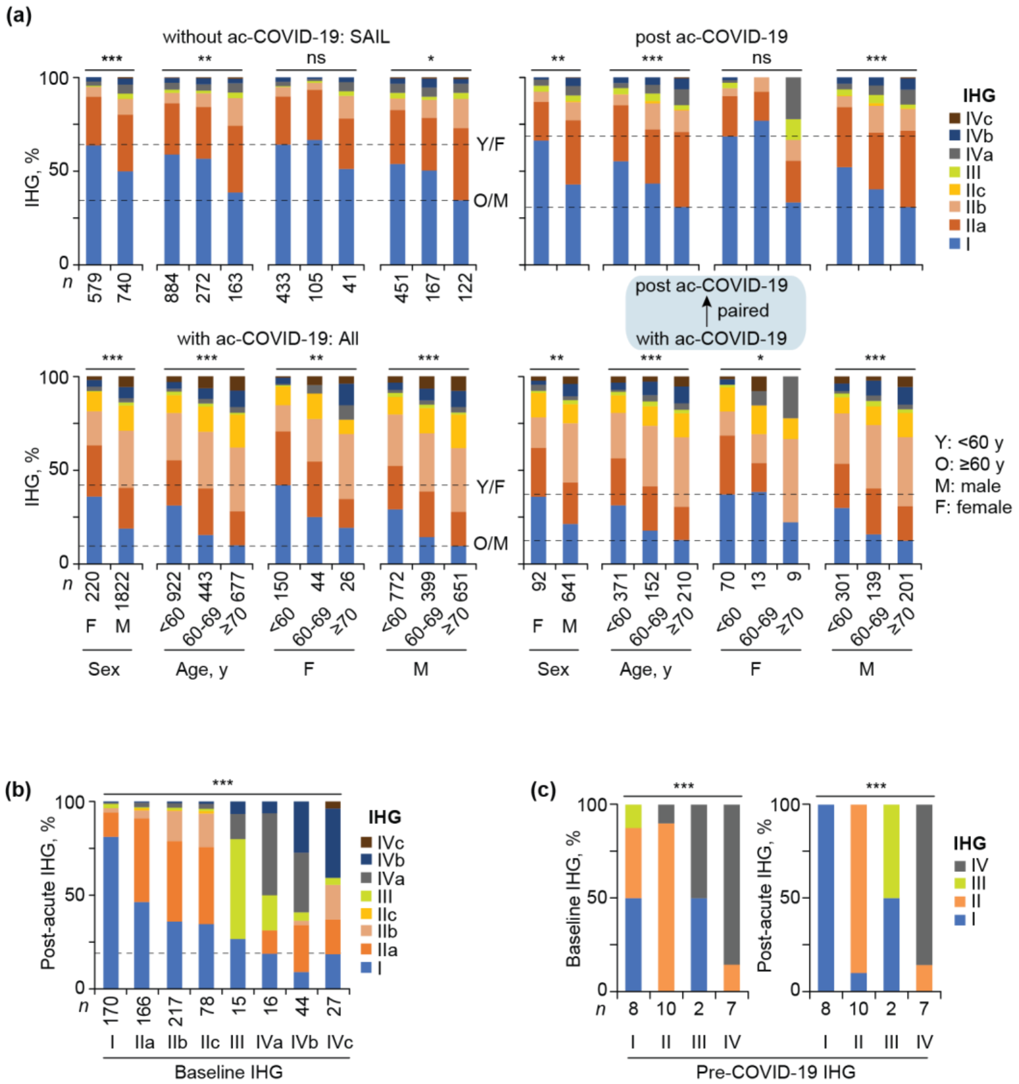
(a)
without ac-COVID-19: SAIL
post ac-COVID-19
with ac-COVID-19: All
post ac-COVID-19
paired
with ac-COVID-19
IHG, %
Y/F
O/M
IHG
IVc
IVb
IVa
III
IIc
IIb
IIa
I
Y: <60 y
O: ≥60 y
M: male
F: female
Sex
Age, y
F
M
(b)
Post-acute IHG, %
Baseline IHG
(c)
Baseline IHG, %
Post-acute IHG, %
Pre-COVID-19 IHG
IV

Figure S28. Reconstitution of immune resilience (IR) during COVID-19 according to the Immune Health Grade (IHG) at baseline in the VA-CLC. IR indexed to IHG status. **(a)** Kaplan-Meier (KM) plots depict time to achieve IHG-I or IHG-IIa (*top row*) and IHG-I (*bottom row*) according to the indicated IHG at presentation (baseline) noted next to each KM plot. *P*, age-adjusted Cox proportional hazards model. **(b)** Odds ratios (OR) with 95% CI of a post-acute IHG-I during convalescence by baseline ac-COVID-19 IHG. x-axis depicts the odds and percentage of achieving IHG-I according to the indicated baseline IHG. Two-sided statistical tests were used. VA-CLC, VA (Veterans Affairs) COVID-19 Longitudinal Cohort. Note: Reduced IHG reconstitution with IHG-IV subgrades. Statistical details are in Section 6.2.28. Data correspond to Figure 6f (main).

Figure S28

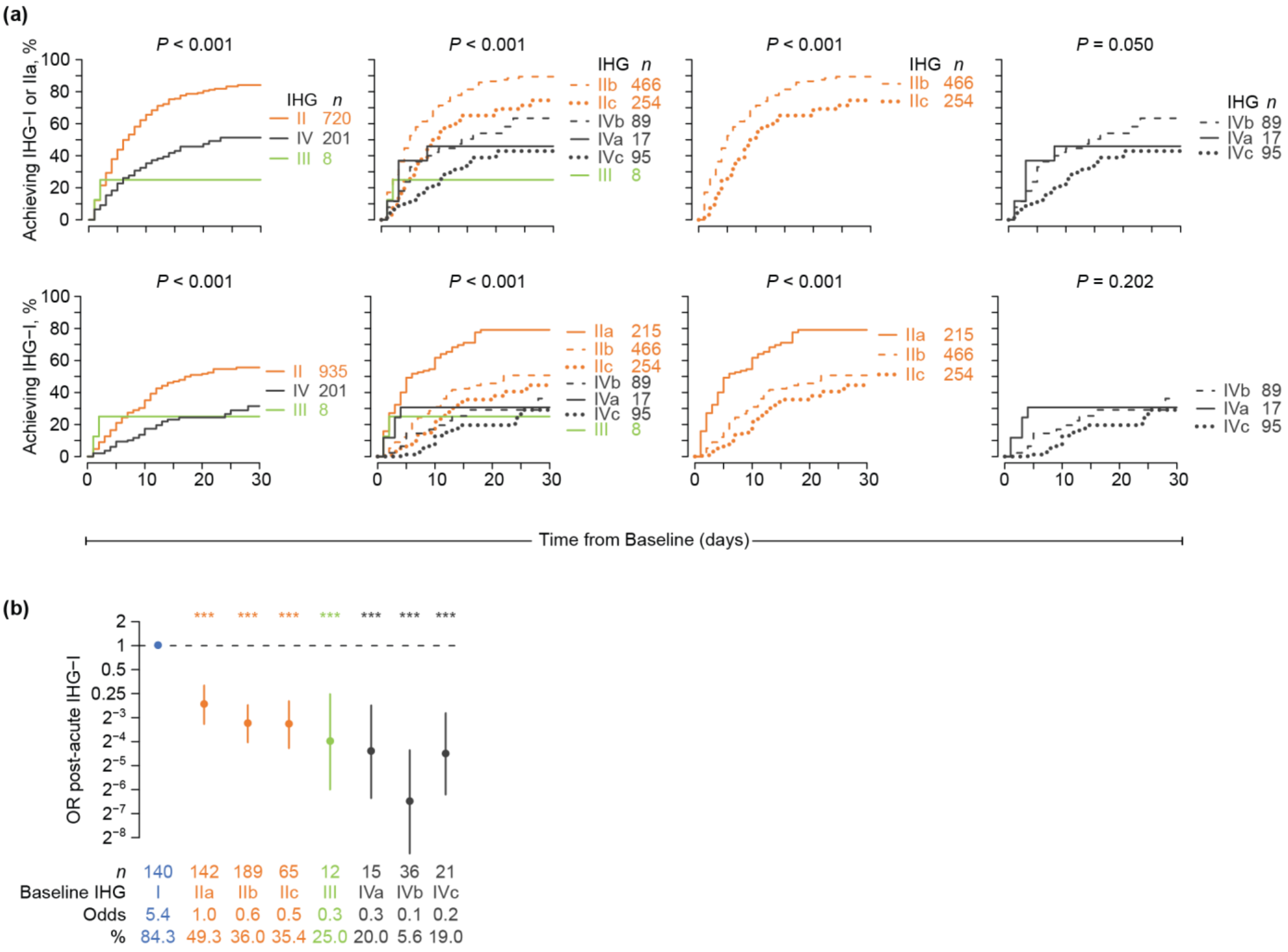

Figure S29. Survival-associated and mortality-associated signature levels by hospitalization and survivorship status and baseline Immune Health Grades (IHG). Cohort: VA-CLC. Linear generalized estimating equation (GEE) models depict the change in expression levels (z-scores) of Survival-associated signature 1 (SAS-1) and Mortality-associated signature 1 (MAS-1) during acute COVID-19 since IHG baseline (0 to 7 days) according to acute COVID-19 outcomes (*top*) and baseline IHG (*bottom*). Shaded areas, pointwise 95% confidence bands. Int. intercept; NH, nonhospitalized; HS, hospitalized survivor; NS, nonsurvivors (30-day survival). SAS, Survival-associated signature; MAS, Mortality-associated signature. Expression data within 7 days from IHG baseline of 45 participants were plotted by baseline IHG and COVID-19 outcomes by 30-day survival status. IHG measurements. IHG baseline is the date of the first available IHG for each participant. There were 3 non-hospitalized individuals with no RNA-Seq measurements within this time window. *P*, linear GEE with analysis of variance (ANOVA). Data from the VA-CLC [VA (Veterans Affairs) COVID-19 Longitudinal Cohort]. <u>Note:</u> Higher expression of SAS-1 and lower expression of MAS-1 in persons presenting with IHG-I vs. IHG-II or IHG-IV. IHG-III is infrequent and was not represented in the dataset. Details of the signatures are in Figure 1d (main) and Table S1. Statistical details are in Section 6.2.29.

Figure S29

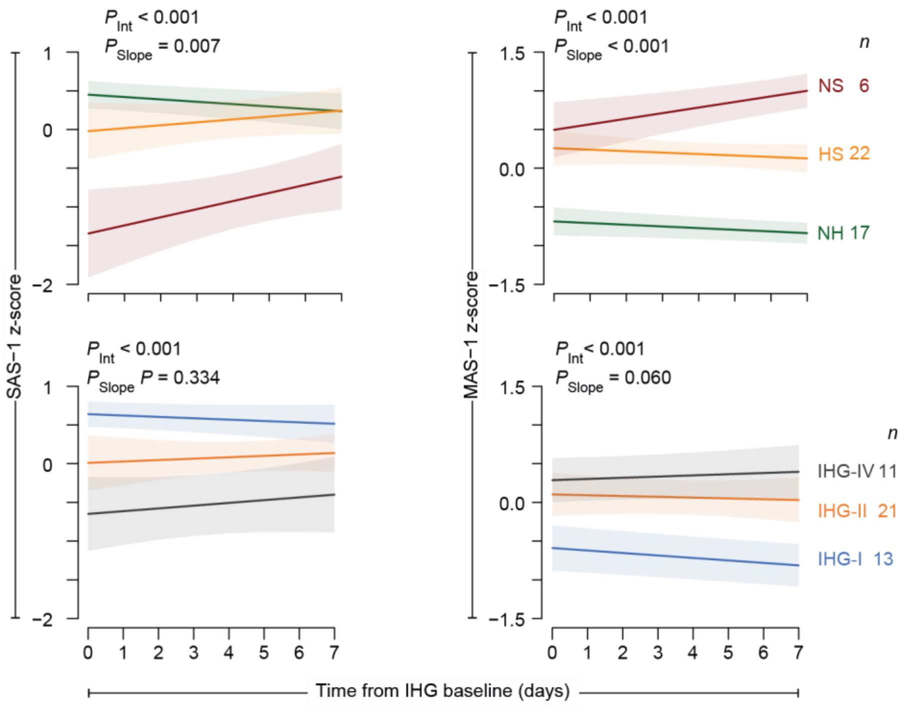

Figure S30. Associations of Immune Health Grades (IHGs) with hospitalization/survival outcomes, serious non-ARDS complications during acute COVID-19 (ac-COVID-19). Cohort: VA-CLC. **(a)** *Top*, IHG distribution and *bottom*, odds ratios (OR) of presenting with IHG-I (blue) *vs.* IHG-II (orange) according to whether persons were non-hospitalized (NH) or hospitalized (H) by indicated age strata. *P*, logistic regression. Note: While hospitalization rates increase with age, in each age stratum, IHG-I is overrepresented in nonhospitalized vs. hospitalized persons. **(b)** Sex-adjusted hazard ratios (aHR) with 95% CI of 30-day acute mortality and post-acute mortality. Analysis by Cox proportional hazards models. **(c)** Probability of 30-day mortality across the age spectrum by IHG baseline. *P*, logistic regression. **(d)** Absolute risk (AR), relative risk (RR), and adjusted population attributable fraction (aPAF) values of 30-day mortality. Note: In the overall cohort, the absolute risk for 30-day mortality associated with older age (≥60 years) was 14%, whereas it was 45% for IHG-IIc and IHG-IVc combined. While the prevalence of IHG-IIc and IHG-IVc is lower in younger persons (Fig. S27a), nearly 44% and 27% of deaths among younger and older patients, respectively, could be attributed to these grades. The relative risk associated with these two grades combined was 10.85 and 5.70 in younger versus older patients. Thus, the overall impact of IHG-IIc and IHG-IVc on 30-day mortality was greater in younger patients. **(e)** 60-day mortality hazards by era (before and on or after October 1st, 2020). aHR, age-adjusted hazard ratio. *P*, Cox proportional hazards models with likelihood ratio test (LRT). **(f)** Rate ratio (RR) with 95% CI of serious non-ARDS complications. A serious complication score was derived (SCS, range: 0-10) and the rate ratio (RR) computed in the indicated groups categorized according to age, sex, and baseline IHG status. aRR, adjusted RR after controlling for age and/or sex as noted on the y-axis. *P*, quasi-Poisson generalized linear models (GLM). Statistical details are in Section 6.2.30. Data in Figure S30b correspond to Figure 7a (main).

Figure S30

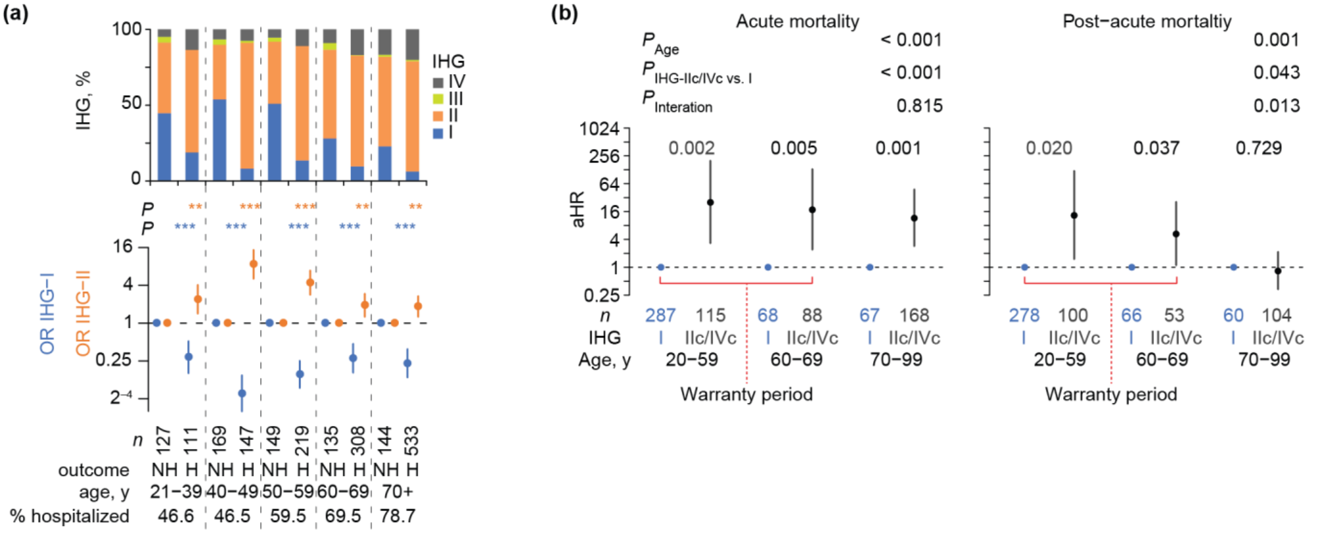

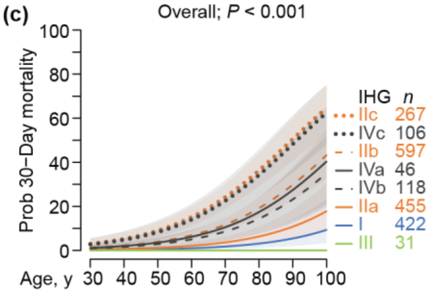

(d)

30-day mortality by IHGs vs. age

	Overall			<60 y			≥60 y		
	**AR,%**	**RR**	**aPAF,%**	**AR,%**	**RR**	**PAF,%**	**AR,%**	**RR**	**PAF,%**
≥60 y	14	6.83	70						
<60 y	2	0.15	–40						
IHG-I	1	0.09	–14	0	0.12	–38	2	0.14	–12
IHG-IIc	22	3.33	21	9	6.83	36	29	2.52	19
IHG-IVc	23	2.93	8	8	4.02	8	29	2.18	8
IIc/IVc	45	6.26	29	17	10.85	44	58	5.70	27

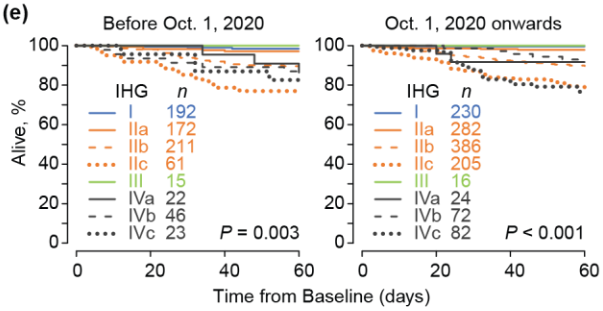

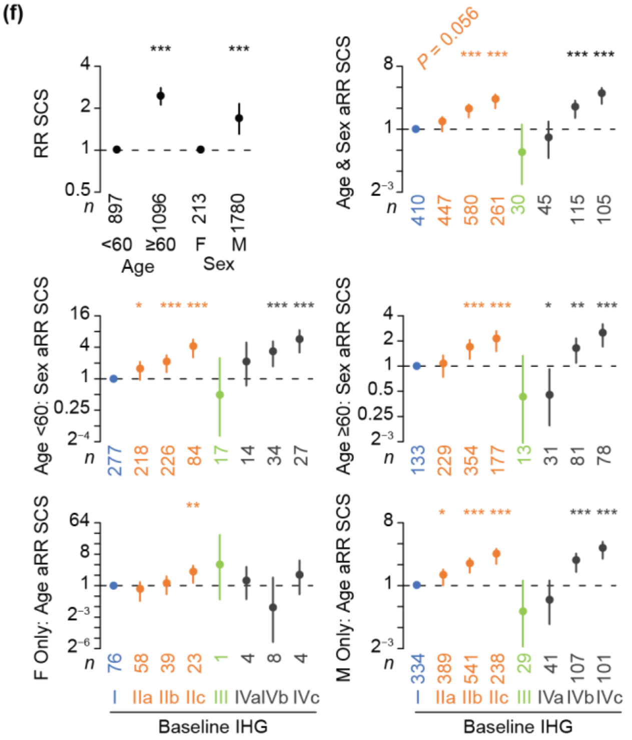

Figure S31. Associations of IR status indexed baseline Immune Health Grades (IHGs) with SARS-CoV-2 viral load, and post-acute conditions and vial load with acute mortality, and biomarkers. Cohort: VA-CLC. **(a)** 30-day mortality by SARS-CoV-2 RT-qPCR cycle threshold (Ct) strata of <30 and ≥30. Ct (SARS-CoV-2 cycle threshold) values were used as a measure of nasopharyngeal SARS-CoV-2 viral load (VL). <30 and ≥30 Ct values indicate higher and lower VL, respectively. *P*, age-adjusted Cox proportional hazards model with LRT. **(b)** Boxplots depict median (IQR) levels of biomarkers (B cells: cells/mm^3; IL-6: pg/mL) by Ct value strata. *P*, linear model with likelihood ratio test. **(c)** Odds of Ct ≥30 by IHG-I vs. non-IHG-I with age. Shaded areas, 95% confidence bands. *P*, logistic regression. **(d)** Age-adjusted odds ratio (aOR) with 95% CI of Ct ≥30 according to baseline IHG. *P*, logistic regression. **, *P*<0.01; *, *P*<0.05. <u>Note:</u> Higher VL associated with a 2.76 higher 30-day mortality hazard, adjusted for age (aHR, 2.76; 95% CI: 1.51-5.03; panel a). Lower VL associated with higher levels of B cells and lower levels of IL-6 (panel b). The likelihood of having a lower VL (Ct ≥30) decreased with age (panel c). However, across the age spectrum, presentation with IHG-I versus a non-IHG-I grade was associated with lower VL; the difference in VL between IHG-I vs. non-IHG-I grade was mathematically modeled to be equivalent to 69 years of younger age (panel c). Each 10-year increase in age associated with a 10% lower likelihood of having a lower VL (aOR=0.90; 95% CI=0.81-0.99; *P*=0.027), whereas presentation with a non-IHG-I grade associated with a 53% lower likelihood having a lower VL (aOR=0.47; 95% CI=0.29-0.74; *P*=0.001). Baseline IHG-IIc and IHG-IVc associated with the lowest likelihood of having a lower VL (panel d). **(e)** Odds ratio (OR) with 95% CI of a post-acute condition (ICD-10-CM code U09.9) by age strata and sex. *P*, univariate logistic regression models. **(f)** Age- and sex-adjusted ORs with 95% CI of post-acute COVID-19 condition by baseline IHG. *P*, logistic regression. ***, *P*<0.001; **, *P*<0.01; *, *P*<0.05. Statistical details are in Section 6.2.31.

Figure S31

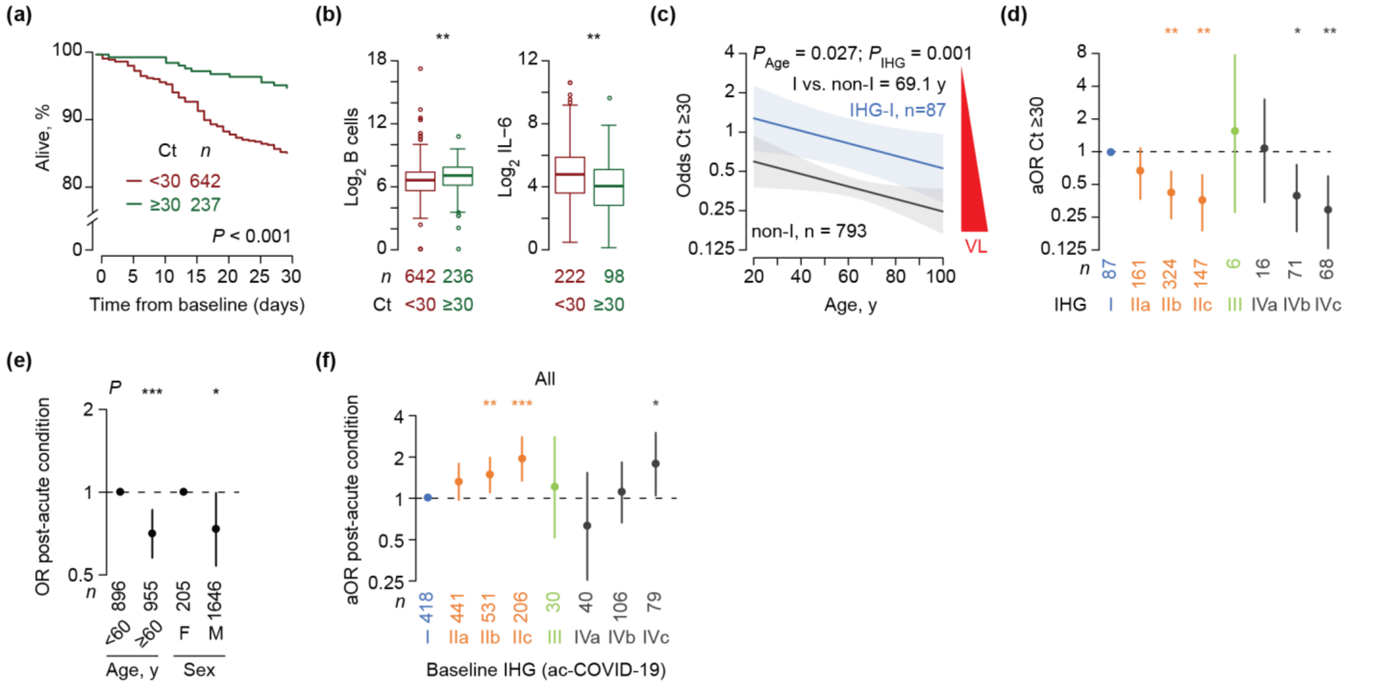

(a)
100
90
80
0
Alive, %
Ct n
<30 642
≥30 237
P < 0.001
0 5 10 15 20 25 30
Time from baseline (days)
(b)
**
18
12
6
0
Log2 B cells
**
12
8
4
0
Log2 IL−6
n 642 236
Ct <30 ≥30
222 98
<30 ≥30
(c)
PAge = 0.027; PIHG = 0.001
I vs. non-I = 69.1 y
IHG-I, n=87
non-I, n = 793
4
2
1
0.5
0.25
0.125
Odds Ct ≥30
20 40 60 80 100
Age, y
VL
(d)
** ** * **
8
4
2
1
0.5
0.25
0.125
aOR Ct ≥30
n 87 161 324 147 6 16 71 68
IHG I IIa IIb IIc III IVa IVb IVc
(e)
P *** *
2
1
0.5
OR post-acute condition
n 896 955 205 1646
<60 ≥60 F M
Age, y Sex
(f)
All
** *** *
4
2
1
0.5
0.25
aOR post-acute condition
n 418 441 531 206 30 40 106 79
I IIa IIb IIc III IVa IVb IVc
Baseline IHG (ac-COVID-19)

Figure S32. Improvements in immune resilience (IR) associated with survival during acute and post-acute phase of COVID-19. Cohort: VA-CLC. **(a)** Kaplan-Meier plots depict 30-day survival (*left*) and survival in the post-acute mortality window (*right*) according to IR improvement status within 5 days of presentation. Schema to the right depict the derivation of the acute and post-acute mortality window. *P*, Cox proportional hazards models adjusted by age and/or number of IHG measures within the first 5 days from baseline. **(b)** Kaplan-Meier plots depict survival in 30-day ac-COVID-19 window (*left column*) and survival in the post-acute mortality windows (*right column*) by IR improvement, in <60 years (*top*) and ≥60 years (*bottom*) old. *P*, Cox proportional hazards model adjusted by age and/or number of IHG measurements each patient had within the first 5 days. Please see methods for definitions of no improvement vs. improvement based on IHG criteria. Statistical details are in Section 6.2.32.

Figure S32

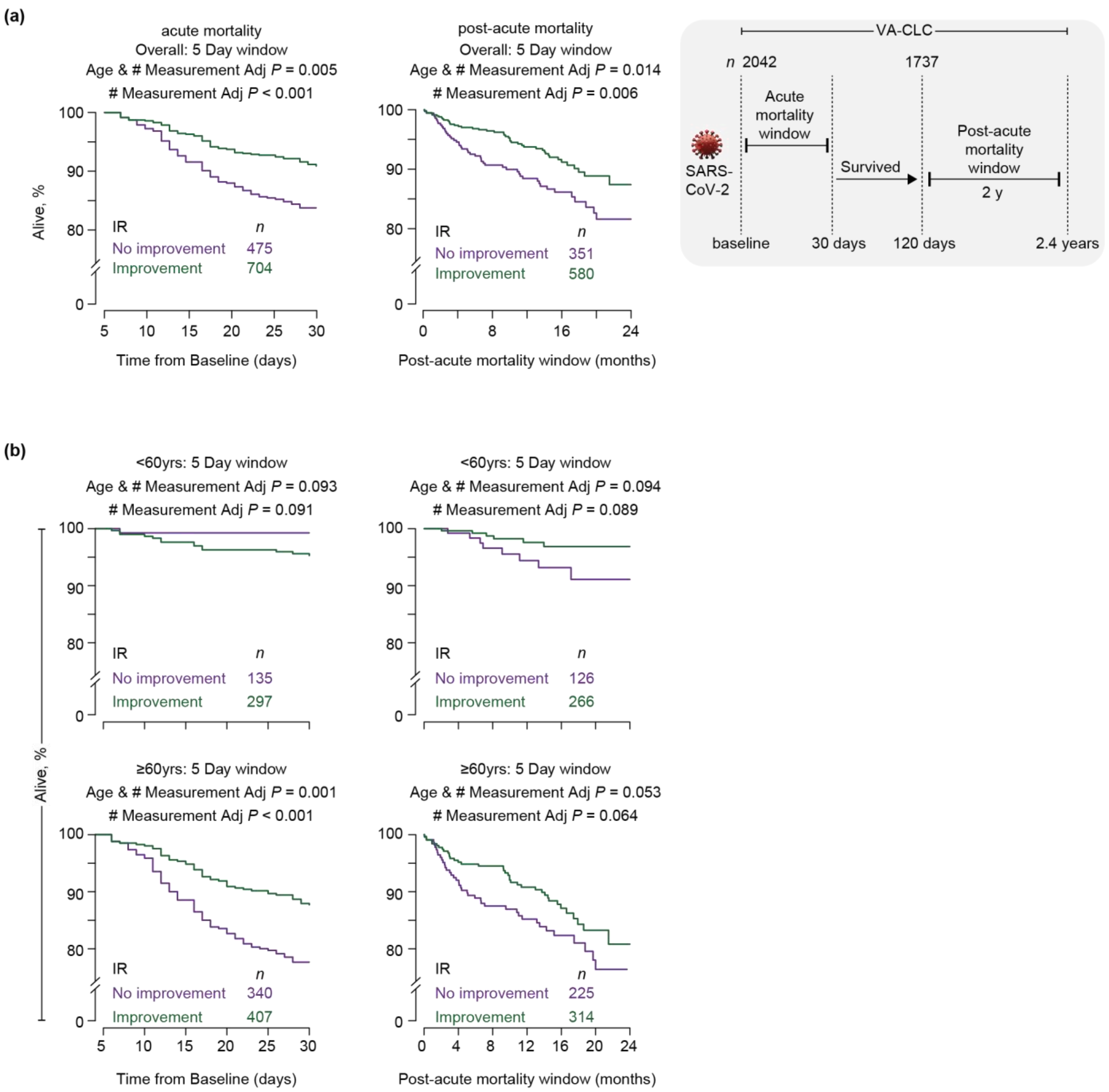
(a)
acute mortality
Overall: 5 Day window
Age & # Measurement Adj P = 0.005
Measurement Adj P < 0.001
Alive, %
IR n
No improvement 475
Improvement 704
Time from Baseline (days)
post-acute mortality
Overall: 5 Day window
Age & # Measurement Adj P = 0.014
Measurement Adj P = 0.006
IR n
No improvement 351
Improvement 580
Post-acute mortality window (months)
VA-CLC
n 2042
1737
Acute mortality window
SARS-CoV-2
Survived
Post-acute mortality window
2 y
baseline
30 days
120 days
2.4 years
(b)
<60yrs: 5 Day window
Age & # Measurement Adj P = 0.093
Measurement Adj P = 0.091
IR n
No improvement 135
Improvement 297
<60yrs: 5 Day window
Age & # Measurement Adj P = 0.094
Measurement Adj P = 0.089
IR n
No improvement 126
Improvement 266
Alive, %
≥60yrs: 5 Day window
Age & # Measurement Adj P = 0.001
Measurement Adj P < 0.001
IR n
No improvement 340
Improvement 407
≥60yrs: 5 Day window
Age & # Measurement Adj P = 0.053
Measurement Adj P = 0.064
IR n
No improvement 225
Improvement 314
Time from Baseline (days)
Post-acute mortality window (months)

Figure S33. Association of IR status and SARS-CoV-2 neutralizing antibody (nAb) responses during natural infection and following vaccination. Cohorts: **a-c,** VA-CLC; **d-e**, SAIL pre-booster cohort. **(a)** Adjusted odds ratios (aOR) with 95% CI of SARS-CoV-2 surrogate virus neutralization test (sVNT) %inhibition <30% (absent nAb response) at baseline by age, sex, and days since onset of symptoms (DoS; ≤7 vs. >7 days). *P*, logistic regression. **(b)** Age and DoS-adjusted ORs with 95% CI of sVNT %inhibition <30% at baseline according to (*left to right*) baseline IHG, IHG subgrades, and indicated COVID-19 outcomes. NH, nonhospitalized; H, hospitalized; HS, hospitalized survivors; NS, nonsurvivors (30-day survival). *P*, logistic regression. **(c)** Change in the odds of sVNT %inhibition ≥30%. Shaded areas indicate a pointwise confidence band as a quadratic function of days since baseline in the acute COVID-19 (ac-COVID-19) outbreak subset (*n*=61); concurrent IHG and sVNT %inhibition values were used in these analyses (median samples per person: 2 [IQR: 1-2]). IHG-III is infrequent and was not represented in the dataset. *P*, logistic GEE with an AR(1) correlation structure as a quadratic function of days from baseline (COVID-19 diagnosis) by IHG. <u>Note:</u> We modeled %inhibition over a 400-day period from diagnosis in 77 patients who became infected with SARS-CoV-2 in a nursing home in March 2020 or January 2021 (median baseline age (IQR): 78 (73-88) years; 173 plasma samples). Baseline IHG-I was associated with a higher likelihood of maintaining %inhibition ≥30% compared to IHG-II or IHG-IV (*P*=0.005 and *P*<0.001, respectively). Thus, panels b and c show that compared with IHG-I, baseline IHG-II and particularly IHG-IV associated with lower nAb responses at baseline and in the early and post-acute phases of COVID-19. **(d)** Age-adjusted (adj.) odds of sVNT %inhibition ≥30%. Shaded areas indicate 95% confidence band as a function of days since last vaccination before booster by IHG in the SAIL cohort adjusted by age and previous COVID-19. *P*, logistic regression. <u>Note:</u> IHG-IV *vs.* IHG-I associated with lower odds of %inhibition ≥30% across nearly 350 days post-vaccination, controlling for age and previous COVID-19. **(e)** aOR with 95% CI of sVNT %inhibition <30% in the SAIL cohort by IHG status, prior COVID-19, and age strata, adjusted for days since last vaccination. *P*, logistic regression. <u>Note:</u> In multivariate analysis, IHG-IV associated independently with nearly a 3-fold higher likelihood of having an absent or lower nAb response (%inhibition <30%) vs. IHG-I, after controlling for age, days since vaccination, and history of COVID-19. Statistical details are in Section 6.2.33.

Figure S33

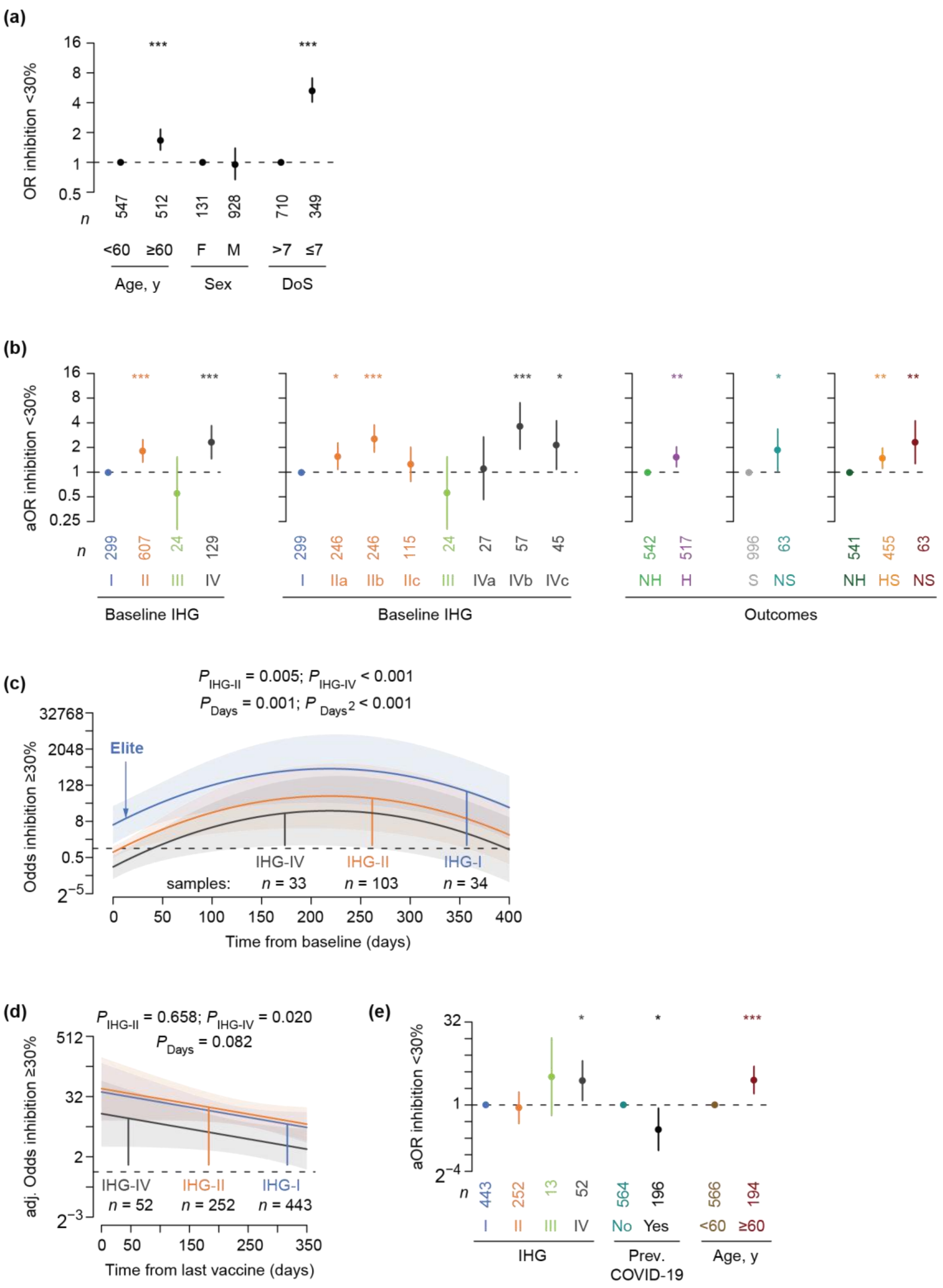

3273

3274

Figure S34. Association of immune resilience (IR) metrics, gene signatures tracking the pathogenic triad and vaccine responses, and *TCF7* expression levels in the fluzone trivalent influenza vaccination (TIV) cohort during 2011/12 flu season (SDY296). (a) Stacked barplots depict the distribution of SAS-1/MAS-1 profiles and dot-and-line plots depict median (IQR) of *TCF7* (NGE) according to pre-vaccination IHG status (IHG-I, IHG-IIa) and days since vaccination. Note: Proportion of extreme IR-degrader status is greater in person with pre-vaccination IHG-IIa *vs.* IHG-I, marked by increased prevalence of SAS-1^{low}-MAS-1^{high} day 1 post-vaccination. IHG-I and IHG-II are the most and second-most prevalent grades in control human populations (Fig. S26b), respectively. **(b)** Line plots (mean ± SEM) of indicated gene signatures (z-scores) and *TCF7* levels (NGE) at the indicated timepoints. Among the gene signatures are those tracking interferon-stimulated genes (ISG_33, ISG_23) and four gene modules associated with vaccine responses (Supplementary section 4.8). NGE, normalized gene expression. Note: Levels of some gene signatures/modules differ by baseline IHG status. Details of the signatures are in Figure 1d (main) and Table S1 and methods. Higher levels of the IMM-AGE signature were computed to signify an association with fewer senescent T-cells (less immune aging and lower mortality; a {+}-salutogenesis readout), as detailed in Section 4.2. Statistical details are in Section 6.2.34.

Figure S34a: SAS-1/MAS-1 distributions & *TCF7* levels

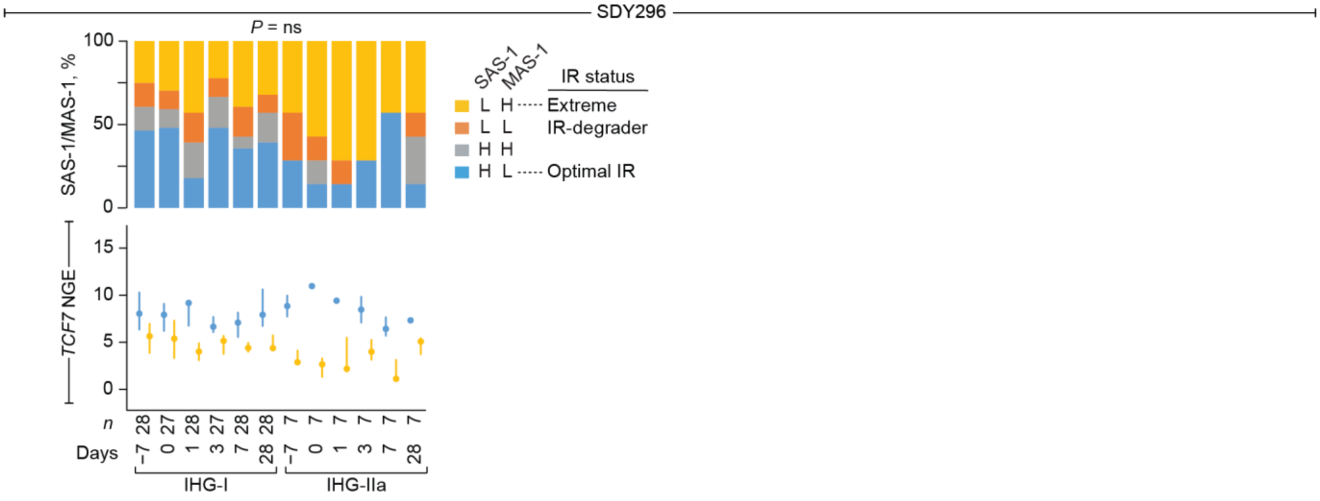

Figure S34b: Signature/gene trajectories by IHG-I vs. IHG-IIa

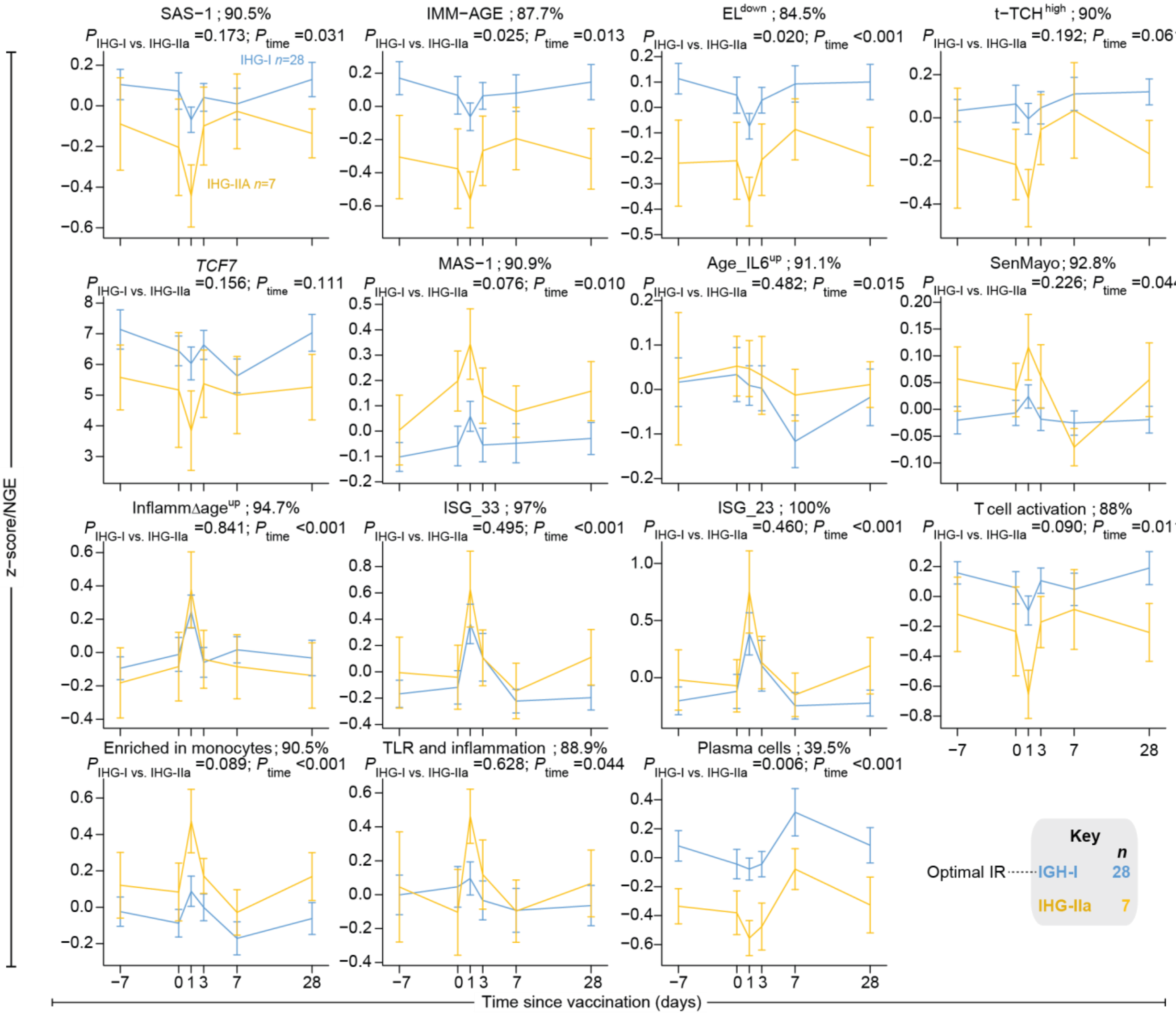

Figure S35. Associations of baseline Immune Health Grade (IHG) status and pre-existing comorbidity status in VA-CLC participants. (a) Rate ratios with 95% CI of comorbidity burden by age strata. Note: Presentation with extreme IR-degrader (IHG-IIc/IHG-IVc) vs. optimal IR (IHG-I) status is associated with increased burden of pre-existing comorbidities **(b)** Age-adjusted comorbidity rates (the average number of comorbidities per patient) with 95% CI of (*top*) 19 and (*bottom*) 8 comorbidity conditions that associated with mortality during the post-acute mortality window by baseline IHG status. Comorbidity conditions were recorded at baseline. *P*, quasi-Poisson generalized linear model. Schema to the left depict the derivation of the comorbidities that associate with post-acute mortality in the VA-CLC. Obesity (BMI ≥30 kg/m^2) associated with a lower age-adjusted post-acute mortality hazard. Note: age-adjusted comorbidity rate was higher in extreme IR-degrader (IHG-IIc or IHG-IVc) vs. optimal (IHG-I) status. **(c)** Percent with 0 or 1 comorbidities (comorbidity score: 0-1) across the age spectrum in those presenting with the indicated IHGs with subgrades. Shaded regions depict 95% confidence bands. *P*, logistic regression. Note: Participants presenting with IHG-I were more likely to have 0-1 comorbidities across the age spectrum. Statistical details are in Section 6.2.35.

Figure S35

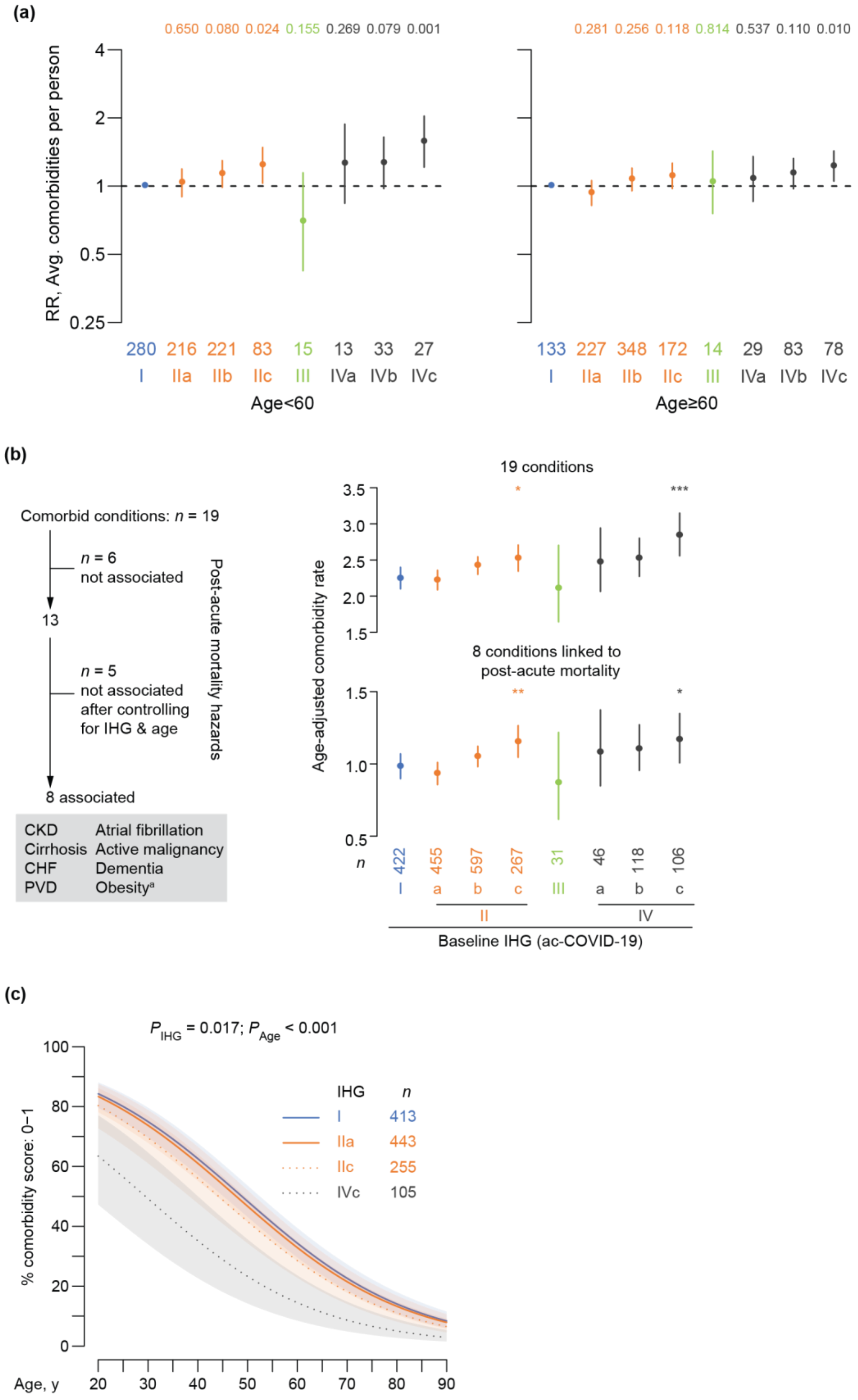
(a)
0.650 0.080 0.024 0.155 0.269 0.079 0.001
0.281 0.256 0.118 0.814 0.537 0.110 0.010
RR, Avg. comorbidities per person
4
2
1
0.5
0.25
280 216 221 83 15 13 33 27
I IIa IIb IIc III IVa IVb IVc
Age<60
133 227 348 172 14 29 83 78
I IIa IIb IIc III IVa IVb IVc
Age≥60
(b)
Comorbid conditions: n = 19
n = 6
not associated
13
n = 5
not associated
after controlling
for IHG & age
8 associated
Post-acute mortality hazards
CKD Atrial fibrillation
Cirrhosis Active malignancy
CHF Dementia
PVD Obesity[a]
19 conditions
3.5
3.0
2.5
2.0
1.5
*
***
8 conditions linked to
post-acute mortality
**
*
1.5
1.0
0.5
Age-adjusted comorbidity rate
n
422 455 597 267 31 46 118 106
I a b c III a b c
II
IV
Baseline IHG (ac-COVID-19)
(c)
P_IHG = 0.017; P_Age < 0.001
100
80
60
40
20
0
% comorbidity score: 0–1
IHG n
I 413
IIa 443
IIc 255
IVc 105
Age, y
20 30 40 50 60 70 80 90

Figure S36. Association of baseline Immune Health Grade (IHG) status with expression levels of the gene signatures tracking IR status and pathogenic triad, and *TCF7* in the VA-CLC. Boxplots depict median (IQR) expression of indicated gene signatures and *TCF7* (NGE) by baseline IHG groupings (IHG-I vs. rest), baseline IHG with subgrades, SAS-1/MAS-1 profiles, and COVID-19 outcomes. IHG-III is infrequent and was not represented in the dataset. *P*, linear model with likelihood ratio test (LRT). Expression data from RNA-Seq baseline of 48 participants was to generate the plots and determine the SAS-1/MAS-1 profiles (*left to right*): SAS-1^{high}-MAS-1^{low} (H-L; optimal IR status); SAS-1^{high}-MAS-1^{high} (H-H); SAS-1^{low}-MAS-1^{low} (L-L); and SAS-1^{low}-MAS-1^{high} (L-H; extreme-IR degrader status). RNA-Seq baseline is the date of the first available RNA-Seq data for each participant. COVID-19 outcomes were determined by 30-day survival status. Details of the signatures are in Figure 1d (main) and Table S1. Higher levels of the IMM-AGE signature were computed to signify an association with fewer senescent T-cells (less immune aging and lower mortality; a {+}-salutogenesis readout), as detailed in Section 4.2. Statistical details are in Section 6.2.36. Data correspond to Figure 7c (main).

Figure S36 (leftmost corresponds to main Figure 7c)

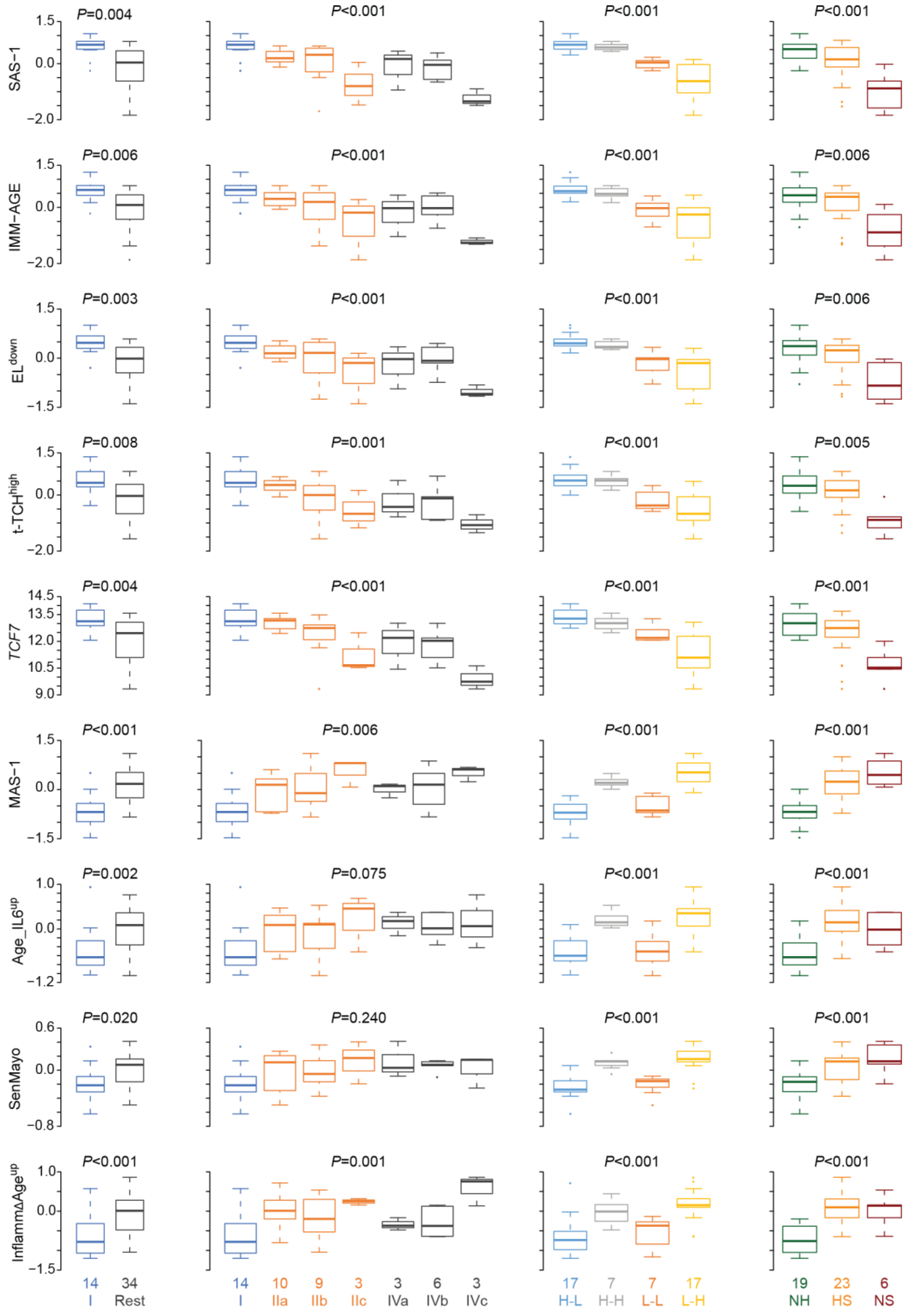

Figure S37. Association of baseline IHG status with biomarkers levels across the age spectrum in the VA-CLC. (a) Trajectories of log_2-transformed biomarker levels in patients stratified by baseline IHG status at presentation with acute COVID-19. Shaded areas, 95% confidence bands. *P* for effects of age and differences between IHG groups, by linear regression model. NE, neutrophils; NK, natural killer cells; C reactive protein (CRP). Note: Biomarkers were evaluated as part of standard-of-care. IHG-I and IHG-IIa are the most- and second-most prevalent grades, respectively, in control human populations (Fig. S26B). **(b)** Trajectories of log_2-transformed biomarker levels in patients with baseline IHG-I vs. IHG-IIc/IVc grade at presentation with acute COVID-19. Shaded areas, 95% confidence bands. *P,* for effects of age and differences between IHG-I vs. IHG-IIc/IVc grade by linear regression model for all biomarkers expect probability of %inhibition (logistic regression model). Units. Neutrophils, NK cells, and B cells, cells/mm^3; IL-6, pg/mL; CRP, mg/L, D-dimer and ferritin, ng/L; sVNT, SARS-CoV-2 surrogate virus neutralization test; Ct, SARS-CoV-2 cycle threshold. Statistical details in Section 6.2.37. Data in Figure S37a correspond to Figure 7d (main).

Figure S37a (corresponds to main Figure 7d)

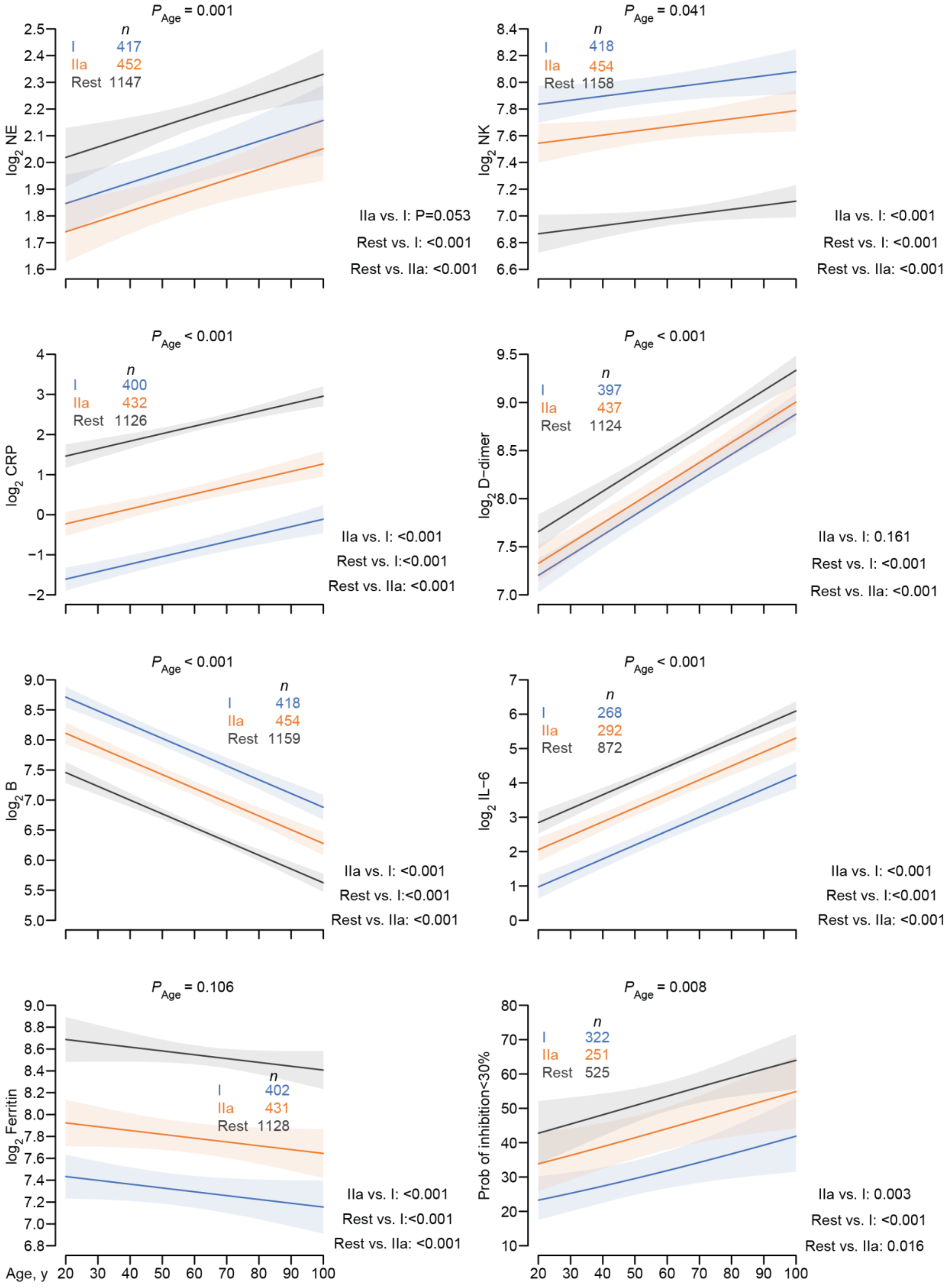

3348

Figures S37b

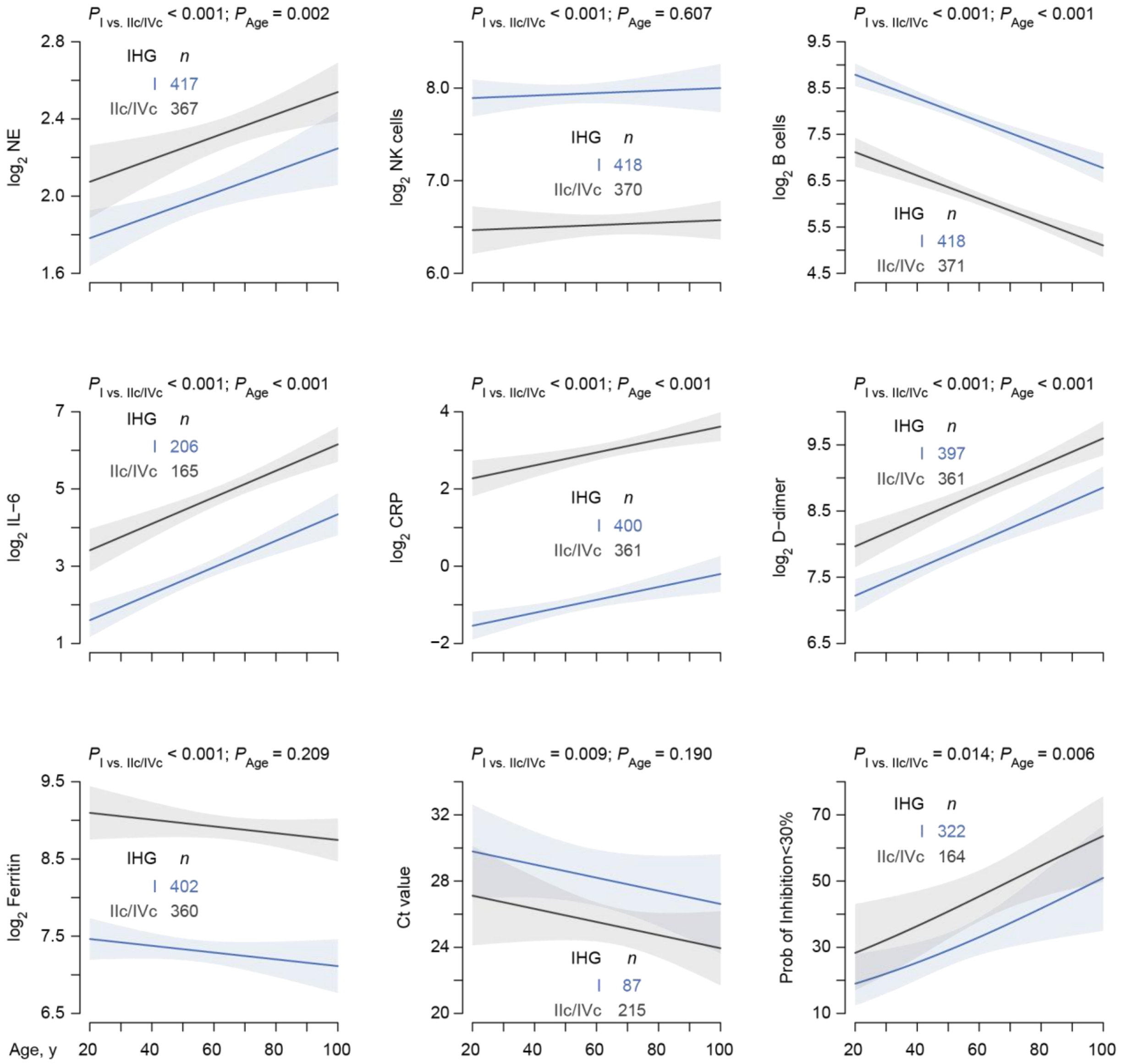

Figure S38. Association of baseline IHG status with biomarker levels at baseline and during the first days since presentation in the VA-CLC. (a) Baseline levels of $\log_2$-transformed biomarker measurements by (*left to right*): IHG status, outcomes, SARS-CoV-2 neutralizing antibody status proxied by sVNT %inhibition <30% vs. ≥30%; younger vs. older age, and sex. NH, hospitalized; HS, hospitalized survivors; NS, nonsurvivors (30-day survival). NE, neutrophils; NK, natural killer cells; C reactive protein (CRP). *P*, linear regression with likelihood ratio test (LRT) for indicated comparison. **(b)** Trajectories of modeled $\log_2$-transformed biomarker measurements with pointwise 95% confidence bands (shaded area) during the first 7 days of acute COVID-19 by following predictors (*left to right*): baseline IHG, IHG subgrades, COVID-19 outcomes, and indicated age strata. *P*, linear GEE testing for interaction of predictors and time. Indicated *n* values are numbers of biomarker measurements. Units. NK, and B cells: cells/mm^3; IL-6: pg/mL; CRP: mg/L; D-dimer and ferritin: ng/L. Statistical details are in Section 6.2.38.

Figure S38a

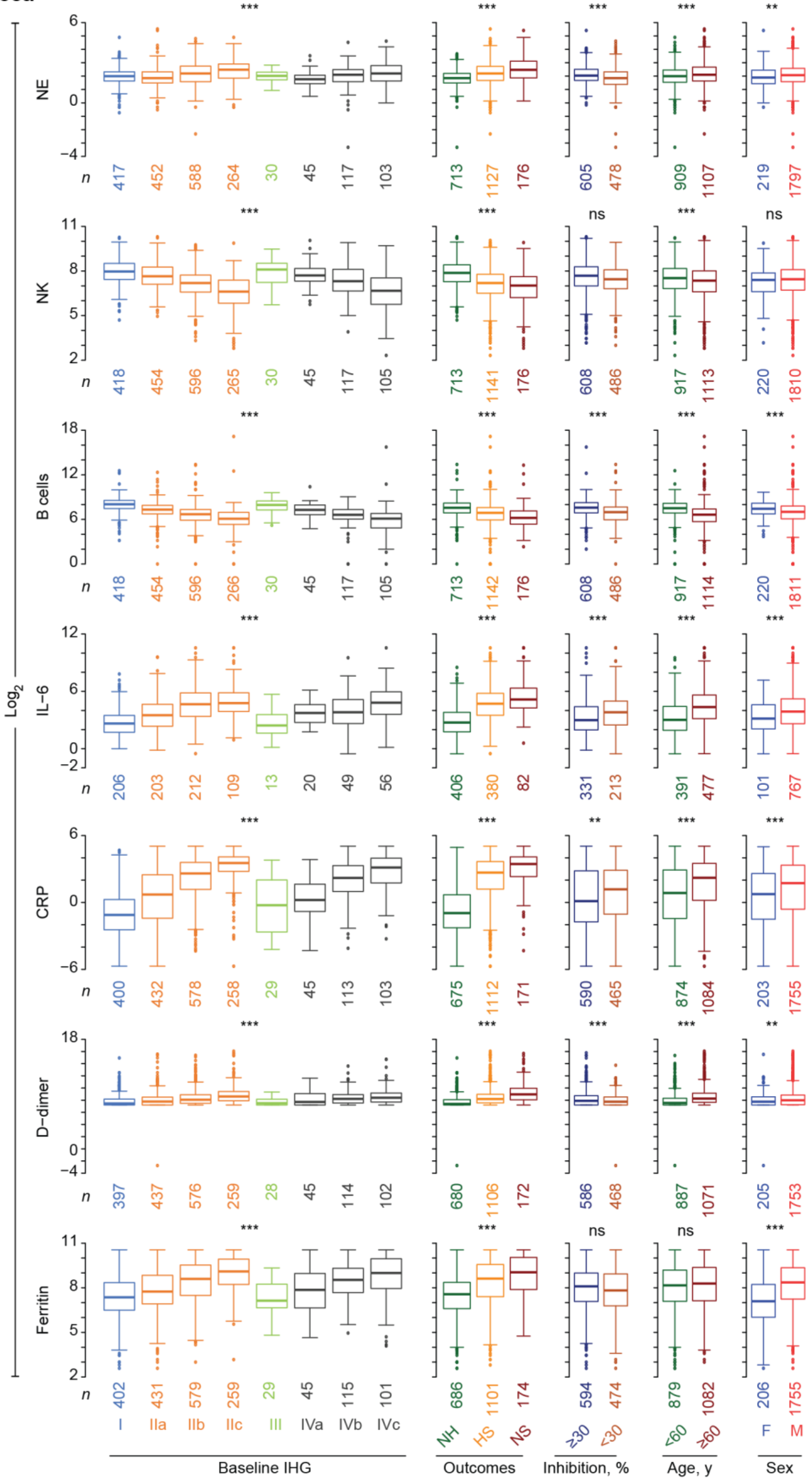
Log2
NE
n 417 452 588 264 30 45 117 103 713 1127 176 605 478 909 1107 219 1797
NK
n 418 454 596 265 30 45 117 105 713 1141 176 608 486 917 1113 220 1810
B cells
n 418 454 596 266 30 45 117 105 713 1142 176 608 486 917 1114 220 1811
IL-6
n 206 203 212 109 13 20 49 56 406 380 82 331 213 391 477 101 767
CRP
n 400 432 578 258 29 45 113 103 675 1112 171 590 465 874 1084 203 1755
D-dimer
n 397 437 576 259 28 45 114 102 680 1106 172 586 468 887 1071 205 1753
Ferritin
n 402 431 579 259 29 45 115 101 686 1101 174 594 474 879 1082 206 1755
I IIa IIb IIc III IVa IVb IVc
NH HS NS
≥30 <30
<60 ≥60
F M
Baseline IHG
Outcomes
Inhibition, %
Age, y
Sex

Figure S38b

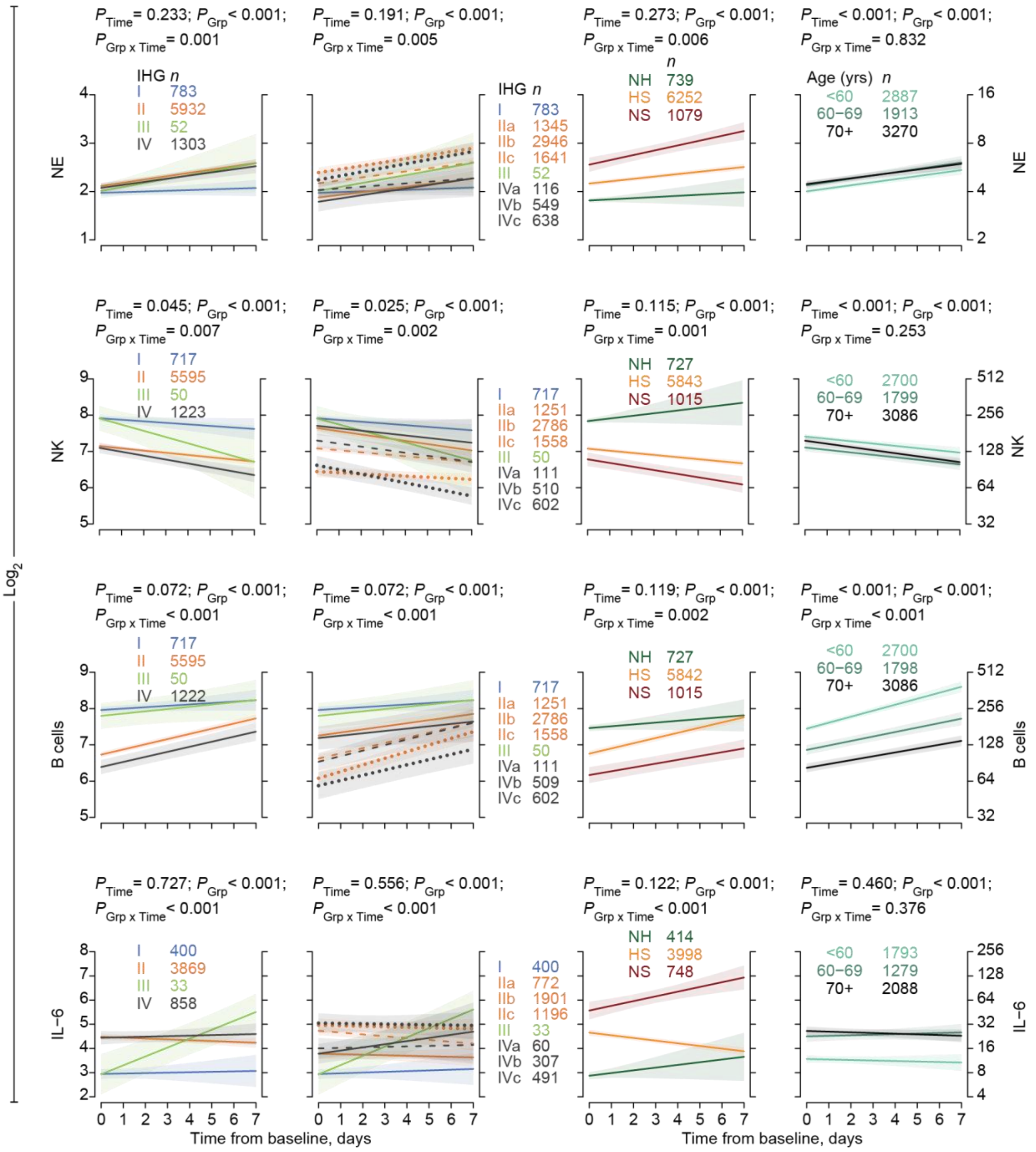

P Time = 0.233; P Grp < 0.001; P Grp x Time = 0.001
P Time = 0.191; P Grp < 0.001; P Grp x Time = 0.005
P Time = 0.273; P Grp < 0.001; P Grp x Time = 0.006
P Time < 0.001; P Grp < 0.001; P Grp x Time = 0.832
IHG n
I 783
II 5932
III 52
IV 1303
IHG n
I 783
IIa 1345
IIb 2946
IIc 1641
III 52
IVa 116
IVb 549
IVc 638
n
NH 739
HS 6252
NS 1079
Age (yrs) n
<60 2887
60–69 1913
70+ 3270
NE
P Time = 0.045; P Grp < 0.001; P Grp x Time = 0.007
P Time = 0.025; P Grp < 0.001; P Grp x Time = 0.002
P Time = 0.115; P Grp < 0.001; P Grp x Time = 0.001
P Time < 0.001; P Grp < 0.001; P Grp x Time = 0.253
I 717
II 5595
III 50
IV 1223
I 717
IIa 1251
IIb 2786
IIc 1558
III 50
IVa 111
IVb 510
IVc 602
NH 727
HS 5843
NS 1015
<60 2700
60–69 1799
70+ 3086
NK
P Time = 0.072; P Grp < 0.001; P Grp x Time < 0.001
P Time = 0.072; P Grp < 0.001; P Grp x Time < 0.001
P Time = 0.119; P Grp < 0.001; P Grp x Time = 0.002
P Time < 0.001; P Grp < 0.001; P Grp x Time < 0.001
I 717
II 5595
III 50
IV 1222
I 717
IIa 1251
IIb 2786
IIc 1558
III 50
IVa 111
IVb 509
IVc 602
NH 727
HS 5842
NS 1015
<60 2700
60–69 1798
70+ 3086
B cells
P Time = 0.727; P Grp < 0.001; P Grp x Time < 0.001
P Time = 0.556; P Grp < 0.001; P Grp x Time < 0.001
P Time = 0.122; P Grp < 0.001; P Grp x Time < 0.001
P Time = 0.460; P Grp < 0.001; P Grp x Time = 0.376
I 400
II 3869
III 33
IV 858
I 400
IIa 772
IIb 1901
IIc 1196
III 33
IVa 60
IVb 307
IVc 491
NH 414
HS 3998
NS 748
<60 1793
60–69 1279
70+ 2088
IL–6
Log2
Time from baseline, days
Time from baseline, days

Figure S38b (continued)

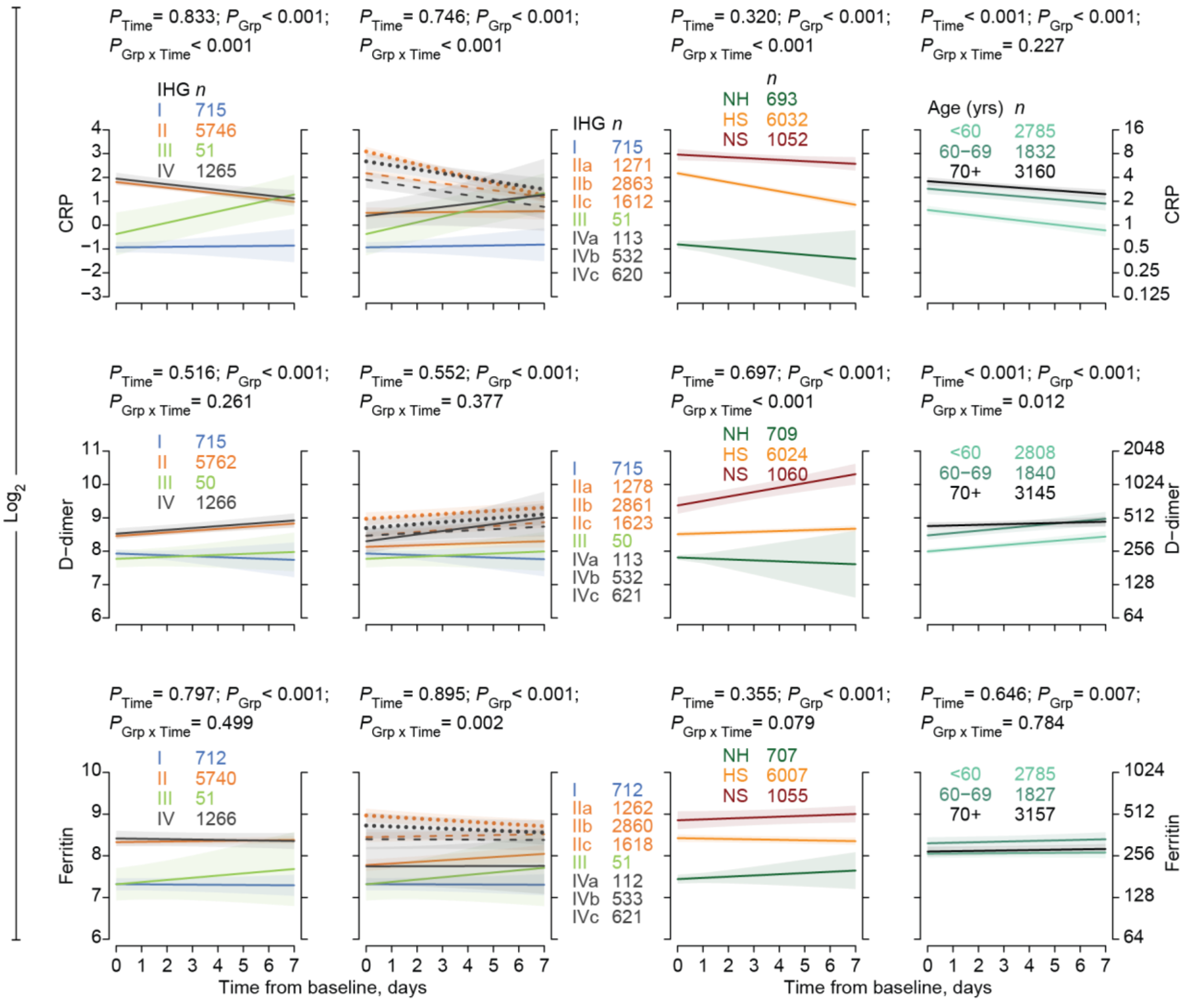

3366

Figure S39. Association of baseline IHG status and pre-existing comorbidity burden with biomarker levels at presentation in the VA-CLC. Adjusted mean (Adj. mean) values with 95% CI of log_2-transformed biomarker measurements [(neutrophils (NE), natural killer (NK), C reactive protein (CRP)] by baseline IHG status and comorbidity burden score (0-1, 2-3. and 4+, based on 19 comorbidities recorded at baseline). *Left*, biomarker levels in those with 0-1 vs. 4+ comorbidities by the presenting IHGs. *Right*, biomarker levels in those with comorbidity burden score 0-1, 2-3, and 4+ by IHG-I vs. IHG-IIc and/or IHG-IVc. Co., comorbidity burden score. *P*, linear regression with contrasts. Statistical details are in Section 6.2.39.

Figure S39 (rightmost corresponds to main Figure 7e)

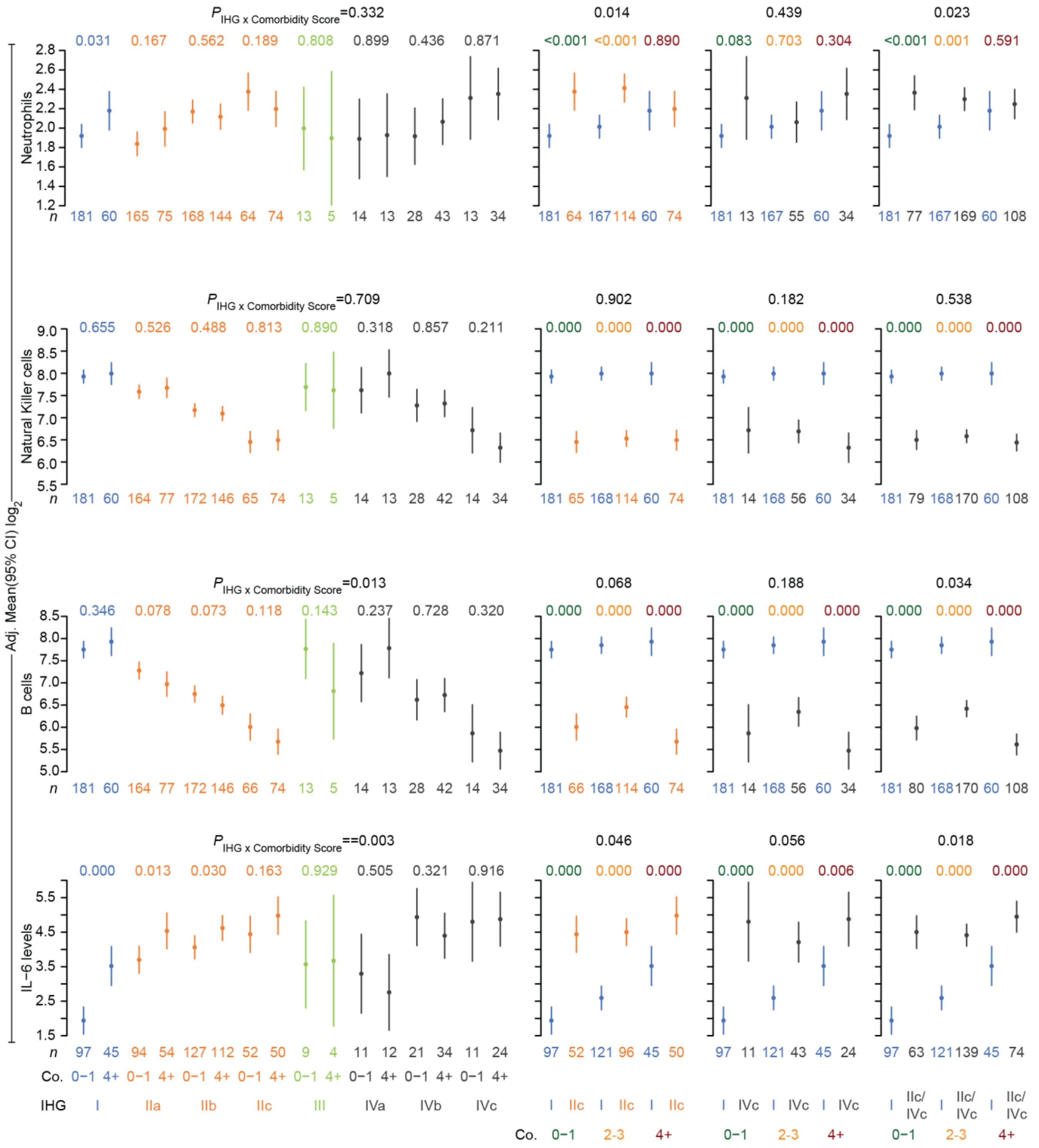

3377

Figure S39 (continued-rightmost corresponds to main Figure 7e)

3378 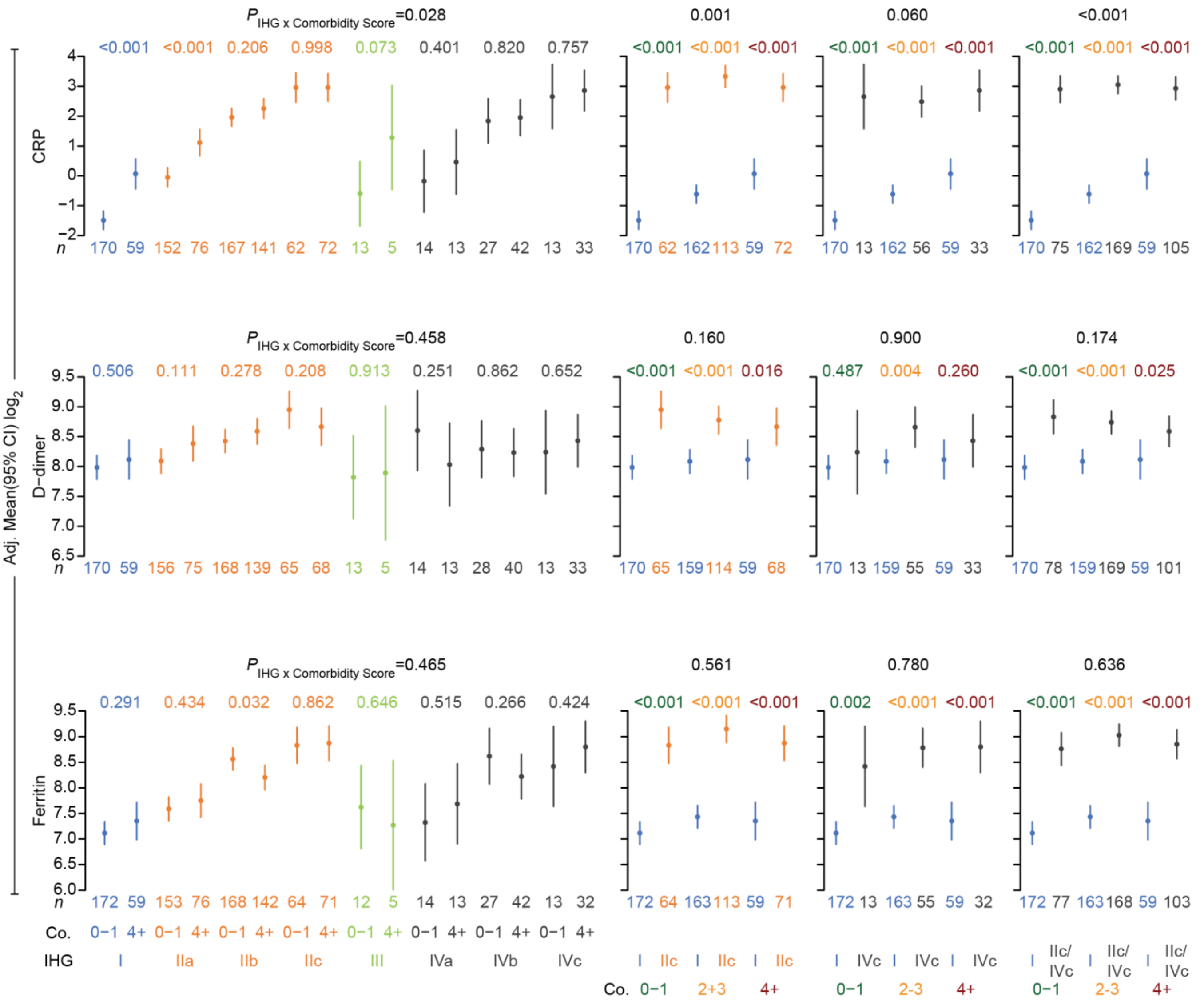

Figure S40. Association of baseline IHG status and obesity status with biomarker levels at presentation in the VA-CLC. Adjusted mean (Adj. mean) values with 95% CI of log_2-transformed biomarker measurements [neutrophils (NE), natural killer (NK), C reactive protein (CRP)] by obesity status overall, and IHG and obesity status. *P*, linear regression with contrasts. Units. NK, and B cells: cells/mm^3; IL-6: pg/mL; CRP: mg/L, D-dimer and ferritin: ng/L. Statistical details are in Section 6.2.40.

Figure S40

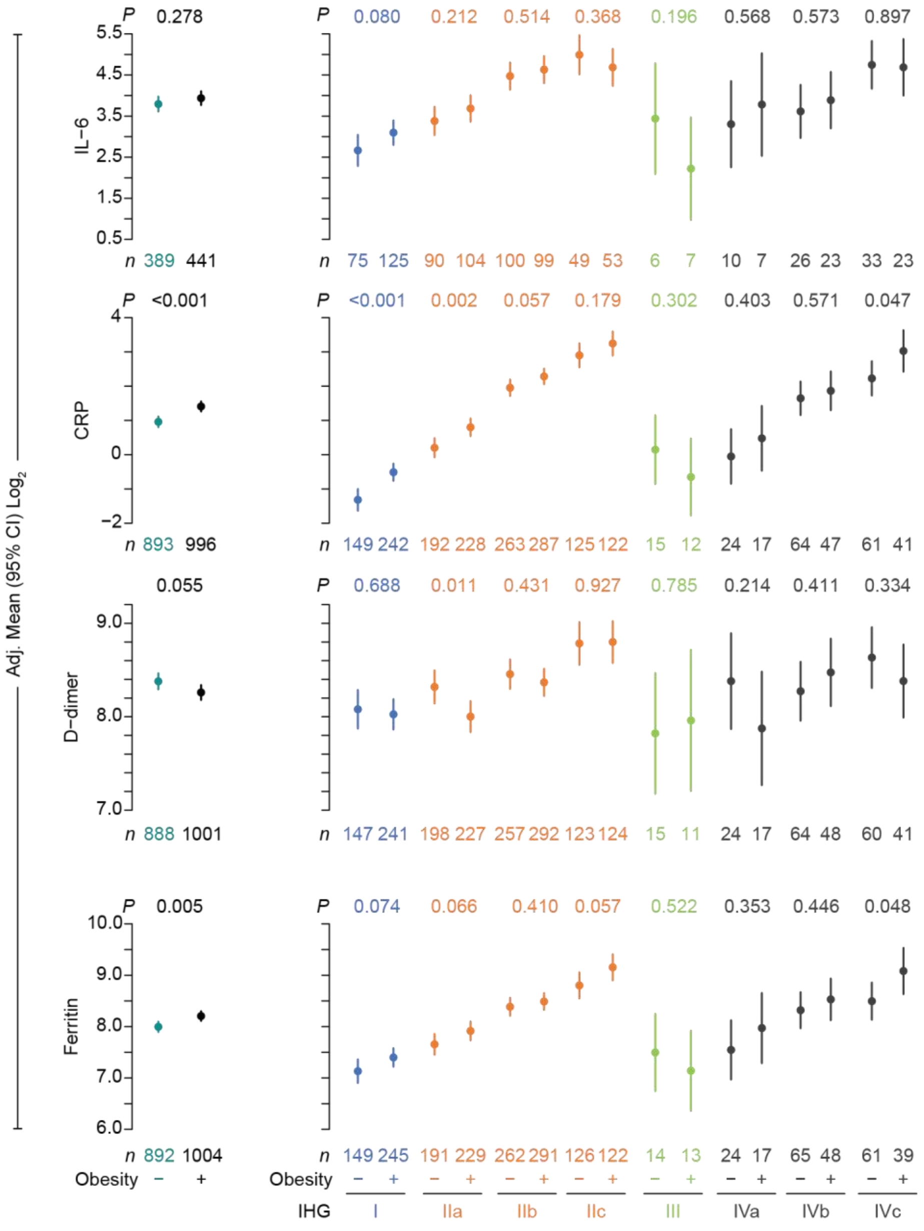

Figure S40 (continued)

3387 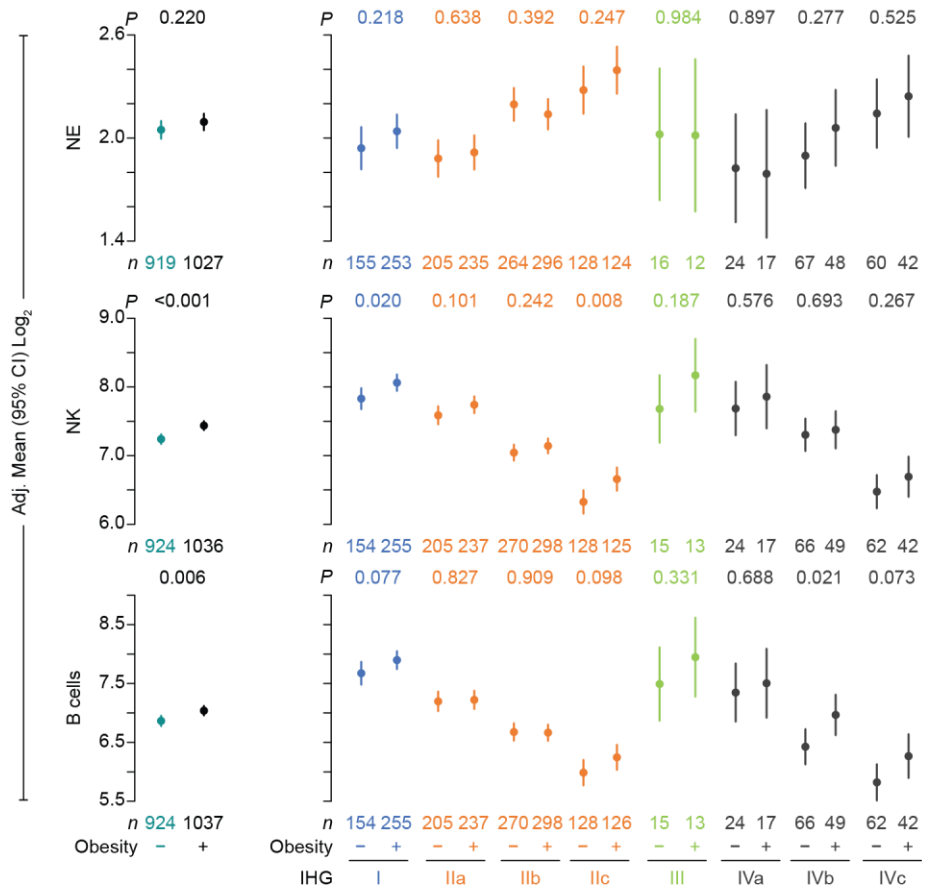

Figure S41. Association of baseline IHG status with acute COVID-19 mortality adjusting for biomarkers and comorbidity burden. (a) Kaplan-Meier plots depict time to acute (30-day) mortality in the VA-CLC by baseline IHG status with (*left*) comorbidity burden (0-1 vs. 3+) and (*right*) plasma IL-6 strata [high (H) and low (L) by median]. Rest, non-IHG-I grade. Note: Among persons with high comorbidity burden (3+), persons presenting with IHG-I (blue) vs. a non-IHG-I grade (black) have minimal mortality events (age-adjusted *P* (a*P*)=0.005). **(b)** Multivariate model of time to 30-day mortality with the indicated variables, depict the independent association of IHG-I controlling for comorbidity burden, and C-reactive protein (CRP) levels, sex, and age. Analysis performed using Cox proportional hazards model. Statistical details are in Section 6.2.41.

Figure S41

(a)

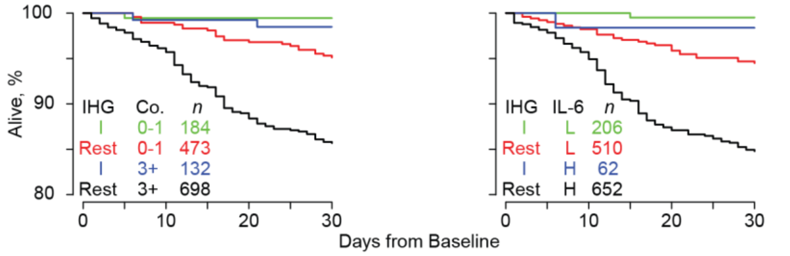

	Comorbidity burden (Co.) 0-1	Comorbidity burden (Co.) 3+	*aP*	Plasma IL-6 Low	Plasma IL-6 High	*aP*
**Rest**	473 (72%)	698 (84%)	0.013	510 (71%)	652 (91.3%)	<0.001
**IHG-I**	184 (28%)	132 (16%)	0.95	206 (29%)	62 (8.7%)	0.905
aP	0.095	0.005		0.070	0.027	

IR-preservers (IHG-I protective)

(b)

30-day all-cause mortality

	n	aHR	95% CI	*P*
**IHG-I**	300	1.00		
**Rest**	1126	3.72	1.15-12.04	0.028
**Comorbidity 0-1**	618	1.00		
**Comorbidity 3+**	808	1.85	1.16-2.95	0.010
**CRP Low**	727	1.00		
**CRP High**	699	3.44	2.13-5.55	<0.001
**F**	147	1.00		
**M**	1279	0.89	0.39-2.04	0.779
**Age, y**	1426	1.70	1.47-1.95	<0.001

Figure S42. Association of levels of signatures tracking the pathogenic triad, IHG and COVID-19 outcomes with immune and cytokine traits in the VA-CLC. After adjusting for age and multiple comparisons, of the 65 immune traits and 21 cytokines analyzed, we identified traits/cytokines that met three criteria: correlate with *TCF7* levels and associate with both IHG-I and better health outcomes (non-hospitalized, survivors). 16 immune traits/cytokines associated with IHG-I or outcomes; of these 10, correlated with both denoted by asterisks in the heatmap. Heatmap (left) depict the correlation of the traits/cytokines with the indicated gene signatures and (*right*) the mean levels of the traits/cytokines within optimal (IHG-I) and extreme IR-degrader (IHG-IIc/IHG-IVc) status, nonhospitalization (NH) status and nonsurvivor (died) status. Details of the signatures are in Figure 1d (main) and Table S1. Analysis performed using Pearson's correlation method with Euclidean distances and Ward's D linkage for heatmap on left. Details of the signatures are in Figure 1d (main) and Table S1. Higher levels of the IMM-AGE signature were computed to signify an association with fewer senescent T-cells (less immune aging and lower mortality; a {+}-salutogenesis readout), as detailed in Section 4.2. Statistical details in Section 6.2.42. Data correspond to Figure 7f left (main).

Figure S42 (corresponds to main Figure 7f, left)

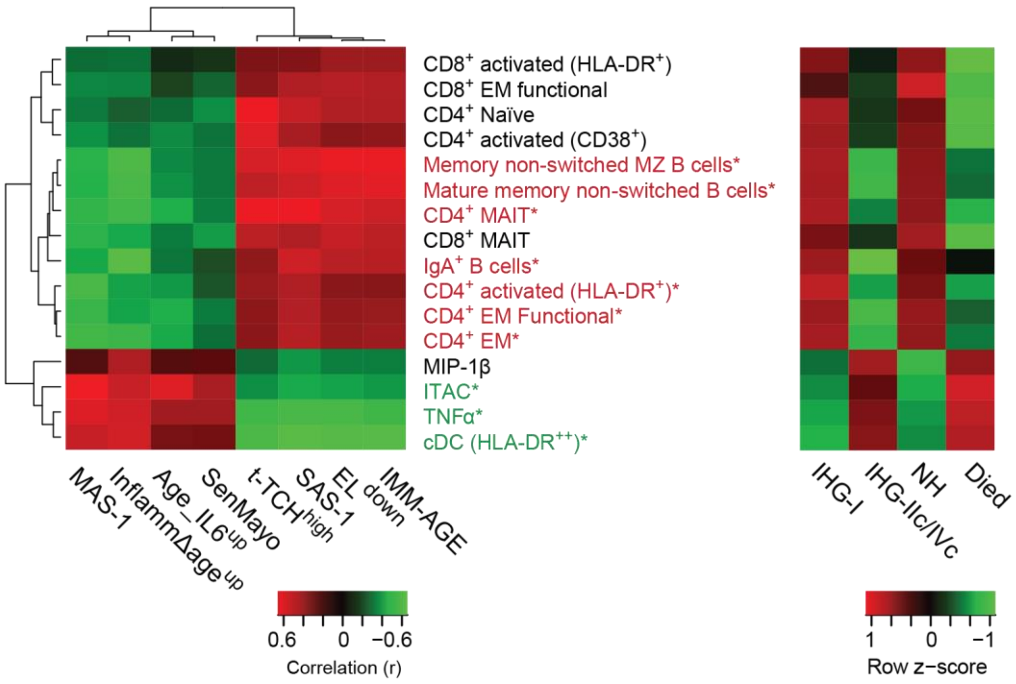

Figure S43. Association of IHG status, SAS-1/MAS-1 profiles, and acute COVID-19 outcomes with immune traits/cytokines. Levels of log_2-transformed immune traits/cytokines by baseline IHG status, SAS-1/MAS-1 profiles, and COVID-19 outcomes (NH: non hospitalized, HS: hospitalized survivals, NS: nonsurvival. Depicted are 16 immune traits/cytokines identified in Fig. S42. *P*, linear regression model with likelihood ratio test. SAS-1/MAS-1 profiles are (*left to right*): SAS-1^{high}-MAS-1^{low} (H-L; optimal IR status); SAS-1^{high}-MAS-1^{high} (H-H); SAS-1^{low}-MAS-1^{low} (L-L); and SAS-1^{low}-MAS-1^{high} (L-H; extreme-IR degrader status). IHG-III is infrequent and was not represented in the dataset. Statistical details are in Section 6.2.43.

Figure S43

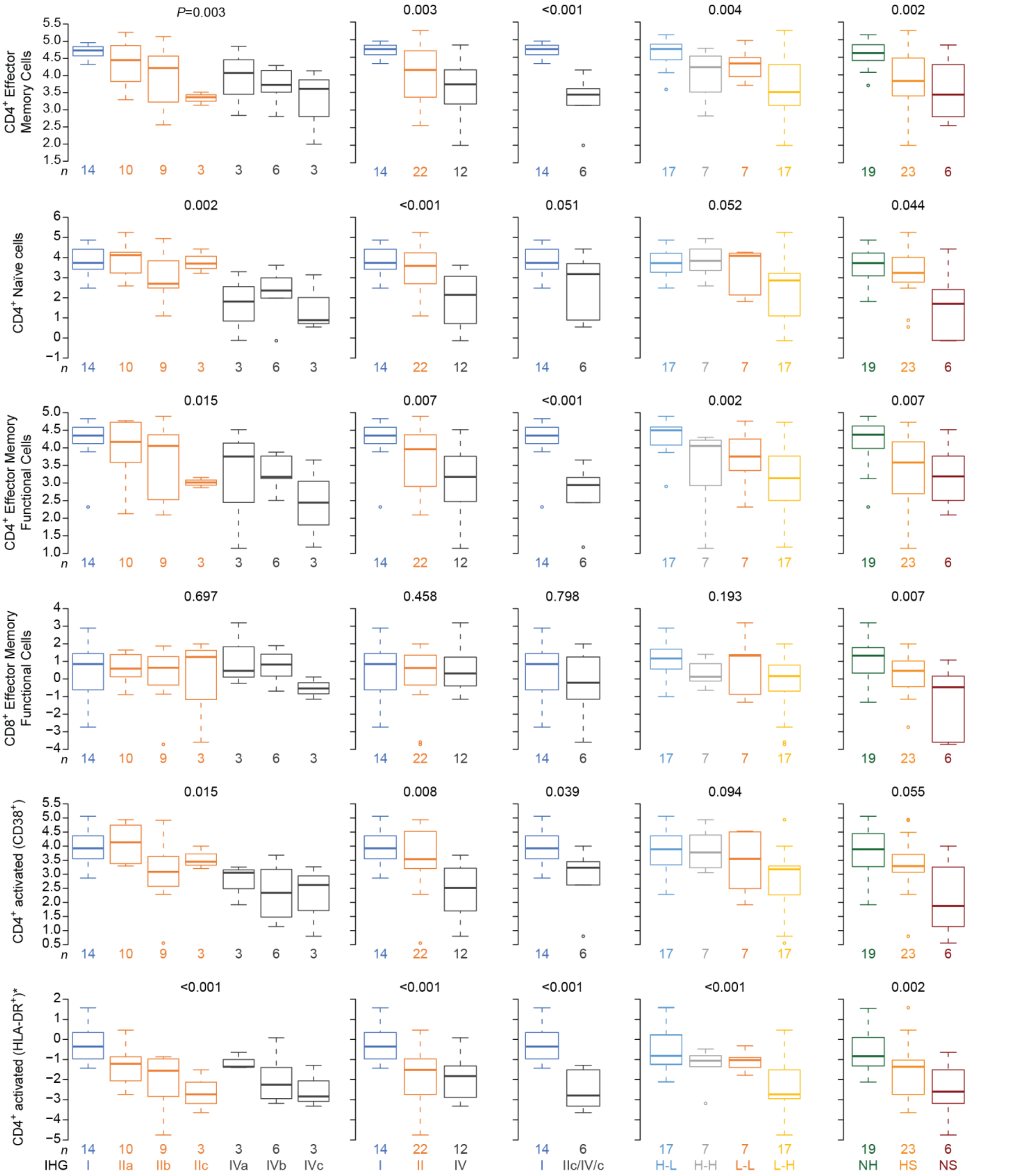

3429

Figure S43 (continued)

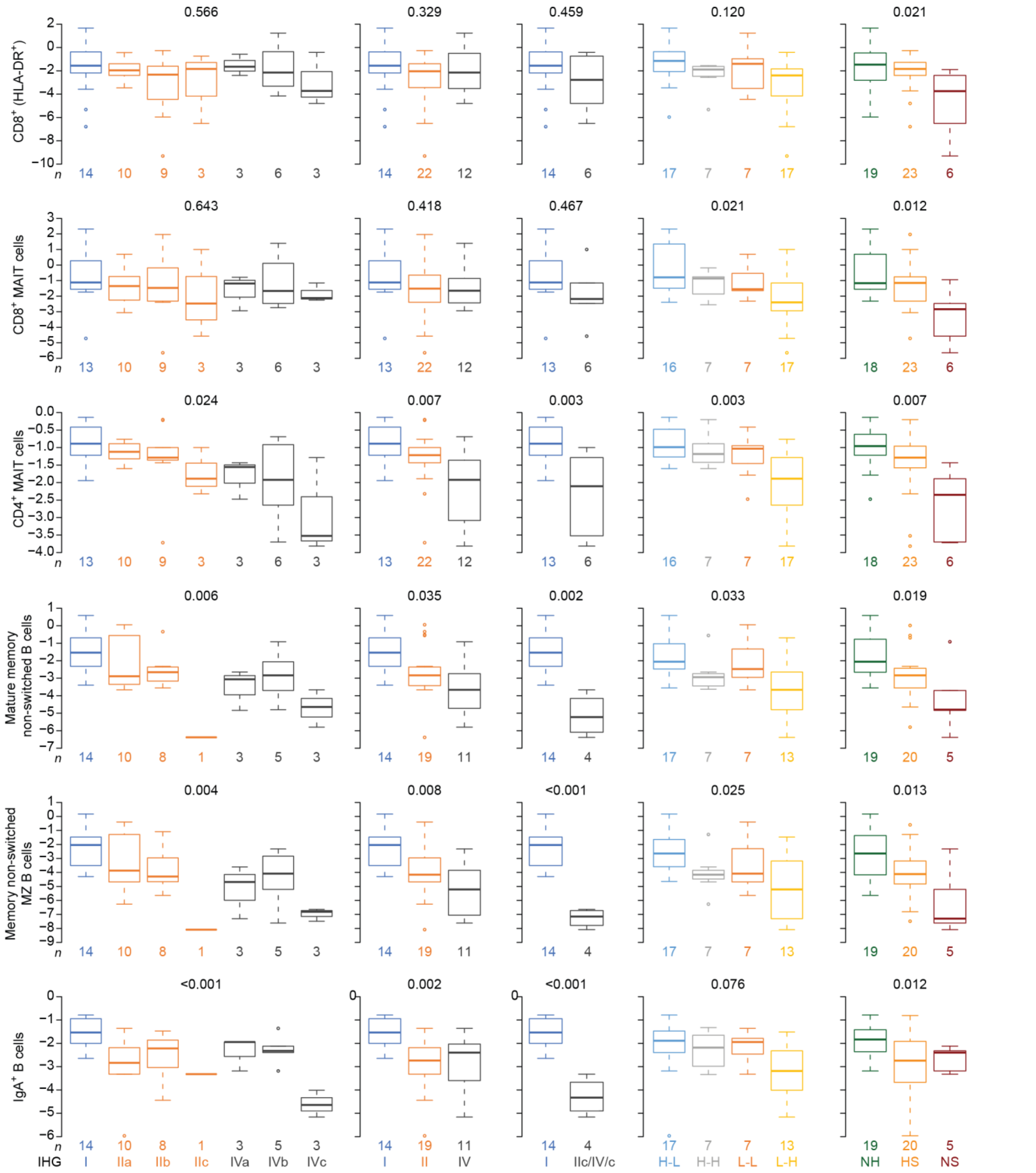

3430

Figure S43 (continued)

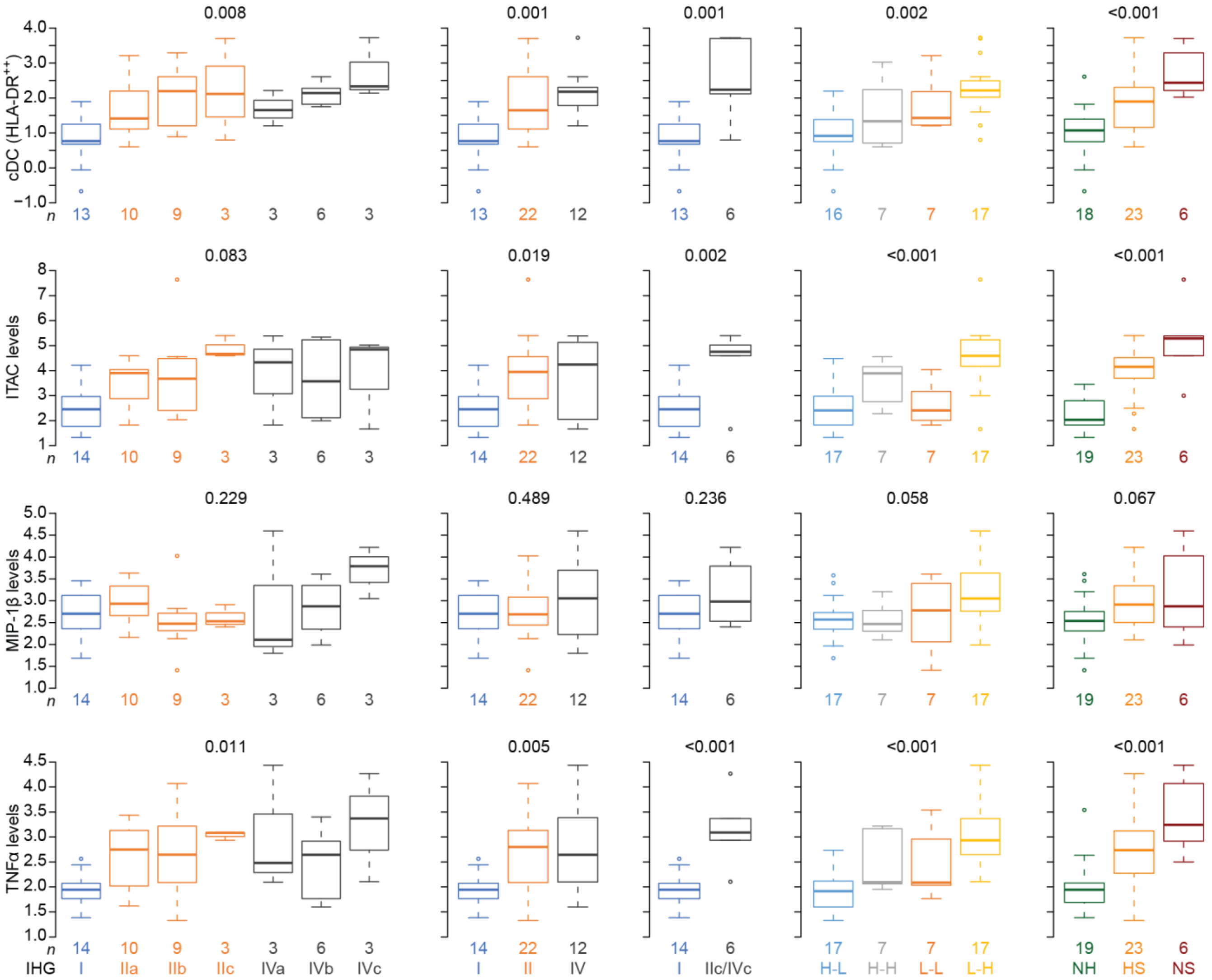

3431

Figure S44. Identification of transcription factors that associate with optimal IR (SAS-1^{high}-MAS-1^{low}) status in the Framingham Heart Study (FHS). (a) Stacked barplots depict distributions of SAS-1/MAS-1 profiles by octiles of expression levels (NGE) of indicated transcription factor genes. For the derivation of the transcription factors, see section 4.11. **(b)** Linear regression modeling the expression of indicated genes with 95% confidence bands (shaded area) over age stratified by sex in the FHS. **(c)** Age- and sex-adjusted hazard ratios of mortality for indicated transcription factors in the FHS. Analysis performed using Cox proportional hazard models. **(d)** Boxplots depict median (IQR) expression levels (NGE) of indicated transcription factor genes in the (*top*) VA-CLC by baseline IHG status and (*bottom*) NYU Medical Center COVID-19 cohort by survival status. NGE, normalized gene expression. Statistical details are in Section 6.2.44. Data correspond to Figure 7f upper right (main).

Figure S44 (corresponds to main Figure 7f upper right)

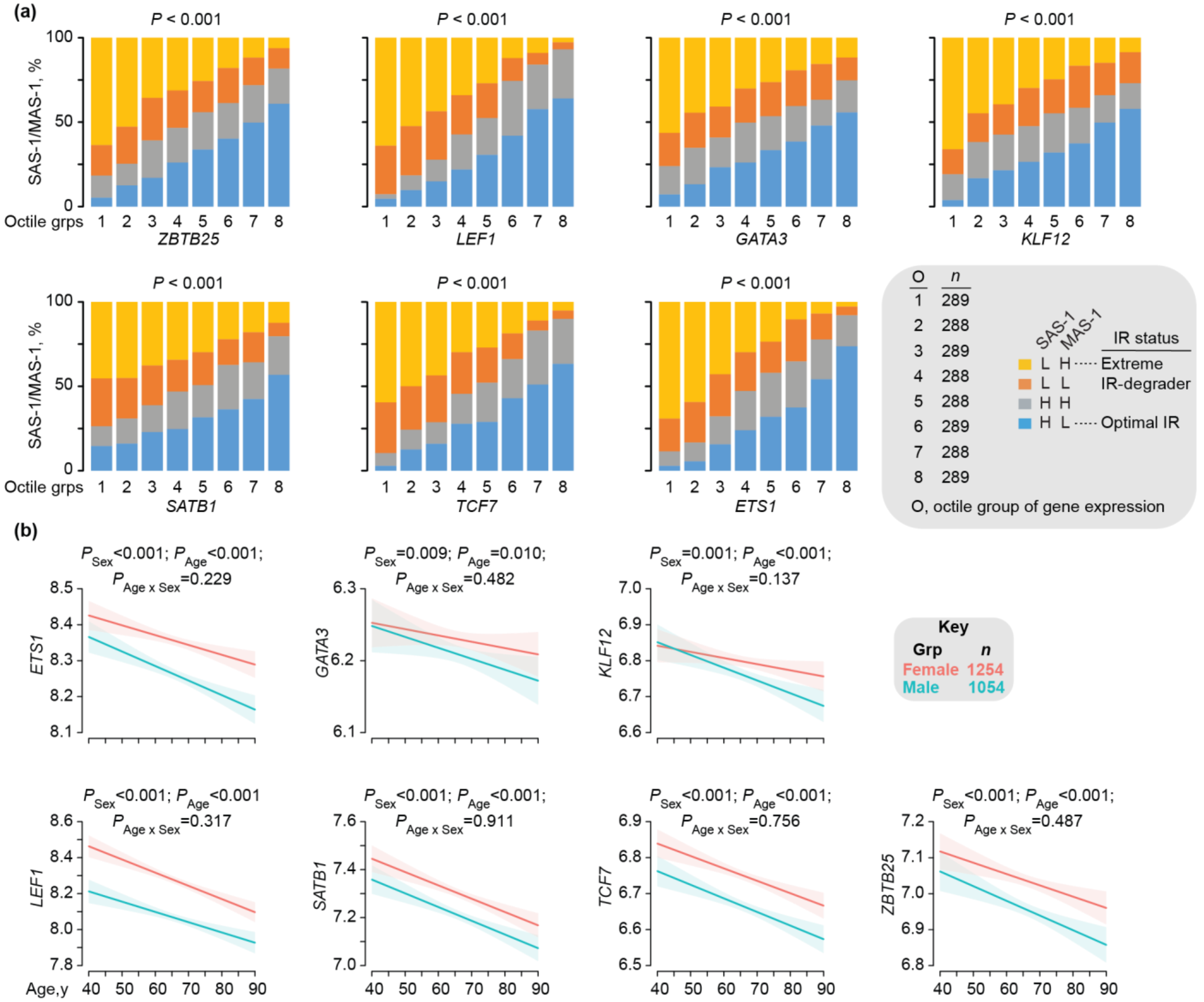

(c)

9-yr Survival in FHS

TF gene	aHR	FDR
ZBTB25	0.760	0.177
LEF1	0.723	0.060
GATA3	0.673	0.177
SATB1	0.625	0.016
TCF7	0.507	0.016
ETS1	0.492	0.012
KLF12	0.395	<0.001

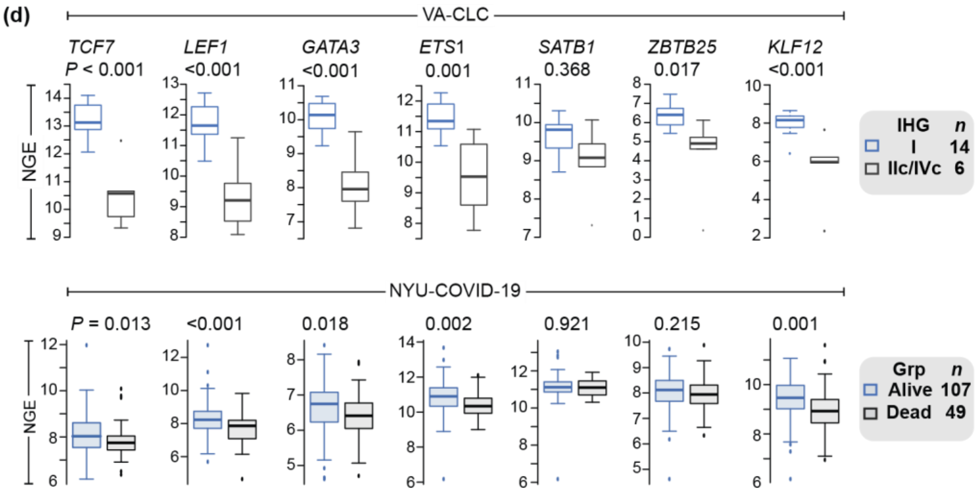

Figure S45. Association of immune resilience (IR) metrics, gene signatures tracking the pathogenic triad, and *TCF7* expression levels in persons with and without Alzheimer's disease (GSE140829). (a-b) Linear regression modeling of expression of indicated gene signatures (z-scores) and *TCF7* (NGE) with 95% confidence bands (shaded area) stratified by **(a)** disease status (AD, Alzheimer's disease) and **(b)** the indicated SAS-1/MAS-1 profiles [SAS-1^{high}-MAS-1^{low} (optimal IR status) vs. SAS-1^{low}-MAS-1^{high} (extreme-IR degrader status)] in persons with AD. *P*, linear model with likelihood ratio test (LRT). Details of the signatures are in Figure 1d (main) and Table S1. Details of the signatures are in Figure 1d (main) and Table S1. Higher levels of the IMM-AGE signature were computed to signify an association with fewer senescent T-cells (less immune aging and lower mortality; a {+}-salutogenesis readout), as detailed in Section 4.2. Statistical details are in Section 6.2.45. Data correspond to Figure 8c-d (main).

Figure S45a (corresponds to main Figure 8c)

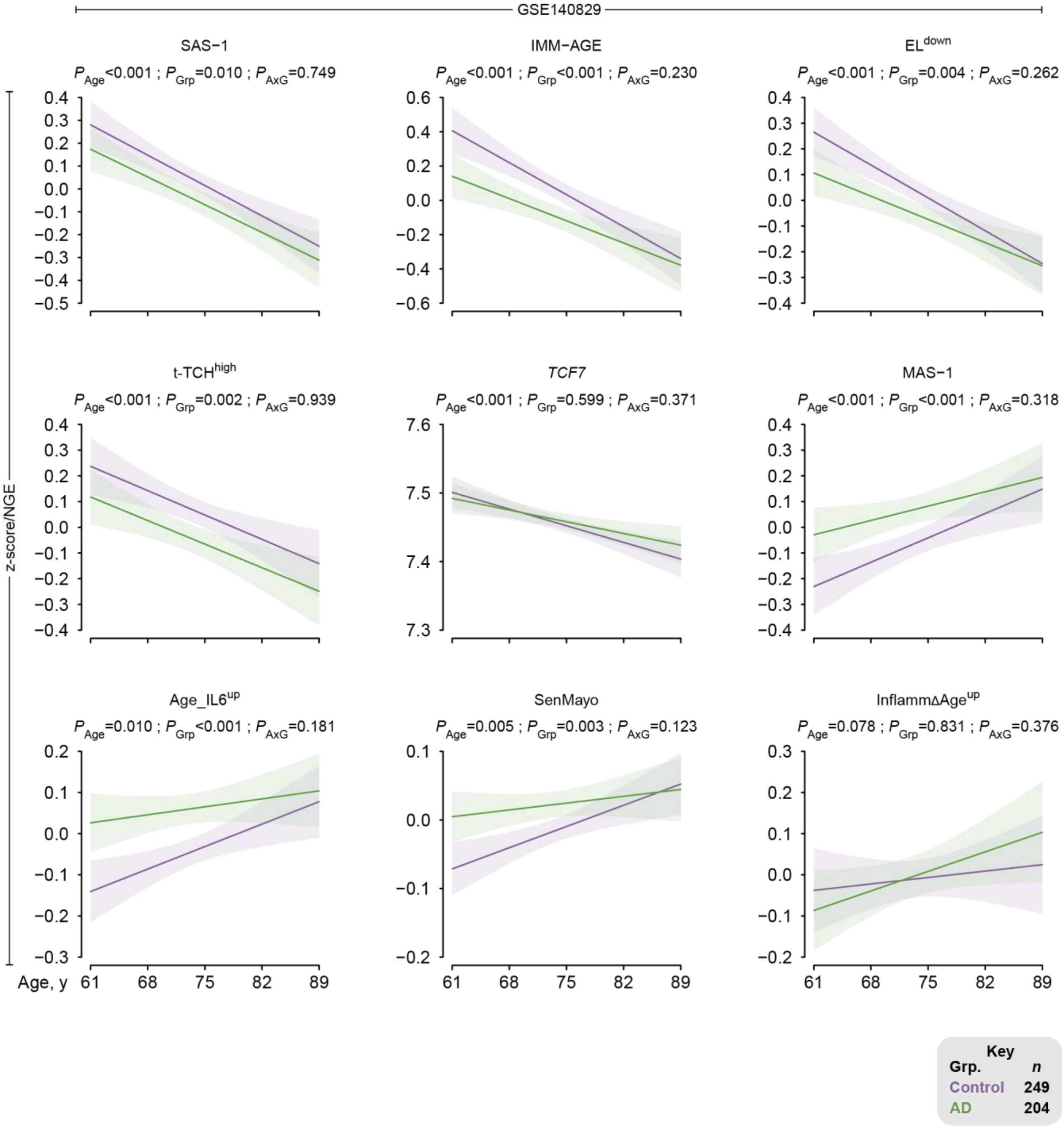

3460

Figure S45b (corresponds to main Figure 8d)

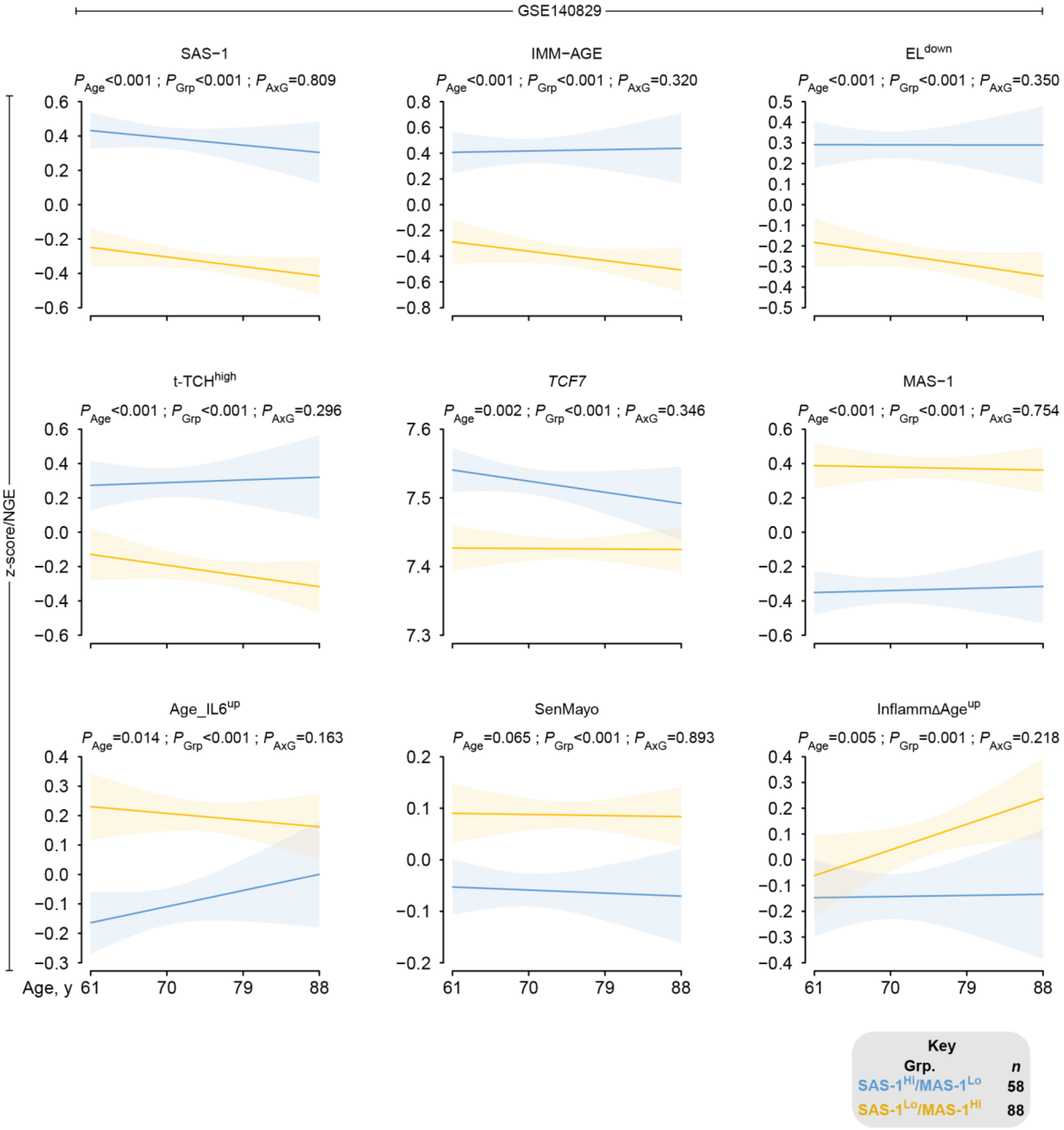

3461

Figure S46. Biomarker trajectories with IR preservation and degradation during COVID-19. Biomarker trajectories during 14 days from baseline in VA-CLC patients who remained IR-preservers (maintained IHG-I) vs. extreme IR-degraders who improved their IHG status (IHG-IIc/IVc to better IHG). Two *n*-values per group presented in panels correspond to number of biomarker measurements and number of unique persons. *P*, by linear GEE with interaction term and generalized linear hypothesis tests at Day 0 and Day 14. *, *P*<0.05; **, *P*<0.01; ***, *P*<0.001. Statistical details are in Section 6.2.46. Data correspond to Figure 8e (main).

Figure S46 (corresponds to main Figure 8e)

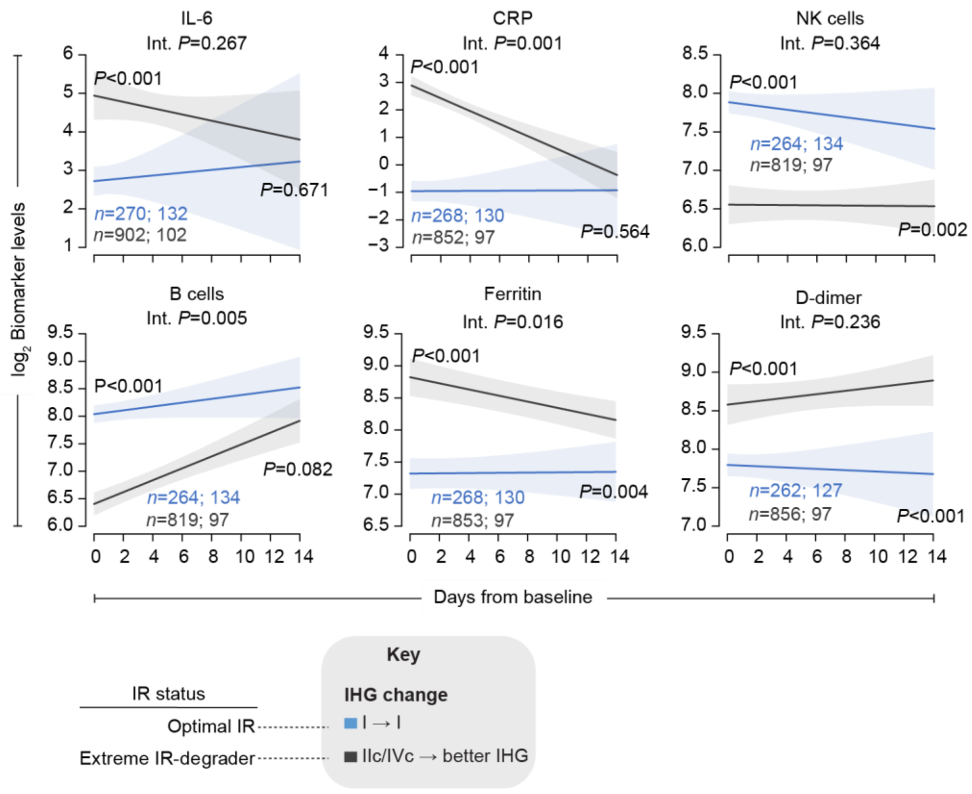

Figure S47. IR-associated plasma protein biomarkers in the MID cohort. Median (IQR) expression levels of indicated IR-associated proteomic biomarkers by SAS-1/MAS-1 profiles. *P*, by ANOVA. Biomarker derivation in Section 4.10.6 and statistical details are in Section 6.2.47. Data correspond to Figure 9b (main).

Figure S47 (corresponds to main Figure 9b)

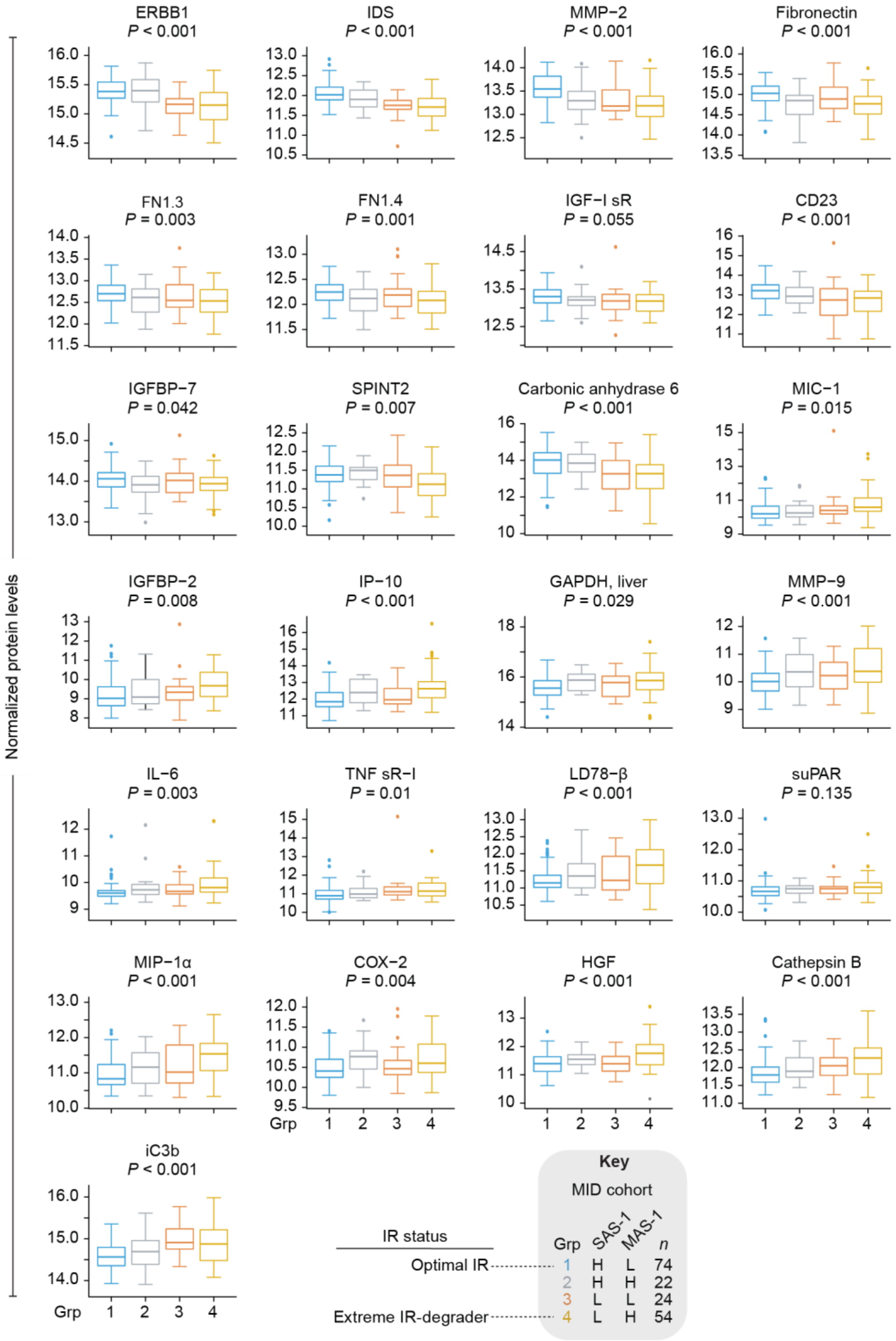

Figure S48. Association of immune resilience (IR) metrics, gene signatures tracking the pathogenic triad, and *TCF7* expression levels in persons with inflammatory bowel disease (IBD) receiving anti-inflammatory biologics (GSE191328). Line plots (mean ± SEM) depict the levels of the indicated gene signatures (z-scores) and *TCF7* levels (NGE) over time in persons with IBD stratified by baseline SAS-1/MAS-1 profiles: SAS-1^{high}-MAS-1^{low} (optimal IR status); SAS-1^{high}-MAS-1^{high}; SAS-1^{low}-MAS-1^{low}; and SAS-1^{low}-MAS-1^{high} (extreme-IR degrader status). Arrow, receipt of biologics. Details of the signatures are in Figure 1d (main) and Table S1. Details of the signatures are in Figure 1d (main) and Table S1. Higher levels of the IMM-AGE signature were computed to signify an association with fewer senescent T-cells (less immune aging and lower mortality; a {+}-salutogenesis readout), as detailed in Section 4.2. Statistical details are in Section 6.2.48. Data correspond to Figure 9c (main).

Figure S48 (corresponds to main Figure 9c)

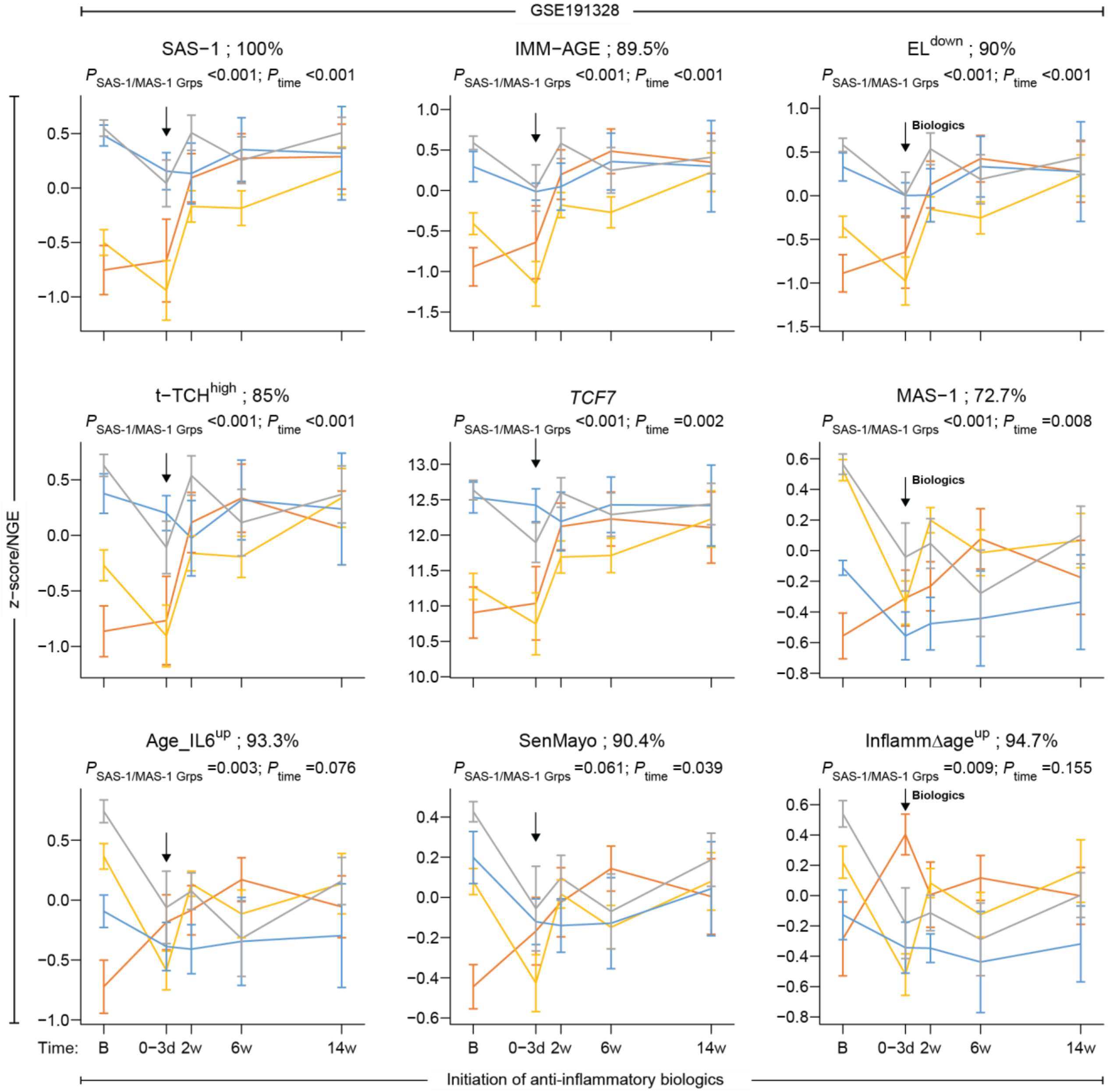

Key	*n*	Antagonism TNFα	Antagonism integrin $\alpha_4\beta_7$
SAS-1^{Hi}/MAS-1^{Lo}	5	2	3
SAS-1^{Hi}/MAS-1^{Hi}	16	10	6
SAS-1^{Lo}/MAS-1^{Lo}	12	8	4
SAS-1^{Lo}/MAS-1^{Hi}	20	16	4

B, baseline
d, days
w, weeks

Figure S49. Association of immune resilience (IR) metrics, gene signatures tracking the pathogenic triad, and *TCF7* expression levels in young adults receiving TNFα antagonist (etanercept) before challenge endotoxin (GSE36177). **(a)** Line plots (mean ± SEM) of indicated gene signatures (z-scores) and *TCF7* levels (NGE) before and 4 hours after intravenous challenge with endotoxin (LPS) in persons according to whether they received placebo or etanercept before LPS infusion. **(b)** Stacked barplots depic distribution of SAS-1/MAS-1 profiles and dot-and-line plots depict median (IQR) of *TCF7* (NGE) by the study groups at indicated timepoints. Details of the signatures are in Figure 1d (main) and Table S1. Higher levels of the IMM-AGE signature were computed to signify an association with fewer senescent T-cells (less immune aging and lower mortality; a {+}-salutogenesis readout), as detailed in Section 4.2. Statistical details are in Section 6.2.49.

Figure S49a

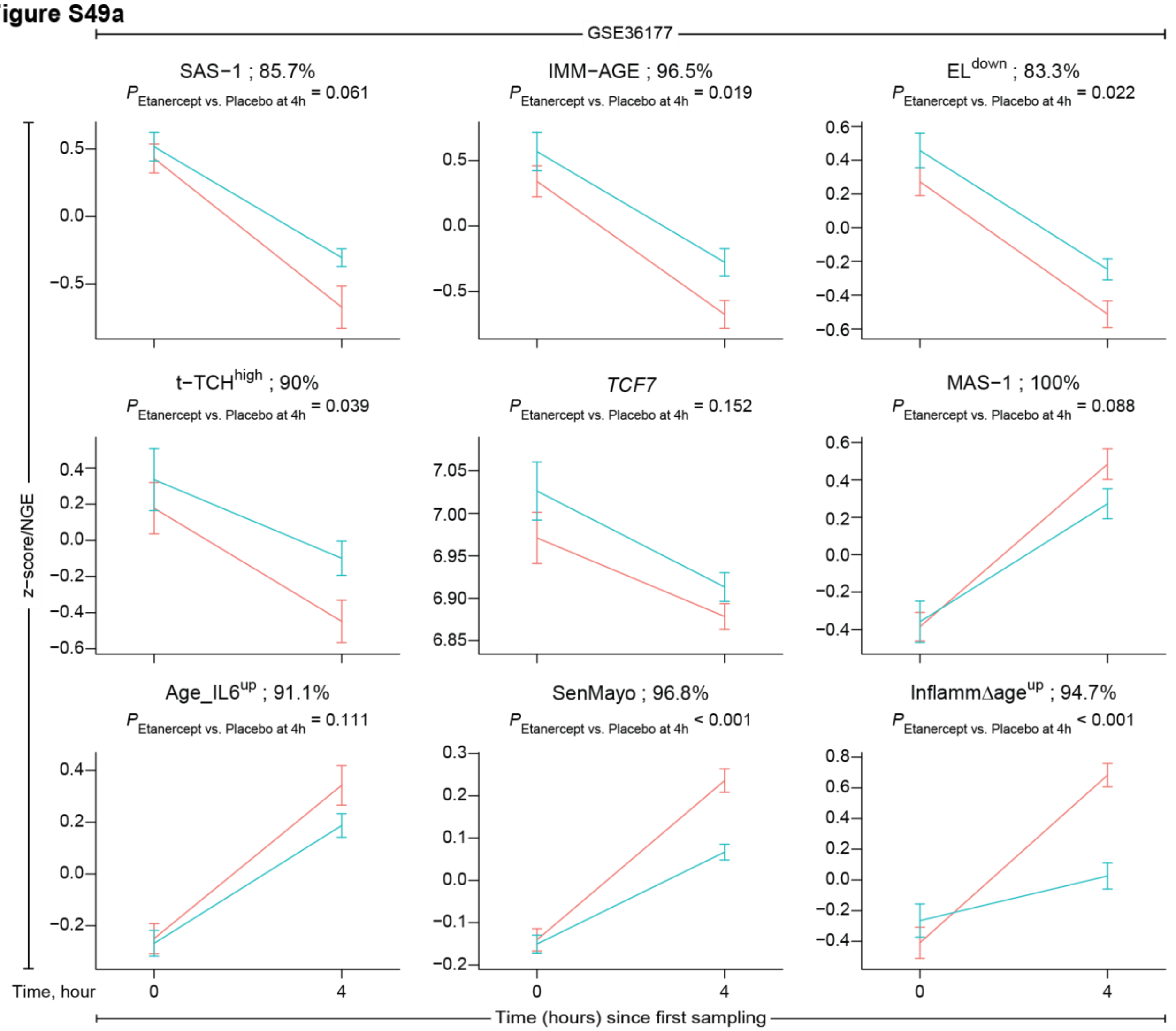

Figure S49b

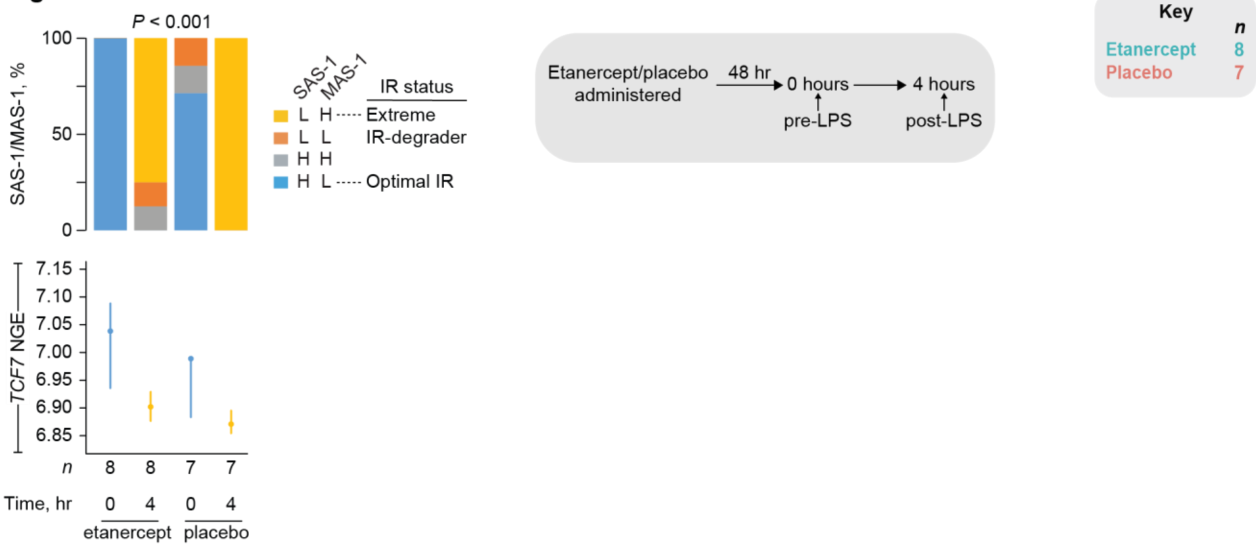

Figure S50. Evolutionarily conserved T-cell genes in the Framingham heart study (FHS). (a) Unsupervised hierarchical clustering heatmap of correlation of indicated T-cell genes with chronological age in the FHS. Color gradient of blue to red, low to high value of Pearson's correlation coefficient (r). **(b)** Correlation plots of indicated genes and age in the FHS are presented with color gradient and pie charts of the correlation values. **(c, d)** Linear regression modeling of the expression of indicated genes [normalized gene expression (NGE)] with 95% confidence bands across age by **(c)** sex and **(d)** SAS-1/MAS-1 profiles. **(e)** Forest plots depict age- and sex-adjusted hazard ratios of mortality of the indicated genes. Statistical details are in Section 6.2.50. Data correspond to Figure 9e (main).

Figure S50 (corresponds to main Figure 9e)

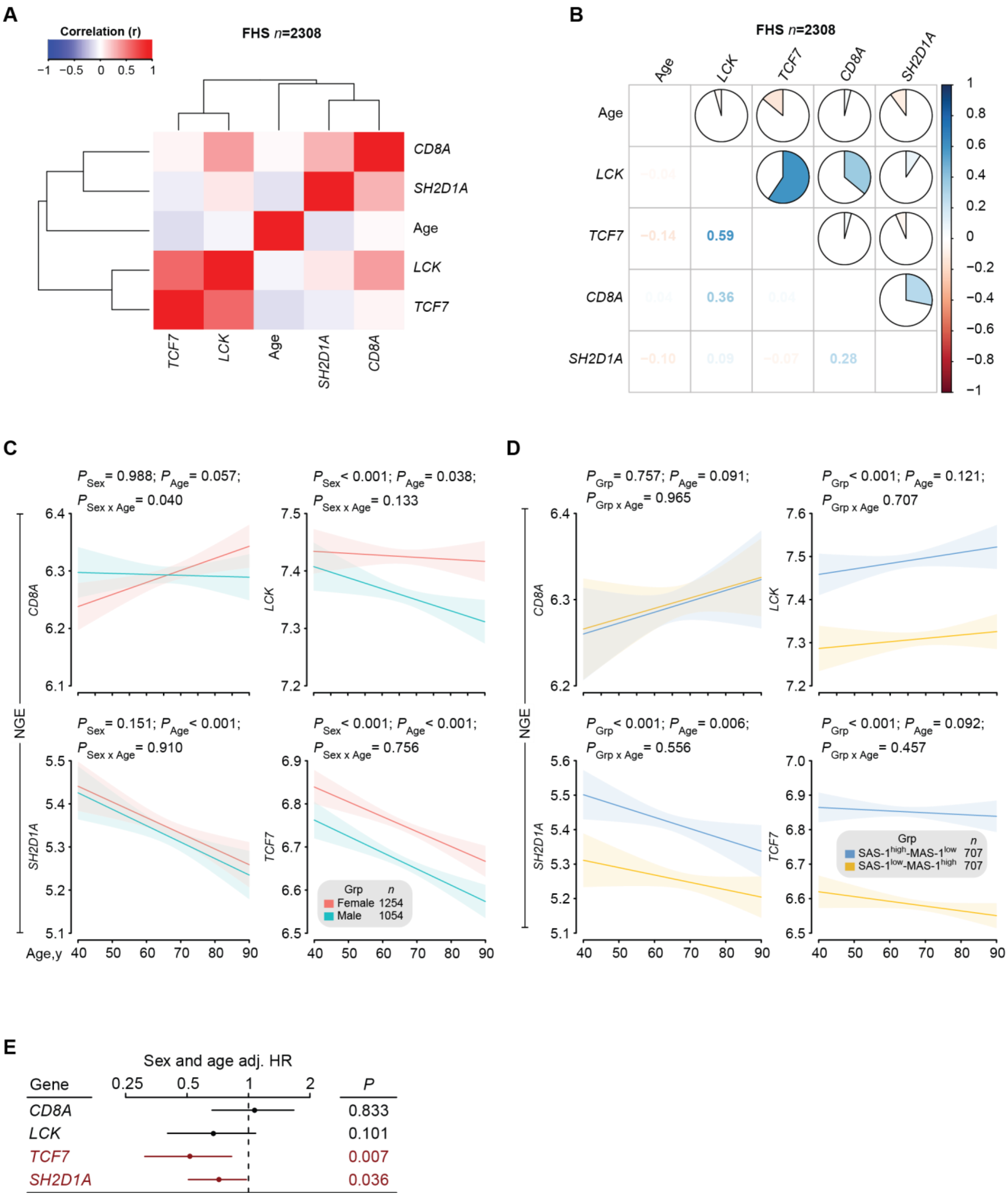

9. Supplementary Tables

Supplementary tables are provided in Microsoft Excel format (.xlsx).

Table S1. Gene signatures for gauging components of pathogenic triad and immune resilience levels

Table S2. Gene expression signature gene **(a)** lists and **(b)** membership

Table S3. Hazard ratios of mortality by SAS-1/MAS-1 profiles within age strata in the Framingham Heart Study (FHS).

Table S4. Models accompanying Figure 5

- **a.** Hazard ratios of mortality by Age_IL6^{up} strata and baseline CVD status in the Framingham Heart Study (FHS). (corresponding to Figure 5g)
- **b.** Hazard ratios of incident CVD diagnosis by SAS-1/MAS-1 profiles and a proxy for inflammaging (Age_IL6^{up} strata) in the Framingham Heart Study (FHS). (corresponding to Fig. 5i)
- **c.** Hazard ratios of mortality by SAS-1/MAS-1 profiles and proxy for inflammaging (Age_IL6^{up} strata) in the Framingham Heart Study (FHS). (corresponding to Fig. 5j)

Table S5. Characteristics of VA-CLC subsets:

- **a.** Characteristics of VA-CLC acute-COVID-19 cohort.
- **b.** Characteristics of the post-acute COVID-19 component of the VA-CLC.
- **c.** Biomarker measurements evaluated in acute and post-acute COVID-19 subsets of VA-CLC.
- **d.** Characteristics of VA-CLC subset with measurement at pre-acute, during ac-COVID-19 (baseline), and post-acute (convalescence) phases of COVID-19.
- **e.** Characteristics of VA-CLC ac-COVID-19 subset with ICD-10-CM U09.9 codes after 30 days of initial presentation.
- **f.** Characteristics of the VA-CLC ac-COVID-19 subset used for mechanistic studies by hospitalization and survival status.

- **g.** Characteristics of the VA-CLC ac-COVID-19 subset used for mechanistic studies by IHG status.
- **h.** Characteristics of ac-COVID-19 outbreak subset of the VA-CLC.

Table S6. Characteristics of SAIL cohort subsets: **(a)** overall and **(b)** pre-booster vaccine subset.

Table S7. Characteristics of New York University Medical Center acute COVID-19 cohort.

Table S8. Associations of COVID-19 outcomes, biomarkers, and IHG.

- **a.** Associations of indicated outcomes with IHG, biomarkers, age, and sex in the ac-COVID-19 component of the VA-CLC.
- **b.** Associations of 30-day discharge with IHG, biomarkers, sex, and age in the ac-COVID-19 subset of the VA-CLC.
- **c.** Associations of post-acute mortality with median groups of biomarkers, IHG, and age in the post-acute component of the VA-CLC.

Table S9. Associations of indicated outcomes with **(a)** IHG, vaccination status, and age, **(b)** difference in era in the VA-CLC, **(c)** viral load, and **(d)** CMV serostatus with IHG and age.

Table S10. Associations of acute and post-acute mortality according to IR improvement within 5-day windows of presentation, controlling for biomarkers, number of measurements, and age in the VA-CLC.

Table S11. Associations of %inhibition (<30% and <50%) by IHG, age, sex, vaccination, and previous SARS-CoV-2 infection in the SAIL pre-booster vaccine subset.

Table S12. Associations of comorbidities with mortality in VA-CLC subsets

- **a.** Associations of 30-day mortality with comorbidities (*left*), and comorbidities, IHG, and age (*right*) in the ac-COVID-19 component of the VA-CLC.

b. Associations of post-acute mortality with comorbidities (*left*), comorbidities, IHG, and age (*right*) in the post-acute component of the VA-CLC.

Table S13. Biomarkers and Immune traits

a. Correlations of immune traits, cytokines, and gene signatures with *TCF7* in the VA-CLC.

b. Correlations of *TCF7*-associated immune traits with IHG and COVID-19 outcomes in the VA-CLC.

c. Biomarker correlations with *TCF7* in acute COVID-19.

Table S14. Proteomic biomarkers

a. Linear modeling of IR-associated biomarkers in the MID cohort (corresponding to Figure 9a)

b. Full set of linear modeling of MID cohort proteomics by SAS-1 and MAS-1 gene expression signatures

Table S15. Cohorts and datasets

a. Cohorts used in the current study by figure panels.

b. Publicly available datasets used in the current study by figure panels.

10. References

Ahuja, S. K., Manoharan, M. S., Lee, G. C., McKinnon, L. R., Meunier, J. A., Steri, M., . . . He, W. (2023). Immune resilience despite inflammatory stress promotes longevity and favorable health outcomes including resistance to infection. *Nat Commun, 14*(1), 3286. doi:10.1038/s41467-023-38238-6

Alpert, A., Pickman, Y., Leipold, M., Rosenberg-Hasson, Y., Ji, X., Gaujoux, R., . . . Shen-Orr, S. S. (2019). A clinically meaningful metric of immune age derived from high-dimensional longitudinal monitoring. *Nat Med, 25*(3), 487-495. doi:10.1038/s41591-019-0381-y

Anders, S., & Huber, W. (2010). Differential expression analysis for sequence count data. *Genome Biol, 11*(10), R106. doi:10.1186/gb-2010-11-10-r106

Armstrong, J. S. (2007). Significance tests harm progress in forecasting. *Int. J. Forecast., 23*, 321-327.

Arunachalam, P. S., Scott, M. K. D., Hagan, T., Li, C., Feng, Y., Wimmers, F., . . . Pulendran, B. (2021). Systems vaccinology of the BNT162b2 mRNA vaccine in humans. *Nature, 596*(7872), 410-416. doi:10.1038/s41586-021-03791-x

Arunachalam, P. S., Wimmers, F., Mok, C. K. P., Perera, R., Scott, M., Hagan, T., . . . Pulendran, B. (2020). Systems biological assessment of immunity to mild versus severe COVID-19 infection in humans. *Science, 369*(6508), 1210-1220. doi:10.1126/science.abc6261

Bacchetti, P. (2002). Peer review of statistics in medical research: the other problem. *BMJ, 324*(7348), 1271-1273. doi:10.1136/bmj.324.7348.1271

Banchereau, R., Hong, S., Cantarel, B., Baldwin, N., Baisch, J., Edens, M., . . . Pascual, V. (2016). Personalized Immunomonitoring Uncovers Molecular Networks that Stratify Lupus Patients. *Cell, 165*(3), 551-565. doi:10.1016/j.cell.2016.03.008

Bandewar, S. V., Kimani, J., & Lavery, J. V. (2010). The origins of a research community in the Majengo Observational Cohort Study, Nairobi, Kenya. *BMC Public Health, 10*, 630. doi:10.1186/1471-2458-10-630

Butler, A., Hoffman, P., Smibert, P., Papalexi, E., & Satija, R. (2018). Integrating single-cell transcriptomic data across different conditions, technologies, and species. *Nat Biotechnol, 36*(5), 411-420. doi:10.1038/nbt.4096

Camargo, J. F., Quinones, M. P., Mummidi, S., Srinivas, S., Gaitan, A. A., Begum, K., . . . Ahuja, S. K. (2009). CCR5 expression levels influence NFAT translocation, IL-2 production, and subsequent signaling events during T lymphocyte activation. *J Immunol, 182*(1), 171-182. Retrieved from http://www.ncbi.nlm.nih.gov/entrez/query.fcgi?cmd=Retrieve&db=PubMed&dopt=Citation&list_uids=19109148

Chundru, V. K., Marioni, R. E., Prendergast, J. G. D., Lin, T., Beveridge, A. J., Martin, N. G., . . . McRae, A. F. (2023). Rare genetic variants underlie outlying levels of DNA methylation and gene-expression. *Hum Mol Genet, 32*(11), 1912-1921. doi:10.1093/hmg/ddad028

Cohen, J. (1994). The Earth is Round (p < .05). *American Psychologist, 49*, 997-1003.

Dawber, T. R., Meadors, G. F., & Moore, F. E., Jr. (1951). Epidemiological approaches to heart disease: the Framingham Study. *Am J Public Health Nations Health, 41*(3), 279-281. doi:10.2105/ajph.41.3.279

De Zuani, M., Laznickova, P., Tomaskova, V., Dvoncova, M., Forte, G., Stokin, G. B., . . . Fric, J. (2022). High CD4-to-CD8 ratio identifies an at-risk population susceptible to lethal COVID-19. *Scand J Immunol, 95*(3), e13125. doi:10.1111/sji.13125

Force, A. D. T., Ranieri, V. M., Rubenfeld, G. D., Thompson, B. T., Ferguson, N. D., Caldwell, E., . . . Slutsky, A. S. (2012). Acute respiratory distress syndrome: the Berlin Definition. *JAMA, 307*(23), 2526-2533. doi:10.1001/jama.2012.5669

Gardner, M. J., & Altman, D. G. (1986). Confidence intervals rather than P values: estimation rather than hypothesis testing. *Br Med J (Clin Res Ed), 292*(6522), 746-750. doi:10.1136/bmj.292.6522.746

Gigerenzer, G. (2004). Mindless statistics. *Journal of Socio-Economics, 33*, 587-606.

Goebeler, S., Jylha, M., & Hervonen, A. (2003). Medical history, cognitive status and mobility at the age of 90. A population-based study in Tampere, Finland. *Aging Clin Exp Res, 15*(2), 154-161. doi:10.1007/BF03324494

Goodman, S. N. (1999). Toward evidence-based medical statistics. 1: The P value fallacy. *Ann Intern Med, 130*(12), 995-1004. doi:10.7326/0003-4819-130-12-199906150-00008

Harris, P. A., Taylor, R., Minor, B. L., Elliott, V., Fernandez, M., O'Neal, L., . . . Consortium, R. E. (2019). The REDCap consortium: Building an international community of software platform partners. *J Biomed Inform, 95*, 103208. doi:10.1016/j.jbi.2019.103208

Harris, P. A., Taylor, R., Thielke, R., Payne, J., Gonzalez, N., & Conde, J. G. (2009). Research electronic data capture (REDCap)--a metadata-driven methodology and workflow process for providing translational research informatics support. *J Biomed Inform, 42*(2), 377-381. doi:10.1016/j.jbi.2008.08.010

Huang da, W., Sherman, B. T., & Lempicki, R. A. (2009). Systematic and integrative analysis of large gene lists using DAVID bioinformatics resources. *Nat Protoc, 4*(1), 44-57. doi:10.1038/nprot.2008.211

INSIGHT START Study Group, Lundgren, J. D., Babiker, A. G., Gordin, F., Emery, S., Grund, B., . . . Neaton, J. D. (2015). Initiation of Antiretroviral Therapy in Early Asymptomatic HIV Infection. *N Engl J Med, 373*(9), 795-807. doi:10.1056/NEJMoa1506816

Jeewandara, C., Jayathilaka, D., Gomes, L., Wijewickrama, A., Narangoda, E., Idampitiya, D., . . . Malavige, G. N. (2021). SARS-CoV-2 neutralizing antibodies in patients with varying severity of acute COVID-19 illness. *Sci Rep, 11*(1), 2062. doi:10.1038/s41598-021-81629-2

Jylhava, J., Raitanen, J., Marttila, S., Hervonen, A., Jylha, M., & Hurme, M. (2014). Identification of a prognostic signature for old-age mortality by integrating genome-wide transcriptomic data with the conventional predictors: the Vitality 90+ Study. *BMC Med Genomics, 7*, 54. doi:10.1186/1755-8794-7-54

Kannel, W. B., Feinleib, M., McNamara, P. M., Garrison, R. J., & Castelli, W. P. (1979). An investigation of coronary heart disease in families. The Framingham offspring study. *Am J Epidemiol, 110*(3), 281-290. doi:10.1093/oxfordjournals.aje.a112813

Karagiannis, T. T., Dowrey, T. W., Villacorta-Martin, C., Montano, M., Reed, E., Belkina, A. C., . . . Sebastiani, P. (2023). Multi-modal profiling of peripheral blood cells across the human lifespan reveals distinct immune cell signatures of aging and longevity. *EBioMedicine, 90*, 104514. doi:10.1016/j.ebiom.2023.104514

Kennedy, R. B., Ovsyannikova, I. G., Haralambieva, I. H., Oberg, A. L., Zimmermann, M. T., Grill, D. E., & Poland, G. A. (2016). Immunosenescence-Related Transcriptomic and Immunologic Changes in Older Individuals Following Influenza Vaccination. *Front Immunol, 7*, 450. doi:10.3389/fimmu.2016.00450

Krantz, E. M., Hullsiek, K. H., Okulicz, J. F., Weintrob, A. C., Agan, B. K., Crum-Cianflone, N. F., . . . Hale, B. R. (2011). Elevated CD8 counts during HAART are associated with HIV virologic treatment failure. *Journal of acquired immune deficiency syndromes, 57*(5), 396-403. doi:10.1097/QAI.0b013e318221c62a

Lambert, K., Moo, K. G., Arnett, A., Goel, G., Hu, A., Flynn, K. J., . . . Khor, B. (2022). Deep immune phenotyping reveals similarities between aging, Down syndrome, and autoimmunity. *Sci Transl Med, 14*(627), eabi4888. doi:10.1126/scitranslmed.abi4888

Lambert, S. A., Jolma, A., Campitelli, L. F., Das, P. K., Yin, Y., Albu, M., . . . Weirauch, M. T. (2018). The Human Transcription Factors. *Cell, 172*(4), 650-665. doi:10.1016/j.cell.2018.01.029

Le, T., Wright, E. J., Smith, D. M., He, W., Catano, G., Okulicz, J. F., . . . Ahuja, S. K. (2013). Enhanced CD4+ T-cell recovery with earlier HIV-1 antiretroviral therapy. *The New England journal of medicine, 368*(3), 218-230. doi:10.1056/NEJMoa1110187

Lecoutre, B., Lecoutre, M.-P., & Poitevineau, J. (2001). Uses, Abuses and Misuses of Significance Tests in the Scientific Community: Won't the Bayesian Choice be Unavoidable? *International Statistical Review, 69*(3), 399-417. doi:https://doi.org/10.1111/j.1751-5823.2001.tb00466.x

Lee, G. C., Restrepo, M. I., Harper, N., Manoharan, M. S., Smith, A. M., Meunier, J. A., . . . Ahuja, S. K. (2021). Immunologic resilience and COVID-19 survival advantage. *J Allergy Clin Immunol, 148*(5), 1176-1191. doi:10.1016/j.jaci.2021.08.021

Li, S., Rouphael, N., Duraisingham, S., Romero-Steiner, S., Presnell, S., Davis, C., . . . Pulendran, B. (2014). Molecular signatures of antibody responses derived from a systems biology study of five human vaccines. *Nat Immunol, 15*(2), 195-204. doi:10.1038/ni.2789

Lin, H., Lunetta, K. L., Zhao, Q., Rong, J., Benjamin, E. J., Mendelson, M. M., . . . Murabito, J. M. (2017). Transcriptome-wide association study of inflammatory biologic age. *Aging (Albany NY), 9*(11), 2288-2301. doi:10.18632/aging.101321

Maciejak, A., Kiliszek, M., Michalak, M., Tulacz, D., Opolski, G., Matlak, K., . . . Burzynska, B. (2015). Gene expression profiling reveals potential prognostic biomarkers associated with the progression of heart failure. *Genome Med, 7*(1), 26. doi:10.1186/s13073-015-0149-z

Marconi, V. C., Grandits, G., Okulicz, J. F., Wortmann, G., Ganesan, A., Crum-Cianflone, N., . . . Kulkarni, H. (2011). Cumulative viral load and virologic decay patterns after antiretroviral therapy in HIV-infected subjects influence CD4 recovery and AIDS. *PLoS One, 6*(5), e17956. doi:10.1371/journal.pone.0017956

Marconi, V. C., Grandits, G. A., Weintrob, A. C., Chun, H., Landrum, M. L., Ganesan, A., . . . Agan, B. K. (2010). Outcomes of highly active antiretroviral therapy in the context of universal access to healthcare: the U.S. Military HIV Natural History Study. *AIDS research and therapy, 7*, 14. doi:10.1186/1742-6405-7-14

McClain, M. T., Constantine, F. J., Henao, R., Liu, Y., Tsalik, E. L., Burke, T. W., . . . Woods, C. W. (2021). Dysregulated transcriptional responses to SARS-CoV-2 in the periphery. *Nat Commun, 12*(1), 1079. doi:10.1038/s41467-021-21289-y

Nachun, D., Ramos, E., Karydas, A., Dokuru, D., Gao, F., Yang, Z., . . . Coppola, G. (2019). Systems-level analysis of peripheral blood gene expression in dementia patients reveals an innate immune response shared across multiple disorders. *bioRxiv*, 2019.2012.2013.875112. doi:10.1101/2019.12.13.875112

Okulicz, J. F., Le, T. D., Agan, B. K., Camargo, J. F., Landrum, M. L., Wright, E., . . . Ahuja, S. K. (2015). Influence of the timing of antiretroviral therapy on the potential for normalization of immune status in human immunodeficiency virus 1-infected individuals. *JAMA Intern Med, 175*(1), 88-99. doi:10.1001/jamainternmed.2014.4010

Okulicz, J. F., Marconi, V. C., Landrum, M. L., Wegner, S., Weintrob, A., Ganesan, A., . . . Dolan, M. J. (2009). Clinical outcomes of elite controllers, viremic controllers, and long-term nonprogressors in the US Department of Defense HIV natural history study. *The Journal of infectious diseases, 200*(11), 1714-1723. doi:10.1086/646609

Orru, V., Steri, M., Sole, G., Sidore, C., Virdis, F., Dei, M., . . . Cucca, F. (2013). Genetic variants regulating immune cell levels in health and disease. *Cell, 155*(1), 242-256. doi:10.1016/j.cell.2013.08.041

Pace, M., Filomena, A., Galli, A., & Manetti, R. (1989). [Surgical treatment of cutaneous melanoma]. *Ann Ital Chir, 60*(4), 247-255. Retrieved from https://www.ncbi.nlm.nih.gov/pubmed/2699709

Paczkowska-Abdulsalam, M., Niemira, M., Bielska, A., Szalkowska, A., Raczkowska, B. A., Junttila, S., . . . Kretowski, A. (2020). Evaluation of Transcriptomic Regulations behind Metabolic Syndrome in Obese and Lean Subjects. *Int J Mol Sci, 21*(4). doi:10.3390/ijms21041455

Panel on Antiretroviral Guidelines for Adults and Adolescents. Guidelines for the Use of Antiretroviral Agents in Adults and Adolescents with HIV. Department of Health and Human Services. Available at http://www.aidsinfo.nih.gov/ContentFiles/AdultandAdolescentGL.pdf. Accessed [July, 26 2019].

Park, J., Munagala, I., Xu, H., Blankenship, D., Maffucci, P., Chaussabel, D., . . . Cunningham-Rundles, C. (2013). Interferon signature in the blood in inflammatory common variable immune deficiency. *PLoS One, 8*(9), e74893. doi:10.1371/journal.pone.0074893

Pilia, G., Chen, W. M., Scuteri, A., Orru, M., Albai, G., Dei, M., . . . Schlessinger, D. (2006). Heritability of cardiovascular and personality traits in 6,148 Sardinians. *PLoS Genet, 2*(8), e132. doi:10.1371/journal.pgen.0020132

Pilling, L. C., Joehanes, R., Melzer, D., Harries, L. W., Henley, W., Dupuis, J., . . . Ferrucci, L. (2015). Gene expression markers of age-related inflammation in two human cohorts. *Exp Gerontol, 70*, 37-45. doi:10.1016/j.exger.2015.05.012

Pistis, G., Porcu, E., Vrieze, S. I., Sidore, C., Steri, M., Danjou, F., . . . Sanna, S. (2015). Rare variant genotype imputation with thousands of study-specific whole-genome sequences: implications for cost-effective study designs. *Eur J Hum Genet, 23*(7), 975-983. doi:10.1038/ejhg.2014.216

Rothman, K. J. (1990). No adjustments are needed for multiple comparisons. *Epidemiology, 1*(1), 43-46. Retrieved from https://www.ncbi.nlm.nih.gov/pubmed/2081237

Saul, D., Kosinsky, R. L., Atkinson, E. J., Doolittle, M. L., Zhang, X., LeBrasseur, N. K., . . . Khosla, S. (2022). A new gene set identifies senescent cells and predicts senescence-associated pathways across tissues. *Nat Commun, 13*(1), 4827. doi:10.1038/s41467-022-32552-1

Sherman, B. T., Hao, M., Qiu, J., Jiao, X., Baseler, M. W., Lane, H. C., . . . Chang, W. (2022). DAVID: a web server for functional enrichment analysis and functional annotation of gene lists (2021 update). *Nucleic Acids Res, 50*(W1), W216-W221. doi:10.1093/nar/gkac194

Silva-Aycaguer, L. C., Suarez-Gil, P., & Fernandez-Somoano, A. (2010). The null hypothesis significance test in health sciences research (1995-2006): statistical analysis and interpretation. *BMC Med Res Methodol, 10*, 44. doi:10.1186/1471-2288-10-44

Smith, A. M., Harper, N., Meunier, J. A., Branum, A. P., Jimenez, F., Pandranki, L., . . . Ahuja, S. K. (2021). Repetitive aeroallergen challenges elucidate maladaptive epithelial and inflammatory traits that underpin allergic airway diseases. *J Allergy Clin Immunol*. doi:10.1016/j.jaci.2021.01.008

Sparks, R., Rachmaninoff, N., Lau, W. W., Hirsch, D. C., Bansal, N., Martins, A. J., . . . Tsang, J. S. (2024). A unified metric of human immune health. *Nat Med, 30*(9), 2461-2472. doi:10.1038/s41591-024-03092-6

Stuart, T., Butler, A., Hoffman, P., Hafemeister, C., Papalexi, E., Mauck, W. M., 3rd, . . . Satija, R. (2019). Comprehensive Integration of Single-Cell Data. *Cell, 177*(7), 1888-1902 e1821. doi:10.1016/j.cell.2019.05.031

Tan, C. W., Chia, W. N., Qin, X., Liu, P., Chen, M. I., Tiu, C., . . . Wang, L. F. (2020). A SARS-CoV-2 surrogate virus neutralization test based on antibody-mediated blockage of ACE2-spike protein-protein interaction. *Nat Biotechnol, 38*(9), 1073-1078. doi:10.1038/s41587-020-0631-z

Tan, L., Wang, Q., Zhang, D., Ding, J., Huang, Q., Tang, Y. Q., . . . Miao, H. (2020). Lymphopenia predicts disease severity of COVID-19: a descriptive and predictive study. *Signal Transduct Target Ther, 5*, 33. doi:10.1038/s41392-020-0148-4

Taylor, S. C., Hurst, B., Charlton, C. L., Bailey, A., Kanji, J. N., McCarthy, M. K., . . . Knight, V. (2021). A New SARS-CoV-2 Dual-Purpose Serology Test: Highly Accurate Infection Tracing and Neutralizing Antibody Response Detection. *J Clin Microbiol, 59*(4). doi:10.1128/JCM.02438-20

Waudby-West, R., Parcell, B. J., Palmer, C. N. A., Bell, S., Chalmers, J. D., & Siddiqui, M. K. (2021). The association between SARS-CoV-2 RT-PCR cycle threshold and mortality in a community cohort. *Eur Respir J, 58*(1). doi:10.1183/13993003.00360-2021

Woods, C. W., McClain, M. T., Chen, M., Zaas, A. K., Nicholson, B. P., Varkey, J., . . . Ginsburg, G. S. (2013). A host transcriptional signature for presymptomatic

detection of infection in humans exposed to influenza H1N1 or H3N2. *PLoS One, 8*(1), e52198. doi:10.1371/journal.pone.0052198
Zhai, Y., Franco, L. M., Atmar, R. L., Quarles, J. M., Arden, N., Bucasas, K. L., . . . Couch, R. B. (2015). Host Transcriptional Response to Influenza and Other Acute Respiratory Viral Infections--A Prospective Cohort Study. *PLoS Pathog, 11*(6), e1004869. doi:10.1371/journal.ppat.1004869
